# Application 2D Descriptors and Artificial Neural Networks for Beta-Glucosidase Inhibitors Screening

**DOI:** 10.3390/molecules25245942

**Published:** 2020-12-15

**Authors:** Maciej Przybyłek

**Affiliations:** Department of Physical Chemistry, Pharmacy Faculty, Collegium Medicum of Bydgoszcz, Nicolaus Copernicus University in Toruń, Kurpińskiego 5, 85-950 Bydgoszcz, Poland; m.przybylek@cm.umk.pl; Tel.: +48-52-585-3678

**Keywords:** beta-glucosidase, enzyme inhibitors, virtual screening, 2D molecular descriptors, binary classification, neural networks

## Abstract

Beta-glucosidase inhibitors play important medical and biological roles. In this study, simple two-variable artificial neural network (ANN) classification models were developed for beta-glucosidase inhibitors screening. All bioassay data were obtained from the ChEMBL database. The classifiers were generated using 2D molecular descriptors and the data miner tool available in the STATISTICA package (STATISTICA Automated Neural Networks, SANN). In order to evaluate the models’ accuracy and select the best classifiers among automatically generated SANNs, the Matthews correlation coefficient (MCC) was used. The application of the combination of maxHBint3 and SpMax8_Bhs descriptors leads to the highest predicting abilities of SANNs, as evidenced by the averaged test set prediction results (MCC = 0.748) calculated for ten different dataset splits. Additionally, the models were analyzed employing receiver operating characteristics (ROC) and cumulative gain charts. The thirteen final classifiers obtained as a result of the model development procedure were applied for a natural compounds collection available in the BIOFACQUIM database. As a result of this beta-glucosidase inhibitors screening, eight compounds were univocally classified as active by all SANNs.

## 1. Introduction

Glucosidases belong to a wide class of hydrolytic enzymes. Some well-known examples are alpha- and beta-glucosidase, cellulase, lactase, mannosyl-oligosaccharide glucosidase, and pullulanase. These biocatalysts are a very diverse group of molecules, playing different biochemical roles. Among many of them, three major functions can be distinguished, namely polysaccharides hydrolysis, glycolipids and glycoproteins synthesis, and glycoprotein metabolism [1,2,3]. The common feature of both alpha- and beta-glucosidase is the presence of nucleophilic and proton donor sites [1,4,5,6,7]. However, kinetic and structural studies showed that there are significant differences between transitional states [1]. In the case of both enzymes, glycosidic bond hydrolysis proceeds via oxocarbenium ion formation [1,4,5,6,7,8]. The differences in mechanisms are related to the orientation of the anomeric carbon atom interacting with the nucleophilic site i.e., carboxyl oxygens [1,7,8,9]. Beta-glucosidases can be found in many living organisms like bacteria (*Caldocellum saccharolyticum* [10], *Proteus mirabilis* [11], *Thermotoga petrophila* [12]), fungi (*Trichoderma* [13], *Trichoderma reesei*, several *Aspergillus* species [14]), plants (*Prunus dulcis* [15,16]), *Sorghum bicolor* L. Moench [17], *Plantago lanceolate* [18], *Trifolium repens* L. [19]) and animals (mammals [20,21,22], birds [23], and fish [24]). This biocatalyst enables the hydrolysis of beta-glycosidic moieties in oligo- or disaccharides, cyanogenic glucosides, and various β-d-glucoside derivatives (alkyl-, aryl-, and amino-β-d-glucosides) [25,26].

Glucosidase inhibitors are interesting from several viewpoints. The common feature of this group is the presence of both hydrogen bonds donors and acceptors, its hydrophobic nature, and backbone flexibility [27]. In general, glucosidase inhibitors can be divided into two major categories—glycosidic compounds, such as saccharides and their analogues (thiosugars, iminosugars, carbasugars) and non-glycosidic compounds [1,28]. These compounds affect important metabolic pathways and their pharmacological applications including obesity, diabetes, hyperlipoproteinemia, cancer, HBV, HCV, and HIV treatment were documented [1,29,30,31,32]. Furthermore, glucosidase inhibitors have been applied for investigating the biochemical paths of various metabolic processes [1,33,34]. From the pharmacological viewpoint, human liposomal glucosidase inhibitors deserve special attention, since these compounds exhibit beneficial effects on the lysosomal storage disorders treatment (Gaucher disease) [35,36,37].

Nowadays, the inhibiting properties can be easily obtained from various sources like the ChEMBL (https://www.ebi.ac.uk/chembl/) [38,39] and PubChem (https://pubchem.ncbi.nlm.nih.gov/) [40] databases. These ligands’ libraries along with molecular descriptor calculations allow for developing useful and effective QSAR/QSPR (quantitative structure-activity relationship/quantitative structure-property relationship) models. The main purpose of this study is to develop a simple and efficient classifier utilizing 2D indices for beta-glucosidase inhibitors. The choice of these descriptors was guided by their low computational cost, since these parameters can be computed using only molecular structure represented by the Simplified Molecular Input Line Entry Specification (SMILES) code. Noteworthy model efficiency is particularly important from the computer-aided drug design perspective, due to the possibility of screening thousands of compounds in a short period of time. This purpose is in general more difficult to accomplish using time-consuming computational methods based on molecular dynamics or quantum-chemical calculations. Furthermore, many studies showed the great usefulness of 2D structure-derived features in the modeling of physicochemical properties [41,42,43,44,45,46,47,48,49,50]. In this study, 2D molecular descriptors, calculated for a large dataset built with the aid of available beta-glucosidase inhibition bioassays results, were used to generate artificial neural networks (ANNs) classifiers. Due to their high accuracy, non-linear methods have found wide application in biological activities and the modelling of physicochemical properties. However, the use of these techniques including ANNs is often associated with the risk of the overfitting problem. Therefore, it is reasonable to create the simplest models containing the smallest possible number of variables, which was also taken into account when constructing the model presented in this paper.

## 2. Results

### 2.1. Descriptors Selection

Due to the very large number of descriptors which can be efficiently computed using various tools such as PaDEL [51], it is necessary to make an appropriate molecular features selection. Therefore, prior to the machine learning procedure, the set of the most suitable descriptors according to the χ^2^ ranking method was selected. This method has been implemented in STATISTICA for automatic descriptor selection and is part of the Data Miner module. It is worth noting that the χ^2^ method and other similar methods of feature selection have been widely used in QSPR/QSAR problem solving including artificial neural networks classifiers [52,53,54,55,56,57].

**Figure 1:**
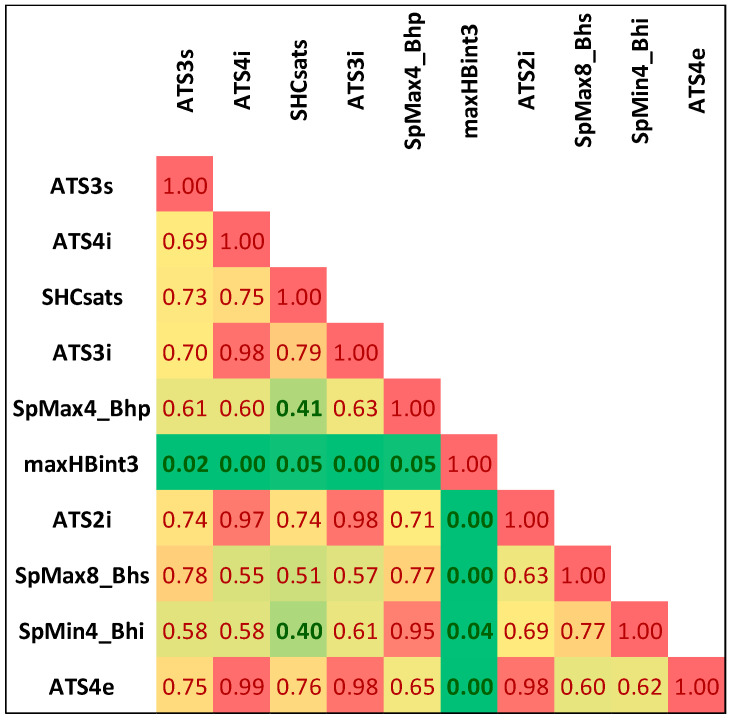
The correlation matrix of selected descriptors.

**Figure 2:**
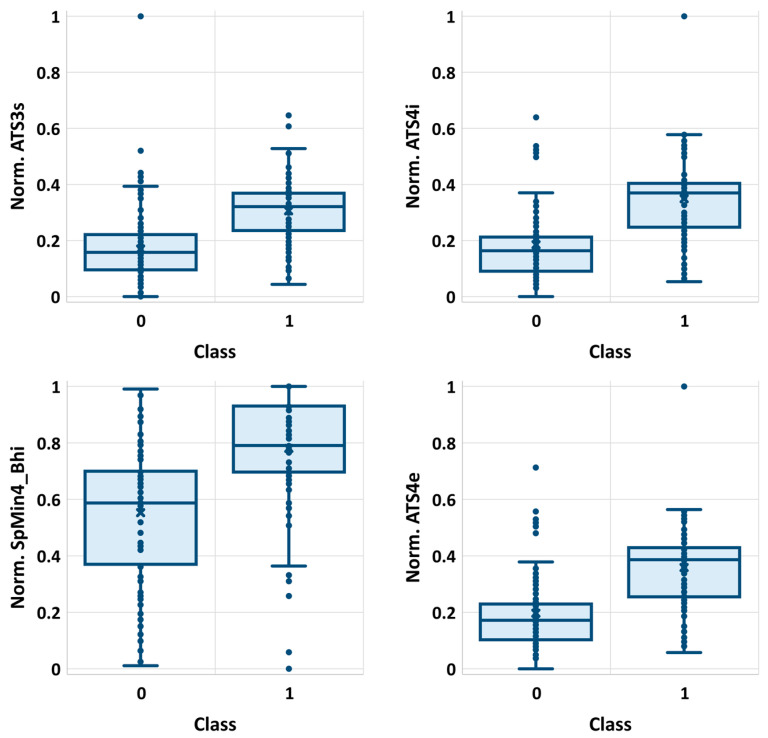
The distributions of four exemplary descriptors (normalized values) characterized by the highest (ATS3s, ATS4i) and the lowest (SpMin4_Bhi, ATS4e) χ^2^ values.

Noteworthily, it happens that many of the selected features are strongly correlated with each other. The list of selected descriptors was summarized in Table 1, while in Figure 1, the correlation matrix was provided. There are significant statistical differences between selected molecular descriptors distributions corresponding to class 0 and class 1 populations, as evidenced by very low *p*-values (Table 1). These differences can be visualized on boxplots corresponding to descriptors characterized by the highest and the lowest χ^2^ values (Figure 2). As can be seen, even the lowest ranked descriptors quite clearly distinguish active and non-active cases. This high ability of individual descriptors to separate class 0 and class 1 populations is important for prevention of the overfitting problem, since a high quality of prediction can be achieved using a low number of variables. Therefore, each STATISTICA Automated Neural Network (SANN) model developed in this study involves only one orthogonal pair of descriptors (R^2^ < 0.5) in the input layer.

The selected descriptors should reflect the relevant physicochemical features important for a particular QSAR task. However, very often the interpretation of molecular features is not clear. Among the selected descriptors, many typical QSAR parameters, such as Burden-modified eigenvalues (SpMax4_Bhp, SpMin4_Bhi, SpMax8_Bhs) [58,59,60], Broto-Moreau autocorrelation (ATS3s, ATS4e, ATS4i, ATS2i) [58,61,62,63,64], and atom-type electrotopological state (SHCsats, maxHBint3) indices [65,66,67,68,69], can be found. Noteworthily, several of them are associated with the relevant intermolecular interactions’ perspective features such as electronegativity (ATS4e), polarizabilities (SpMax4_Bhp), first ionization potential (SpMin4_Bhi, ATS4i, ATS2i), and intrinsic state (SpMax8_Bhs, ATS3s). The enzymes’ inhibition is determined by the ligand-active center interaction’s nature related to the molecules’ polarity. However, the “global” polarity does not provide sufficient information, since the molecule being an effective inhibitor often contains both polar and non-polar fragments interacting with the appropriate sites in the biocatalyst. The appearance of the SHCsats descriptor in QSAR models, defined as the hydrogen E-states sum on the sp^3^-hybridized carbon atom of the saturated bond, reflects the weak interactions with non-polar sites in the biomolecular targets [70,71]. It is worth noting that many of the active compounds employed for model development are typical Gaucher treatment agents containing a hydrocarbon chain. The presence of both polar (piperidine, pyrrolidine, and pyrrolizidine iminosugar analogues) and non-polar hydrocarbon moieties (e.g., miglustat, CHEMBL1029) in the active ingredients used for the treatment of lysosomal storage disorders is strictly associated with the specific interaction of these compounds with glucocerebrosidase (human acid β-glucosidase) active centers [72,73].

### 2.2. The SANN Models

In this study, several well-known parameters, such as percentage of properly classified cases (%All), Matthews correlation coefficient (MCC), and area under the receiver operating characteristic (ROC) curve AUC_ROC_, were used to analyze the obtained models. The concept of a popular classifiers quality measure, namely MCC, is based on Pearson’s correlation analysis adapted for binary results [74,75]. Hence, MCC characterizes the quality of a correlation between estimated and actual data. In the case of a perfect classifier, the MCC is 1, while in the case of the worst model, MCC is −1. It is worth noting that in this study, a tenfold division into a training, validation, and test set was carried out, which enables a reliable selection of descriptor pairs characterized by the highest predicting power. Since, as a result of each machine learning step performed for a particular data split, the top five SANNs were saved, the total number of classifiers for each descriptor pair was 50. The predicting abilities of descriptor pairs can be evaluated based on the average parameters calculated for the test sets’ predictions. Noteworthily, in this study, the SANN classifiers were externally tested, which confirms the reliability of this analysis. As it can be inferred from Table 2, all two-variable models proved to be characterized by a very high accuracy. In the case of most classifiers, MCC values are higher than 0.7, which is characteristic of very strong positive correlations [76]. The AUC_ROC_ values, which are in general very close to 1, also suggest good performance of the models. It is noteworthy that the AUC_ROC_ parameter has been very commonly applied as a useful machine learning tool [77,78,79,80]. It is not clear which of the AUC_ROC_ or MCC parameters are more appropriate to assess the accuracy of the model since there are critical opinions on both criterions [81,82,83,84,85]. Taking into account all three criterions (MCC, AUC_ROC_ and %All) determined for all SANNs, pair 1 (maxHBint3, SpMax8_Bhs) appears to exhibit the highest predicting abilities. Although pair 3 (maxHBint3, SpMin4_Bhi) is characterized by a slightly higher AUC_ROC_ value, the other two parameters (MCC, %All) indicate a significant advantage of pair 1. Therefore, the models employing this pair of descriptors were further analyzed. Among them, classifiers exhibiting the highest MCC values calculated for the test set were selected.

The selected models’ details including neural net architecture, structural features, and predictions are summarized in Table 3. Although ten dataset splits were performed, the number of SANNs is thirteen, since for splits 1, 5, and 9, the highest accuracy expressed by the MCC was achieved in the case of two different classifiers. These models were saved in the universal Predictive Model Markup Language (PMML) format (supplementary SANNs.zip file), which allows for their implementation. As can be inferred from Table 3, in most cases, the redundant Byzantine fault tolerance (RBFT) learning algorithm was applied during the SANN procedure. Another algorithm was based on the Broyden-Fletcher-Goldfarb-Shanno (BFGS) approach. In most cases, except two models, the entropy was used as an error function. During the machine learning steps, both multilayer perceptron (MLP) and radial basis function (RBF) networks were allowed to be generated. Due to the SANN methodology limitations, in all cases, only one hidden layer was applied. As it can be inferred from Table 3, the number of neurons in the hidden layer ranges from 4 to 30. Taking into account the relatively high number of instances in the training set (*N* = 228), the complexity of the SANNs seems to be quite low. In the case of most dataset splits, the RBF networks were preferred.

**Figure 3:**
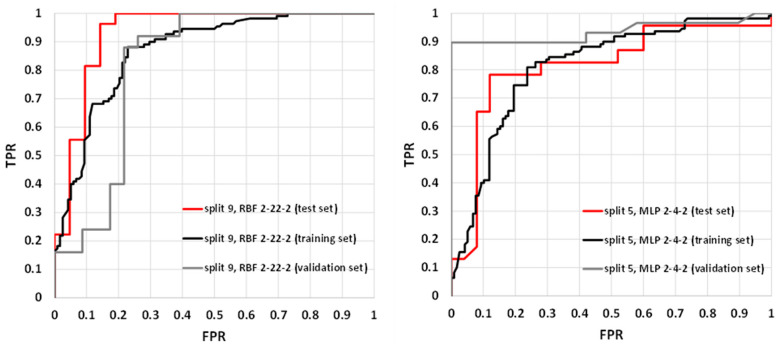
Receiver operating characteristics (ROC) plots for RBF 2-22-2 (split 9) and MPL 2-4-2 (split 5) models.

**Figure 4:**
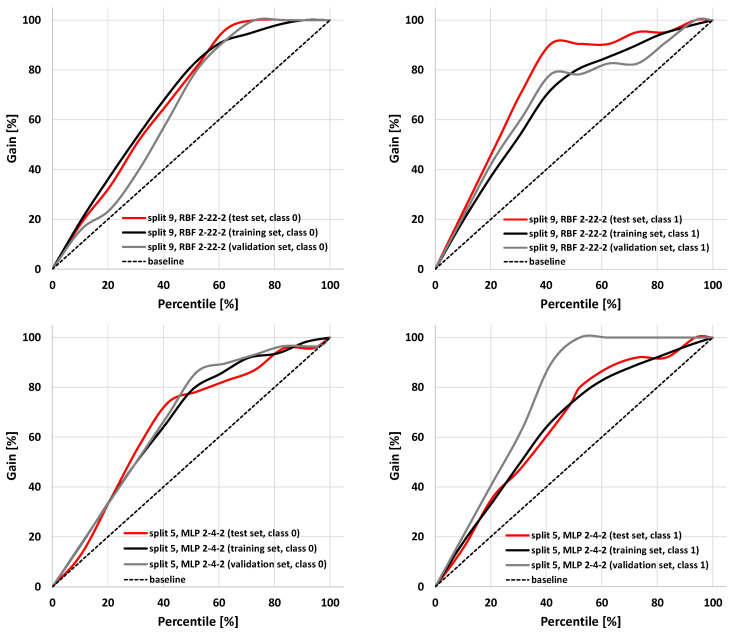
Cumulative gain charts for RBF 2-22-2 (split 9) and MPL 2-4-2 (split 5) models.

The parameters characterizing summary prediction ability are clearly not sufficient to fully describe the model. Therefore, it is worth analyzing the relationships showing the whole population. The exemplary ROC and cumulative gain charts for SANNs characterized by the highest (split 9, RBF 2-22-2) and the lowest MCC values calculated for the test set (split 5, MLP 2-4-2) are summarized in Figure 3 and Figure 4. As one can see, in both cases, fairly typical shapes of ROC and gain charts characteristic of high classification quality can be observed. In the case of the ROC plot, the good performance of the model is reflected by the high sensitivity observed for low false-positives rate values. In the case of a gain chart, good classification quality is reflected by a significant distance from the baseline corresponding to the random classifier. As can be inferred from each gain chart, in the case of both exemplary classifiers, there are noticeable differences between class 0 and class 1 (Figure 4). The overall accuracy can be illustrated by ROC curves. In the case of the best classifier, the test set is characterized by a much steeper trend than in the case of the training and validation sets. The AUC_ROC_ values for training, validation, and test sets are equal to 0.869, 0.819, and 0.931, respectively. It is worth noting that the higher fraction of properly classified cases in the test set than in the training set indicates the optimal model complexity. This can be explained by the fact that in the case of overfitted classifiers, the training set cases are well reproduced in contrast to the externally excluded ones. Additionally, in the case of the model characterized by the lowest accuracy among selected SANNs, analysis of the ROC charts indicates the optimal complexity of the neural network. In the case of this model, the AUC_ROC_ values corresponding to the training, validation, and test sets are 0.812, 0.935, and 0.813, respectively. This indicates similar training and test set prediction quality. On the other hand, in the case of the validation set, a much steeper plot can be observed, resulting in a higher AUC_ROC_ value. This high quality of prediction can also be observed in the case of the gain chart (Figure 4). As it can be inferred, this is mainly caused by the high properly assigned active compounds score (class 1). The role of the validation set is associated with model selection during the SANN machine learning process. Therefore, it is somewhat involved in the development of the model, and hence, the external test set is more appropriate for the accuracy evaluation. Nevertheless, a satisfactory result for the validation sets can also be regarded as important information. Fortunately, in most cases, the MCC values calculated for the validation set are higher than 0.7 (Table 3).

From the model validation perspective, it is important to develop a classifier characterized by the application domain, which comprises the range of descriptors values calculated for the test set. This is very important since the applicability domain determines the area in which the obtained results are reliable. According to common practice, the applicability domain is determined by the training set descriptors’ values ranges. However, because in the case of SANN, the validation set is also involved in the machine learning process, it seems to be reasonable to assume the total ranges corresponding to these two sets as the applicability domain. As one can see from Table 4, the range of test sets for both the maxHBint3 and SpMax8_Bhs descriptors in most cases does not exceed or only slightly exceeds the applicability domain (Table 4). Nevertheless, the comparability of these ranges is not sufficient. It is understandable that both populations used for model development and external validation ought to be statistically similar [86,87,88]. As was established, training sets do not differ significantly from the corresponding test sets, since in most cases, the *p*-values calculated using two different statistical tests (Mann-Whitney U and Kolmogorov-Smirnov tests) are higher than 0.1.

### 2.3. Application of SANNs

**Figure 5:**
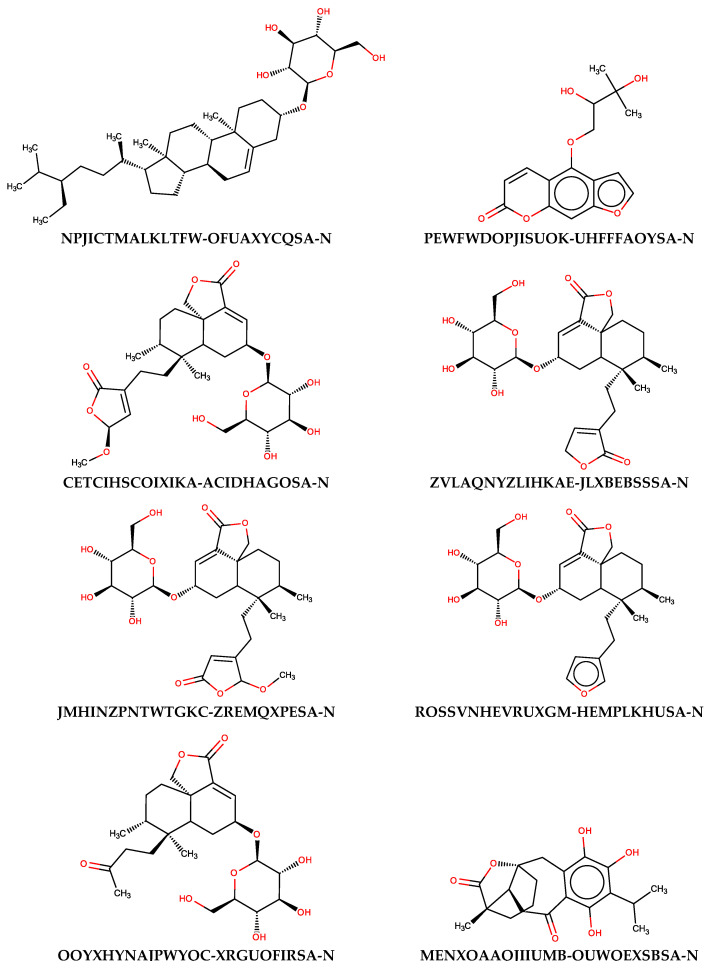
The structures of natural compounds along with the InChIKey codes found in the BIOFACQUIM database, which were classified as active beta-glucosidase inhibitors by all SANNs employing maxHBint3 and SpMax8_Bhs descriptors.

To evaluate the usefulness of the generated models, additional predictions were performed with the use of a set of naturally occurring compounds. The BIOFACQUIM (https://biofacquim.herokuapp.com/) database containing 423 compounds represented by SMILES codes was selected for this purpose. This database was co-created by four countries (Brazil, France, Panama, Vietnam) with the intention of providing a diverse set of compounds useful for various chemometric purposes, including data mining and drug design [89]. In Figure 5, the potential beta-glucosidase inhibitors selected using thirteen SANN models employing maxHBint3 and SpMax8_Bhs descriptors were presented. The results of the prediction were summarized in Table S3. As a result of such in silico screening, eight compounds were selected. Of course, many more compounds can be classified as active when less restrictive criteria will be used. If the criterion of assignment by the majority of SANNs is taken into account, 49 compounds can be distinguished as being potential inhibitors.

As can be noticed from Figure 5, the majority of compounds contain characteristic carbohydrate moieties. The presence of substrate mimicking structural features, such as iminosugar rings, is characteristic for beta-glucosidase inhibitors. Indication of such compounds by SANNs, from the set of various structures available in the BIOFACQUIM database, additionally confirms their usefulness. It is noteworthy that the maxHBint3 and SpMax8_Bhs values corresponding to the compounds assigned by the majority of SANNs as active were within the range of applicability domain determined by the training and validation sets (Table 4). Furthermore, only 16 outliners, among 423 records, can be found when all SANNs are considered.

## 3. Methods

### 3.1. Dataset Selection and Pretreatment

All data used in this study were obtained from the ChEMBL database (https://www.ebi.ac.uk/chembl/) [38,39], which is a comprehensive tool allowing researchers to obtain bioactivity information of approved pharmaceuticals and drug-like compounds [38]. Among collected data, only those records for which IC_50_ (half maximal inhibitory concentration) information could be found were taken into account. When more than one IC_50_ value was available for a particular compound, the arithmetic mean was taken into account. The compounds were classified as low-active or inactive (class 0, IC_50_ > 50 μM) and significantly active (class 1, IC_50_ ≤ 50 μM). This is an arbitrary criterion; however, the 50 μM threshold has been widely used for evaluating various biological activities including enzymes inhibitors [90,91,92,93,94,95]. All data used for model development and validation are summarized in the Supplementary Materials (Table S1).

### 3.2. Descriptors Calculation

The molecular descriptors were calculated using PaDEL software [51]. As the input file, a list of SMILES codes obtained from the ChEMBL database were used. Among the 1444 available 1D and 2D indices, 293 were removed, since they were not calculable for all of the dataset’s objects or because of zero variance.

### 3.3. The Artificial Neutral Networks Models

In this study, a popular and universal STATISTICA Automated Neural Networks (SANNs) approach was applied. Prior to the models’ generation, descriptors’ predicting abilities were evaluated using the χ^2^ test. Based on this analysis, ten selected molecular features characterized by the highest χ^2^ were further applied for SANN classifiers generation. All computations including variables selection, model development, and validation were performed automatically using datamining tools available in STATISTICA12 software [96]. During each machine learning procedure, both multilayer perceptron (MLP) and radial basis function (RBF) algorithms were taken into account. The number of generated SANNs was set to 1000, from which 5 of the highest predicting ability were selected automatically by the software. In this study, ten random data splits to the training (70%), test (15%), and validation (15%) sets were applied. The model training, validation, and external testing procedure were performed using default STATISTICA settings. All dataset splits were summarized in Supplementary Table S2. The selected models were saved in PMML (Predictive Model Markup Language) format (http://dmg.org/pmml/pmml-v3-0.html) and are available in the Supplementary Materials (SANNs.zip).

### 3.4. Classification Quality Evaluation

In order to evaluate the SANNs’ performance, several well-known statistical measures were applied. A detailed and comprehensive description of the model can be made by analyzing parameters such as the percentage of properly classified cases (%All), the Matthews correlation coefficient (MCC) [74,75], and area under the receiver operating characteristic (ROC) curve (AUC_ROC_). The former two parameters can be directly calculated using the following equations:

(1) $$\%TP=\;\frac{TP}{P}\times 100\%=TPR\times 100\%$$

(2) $$\%FP=\frac{FP}{P}\times 100\%=FPR\times 100\%$$

(3) $$\%TN=\frac{TN}{N}\times 100\%=TNR\times 100\%$$

(4) $$\%FN=\frac{FN}{N}\times 100\%=FNR\times 100\%$$

(5) $$MCC=\frac{TP\times TN-FP\times FN}{\sqrt{\left(TP+FP\right)\left(TP+FN\right)\left(TN+FP\right)\left(TN+FN\right)}}$$

where *TP*, *FP*, *TN*, and *FN* denote the number of true positives, false positives, true negatives, and false negatives, respectively. The *P* and *N* stand for all positive or negative cases, while the *%TP*, *%FP*, *%TN*, *%FN*, *TPR*, *FPR*, *TNR*, and *FNR* parameters are the percentages or rates of true positives, false positives, true negatives, and true positives. The AUC_ROC_ parameter is determined based on the ROC curve which is the relationship between sensitivity expressed by the *TPR* and 1-specificity term equal to *FPR*.

## 4. Conclusions

The screening of new biologically active compounds requires performing various time-consuming and expensive experiments including synthesis of new compounds and bioassays. Therefore, theoretical models are particularly useful, since they can direct the experimental effort to a selected group of compounds, which are expected to be active. Therefore, accuracy, effectiveness, and simplicity are of a significant importance when developing new models. Beta-glucosidase inhibitors are an important class of compounds, widely used in medicine due to their beneficial effects on health. Probably, the main application of beta-glucosidase inhibitors is the treatment of lysosomal storage disorders including Gaucher disease. In this paper, an accurate QSAR beta-glucosidase classification model based on simple and intuitive methodology was developed. The models described in this study were developed and validated using a universal SANN methodology implemented in STATISTICA. Prior to the SANN models’ generation, a variables selection procedure was performed, which resulted in the collection of orthogonal pairs of descriptors. As it was established, the combination of maxHBint3 and SpMax8_Bhs indices leads to the highest accuracy expressed by the highest average MCC value calculated for ten random dataset splits. This simple and intuitive concept of model development seems to be promising in the case of other enzymes’ inhibitors. It is noteworthy that the application of the final SANNs for a collection of natural compounds available in the BIOFACQUIM database resulted in the selection of eight compounds univocally classified as active by all models. Most of these compounds contain carbohydrate moieties, which is characteristic for a substrate mimicking glucosidase inhibitors.

## Figures and Tables

**Table 1:** The list of selected descriptors with the aid of the χ^2^ ranking approach. The analysis was augmented with Mann-Whitney U and Kolmogorov-Smirnov tests results.

Rank	Descriptor	χ^2^	*p*-Value		
			χ^2^ Test	Mann-Whitney U	Kolmogorov-Smirnov
1	ATS3s	124.6913	0.00	0.00	<0.001
2	ATS4i	124.4754	0.00	0.00	<0.001
3	SHCsats	122.8091	0.00	0.00	<0.001
4	ATS3i	122.4608	0.00	0.00	<0.001
5	SpMax4_Bhp	121.9436	0.00	0.00	<0.001
6	maxHBint3	121.1082	0.00	0.00	<0.001
7	ATS2i	120.4616	0.00	0.00	<0.001
8	SpMax8_Bhs	120.3141	0.00	0.00	<0.001
9	SpMin4_Bhi	119.4392	0.00	0.00	<0.001
10	ATS4e	118.9435	0.00	0.00	<0.001

**Table 2:** The analysis of orthogonal pairs of descriptors used in models (external test set prediction results). All data are the average values calculated for 50 different STATISTICA Automated Neural Networks (SANNs). In the parentheses, the standard deviation values were provided.

No	Descriptor Pairs	%TN	%TP	%All	MCC	AUC_ROC_
1	maxHBint3, SpMax8_Bhs	89.0(6.7)	81.4(5.7)	85.3(4.3)	0.748(0.063)	0.860(0.059)
2	maxHBint3, SpMax4_Bhp	89.3(8.8)	77.3(9.0)	83.4(5.3)	0.722(0.070)	0.854(0.05)
3	maxHBint3, SpMin4_Bhi	81.5(8.1)	84.0(10.0)	82.4(4.5)	0.715(0.063)	0.868(0.055)
4	maxHBint3,SHCsats	86.9(8.0)	78.6(7.2)	82.8(4.7)	0.715(0.057)	0.847(0.056)
5	SHCsats, SpMax4_Bhp	83.0(8.6)	81.5(8.4)	82.2(5.2)	0.711(0.069)	0.853(0.057)
6	maxHBint3,ATS4i	86.1(9.1)	78.1(12.1)	82.4(6.7)	0.710(0.093)	0.839(0.089)
7	maxHBint3,ATS3i	86.2(9.0)	78.5(7.4)	82.4(4.5)	0.710(0.062)	0.848(0.052)
8	SHCsats, SpMin4_Bhi	79.9(9.8)	85.0(7.4)	82.0(4.6)	0.710(0.059)	0.847(0.076)
9	maxHBint3,ATS3s	83.9(9.6)	78.2(9.1)	80.9(5.7)	0.696(0.076)	0.818(0.071)
10	maxHBint3,ATS4e	85.3(8.2)	76.6(7.9)	80.7(5.2)	0.693(0.060)	0.816(0.078)
11	maxHBint3,ATS2i	83.6(6.7)	71.7(9.9)	77.6(5.4)	0.654(0.061)	0.794(0.088)

**Table 3:** The selected details of SANNs developed employing maxHBint3 and SpMax8_Bhs descriptors. The models were generated using ten different dataset splits (Tr, V, and Ts denote the training, validation, and test sets respectively).

Data Split	SANN	Learning Algorithm	Error Function	Activation Function		MCC		
				Hidden Layer	Output Layer	Tr	V	Ts
1	RBF 2-24-2	RBFT	Entropy	Gauss	Softmax	0.698	0.748	0.745
1	RBF 2-22-2	RBFT	Entropy	Gauss	Softmax	0.733	0.748	0.745
2	RBF 2-27-2	RBFT	Entropy	Gauss	Softmax	0.739	0.813	0.813
3	MLP 2-4-2	BFGS 22	Entropy	Tanh	Softmax	0.693	0.722	0.798
4	RBF 2-24-2	RBFT	Entropy	Gauss	Softmax	0.734	0.660	0.742
5	MLP 2-4-2	BFGS 7	Entropy	Exponential	Softmax	0.603	0.745	0.720
5	RBF 2-22-2	RBFT	Entropy	Gauss	Softmax	0.633	0.742	0.722
6	MLP 2-5-2	BFGS 33	Entropy	Exponential	Softmax	0.678	0.847	0.781
7	MLP 2-4-2	BFGS 42	Entropy	Tanh	Softmax	0.698	0.589	0.778
8	RBF 2-27-2	RBFT	Sum of squares	Gauss	Linear	0.705	0.809	0.811
9	RBF 2-22-2	RBFT	Sum of squares	Gauss	Linear	0.728	0.747	0.918
9	RBF 2-28-2	RBFT	Entropy	Gauss	Softmax	0.667	0.750	0.918
10	RBF 2-30-2	RBFT	Entropy	Gauss	Softmax	0.700	0.720	0.769

**Table 4:** The analysis maxHBint3 and SpMax8_Bhs descriptor ranges and distributions.

Data Split	Dataset	MaxHBint3		*p*-Value		SpMax8_Bhs		*p*-Value	
		Min	Max	U ^1^	KS ^2^	Min	Max	U ^1^	KS ^2^
1	training and validation	0.0000	6.8353	0.92	>0.1	1.2027	4.7812	0.87	>0.1
	test set	0.0000	6.5696			0.7059	4.7447		
2	training and validation	0.0000	6.5696	0.62	>0.1	0.7059	4.7812	0.73	>0.1
	test set	0.0000	6.8353			1.2907	4.0981		
3	training and validation	0.0000	6.8353	0.10	>0.1	0.7059	4.6921	0.17	<0.1
	test set	0.0000	5.0901			1.2907	4.7812		
4	training and validation	0.0000	6.5696	0.95	>0.1	0.7059	4.7812	0.31	>0.1
	test set	0.0000	6.8353			1.2907	4.6316		
5	training and validation	0.0000	6.8353	0.97	>0.1	1.2027	4.7812	0.43	>0.1
	test set	0.0000	5.5066			0.7059	4.0927		
6	training and validation	0.0000	6.5696	0.27	>0.1	0.7059	4.7812	0.23	>0.1
	test set	0.0000	6.8353			1.4435	4.6613		
7	training and validation	0.0000	6.8353	0.89	>0.1	1.2027	4.7812	0.54	>0.1
	test set	0.0000	5.5717			0.7059	4.6921		
8	training and validation	0.0000	6.8353	0.63	>0.1	0.7059	4.7812	0.59	>0.1
	test set	0.0000	5.2672			1.6043	4.0981		
9	training and validation	0.0000	6.8353	0.69	>0.1	0.7059	4.7812	0.26	>0.1
	test set	0.0000	5.1510			1.6609	4.2558		
10	training and validation	0.0000	6.8353	0.11	>0.1	0.7059	4.6921	0.31	>0.1
	test set	0.0000	5.5574			1.2027	4.7812		

^1^ Mann-Whitney U test. ^2^ Kolmogorov-Smirnov test.

## Supplementary Materials

The following are available online, Table S1: The dataset used for model development and validation obtained from ChEMBL database, Table S2: The dataset splits into training, validation and test sets, Table S3: The results of prediction performed for BIOFACQUIM database, SANNs.zip file containing PMML codes of SANNs.

## References

[1] De Melo B.E., Da Silveira G.A., Carvalho I. (2006). α- and β-Glucosidase inhibitors: Chemical structure and biological activity. Tetrahedron.

[2] Campo V.L., Aragão-Leoneti V., Carvalho I. (2013). Glycosidases and diabetes: Metabolic changes, mode of action and therapeutic perspectives. Carbohydrate Chemistry.

[3] Bieberich E., Yu R., Schengrund C.L. (2014). Synthesis, Processing, and Function of *N*-glycans in *N*-glycoproteins. Glycobiology of the Nervous System. Advances in Neurobiology.

[4] Heightman T.D., Vasella A.T. (1999). Recent Insights into Inhibition, Structure, and Mechanism of Con-figuration-Retaining Glycosidases. Angew. Chem. Int. Ed..

[5] Krasikov V.V., Karelov D.V., Firsov L.M. (2001). α-Glucosidases. Biochemistry.

[6] Lillelund V.H., Jensen H.H., Liang X., Bols M. (2002). Recent Developments of Transition-State Analogue Glycosidase Inhibitors of Non-Natural Product Origin. Chem. Rev..

[7] Legler G. (1990). Glycoside Hydrolases: Mechanistic Information from Studies with Reversible and Irre-versible Inhibitors. Adv. Carbohydr. Chem. Biochem..

[8] Chiba S. (1997). Molecular Mechanism in α-Glucosidase and Glucoamylase. Biosci. Biotechnol. Biochem..

[9] Piszkiewicz D., Bruice T.C. (1968). Glycoside Hydrolysis. II. Intramolecular Carboxyl and Acetamido Group Catalysis in β-Glycoside Hydrolysis. J. Am. Chem. Soc..

[10] Bauer M.W., Bylina E.J., Swanson R.V., Kelly R.M. (1996). Comparison of a β-Glucosidase and a β-Mannosidase from the Hyperthermophilic ArchaeonPyrococcus furiosus. J. Biol. Chem..

[11] Mahapatra S., Vickram A.S., Sridharan T.B., Parameswari R., Pathy M.R. (2016). Screening, production, optimization and characterization of β-glucosidase using microbes from shellfish waste. 3 Biotech.

[12] Zhang S., Xie J., Zhao L., Pei J., Su E., Xiao W., Wang Z. (2019). Cloning, overexpression and character-ization of a thermostable β-xylosidase from Thermotoga petrophila and cooperated transformation of ginsenoside extract to ginsenoside 20(S)-Rg3 with a β-glucosidase. Bioorg. Chem..

[13] Tiwari P., Misra B.N., Sangwan N.S. (2013). β-Glucosidases from the FungusTrichoderma: An Efficient Cellulase Machinery in Biotechnological Applications. BioMed Res. Int..

[14] Sørensen A., Lübeck M., Lubeck P.S., Ahring B.K. (2013). Fungal Beta-Glucosidases: A Bottleneck in Industrial Use of Lignocellulosic Materials. Biomolecules.

[15] Del Cueto J., Møller B.L., Dicenta F., Sánchez-Pérez R. (2018). β-Glucosidase activity in almond seeds. Plant Physiol. Biochem..

[16] Li Y.-K., Chang L.-F., Shu H.-H., Chir J. (1997). Characterization of an Isozyme of β-Glucosidase from Sweet Almond. J. Chin. Chem. Soc..

[17] Cicek M., Esen A. (1998). Structure and Expression of a Dhurrinase (β-Glucosidase) from Sorghum. Plant Physiol..

[18] Pankoke H., Buschmann T., Müller C. (2013). Role of plant β-glucosidases in the dual defense system of iridoid glycosides and their hydrolyzing enzymes in Plantago lanceolata and Plantago major. Phytochemistry.

[19] Barrett T., Suresh C.G., Tolley S.P., Dodson E.J., Hughes M.A. (1995). The crystal structure of a cyanogenic β-glucosidase from white clover, a family 1 glycosyl hydrolase. Structure.

[20] Ioku K., Pongpiriyadacha Y., Konishi Y., Takei Y., Nakatani N., Terao J. (1998). β-Glucosidase Activity in the Rat Small Intestine toward Quercetin Monoglucosides. Biosci. Biotechnol. Biochem..

[21] Raychaudhuri C., Desai I.D. (1972). Lysosomal β-glucosidase and β-xylosidase of rat intestine. Int. J. Biochem..

[22] Gopalan V., Vander Jagt D.J., Libell D.P., Glew R.H. (1992). Transglucosylation as a probe of the mecha-nism of action of mammalian cytosolic β-glucosidase. J. Biol. Chem..

[23] Philip J.S., Gilbert H.J., Smithard R.R. (1995). Growth, viscosity and beta-glucanase activity of intestinal fluid in broiler chickens fed on barley-based diets with or without exogenous beta-glucanase. Br. Poult. Sci..

[24] Lelieveld L.T., Mirzaian M., Kuo C.L., Artola M., Ferraz M.J., Peter R.E.A., Akiyama H., Greimel P., Van den Berg R.J.B.H.N., Overkleeft H.S. (2019). Role of β-glucosidase 2 in aberrant glycosphin-golipid metabolism: Model of glucocerebrosidase deficiency in zebrafish. J. Lipid Res..

[25] Yeoman C.J., Han Y., Dodd D., Schroeder C.M., Mackie R.I., Cann I.K.O. (2010). Thermostable enzymes as biocatalysts in the biofuel industry. Adv. Appl. Microbiol..

[26] Asati V., Sharma P.K. (2019). Purification and characterization of an isoflavones conjugate hydrolyzing β-glucosidase (ICHG) from Cyamopsis tetragonoloba (guar). Biochem. Biophys. Rep..

[27] Amiri B., Hosseini N.S., Taktaz F., Amini K., Rahmani M., Amiri M., Sadrjavadi K., Jangholi A., Esmaeili S. (2019). Inhibitory effects of selected antibiotics on the activities of α-amylase and α-glucosidase: In-vitro, in-vivo and theoretical studies. Eur. J. Pharm. Sci..

[28] Martínez-Bailén M., Jiménez-Ortega E., Carmona A.T., Robina I., Sanz-Aparicio J., Talens-Perales D., Polaina J., Matassini C., Cardona F., Moreno-Vargas A.J. (2019). Structural basis of the inhibition of GH1 β-glucosidases by multivalent pyrrolidine iminosugars. Bioorg. Chem..

[29] Durantel D., Alotte C., Zoulim F. (2007). Glucosidase inhibitors as antiviral agents for hepatitis B and C. Curr. Opin. Investig..

[30] Pandey S., Sree A., Dash S.S., Sethi D.P., Chowdhury L. (2013). Diversity of marine bacteria producing beta-glucosidase inhibitors. Microb. Cell Fact..

[31] Puls W., Keup U., Krause H.P., Thomas G., Hoffmeister F. (1977). Glucosidase inhibition—A new approach to the treatment of diabetes, obesity, and hyperlipoproteinaemia. Naturwissenschaften.

[32] Brogard J.M., Willemin B., Blicklé J.F., Lamalle A.M., Stahl A. (1989). Inhibiteurs des alpha-glucosidases: Une nouvelle approche thérapeutique du diabète et des hypoglycémies fonctionnelles. Rev. Med. Intern..

[33] Lankatillake C., Huynh T., Dias D.A. (2019). Understanding glycaemic control and current approaches for screening antidiabetic natural products from evidence-based medicinal plants. Plant Methods.

[34] Teng H., Chen L., Fang T., Yuan B., Lin Q. (2017). Rb2 inhibits α-glucosidase and regulates glucose me-tabolism by activating AMPK pathways in HepG2 cells. J. Funct. Foods.

[35] Kato A., Kato N., Kano E., Adachi I., Ikeda K., Yu L., Okamoto T., Banba Y., Ouchi H., Takahata H. (2005). Biological properties of D- and L-1-deoxyazasugars. J. Med. Chem..

[36] Fan J.-Q., Ishii S., Asano N., Suzuki Y. (1999). Accelerated transport and maturation of lysosomal α-galactosidase A in Fabry lymphoblasts by an enzyme inhibitor. Nat. Med..

[37] Sawkar A.R., Cheng W.C., Beutler E., Wong C.H., Balch W.E., Kelly J.W. (2002). Chemical chaperones increase the cellular activity of N370S β-glucosidase: A therapeutic strategy for Gaucher disease. Proc. Natl. Acad. Sci. USA.

[38] Gaulton A., Bellis L.J., Bento A.P., Chambers J., Davies M., Hersey A., Light Y., McGlinchey S., Michalovich D., Al-Lazikani B. (2012). ChEMBL: A large-scale bioactivity database for drug discovery. Nucleic Acids Res..

[39] Bender A. (2010). Databases: Compound bioactivities go public. Nat. Chem. Biol..

[40] Kim S., Thiessen P.A., Bolton E.E., Chen J., Fu G., Gindulyte A., Han L., He J., He S., Shoemaker B.A. (2016). PubChem Substance and Compound databases. Nucleic Acids Res..

[41] Toropov A.A., Toropova A.P., Raitano G., Benfenati E. (2019). CORAL: Building up QSAR models for the chromosome aberration test. Saudi J. Biol. Sci..

[42] Ahmadi S., Ghanbari H., Lotfi S., Azimi N. (2020). Predictive QSAR modeling for the antioxidant activity of natural compounds derivatives based on Monte Carlo method. Mol. Divers..

[43] Przybyłek M., Jeliński T., Słabuszewska J., Ziółkowska D., Mroczyńska K., Cysewski P. (2019). Application of Multivariate Adaptive Regression Splines (MARSplines) Methodology for Screening of Di-carboxylic Acid Cocrystal Using 1D and 2D Molecular Descriptors. Cryst. Growth Des..

[44] Sundar K., Rosy J.C., Balamurali S., Mary J.A., Shenbagara R. (2016). Generation of 2D-QSAR Model for Angiogenin Inhibitors: A Ligand-Based Approach for Cancer Drug Design. Trends Bioinform..

[45] Toropov A.A., Toropova A.P., Veselinović A.M., Leszczynska D., Leszczynski J. (2020). SARS-CoV Mpro inhibitory activity of aromatic disulfide compounds: QSAR model. J. Biomol. Struct. Dyn..

[46] Tran T.-S., Le M.-T., Tran T.-D., Tran T.-H., Thai K.-M. (2020). Design of Curcumin and Flavonoid Derivatives with Acetylcholinesterase and Beta-Secretase Inhibitory Activities Using in Silico Approaches. Molecules.

[47] Przybyłek M., Cysewski P. (2018). Distinguishing Cocrystals from Simple Eutectic Mixtures: Phenolic Acids as Potential Pharmaceutical Coformers. Cryst. Growth Des..

[48] Dieguez-Santana K., Pham-The H., Rivera-Borroto O.M., Puris A., Le-Thi-Thu H., Casanola-Martin G.M. (2017). A Two QSAR Way for Antidiabetic Agents Targeting Using α-Amylase and α-Glucosidase In-hibitors: Model Parameters Settings in Artificial Intelligence Techniques. Lett. Drug Des. Discov..

[49] Taxak N., Bharatam P.V. (2013). 2D QSAR study for gemfibrozil glucuronide as the mechanism-based in-hibitor of CYP2C8. Indian J. Pharm. Sci..

[50] Jafari K., Fatemi M.H., Toropova A.P., Toropov A.A. (2020). Correlation Intensity Index (CII) as a criterion of predictive potential: Applying to model thermal conductivity of metal oxide-based ethylene glycol nanofluids. Chem. Phys. Lett..

[51] Yap C.W. (2011). PaDEL-descriptor: An open source software to calculate molecular descriptors and fin-gerprints. J. Comput. Chem..

[52] Lei T., Li Y., Song Y., Li D., Sun H., Hou T.-J. (2016). ADMET evaluation in drug discovery: 15. Accurate prediction of rat oral acute toxicity using relevance vector machine and consensus modeling. J. Cheminform..

[53] Goodarzi M., Dejaegher B., Heyden Y. (2012). Vander Feature selection methods in QSAR studies. J. AOAC Int..

[54] Li Y., Dai Z., Cao D., Luo F., Chen Y., Yuan Z. (2020). Chi-MIC-share: A new feature selection algorithm for quantitative structure–activity relationship models. RSC Adv..

[55] Alsenan S.A., Al-Turaiki I.M., Hafez A.M. (2020). Feature extraction methods in quantitative struc-ture-activity relationship modeling: A comparative study. IEEE Access.

[56] Newby D., Freitas A.A., Ghafourian T. (2013). Pre-processing Feature Selection for Improved C&RT Models for Oral Absorption. J. Chem. Inf. Model..

[57] Antanasijević J., Antanasijević D., Pocajt V., Trišović N., Fodor-Csorba K. (2016). A QSPR study on the liquid crystallinity of five-ring bent-core molecules using decision trees, MARS and artificial neural networks. RSC Adv..

[58] Todeschini R., Consonni V. (2009). Molecular Descriptors for Chemoinformatics. Molecular Descriptors for Chemoinformatics.

[59] Burden F.R. (1989). Molecular identification number for substructure searches. J. Chem. Inf. Model..

[60] Burden F.R. (1997). A Chemically Intuitive Molecular Index Based on the Eigenvalues of a Modified Adjacency Matrix. Quant. Struct. Relatsh..

[61] Broto P., Moreau G., Vandycke C. (1984). Molecular structures: Perception, autocorrelation descriptor and sar studies: System of atomic contributions for the calculation of the n-octanol/water partition coef-ficients. Eur. J. Med. Chem..

[62] Broto P., Moreau G., Vandycke C. (1984). Molecular structures: Perception, autocorrelation descriptor and sar studies: Autocorrelation descriptor. Eur. J. Med. Chem..

[63] Moreau G., Broto P. (1980). Autocorrelation of molecular structures. Application to SAR studies. Nouv. J. Chim..

[64] Moreau J.L., Broto P. (1980). The autocorrelation of a topologial structure: A new molecular descriptor. Nouv. J. Chim..

[65] Huuskonen J.J., Livingstone D.J., Tetko I.V. (2000). Neural network modeling for estimation of partition coefficient based on atom-type electrotopological state indices. J. Chem. Inf. Comput. Sci..

[66] Huuskonen J.J., Villa A.E.P., Tetko I.V. (1999). Prediction of partition coefficient based on atom-type electrotopological state indices. J. Pharm. Sci..

[67] Kier L.B., Hall L.H. (1999). Molecular Structure Description: The Electrotopological State.

[68] Kier L.B., Hall L.H. (1990). An Electrotopological-State Index for Atoms in Molecules. Pharm. Res..

[69] Kier L.B., Hall L.H., Frazer J.W. (1991). An index of electrotopological state for atoms in molecules. J. Math. Chem..

[70] Votano J.R., Parham M., Hall L.H., Kier L.B., Oloff S., Tropsha A., Xie Q., Tong W. (2004). Three new consensus QSAR models for the prediction of Ames genotoxicity. Mutagenesis.

[71] Fjodorova N., Vračko M., Novič M., Roncaglioni A., Benfenati E. (2010). New public QSAR model for carcinogenicity. Chem. Cent. J..

[72] Parmeggiani C., Catarzi S., Matassini C., D’Adamio G., Morrone A., Goti A., Paoli P., Cardona F. (2015). Human Acid β-Glucosidase Inhibition by Carbohydrate Derived Iminosugars: Towards New Pharmacological Chaperones for Gaucher Disease. ChemBioChem.

[73] Yamashita T., Yasuda K., Kizu H., Kameda Y., Watson A.A., Nash R.J., Fleet G.W.J., Asano N. (2002). New polyhydroxylated pyrrolidine, piperidine, and pyrrolizidine alkaloids from *Scilla sibirica*. J. Nat. Prod..

[74] Matthews B.W. (1975). Comparison of the predicted and observed secondary structure of T4 phage lyso-zyme. BBA Protein Struct..

[75] Boughorbel S., Jarray F., El-Anbari M. (2017). Optimal classifier for imbalanced data using Matthews Correlation Coefficient metric. PLoS ONE.

[76] Paul S., Arlehamn C.S.L., Schulten V., Westernberg L., Sidney J., Peters B., Sette A. (2017). Experimental validation of the RATE tool for inferring HLA restrictions of T cell epitopes. BMC Immunol..

[77] Klingspohn W., Mathea M., Ter Laak A., Heinrich N., Baumann K. (2017). Efficiency of different measures for defining the applicability domain of classification models. J. Cheminform..

[78] Cai C., Fang J., Guo P., Wang Q., Hong H., Moslehi J., Cheng F. (2018). In Silico Pharmacoepidemiologic Evaluation of Drug-Induced Cardiovascular Complications Using Combined Classifiers. J. Chem. Inf. Model..

[79] Davis J., Goadrich M. (2006). The relationship between precision-recall and ROC curves. ACM Int. Conf. Proc. Ser..

[80] Bradley A.P. (1997). The use of the area under the ROC curve in the evaluation of machine learning algo-rithms. Pattern Recognit..

[81] Brown J. (2018). Classifiers and their Metrics Quantified. Mol. Inform..

[82] Chicco D., Jurman G. (2020). The advantages of the Matthews correlation coefficient (MCC) over F1 score and accuracy in binary classification evaluation. BMC Genom..

[83] Halimu C., Kasem A., Newaz S.H.S. (2019). Empirical comparison of area under ROC curve (AUC) and Mathew correlation coefficient (MCC) for evaluating machine learning algorithms on imbalanced datasets for binary classification. ACM Int. Conf. Proc. Ser..

[84] Lobo J.M., Jiménez-valverde A., Real R. (2008). AUC: A misleading measure of the performance of pre-dictive distribution models. Glob. Ecol. Biogeogr..

[85] Muschelli J. (2020). ROC and AUC with a Binary Predictor: A Potentially Misleading Metric. J. Classif..

[86] Kovalishyn V., Aires-de-Sousa J., Ventura C., Leitão E.R., Martins F. (2011). QSAR modeling of an-titubercular activity of diverse organic compounds. Chemom. Intell. Lab. Syst..

[87] Tropsha A., Gramatica P., Gombar V.K. (2003). The Importance of Being Earnest: Validation is the Absolute Essential for Successful Application and Interpretation of QSPR Models. QSAR Comb. Sci..

[88] Puzyn T., Mostrag-Szlichtyng A., Gajewicz-Skretna A., Skrzyński M., Worth A. (2011). Investigating the influence of data splitting on the predictive ability of QSAR/QSPR models. Struct. Chem..

[89] Pilón-Jiménez B.A., Saldívar-González F.I., Díaz-Eufracio B.I., Medina-Franco J.L. (2019). BIOFACQUIM: A Mexican Compound Database of Natural Products. Biomolecules.

[90] Nikitina A., Orlov A., Kozlovskaya L., Palyulin V., Osolodkin D.I. (2019). Enhanced taxonomy annotation of antiviral activity data from ChEMBL. Database.

[91] Haudecoeur R., Peuchmaur M., Ahmed-Belkacem A., Pawlotsky J.M., Boumendjel A. (2013). Structure-activity relationships in the development of allosteric hepatitis C virus RNA-dependent RNA polymerase inhibitors: Ten years of research. Med. Res. Rev..

[92] Bankar A., Siriwardena T.P., Rizoska B., Rydergård C., Kylefjord H., Rraklli V., Eneroth A., Pinho P., Norin S., Bylund J. (2018). 5-Fluorotroxacitabine Displays Potent Anti-Leukemic Effects and Circumvents Resistance to Ara-C. Blood.

[93] Szilágyi K., Hajdú I., Flachner B., Lőrincz Z., Balczer J., Gál P., Závodszky P., Pirli C., Balogh B., Mándity I.M. (2019). Design and Selection of Novel C1s Inhibitors by In Silico and In Vitro Approaches. Molecules.

[94] Zhong M., Munzer J.S., Basak A., Benjannet S., Mowla S.J., Decroly E., Chrétien M., Seidah N.G. (1999). The Prosegments of Furin and PC7 as Potent Inhibitors of Proprotein Convertases. J. Biol. Chem..

[95] Poumale H.M.P., Hamm R., Zang Y., Shiono Y., Kuete V. (2013). Coumarins and Related Compounds from the Medicinal Plants of Africa. Medicinal Plant Research in Africa: Pharmacology and Chemistry.

[96] Statsoft (2012). Statistica.

