# Supplementary material for: Application 2D Descriptors and Artificial Neural Networks for Beta-Glucosidase Inhibitors Screening
Source: Molecules. 2020 Dec 15;25(24):5942. doi: 10.3390/molecules25245942 (PMC7765417; doi:10.3390/molecules25245942)
Supplement: Supplementary file 1 [file molecules-25-05942-s001.zip › molecules-1025529-SI/molecules-1025529-SI.docx]

**Table S1** The dataset used for model development and validation obtained from ChEMBL database (<https://www.ebi.ac.uk/chembl/>). In case of more than IC50 values the mean was taken into account. The classification threshold was 50µM. Class 0 denotes weakly active or non-active compounds, while Class 1, active ones.

| **No.** | **Compound ID** | **SMILES** | **IC50 [µM]** | **ChEMBL Assay ID** | **Class** |
| --- | --- | --- | --- | --- | --- |
| 1 | CHEMBL3736317 | Cl.CCCCCCN[C@@H]1C=C[C@H](O)[C@H](O)[C@H]1O | >1000 | CHEMBL3737684 | 0 |
| 2 | CHEMBL82895 | OC1CNC(CNCn2ccnc2)C1O | 420.00 | CHEMBL650045 | 0 |
| 3 | CHEMBL2011623 | CCCCN1CCC[C@H](O)[C@@H]1CO | >500 | CHEMBL2015864 | 0 |
| 4 | CHEMBL84272 | OC1CNC(CNCC2CCN(Cc3ccccc3)CC2)C1O | 430.00 | CHEMBL650045 | 0 |
| 5 | CHEMBL501385 | OC[C@@H]1CNC[C@H](O)[C@H]1O | 84.57 | CHEMBL993569, CHEMBL1820880, CHEMBL1820877 | 0 |
| 6 | CHEMBL83278 | OC(O)CC1NCC(O)C1O | 70.00 | CHEMBL650045 | 0 |
| 7 | CHEMBL3739825 | COc1ccc(\C=C\c2cc(OC)cc(OC)c2O[C@H]3O[C@H](CO)[C@H](O)[C@H](O)[C@H]3O)cc1 | 350.00 | CHEMBL3742524 | 0 |
| 8 | CHEMBL2011637 | CCCCN1CC[C@H](O)[C@@H](O)[C@@H]1CO | >1000 | CHEMBL2015864 | 0 |
| 9 | CHEMBL3736333 | Cl.CC(C)CN[C@@H]1C=C[C@H](O)[C@H](O)[C@H]1O | >1000 | CHEMBL3737684 | 0 |
| 10 | CHEMBL4085739 | CCCCC[C@H]1NC[C@@H](O)[C@@H](O)[C@H]1CO | 100.00 | CHEMBL4007594 | 0 |
| 11 | CHEMBL186150 | CCCCCCN1CC(O)C(O)C(O)C1CO | 350.00 | CHEMBL832785 | 0 |
| 12 | CHEMBL2011621 | OC[C@H]1[C@H](O)CCCN1CCCCCOCC23CC4CC(CC(C4)C2)C3 | >1000 | CHEMBL2015864 | 0 |
| 13 | CHEMBL87120 | NC1C(O)C(O)C(O)C1O | 131.83 | CHEMBL647637 | 0 |
| 14 | CHEMBL2011640 | OC[C@H]1[C@@H](O)[C@@H](O)CCN1CCCCCOCC23CC4CC(CC(C4)C2)C3 | 75.00 | CHEMBL2015864 | 0 |
| 15 | CHEMBL84104 | OC1CNC(CNCc2ccccc2)C1O | 370.00 | CHEMBL650045 | 0 |
| 16 | CHEMBL2011607 | OC[C@@H]1NCC=C[C@H]1O | >500 | CHEMBL2015864 | 0 |
| 17 | CHEMBL362754 | CCCN1CC(O)C(O)C(O)C1CO | 900.00 | CHEMBL832785 | 0 |
| 18 | CHEMBL2011624 | OC[C@H]1[C@@H](O)CCCN1CCCCCOCC23CC4CC(CC(C4)C2)C3 | 500.00 | CHEMBL2015864 | 0 |
| 19 | CHEMBL2011614 | CCCCN1CCC[C@H](O)[C@H]1CO | >1000 | CHEMBL2015864 | 0 |
| 20 | CHEMBL3735749 | Cl.O[C@H]1C=C[C@@H](NCc2ccccc2)[C@H](O)[C@H]1O | >1000 | CHEMBL3737684 | 0 |
| 21 | CHEMBL3754250 | CO[C@@H]1[C@@H](O)[C@@H](O)[C@@H](CO)N=C1NCc2ccc(C)cc2 | >5000 | CHEMBL3815659 | 0 |
| 22 | CHEMBL11216 | Clc1c(Cl)c(Cl)c2C(=O)N(CCCc3ccccc3)C(=O)c2c1Cl | >500 | CHEMBL647638 | 0 |
| 23 | CHEMBL2011613 | OC[C@H]1NCCC[C@@H]1O | >1000 | CHEMBL2015864 | 0 |
| 24 | CHEMBL2011608 | CCCCN1CC=C[C@@H](O)[C@@H]1CO | >500 | CHEMBL2015864 | 0 |
| 25 | CHEMBL577370 | OC[C@H]1O[C@@H]2[C@@H](NC(=O)CCCCOCC34CC5CC(CC(C5)C3)C4)[C@@H]2[C@@H](O)[C@@H]1O | >1000 | CHEMBL1040323 | 0 |
| 26 | CHEMBL573995 | OC[C@H]1O[C@@H]2[C@@H](NCCCCCOCC34CC5CC(CC(C5)C3)C4)[C@@H]2[C@@H](O)[C@@H]1O | >1000 | CHEMBL1040323 | 0 |
| 27 | CHEMBL4096086 | OC[C@@]1(NC[C@@H](O)[C@H]1O)C(=O)O | 445.00 | CHEMBL4044715 | 0 |
| 28 | CHEMBL3736427 | Cl.CC(C)CCN[C@@H]1C=C[C@H](O)[C@H](O)[C@H]1O | >1000 | CHEMBL3737684 | 0 |
| 29 | CHEMBL3735899 | Cl.CCCCCCCCN[C@@H]1C=C(C)[C@H](O)[C@H](O)[C@H]1O | >1000 | CHEMBL3737684 | 0 |
| 30 | CHEMBL184028 | CCCCCCCCCC1NC(CO)C(O)C(O)C1O | 150.00 | CHEMBL832785 | 0 |
| 31 | CHEMBL88408 | OCC1(O)C(O)C(O)C2N=C(Nc3ccccc3)OC12 | >300 | CHEMBL647636 | 0 |
| 32 | CHEMBL468062 | OC[C@H]1N[C@@H]([C@@H](O)[C@@H]1O)c2ccccc2 | 197.00 | CHEMBL1014583 | 0 |
| 33 | CHEMBL2011638 | OC[C@H]1[C@H](O)[C@@H](O)CCN1CCCCCOCC23CC4CC(CC(C4)C2)C3 | 20.00 | CHEMBL2015864 | 1 |
| 34 | CHEMBL470659 | OCCCC(=O)CCCCCCCC[C@@H](O)[C@H]1N[C@H](CO)[C@@H](O)[C@@H]1O | 0.01 | CHEMBL1007617 | 1 |
| 35 | CHEMBL3354620 | OC[C@@H]1[C@@H](O)[C@H](O)[C@@H](O)CN1CC(F)(F)CCCOCc2ccc(cc2)c3ccccc3 | 0.01 | CHEMBL3384691 | 1 |
| 36 | CHEMBL115510 | CCCCCCNC1CC(C)C(O)C(O)C1O | 0.73 | CHEMBL647640 | 1 |
| 37 | CHEMBL2011642 | O[C@@H]1CN(CCCCCOCC23CC4CC(CC(C4)C2)C3)C[C@H](O)[C@H]1O | 0.80 | CHEMBL2015864 | 1 |
| 38 | CHEMBL324840 | CC1CC(NCCCCc2ccccc2)C(O)C(O)C1O | 1.40 | CHEMBL647640 | 1 |
| 39 | CHEMBL186068 | CCCCCCCCN1CC(O)C(O)C(O)C1CO | 34.00 | CHEMBL832785 | 1 |
| 40 | CHEMBL3354028 | OC[C@H]1[C@@H](O)[C@H](O)[C@@H](O)CN1CCCC\C=C/C23CC4CC(CC(C4)C2)C3 | 0.01 | CHEMBL3384691 | 1 |
| 41 | CHEMBL3354036 | OC[C@H]1[C@@H](O)[C@H](O)[C@@H](O)CN1CCCCCOCc2cc3ccccc3c4ccccc24 | 0.20 | CHEMBL3384691 | 1 |
| 42 | CHEMBL3354014 | OC[C@@H]1[C@@H](O)[C@H](O)[C@@H](O)CN1CCCCCOCC2CCCCC2 | 4.00•10^-3^ | CHEMBL3384691 | 1 |
| 43 | CHEMBL3815031 | CCCCC(CC)CNC1=N[C@H](CO)[C@H](O)[C@H](O)[C@H]1O | 2.50 | CHEMBL3815659 | 1 |
| 44 | CHEMBL3354021 | OC[C@@H]1[C@@H](O)[C@H](O)[C@@H](O)CN1CCCCCOCc2ccccc2 | 1.00•10^-3^ | CHEMBL3384691 | 1 |
| 45 | CHEMBL3354038 | OC[C@H]1[C@@H](O)[C@H](O)[C@@H](O)CN1CCCCCOCc2ccc(cc2)c3ccccc3 | 3.00•10^-3^ | CHEMBL3384691 | 1 |
| 46 | CHEMBL2011629 | CCCCN1CC[C@@H](O)[C@@H](O)[C@@H]1CO | 30.00 | CHEMBL2015864 | 1 |
| 47 | CHEMBL513394 | OC[C@H]1N[C@H]([C@H](O)CCCCCCCCC(=O)CCCO[C@@H]2O[C@H](CO)[C@@H](O)[C@H](O)[C@H]2O)[C@@H](O)[C@@H]1O | 0.01 | CHEMBL1007617 | 1 |
| 48 | CHEMBL326599 | CCCCCCCCCCCCN[C@H]1CC(C)[C@@H](O)C(O)[C@@H]1O | 3.60 | CHEMBL647640 | 1 |
| 49 | CHEMBL3354029 | OC[C@H]1[C@@H](O)[C@H](O)[C@@H](O)CN1CCCCCCOCC23CC4CC(CC(C4)C2)C3 | 2.50•10^-3^ | CHEMBL3384691 | 1 |
| 50 | CHEMBL2011631 | CCCCN1CC[C@@H](O)[C@H](O)[C@@H]1CO | 20.00 | CHEMBL2015864 | 1 |
| 51 | CHEMBL3354626 | OC[C@@H]1[C@@H](O)[C@H](O)[C@@H](O)CN1CCCCCOCc2ccc(cc2C(F)(F)F)c3ccccc3 | 1.20•10^-4^ | CHEMBL3384691 | 1 |
| 52 | CHEMBL86865 | OC1C(O)C2OC(=NC2C1O)Nc3ccccc3 | 10.85 | CHEMBL647636 | 1 |
| 53 | CHEMBL314757 | CCCCCCCCNC1CC(C)C(O)C(O)C1O | 1.50 | CHEMBL647640 | 1 |
| 54 | CHEMBL3354022 | OC[C@@H]1[C@@H](O)[C@H](O)[C@@H](O)CN1CCCCCOCc2ccc3ccccc3c2 | 1.00•10^-3^ | CHEMBL3384691 | 1 |
| 55 | CHEMBL3354024 | OC[C@@H]1[C@@H](O)[C@H](O)[C@@H](O)CN1CCCCCOCc2ccc3ccc4cccc5ccc2c3c45 | 3.00•10^-3^ | CHEMBL3384691 | 1 |
| 56 | CHEMBL469844 | OC[C@H]1N[C@H](CO)[C@@H](O)[C@H]1O | 23.90 | CHEMBL1007695, CHEMBL1168037 | 1 |
| 57 | CHEMBL3354035 | OC[C@H]1[C@@H](O)[C@H](O)[C@@H](O)CN1CCCCCOCc2ccc3ccccc3c2 | 1.00•10^-3^ | CHEMBL3384691 | 1 |
| 58 | CHEMBL3359675 | CCCCCCCCCCCC\N=C\1/N[C@H](CO)[C@H](O)[C@H](O)[C@H]1O | 0.40 | CHEMBL3815659 | 1 |
| 59 | CHEMBL3354049 | OC[C@@H]1[C@@H](O)[C@H](O)[C@@H](O)CN1CCCCCOCc2ccc(cc2)c3cccc(F)c3 | 1.00•10^-3^ | CHEMBL3384691 | 1 |
| 60 | CHEMBL3354630 | OC[C@H]1[C@@H](O)[C@H](O)[C@@H](O)CN1CCCCCOCc2ccccc2c3ccccc3 | 3.00•10^-3^ | CHEMBL3384691 | 1 |
| 61 | CHEMBL3354634 | OC[C@H]1[C@@H](O)[C@H](O)[C@@H](O)CN1CCCCCOCc2ccc(cc2C(F)(F)F)c3ccccc3 | 1.00•10^-4^ | CHEMBL3384691 | 1 |
| 62 | CHEMBL3354018 | CC(C)CCC[C@@H](C)[C@H]1CC[C@H]2[C@@H]3CC=C4C[C@H](CC[C@]4(C)[C@H]3CC[C@]12C)OCCCCCN5C[C@H](O)[C@@H](O)[C@H](O)[C@H]5CO | 0.01 | CHEMBL3384691 | 1 |
| 63 | CHEMBL3972306 | CC1=CC(=O)Oc2cc(OCCCCn3cc(CCCCN4C[C@H](O)[C@@H](O)[C@H](O)[C@H]4CO)nn3)ccc12 | 9.30 | CHEMBL3874807 | 1 |
| 64 | CHEMBL3354055 | OC[C@@H]1[C@@H](O)[C@H](O)[C@@H](O)CN1CCCCCOCc2ccc(cc2)c3ccc4OCCOc4c3 | 0.03 | CHEMBL3384691 | 1 |
| 65 | CHEMBL9020 | O=C1N(C(=O)c2ccccc12)c3ccccc3 | >500 | CHEMBL647638 | 0 |
| 66 | CHEMBL2011601 | OC[C@H]1NCC=C[C@@H]1O | >1000 | CHEMBL2015864 | 0 |
| 67 | CHEMBL356023 | CCCCN1CC(C)C(O)C(O)C1 | 80.00 | CHEMBL820821 | 0 |
| 68 | CHEMBL3734817 | Cl.O[C@H]1C=C[C@@H](NC2CCCCC2)[C@H](O)[C@H]1O | >1000 | CHEMBL3737684 | 0 |
| 69 | CHEMBL2011602 | CCCCN1CC=C[C@H](O)[C@H]1CO | 250.00 | CHEMBL2015864 | 0 |
| 70 | CHEMBL445624 | CC(=O)N(CC1NCC(O)C1O)Cc2ccccc2 | 430.00 | CHEMBL650045 | 0 |
| 71 | CHEMBL511823 | CC(O)[C@@H](O)[C@H]1N[C@H](CO)[C@@H](O)[C@@H]1O | 120.00 | CHEMBL1007617 | 0 |
| 72 | CHEMBL3980365 | CN(C)c1cccc2c(cccc12)S(=O)(=O)NCCOCCn3cc(CCCCN4C[C@H](O)[C@@H](O)[C@H](O)[C@H]4CO)nn3 | 97.80 | CHEMBL3874807 | 0 |
| 73 | CHEMBL313295 | OC1CNC(CNCc2occc2)C1O | 140.00 | CHEMBL650045 | 0 |
| 74 | CHEMBL2011610 | OC[C@@H]1NCC=C[C@@H]1O | >1000 | CHEMBL2015864 | 0 |
| 75 | CHEMBL3736274 | Cl.CCCCCCCCCCN[C@@H]1C=C[C@H](O)[C@H](O)[C@H]1O | >1000 | CHEMBL3737684 | 0 |
| 76 | CHEMBL2204848 | O[C@H]1[C@H](O)[C@H](C=C[C@@H]1N2CCc3ccccc23)N4CCc5ccccc45 | 67.70 | CHEMBL2216697 | 0 |
| 77 | CHEMBL2207396 | O[C@@H]1CNC[C@@H]1O | 600.00 | CHEMBL650045 | 0 |
| 78 | CHEMBL2011630 | OC[C@H]1[C@H](O)[C@H](O)CCN1CCCCCOCC23CC4CC(CC(C4)C2)C3 | 100.00 | CHEMBL2015864 | 0 |
| 79 | CHEMBL2011633 | CCCCN1CC[C@H](O)[C@H](O)[C@H]1CO | 1000.00 | CHEMBL2015864 | 0 |
| 80 | CHEMBL275285 | Clc1c(Cl)c(Cl)c2C(=O)N(CCCCc3ccccc3)C(=O)c2c1Cl | >500 | CHEMBL647638 | 0 |
| 81 | CHEMBL3970812 | CCCCCCCCCCNC(=S)N1[C@@H](CO)[C@H](O)[C@@H](O)[C@@H]1CO | 65.00 | CHEMBL3869409 | 0 |
| 82 | CHEMBL369297 | OC[C@@H]1NC[C@@H](O)[C@H](O)[C@H]1O | 980.00 | CHEMBL1820877 | 0 |
| 83 | CHEMBL2011605 | CCCCN1CC=C[C@@H](O)[C@H]1CO | >1000 | CHEMBL2015864 | 0 |
| 84 | CHEMBL2011609 | OC[C@H]1[C@H](O)C=CCN1CCCCCOCC23CC4CC(CC(C4)C2)C3 | >500 | CHEMBL2015864 | 0 |
| 85 | CHEMBL463872 | COc1ccc(cc1)[C@@H]2N[C@@H](CO)[C@H](O)[C@H]2O | >200 | CHEMBL1014583 | 0 |
| 86 | CHEMBL86772 | OCC12OC(=NC1C(O)C(O)C2O)Nc3ccccc3 | 201.63 | CHEMBL647636 | 0 |
| 87 | CHEMBL2011617 | CCCCN1CCC[C@@H](O)[C@H]1CO | >1000 | CHEMBL2015864 | 0 |
| 88 | CHEMBL3735277 | Cl.CCCCN[C@@H]1C=C[C@H](O)[C@H](O)[C@H]1O | >1000 | CHEMBL3737684 | 0 |
| 89 | CHEMBL152232 | CCCCN1CC(O)C(O)C(O)C1 | 530.00 | CHEMBL820821 | 0 |
| 90 | CHEMBL421340 | CC(NCC1NCC(O)C1O)C(O)(c2ccccc2)c3ccccc3 | 550.00 | CHEMBL650045 | 0 |
| 91 | CHEMBL2206827 | C[C@H](O)NC[C@H]1NC[C@H](O)[C@@H]1O | 290.00 | CHEMBL650045 | 0 |
| 92 | CHEMBL1561 | OCCN1C[C@H](O)[C@@H](O)[C@H](O)[C@H]1CO | 231.00 | CHEMBL993569, CHEMBL3874807 | 0 |
| 93 | CHEMBL11516 | Clc1c(Cl)c(Cl)c2C(=O)N(Cc3ccccc3)C(=O)c2c1Cl | >500 | CHEMBL647638 | 0 |
| 94 | CHEMBL314403 | OCC1(O)C(O)C(O)C2(CO)OCC(=NC12)Nc3ccccc3 | 211.00 | CHEMBL647636 | 0 |
| 95 | CHEMBL3742008 | COc1ccc(\C=C\c2cc(OC)cc(OC)c2O[C@H]3O[C@H](CO)[C@@H](O)[C@H](O)[C@H]3O)cc1 | 208.00 | CHEMBL3742524 | 0 |
| 96 | CHEMBL507492 | COc1ccc(cc1)[C@H]2[C@H](O)[C@@H](O)[C@H](CO)N2C | >200 | CHEMBL1014583 | 0 |
| 97 | CHEMBL313375 | OCC1C(O)C(O)C2OC(=NC12)Nc3ccccc3 | >300 | CHEMBL647636 | 0 |
| 98 | CHEMBL1818439 | OC[C@@H]1NCC[C@H](O)[C@@H]1O | >1000 | CHEMBL2015864 | 0 |
| 99 | CHEMBL574865 | N[C@@H]1[C@H]2O[C@H](CO)[C@@H](O)[C@H](O)[C@@H]12 | >1000 | CHEMBL1040323 | 0 |
| 100 | CHEMBL464053 | COc1ccc(cc1OC)[C@@H]2[C@@H](O)[C@H](O)[C@@H](CO)N2C | >200 | CHEMBL1014583 | 0 |
| 101 | CHEMBL1933096 | Cl.OC[C@@H]1CNC[C@@H](O)[C@@H](O)[C@@H]1O | 157.00 | CHEMBL1937704 | 0 |
| 102 | CHEMBL3740658 | COc1cc(OC)c(O[C@H]2O[C@H](CO)[C@@H](O)[C@H](O)[C@H]2O)c(\C=C\c3ccc(OC)c(OC)c3)c1 | 192.00 | CHEMBL3742524 | 0 |
| 103 | CHEMBL2011620 | CCCCN1CCC[C@@H](O)[C@@H]1CO | >500 | CHEMBL2015864 | 0 |
| 104 | CHEMBL3736474 | Cl.OCCCN[C@@H]1C=C[C@H](O)[C@H](O)[C@H]1O | >1000 | CHEMBL3737684 | 0 |
| 105 | CHEMBL448744 | COc1ccc(cc1)[C@H]2N[C@H](CO)[C@@H](O)[C@@H]2O | 122.00 | CHEMBL1014583 | 0 |
| 106 | CHEMBL3740283 | COc1cc(OC)cc(\C=C\c2cc(OC)cc(OC)c2O[C@H]3O[C@H](CO)[C@@H](O)[C@H](O)[C@H]3O)c1 | 272.00 | CHEMBL3742524 | 0 |
| 107 | CHEMBL3359684 | OC[C@H]1NC(=O)[C@H](O)[C@@H](O)[C@H]1O | >1000 | CHEMBL3815659 | 0 |
| 108 | CHEMBL584580 | CCCC(=O)N[C@@H]1[C@H]2O[C@H](CO)[C@@H](O)[C@H](O)[C@@H]12 | >1000 | CHEMBL1040323 | 0 |
| 109 | CHEMBL1236649 | OC[C@H]1C[C@H](N[C@H]2C=C(CO)[C@@H](O)[C@H](O)[C@H]2O)[C@H](O)[C@@H](O)[C@@H]1O | >1000 | CHEMBL3073479 | 0 |
| 110 | CHEMBL465105 | COc1ccc([C@@H]2[C@@H](O)[C@H](O)[C@@H](CO)N2C)c(OC)c1 | >200 | CHEMBL1014583 | 0 |
| 111 | CHEMBL275509 | Clc1c(Cl)c(Cl)c2C(=O)N(CCCCCCc3ccccc3)C(=O)c2c1Cl | >500 | CHEMBL647638 | 0 |
| 112 | CHEMBL3133385 | Cl.CCCCCCN1C[C@H](O)[C@@H](O)[C@H](O)[C@H]1CO | 59.00 | CHEMBL3134886 | 0 |
| 113 | CHEMBL583176 | CCCCN[C@@H]1[C@H]2O[C@H](CO)[C@@H](O)[C@H](O)[C@@H]12 | >1000 | CHEMBL1040323 | 0 |
| 114 | CHEMBL503122 | COc1ccc(cc1O)[C@@H]2[C@@H](O)[C@H](O)[C@@H](CO)N2C | >200 | CHEMBL1014583 | 0 |
| 115 | CHEMBL2011618 | OC[C@@H]1[C@H](O)CCCN1CCCCCOCC23CC4CC(CC(C4)C2)C3 | >1000 | CHEMBL2015864 | 0 |
| 116 | CHEMBL502396 | COc1ccc([C@H]2N[C@H](CO)[C@@H](O)[C@@H]2O)c(OC)c1 | 96.00 | CHEMBL1014583 | 0 |
| 117 | CHEMBL1818435 | OC[C@@H]1NCC[C@H](O)[C@H]1O | >1000 | CHEMBL2015864 | 0 |
| 118 | CHEMBL3359683 | OC[C@H]1NC(=N)[C@H](O)[C@@H](O)[C@H]1O | >1000 | CHEMBL3815659 | 0 |
| 119 | CHEMBL11477 | Nc1ccc2C(=O)N(C(=O)c2c1)c3ccccc3 | >500 | CHEMBL647638 | 0 |
| 120 | CHEMBL465034 | COc1ccc(OC)c(c1)[C@@H]2[C@@H](O)[C@H](O)[C@@H](CO)N2C | >200 | CHEMBL1014583 | 0 |
| 121 | CHEMBL3133390 | Cl.OCCNC(=N)N1C[C@H](O)[C@@H](O)[C@H](O)[C@H]1CO | 457.00 | CHEMBL3134886 | 0 |
| 122 | CHEMBL11322 | O=C1N(CCCc2ccccc2)C(=O)c3ccccc13 | >500 | CHEMBL647638 | 0 |
| 123 | CHEMBL108084 | OC[C@H]1NCC[C@@H](O)[C@@H]1O | >1000 | CHEMBL2015864 | 0 |
| 124 | CHEMBL505237 | OC[C@H]1NCC[C@H](O)[C@H]1O | >1000 | CHEMBL2015864 | 0 |
| 125 | CHEMBL2011622 | OC[C@@H]1NCCC[C@@H]1O | >1000 | CHEMBL2015864 | 0 |
| 126 | CHEMBL80254 | OC[C@H]1NC[C@@H](O)[C@@H]1O | 120.00 | CHEMBL922471 | 0 |
| 127 | CHEMBL2011639 | CCCCN1CC[C@H](O)[C@H](O)[C@@H]1CO | >1000 | CHEMBL2015864 | 0 |
| 128 | CHEMBL259905 | OC[C@@H]1N[C@H](CO)[C@@H](O)[C@@H]1O | 320.00 | CHEMBL1007695 | 0 |
| 129 | CHEMBL3736146 | Cl.CCCCCN[C@@H]1C=C[C@H](O)[C@H](O)[C@H]1O | >1000 | CHEMBL3737684 | 0 |
| 130 | CHEMBL11566 | Oc1cccc2C(=O)N(C(=O)c12)c3ccccc3 | >500 | CHEMBL647638 | 0 |
| 131 | CHEMBL3891002 | CCCCCCCCCCCCNC(=S)N1[C@@H](CO)[C@H](O)[C@@H](O)[C@@H]1CO | 73.00 | CHEMBL3869409 | 0 |
| 132 | CHEMBL1566 | C[C@H]1O[C@H](O[C@H]2[C@H](O)[C@@H](O)[C@@H](O[C@H]3[C@H](O)[C@@H](O)[C@H](O)O[C@@H]3CO)O[C@@H]2CO)[C@H](O)[C@@H](O)[C@@H]1N[C@H]4C=C(CO)[C@@H](O)[C@H](O)[C@H]4O | 103.12 | CHEMBL2216697 | 0 |
| 133 | CHEMBL276643 | Clc1c(Cl)c(Cl)c2C(=O)N(CCCCCc3ccccc3)C(=O)c2c1Cl | >500 | CHEMBL647638 | 0 |
| 134 | CHEMBL468063 | COc1ccc(cc1O)[C@H]2N[C@H](CO)[C@@H](O)[C@@H]2O | >200 | CHEMBL1014583 | 0 |
| 135 | CHEMBL456583 | OC[C@H]1NCC[C@H](O)[C@@H]1O | 416.00 | CHEMBL2015864, CHEMBL1008472, CHEMBL1820877, CHEMBL1007669, CHEMBL647648 | 0 |
| 136 | CHEMBL312653 | OC[C@H]1N[C@H](CO)[C@@H](O)[C@@H]1O | 64.67 | CHEMBL922471, CHEMBL3869402, CHEMBL647648 | 0 |
| 137 | CHEMBL273605 | [O-][N+](=O)c1ccc2C(=O)N(C(=O)c2c1)c3ccccc3 | >500 | CHEMBL647638 | 0 |
| 138 | CHEMBL3742045 | COc1cc(OC)c(O[C@H]2O[C@H](CO)[C@H](O)[C@H](O)[C@H]2O)c(\C=C\c3ccc(OC)c(OC)c3)c1 | 170.00 | CHEMBL3742524 | 0 |
| 139 | CHEMBL150938 | OC1CNCC(=C)C1O | >1000 | CHEMBL820821 | 0 |
| 140 | CHEMBL1818321 | OC[C@H]1CNC[C@H](O)[C@@H]1O | 278.00 | CHEMBL1820880, CHEMBL1820877 | 0 |
| 141 | CHEMBL3753015 | CO[C@@H]1[C@H](O)[C@@H](O)C(=N[C@@H]1CO)NCc2ccc(C)cc2 | >5000 | CHEMBL3815659 | 0 |
| 142 | CHEMBL466791 | OC[C@H]1N[C@@H]([C@@H](O)[C@@H]1O)c2ccc(O)cc2 | >200 | CHEMBL1014583 | 0 |
| 143 | CHEMBL3133386 | CCCCNC(=O)N1C[C@H](O)[C@@H](O)[C@H](O)[C@H]1CO | >1000 | CHEMBL3134886 | 0 |
| 144 | CHEMBL3943978 | CCCC\N=C/1\SC[C@@H]2[C@@H](O)[C@H](O)[C@@H](CO)N12 | 532.00 | CHEMBL3869409 | 0 |
| 145 | CHEMBL11766 | Clc1c(Cl)c(Cl)c2C(=O)N(CCc3ccccc3)C(=O)c2c1Cl | >500 | CHEMBL647638 | 0 |
| 146 | CHEMBL3752112 | Cc1ccc(CNC2=N[C@H](CO)[C@H](O)[C@H](O)C2)cc1 | >5000 | CHEMBL3815659 | 0 |
| 147 | CHEMBL2115197 | CC[C@H]1NC[C@H](O)[C@@H]1O | 175.00 | CHEMBL650045, CHEMBL647648 | 0 |
| 148 | CHEMBL2207397 | OCC(O)[C@H]1NC[C@H](O)[C@@H]1O | 120.00 | CHEMBL650045 | 0 |
| 149 | CHEMBL3736331 | Cl.CCC(CC)N[C@@H]1C=C[C@H](O)[C@H](O)[C@H]1O | >1000 | CHEMBL3737684 | 0 |
| 150 | CHEMBL11452 | O=C1N(CCc2ccccc2)C(=O)c3ccccc13 | >500 | CHEMBL647638 | 0 |
| 151 | CHEMBL2011616 | OC[C@H]1NCCC[C@H]1O | >1000 | CHEMBL2015864 | 0 |
| 152 | CHEMBL2011619 | OC[C@@H]1NCCC[C@H]1O | >1000 | CHEMBL2015864 | 0 |
| 153 | CHEMBL463871 | COc1ccc(cc1)[C@@H]2[C@@H](O)[C@H](O)[C@@H](CO)N2C | >200 | CHEMBL1014583 | 0 |
| 154 | CHEMBL2011606 | OC[C@@H]1[C@H](O)C=CCN1CCCCCOCC23CC4CC(CC(C4)C2)C3 | 130.00 | CHEMBL2015864 | 0 |
| 155 | CHEMBL416887 | O=C1N(Cc2ccccc2)C(=O)c3ccccc13 | >500 | CHEMBL647638 | 0 |
| 156 | CHEMBL2011615 | OC[C@@H]1[C@@H](O)CCCN1CCCCCOCC23CC4CC(CC(C4)C2)C3 | 100.00 | CHEMBL2015864 | 0 |
| 157 | CHEMBL2206826 | C[C@@H](O)NC[C@H]1NC[C@H](O)[C@@H]1O | 410.00 | CHEMBL650045 | 0 |
| 158 | CHEMBL3735475 | Cl.O[C@H]1C=C[C@@H](NCCc2ccccc2)[C@H](O)[C@H]1O | >1000 | CHEMBL3737684 | 0 |
| 159 | CHEMBL3359679 | OC[C@H]1N\C(=N/C2CCCCC2)\[C@H](O)[C@@H](O)[C@H]1O | >5000 | CHEMBL3815659 | 0 |
| 160 | CHEMBL187158 | CCCCCCCCCN1CC(O)C(O)C(O)C1CO | 150.00 | CHEMBL832785 | 0 |
| 161 | CHEMBL3133389 | Cl.OCCOCCNC(=N)N1C[C@H](O)[C@@H](O)[C@H](O)[C@H]1CO | >1000 | CHEMBL3134886 | 0 |
| 162 | CHEMBL1163254 | CC[C@H]1CC[C@@H](O)[C@@H](CO[C@@H]2O[C@H](CO)[C@@H](O)[C@H](O)[C@H]2O)N1 | 321.00 | CHEMBL1168037 | 0 |
| 163 | CHEMBL1818437 | OC[C@@H]1NCC[C@@H](O)[C@@H]1O | >1000 | CHEMBL2015864 | 0 |
| 164 | CHEMBL3736234 | Cl.CCC(CC)CN[C@@H]1C=C[C@H](O)[C@H](O)[C@H]1O | >1000 | CHEMBL3737684 | 0 |
| 165 | CHEMBL2011625 | CCCCN1CC[C@@H](O)[C@H](O)[C@H]1CO | >1000 | CHEMBL2015864 | 0 |
| 166 | CHEMBL3735278 | Cl.O[C@H]1C=C[C@@H](NCC2CCCCC2)[C@H](O)[C@H]1O | >1000 | CHEMBL3737684 | 0 |
| 167 | CHEMBL2409320 | OC[C@H](O)[C@@H]1NC[C@@H]1O | 347.00 | CHEMBL2412536 | 0 |
| 168 | CHEMBL276030 | Oc1ccc2C(=O)N(C(=O)c2c1)c3ccccc3 | >500 | CHEMBL647638 | 0 |
| 169 | CHEMBL1818434 | OC[C@@H]1CNC[C@H](O)[C@@H]1O | 983.00 | CHEMBL1820877 | 0 |
| 170 | CHEMBL2011627 | CCCCN1CC[C@@H](O)[C@@H](O)[C@H]1CO | >1000 | CHEMBL2015864 | 0 |
| 171 | CHEMBL3814915 | Cc1ccc(CNC2=N[C@H](CO)[C@@H](O)[C@@H]2O)cc1 | 6200.00 | CHEMBL3815659 | 0 |
| 172 | CHEMBL11614 | [O-][N+](=O)c1cccc2C(=O)N(C(=O)c12)c3ccccc3 | >500 | CHEMBL647638 | 0 |
| 173 | CHEMBL3133392 | Cl.OC[C@@H]1[C@@H](O)[C@H](O)[C@@H](O)CN1C(=N)NCCCc2ccccc2 | 67.00 | CHEMBL3134886 | 0 |
| 174 | CHEMBL269410 | Nc1cccc2C(=O)N(C(=O)c12)c3ccccc3 | >500 | CHEMBL647638 | 0 |
| 175 | CHEMBL445980 | COc1ccc(OC)c(c1)[C@H]2N[C@H](CO)[C@@H](O)[C@@H]2O | 169.00 | CHEMBL1014583 | 0 |
| 176 | CHEMBL1818438 | OC[C@H]1NCC[C@@H](O)[C@H]1O | >1000 | CHEMBL2015864 | 0 |
| 177 | CHEMBL2011604 | OC[C@H]1NCC=C[C@H]1O | >1000 | CHEMBL2015864 | 0 |
| 178 | CHEMBL511534 | CCCCN1[C@H](CO)[C@@H](O)[C@H](O)[C@H]1c2ccc(OC)c(O)c2 | >200 | CHEMBL1014583 | 0 |
| 179 | CHEMBL84940 | OC1CNC(CNC2CCCC2)C1O | 500.00 | CHEMBL650045 | 0 |
| 180 | CHEMBL185536 | CC(=O)NC1CC(CO)C(O)C(O)C1O | 14.00•10^6^ | CHEMBL831683 | 0 |
| 181 | CHEMBL1628264 | N[C@H]1C[C@H](CO)[C@@H](O)[C@H](O)[C@H]1O | 13.00•10^7^ | CHEMBL831683 | 0 |
| 182 | CHEMBL3349573 | OC[C@@H](OS(=O)(=O)[O-])[C@@H](O)C[S+]1C[C@@H](O)[C@H](O)[C@@H]1CO | 3400.00 | CHEMBL650041 | 0 |
| 183 | CHEMBL357108 | CC1CNCC(O)C1O | 500.00 | CHEMBL820821 | 0 |
| 184 | CHEMBL88413 | NC1C(O)C(O)C(O)C1(O)CO | 164.65 | CHEMBL647637 | 0 |
| 185 | CHEMBL1922579 | Cl.CCCCCCCCN[C@@H]1C=C(CO)[C@H](O)[C@H](O)[C@H]1O | 504.05 | CHEMBL3737684, CHEMBL1924471 | 0 |
| 186 | CHEMBL368121 | OC[C@H]1NC[C@@H](O)[C@H](O)[C@H]1O | 66.00 | CHEMBL826964 | 0 |
| 187 | CHEMBL454551 | COc1ccc(cc1O)[C@@H]2N[C@@H](CO)[C@H](O)[C@H]2O | >200 | CHEMBL1014583 | 0 |
| 188 | CHEMBL511366 | COc1ccc(cc1OC)[C@@H]2N[C@@H](CO)[C@H](O)[C@H]2O | >200 | CHEMBL1014583 | 0 |
| 189 | CHEMBL2011635 | CCCCN1CC[C@H](O)[C@@H](O)[C@H]1CO | >1000 | CHEMBL2015864 | 0 |
| 190 | CHEMBL2204849 | CC(=O)O[C@H]1[C@H](OC(=O)C)[C@H](C=C[C@@H]1n2ccc3ccccc23)n4ccc5ccccc45 | 52.80 | CHEMBL2216697 | 0 |
| 191 | CHEMBL3735383 | Cl.CCCCCCCCN[C@@H]1C=C[C@H](O)[C@H](O)[C@H]1O | >1000 | CHEMBL3737684 | 0 |
| 192 | CHEMBL3133393 | Cl.OC[C@@H]1[C@@H](O)[C@H](O)[C@@H](O)CN1C(=N)NCCCOCC23CC4CC(CC(C4)C2)C3 | 489.00 | CHEMBL3134886 | 0 |
| 193 | CHEMBL307429 | OC[C@H]1NC[C@H](O)[C@@H](O)[C@@H]1O | 241.45 | CHEMBL826964, CHEMBL993569, CHEMBL1040323, CHEMBL647638, CHEMBL3134886, CHEMBL647636, CHEMBL3874807, CHEMBL832765, CHEMBL1820877, CHEMBL647648 | 0 |
| 194 | CHEMBL414447 | COC1CNCC(O)C1O | >1000 | CHEMBL820821 | 0 |
| 195 | CHEMBL117201 | CCCCCCCCN[C@H]1CC(C)[C@@H](O)C(O)[C@@H]1O | 14.70 | CHEMBL647640 | 1 |
| 196 | CHEMBL3359670 | Cc1ccccc1C\N=C\2/N[C@H](CO)[C@H](O)[C@H](O)[C@H]2O | 8.40 | CHEMBL3815659 | 1 |
| 197 | CHEMBL2011636 | OC[C@@H]1[C@H](O)[C@@H](O)CCN1CCCCCOCC23CC4CC(CC(C4)C2)C3 | 3.00 | CHEMBL2015864 | 1 |
| 198 | CHEMBL3354044 | COc1ccccc1c2ccc(COCCCCCN3C[C@H](O)[C@@H](O)[C@H](O)[C@H]3CO)cc2 | 3.00•10^-3^ | CHEMBL3384691 | 1 |
| 199 | CHEMBL35576 | OC[C@@H]1[C@@H](O)[C@H](O)[C@@H](O)[C@@H]2O[C@H]12 | 4.54 | CHEMBL1024152 | 1 |
| 200 | CHEMBL3359668 | OC[C@H]1N\C(=N/Cc2cccc(F)c2)\[C@H](O)[C@@H](O)[C@H]1O | 11.00 | CHEMBL3815659 | 1 |
| 201 | CHEMBL117074 | CCCCCCCCCCNC1CC(C)C(O)C(O)C1O | 2.00 | CHEMBL647640 | 1 |
| 202 | CHEMBL206468 | OC[C@H]1CNC[C@@H](O)[C@@H]1O | 0.37 | CHEMBL1820880, CHEMBL993569, CHEMBL1820877 | 1 |
| 203 | CHEMBL470661 | OC[C@H]1N[C@H]([C@H](O)CCCC[C@@H]2CCC[C@]3(CCCO3)O2)[C@@H](O)[C@@H]1O | 0.01 | CHEMBL1007617 | 1 |
| 204 | CHEMBL3359682 | OC[C@H]1N\C(=N/CC2CCCCCC2)\[C@H](O)[C@@H](O)[C@H]1O | 5.00 | CHEMBL3815659 | 1 |
| 205 | CHEMBL3354635 | OC[C@H]1[C@@H](O)[C@H](O)[C@@H](O)CN1CCCCCOCc2ccc(c3ccccc3)c(c2)C(F)(F)F | 1.00•10^-4^ | CHEMBL3384691 | 1 |
| 206 | CHEMBL3897971 | CCCCCCCCCC\N=C/1\SC[C@@H]2[C@@H](O)[C@H](O)[C@@H](CO)N12 | 1.00 | CHEMBL3869409 | 1 |
| 207 | CHEMBL3354043 | COc1cccc(c1)c2ccc(COCCCCCN3C[C@H](O)[C@@H](O)[C@H](O)[C@H]3CO)cc2 | 0.02 | CHEMBL3384691 | 1 |
| 208 | CHEMBL3359124 | OC[C@H]1N\C(=N/Cc2ccc(F)cc2)\[C@H](O)[C@@H](O)[C@H]1O | 4.70 | CHEMBL3815659 | 1 |
| 209 | CHEMBL3354064 | OC[C@@H]1[C@@H](O)[C@H](O)[C@@H](O)CN1CCCCCOCc2ccc(cn2)c3ccccc3 | 3.00•10^-3^ | CHEMBL3384691 | 1 |
| 210 | CHEMBL3354061 | OC[C@@H]1[C@@H](O)[C@H](O)[C@@H](O)CN1CCCCCOCc2ccc(cc2)c3ccc(F)nc3 | 0.01 | CHEMBL3384691 | 1 |
| 211 | CHEMBL3359677 | OC[C@H]1N\C(=N/CCCc2ccccc2)\[C@H](O)[C@@H](O)[C@H]1O | 1.30 | CHEMBL3815659 | 1 |
| 212 | CHEMBL3359122 | COc1ccc(C\N=C\2/N[C@H](CO)[C@H](O)[C@H](O)[C@H]2O)cc1 | 13.00 | CHEMBL3815659 | 1 |
| 213 | CHEMBL3916914 | CCCCCC\N=C/1\SC[C@@H]2[C@@H](O)[C@H](O)[C@@H](CO)N12 | 16.00 | CHEMBL3869409 | 1 |
| 214 | CHEMBL3354042 | COc1ccc(cc1)c2ccc(COCCCCCN3C[C@H](O)[C@@H](O)[C@H](O)[C@H]3CO)cc2 | 0.01 | CHEMBL3384691 | 1 |
| 215 | CHEMBL1933098 | Cl.C[C@@H]1CNC[C@@H](O)[C@@H](O)[C@@H]1O | 14.00 | CHEMBL1937704 | 1 |
| 216 | CHEMBL3354627 | OC[C@@H]1[C@@H](O)[C@H](O)[C@@H](O)CN1CCCCCOCc2ccc(c3ccccc3)c(c2)C(F)(F)F | 8.00•10^-5^ | CHEMBL3384691 | 1 |
| 217 | CHEMBL2011641 | C[C@@H]1[C@@H](O)[C@H](O)[C@@H](O)CN1CCCCCOCC23CC4CC(CC(C4)C2)C3 | 0.03 | CHEMBL2015864 | 1 |
| 218 | CHEMBL3354050 | OC[C@@H]1[C@@H](O)[C@H](O)[C@@H](O)CN1CCCCCOCc2ccc(cc2)c3ccccc3F | 2.00•10^-3^ | CHEMBL3384691 | 1 |
| 219 | CHEMBL116366 | CCCCCCCCCCN[C@H]1CC(C)[C@@H](O)C(O)[C@@H]1O | 16.60 | CHEMBL647640 | 1 |
| 220 | CHEMBL3359672 | Cc1ccc(C\N=C\2/N[C@H](CO)[C@H](O)[C@H](O)[C@H]2O)cc1 | 3.00 | CHEMBL3815659 | 1 |
| 221 | CHEMBL110830 | O[C@@H]1CNC[C@H](O)[C@H]1O | 5.55 | CHEMBL993569, CHEMBL820821 | 1 |
| 222 | CHEMBL3359678 | OC[C@H]1N\C(=N/C2CC2)\[C@H](O)[C@@H](O)[C@H]1O | 32.00 | CHEMBL3815659 | 1 |
| 223 | CHEMBL3354629 | C[C@@H](OCCCCCN1C[C@H](O)[C@@H](O)[C@H](O)[C@@H]1CO)c2ccc(cc2)c3ccccc3 | 1.00•10^-3^ | CHEMBL3384691 | 1 |
| 224 | CHEMBL469435 | OCC(O)CCCCC(O)CC(O)CCC[C@@H](O)[C@H]1N[C@H](CO)[C@@H](O)[C@@H]1O | 6.60 | CHEMBL1007617 | 1 |
| 225 | CHEMBL3354025 | OC[C@@H]1[C@@H](O)[C@H](O)[C@@H](O)CN1CCCCCOCc2ccc(cc2)c3ccccc3 | 2.00•10^-3^ | CHEMBL3384691 | 1 |
| 226 | CHEMBL311226 | O[C@H]1CCN2C[C@H](O)[C@@H](O)[C@H](O)[C@@H]12 | 19.00 | CHEMBL2342949 | 1 |
| 227 | CHEMBL3354053 | OC[C@@H]1[C@@H](O)[C@H](O)[C@@H](O)CN1CCCCCOCc2ccc(cc2)c3cc(cc(c3)C(F)(F)F)C(F)(F)F | 0.02 | CHEMBL3384691 | 1 |
| 228 | CHEMBL3354048 | OC[C@@H]1[C@@H](O)[C@H](O)[C@@H](O)CN1CCCCCOCc2ccc(cc2)c3ccc(F)cc3 | 1.00•10^-3^ | CHEMBL3384691 | 1 |
| 229 | CHEMBL364554 | CCCCCCC1NC(CO)C(O)C(O)C1O | 12.00 | CHEMBL832785 | 1 |
| 230 | CHEMBL3354062 | OC[C@@H]1[C@@H](O)[C@H](O)[C@@H](O)CN1CCCCCOCc2ccccc2c3ccccc3 | 3.00•10^-3^ | CHEMBL3384691 | 1 |
| 231 | CHEMBL574645 | OC[C@@H]1[C@@H](O)[C@H](O)[C@@H](O)CN1CCCCCOCC23CC4CC(CC(C4)C2)C3 | 1.33•10^-3^ | CHEMBL3384691, CHEMBL1040323, CHEMBL2015864 | 1 |
| 232 | CHEMBL2011632 | OC[C@H]1[C@@H](O)[C@H](O)CCN1CCCCCOCC23CC4CC(CC(C4)C2)C3 | 0.50 | CHEMBL2015864 | 1 |
| 233 | CHEMBL3354045 | OC[C@@H]1[C@@H](O)[C@H](O)[C@@H](O)CN1CCCCCOCc2ccc(cc2)c3ccc(cc3)C#N | 3.00•10^-3^ | CHEMBL3384691 | 1 |
| 234 | CHEMBL3354032 | OC[C@H]1[C@@H](O)[C@H](O)[C@@H](O)CN1CCCCCOCC2C3CC4CC(CC2C4)C3 | 2.00•10^-3^ | CHEMBL3384691 | 1 |
| 235 | CHEMBL1088158 | OC[C@@H]1[C@H](O)[C@H](O)[C@@H](O)CN1CCCCCOCC23CC4CC(CC(C4)C2)C3 | 1.00•10^-3^ | CHEMBL3384691 | 1 |
| 236 | CHEMBL3133387 | Cl.CCCCNC(=N)N1C[C@H](O)[C@@H](O)[C@H](O)[C@H]1CO | 41.00 | CHEMBL3134886 | 1 |
| 237 | CHEMBL3944457 | CCCCCCCCNC(=S)N1[C@H](CO)[C@@H](O)[C@H](O)[C@H]1CO | 12.00 | CHEMBL3869409 | 1 |
| 238 | CHEMBL425308 | CC(O)CC[C@@H]1CC[C@@H]2[C@@H](O)[C@H](O)[C@@H](CO)N12 | 49.00 | CHEMBL1007617 | 1 |
| 239 | CHEMBL485840 | CCCCCCCCCOC(=O)N1C[C@H](O)[C@@H](O)[C@H](O)[C@H]1CO | 0.02 | CHEMBL971353 | 1 |
| 240 | CHEMBL3354057 | OC[C@@H]1[C@@H](O)[C@H](O)[C@@H](O)CN1CCCCCOCc2ccc(cc2)c3ccncc3 | 0.01 | CHEMBL3384691 | 1 |
| 241 | CHEMBL1086996 | OC[C@H]1[C@@H](O)[C@H](O)[C@@H](O)CN1CCCCCOCC23CC4CC(CC(C4)C2)C3 | 0.02 | CHEMBL2015864, CHEMBL3384691 | 1 |
| 242 | CHEMBL4059852 | OCC1(CO)NC[C@H](F)[C@H]1O | 0.18 | CHEMBL4044716 | 1 |
| 243 | CHEMBL3354059 | OC[C@@H]1[C@@H](O)[C@H](O)[C@@H](O)CN1CCCCCOCc2ccc(cc2)c3cncnc3 | 0.03 | CHEMBL3384691 | 1 |
| 244 | CHEMBL498968 | CC(O)CC(O)[C@@H]1CC[C@@H]2[C@H](O)[C@H](O)[C@@H](CO)N12 | 9.50 | CHEMBL1007617 | 1 |
| 245 | CHEMBL470868 | OC[C@H]1N[C@H](C(O)CCC[C@@H]2CCC[C@]3(CCCCO3)O2)[C@@H](O)[C@@H]1O | 0.01 | CHEMBL1007617 | 1 |
| 246 | CHEMBL2011628 | OC[C@@H]1[C@H](O)[C@H](O)CCN1CCCCCOCC23CC4CC(CC(C4)C2)C3 | 6.50 | CHEMBL2015864 | 1 |
| 247 | CHEMBL2164231 | Cl.CCCCCCCCCN[C@H]1[C@H](O)[C@@H](O)[C@H](O)[C@@H](O)[C@H]1OCCCCCCCCC | 4.30•10^-3^ | CHEMBL2168210 | 1 |
| 248 | CHEMBL3354625 | OC[C@@H]1[C@@H](O)[C@H](O)[C@@H](O)CN1CCCCCOCc2ccc(c(F)c2)c3ccccc3 | 8.00•10^-5^ | CHEMBL3384691 | 1 |
| 249 | CHEMBL3354034 | OC[C@H]1[C@@H](O)[C@H](O)[C@@H](O)CN1CCCCCOCc2ccccc2 | 3.00•10^-3^ | CHEMBL3384691 | 1 |
| 250 | CHEMBL1922580 | Cl.CCCCCCCCN[C@@H]1C=C(CO)[C@@H](O)[C@H](O)[C@H]1O | 47.00 | CHEMBL1924471 | 1 |
| 251 | CHEMBL1076754 | CCCCN1C[C@H](O)[C@@H](O)[C@H](O)[C@@H]1CO | 0.25 | CHEMBL3384691 | 1 |
| 252 | CHEMBL511581 | COc1ccc(cc1OC)[C@H]2N[C@H](CO)[C@@H](O)[C@@H]2O | 34.00 | CHEMBL1014583 | 1 |
| 253 | CHEMBL3354023 | OC[C@@H]1[C@@H](O)[C@H](O)[C@@H](O)CN1CCCCCOCc2cc3ccccc3c4ccccc24 | 0.10 | CHEMBL3384691 | 1 |
| 254 | CHEMBL3901083 | CCCCCCCCCCCC\N=C/1\SC[C@@H]2[C@@H](O)[C@H](O)[C@@H](CO)N12 | 1.00 | CHEMBL3869409 | 1 |
| 255 | CHEMBL3354016 | OC[C@@H]1[C@@H](O)[C@H](O)[C@@H](O)CN1CCCCCCOCC23CC4CC(CC(C4)C2)C3 | 2.00•10^-3^ | CHEMBL3384691 | 1 |
| 256 | CHEMBL2204850 | CC(=O)O[C@@H]1C=C[C@H]([C@H](OC(=O)C)[C@H]1O)N2CCc3ccccc23 | 24.30 | CHEMBL2216697 | 1 |
| 257 | CHEMBL3354623 | C[C@@H](OCCCCCN1C[C@H](O)[C@@H](O)[C@H](O)[C@H]1CO)c2ccc(cc2)c3ccccc3 | 3.00•10^-3^ | CHEMBL3384691 | 1 |
| 258 | CHEMBL2011603 | OC[C@@H]1[C@@H](O)C=CCN1CCCCCOCC23CC4CC(CC(C4)C2)C3 | 30.00 | CHEMBL2015864 | 1 |
| 259 | CHEMBL450985 | CCCCCCCCCCCCN1C[C@H](O)[C@@H](O)[C@H](O)[C@H]1CO | 2.00•10^-3^ | CHEMBL971353 | 1 |
| 260 | CHEMBL2011634 | OC[C@@H]1[C@@H](O)[C@@H](O)CCN1CCCCCOCC23CC4CC(CC(C4)C2)C3 | 25.00 | CHEMBL2015864 | 1 |
| 261 | CHEMBL186291 | CCCCCCCCC1NC(CO)C(O)C(O)C1O | 27.00 | CHEMBL832785 | 1 |
| 262 | CHEMBL3354632 | OC[C@H]1[C@@H](O)[C@H](O)[C@@H](O)CN1CC(F)(F)CCCOCc2ccc(cc2)c3ccccc3 | 2.00•10^-3^ | CHEMBL3384691 | 1 |
| 263 | CHEMBL3354051 | OC[C@@H]1[C@@H](O)[C@H](O)[C@@H](O)CN1CCCCCOCc2ccc(cc2)c3ccc(Cl)cc3 | 2.00•10^-3^ | CHEMBL3384691 | 1 |
| 264 | CHEMBL1818436 | OC[C@@H]1NCC[C@@H](O)[C@H]1O | 30.00 | CHEMBL2015864 | 1 |
| 265 | CHEMBL3354631 | OC[C@H]1[C@@H](O)[C@H](O)[C@@H](O)CN1CCCCCOCc2cccc(c2)c3ccccc3 | 2.00•10^-3^ | CHEMBL3384691 | 1 |
| 266 | CHEMBL3354060 | OC[C@@H]1[C@@H](O)[C@H](O)[C@@H](O)CN1CCCCCOCc2ccc(cc2)c3ccnc(F)c3 | 4.00•10^-3^ | CHEMBL3384691 | 1 |
| 267 | CHEMBL3354031 | CC(C)CCC[C@@H](C)[C@H]1CC[C@H]2[C@@H]3CC=C4C[C@H](CC[C@]4(C)[C@H]3CC[C@]12C)OCCCCCN5C[C@H](O)[C@@H](O)[C@H](O)[C@@H]5CO | 0.02 | CHEMBL3384691 | 1 |
| 268 | CHEMBL3354637 | OC[C@H]1[C@@H](O)[C@H](O)[C@@H](O)CN1CCCCCOCc2ccc(c(F)c2)c3ccccc3 | 6.00•10^-4^ | CHEMBL3384691 | 1 |
| 269 | CHEMBL3359669 | OC[C@H]1N\C(=N/Cc2ccccc2)\[C@H](O)[C@@H](O)[C@H]1O | 2.80 | CHEMBL3815659 | 1 |
| 270 | CHEMBL2170199 | OC[C@@H]1[C@@H](O)[C@H](O)[C@@H](O)CN1CCCCCC(=O)NC23CC4CC(CC(C4)C2)C3 | 0.01 | CHEMBL3384691 | 1 |
| 271 | CHEMBL3354624 | OC[C@@H]1[C@@H](O)[C@H](O)[C@@H](O)CN1CCCCCOCc2ccc(cc2F)c3ccccc3 | 8.00•10^-4^ | CHEMBL3384691 | 1 |
| 272 | CHEMBL3354628 | C[C@H](OCCCCCN1C[C@H](O)[C@@H](O)[C@H](O)[C@@H]1CO)c2ccc(cc2)c3ccccc3 | 2.00•10^-3^ | CHEMBL3384691 | 1 |
| 273 | CHEMBL3354621 | OC[C@@H]1[C@@H](O)[C@H](O)[C@@H](O)CN1CCCC(F)(F)COCc2ccc(cc2)c3ccccc3 | 1.00•10^-3^ | CHEMBL3384691 | 1 |
| 274 | CHEMBL3133388 | Cl.CCCCCCCCNC(=N)N1C[C@H](O)[C@@H](O)[C@H](O)[C@H]1CO | 25.00 | CHEMBL3134886 | 1 |
| 275 | CHEMBL2205523 | CC(=O)O[C@@H]1C=C[C@H]([C@H](OC(=O)C)[C@H]1O)n2ccc3ccccc23 | 23.10 | CHEMBL2216697 | 1 |
| 276 | CHEMBL3901171 | OC[C@@H]1[C@@H](O)[C@H](O)[C@@H](O)CN1CCCCc2cn(CCCCOc3ccc4C(=CC(=O)Oc4c3)C(F)(F)F)nn2 | 3.80 | CHEMBL3874807 | 1 |
| 277 | CHEMBL3359666 | CC(C)(C)c1ccc(C\N=C\2/N[C@H](CO)[C@H](O)[C@H](O)[C@H]2O)cc1 | 2.30 | CHEMBL3815659 | 1 |
| 278 | CHEMBL3354046 | OC[C@@H]1[C@@H](O)[C@H](O)[C@@H](O)CN1CCCCCOCc2ccc(cc2)c3cccc(c3)C#N | 2.00•10^-3^ | CHEMBL3384691 | 1 |
| 279 | CHEMBL3354622 | C[C@H](OCCCCCN1C[C@H](O)[C@@H](O)[C@H](O)[C@H]1CO)c2ccc(cc2)c3ccccc3 | 3.00•10^-3^ | CHEMBL3384691 | 1 |
| 280 | CHEMBL3354063 | OC[C@@H]1[C@@H](O)[C@H](O)[C@@H](O)CN1CCCCCOCc2cccc(c2)c3ccccc3 | 2.00•10^-3^ | CHEMBL3384691 | 1 |
| 281 | CHEMBL3354033 | CC(=CCC\C(=C\CC\C(=C\CCOCCCCCN1C[C@H](O)[C@@H](O)[C@H](O)[C@@H]1CO)\C)\C)C | 0.10 | CHEMBL3384691 | 1 |
| 282 | CHEMBL3354636 | OC[C@H]1[C@@H](O)[C@H](O)[C@@H](O)CN1CCCCCOCc2ccc(cc2F)c3ccccc3 | 4.00•10^-4^ | CHEMBL3384691 | 1 |
| 283 | CHEMBL3354040 | Cc1cccc(c1)c2ccc(COCCCCCN3C[C@H](O)[C@@H](O)[C@H](O)[C@H]3CO)cc2 | 2.00•10^-3^ | CHEMBL3384691 | 1 |
| 284 | CHEMBL469427 | OC[C@H]1N[C@H]([C@H](O)CCCCCCCC(=O)CCCCO[C@@H]2O[C@H](CO)[C@@H](O)[C@H](O)[C@H]2O)[C@@H](O)[C@@H]1O | 0.01 | CHEMBL1007617 | 1 |
| 285 | CHEMBL3359681 | OC[C@H]1N\C(=N/CC2CCCCC2)\[C@H](O)[C@@H](O)[C@H]1O | 1.60 | CHEMBL3815659 | 1 |
| 286 | CHEMBL2011611 | CCCCN1CC=C[C@H](O)[C@@H]1CO | 25.00 | CHEMBL2015864 | 1 |
| 287 | CHEMBL469437 | OCC(O)CC(O)[C@@H]1CC[C@@H]2[C@H](O)[C@H](O)[C@@H](CO)N12 | 25.40 | CHEMBL1007617 | 1 |
| 288 | CHEMBL3354039 | Cc1ccc(cc1)c2ccc(COCCCCCN3C[C@H](O)[C@@H](O)[C@H](O)[C@H]3CO)cc2 | 3.00•10^-3^ | CHEMBL3384691 | 1 |
| 289 | CHEMBL1029 | CCCCN1C[C@H](O)[C@@H](O)[C@H](O)[C@H]1CO | 0.23 | CHEMBL1040323, CHEMBL3384691, CHEMBL2015864 | 1 |
| 290 | CHEMBL3354056 | OC[C@@H]1[C@@H](O)[C@H](O)[C@@H](O)CN1CCCCCOCc2ccc(cc2)c3ccc4OCCCOc4c3 | 0.02 | CHEMBL3384691 | 1 |
| 291 | CHEMBL3354037 | OC[C@H]1[C@@H](O)[C@H](O)[C@@H](O)CN1CCCCCOCc2ccc3ccc4cccc5ccc2c3c45 | 1.00•10^-3^ | CHEMBL3384691 | 1 |
| 292 | CHEMBL3359667 | OC[C@H]1N\C(=N/Cc2ccccc2F)\[C@H](O)[C@@H](O)[C@H]1O | 1.80 | CHEMBL3815659 | 1 |
| 293 | CHEMBL2011626 | OC[C@@H]1[C@@H](O)[C@H](O)CCN1CCCCCOCC23CC4CC(CC(C4)C2)C3 | 1.00 | CHEMBL2015864 | 1 |
| 294 | CHEMBL3354633 | OC[C@H]1[C@@H](O)[C@H](O)[C@@H](O)CN1CCCC(F)(F)COCc2ccc(cc2)c3ccccc3 | 2.00•10^-3^ | CHEMBL3384691 | 1 |
| 295 | CHEMBL3354054 | OC[C@@H]1[C@@H](O)[C@H](O)[C@@H](O)CN1CCCCCOCc2ccc(cc2)c3ccc4OCOc4c3 | 2.00•10^-3^ | CHEMBL3384691 | 1 |
| 296 | CHEMBL3354638 | OC[C@@H]1[C@H](O)[C@H](O)[C@@H](O)CN1CCCCCOCc2ccc(cc2)c3ccccc3 | 2.00•10^-3^ | CHEMBL3384691 | 1 |
| 297 | CHEMBL1818433 | OC[C@H]1CNC[C@@H](O)[C@H]1O | 28.60 | CHEMBL1820880, CHEMBL1820877 | 1 |
| 298 | CHEMBL3354058 | OC[C@@H]1[C@@H](O)[C@H](O)[C@@H](O)CN1CCCCCOCc2ccc(cc2)c3cccnc3 | 0.01 | CHEMBL3384691 | 1 |
| 299 | CHEMBL3133391 | Cl.OC[C@@H]1[C@@H](O)[C@H](O)[C@@H](O)CN1C(=N)NCc2ccccc2 | 4.30 | CHEMBL3134886 | 1 |
| 300 | CHEMBL3354015 | OC[C@@H]1[C@@H](O)[C@H](O)[C@@H](O)CN1CCCC\C=C/C23CC4CC(CC(C4)C2)C3 | 0.01 | CHEMBL3384691 | 1 |
| 301 | CHEMBL3935498 | CCCCCCCCCCCCNC(=S)N1[C@H](CO)[C@@H](O)[C@H](O)[C@H]1CO | 0.16 | CHEMBL3869409 | 1 |
| 302 | CHEMBL2029773 | Cl.CCCCCCCCSCC[C@H]1NC[C@@H](O)[C@H](O)[C@H]1O | 2.00 | CHEMBL2032683 | 1 |
| 303 | CHEMBL3359123 | OC[C@H]1N\C(=N/Cc2ccc(cc2)C(F)(F)F)\[C@H](O)[C@@H](O)[C@H]1O | 2.80 | CHEMBL3815659 | 1 |
| 304 | CHEMBL2114289 | C[C@@H]1CC(NCCc2ccccc2)[C@@H](O)[C@@H](O)[C@@H]1O | 0.38 | CHEMBL647640 | 1 |
| 305 | CHEMBL3354041 | Cc1ccccc1c2ccc(COCCCCCN3C[C@H](O)[C@@H](O)[C@H](O)[C@H]3CO)cc2 | 5.00•10^-4^ | CHEMBL3384691 | 1 |
| 306 | CHEMBL3354027 | OC[C@H]1[C@@H](O)[C@H](O)[C@@H](O)CN1CCCCCC(=O)NC23CC4CC(CC(C4)C2)C3 | 0.02 | CHEMBL3384691 | 1 |
| 307 | CHEMBL3354030 | OC[C@H]1[C@@H](O)[C@H](O)[C@@H](O)CN1CCCCCCC23CC4CC(CC(C4)C2)C3 | 0.02 | CHEMBL3384691 | 1 |
| 308 | CHEMBL3907555 | CCCCCCCCCCNC(=S)N1[C@H](CO)[C@@H](O)[C@H](O)[C@H]1CO | 0.74 | CHEMBL3869409 | 1 |
| 309 | CHEMBL3354047 | OC[C@@H]1[C@@H](O)[C@H](O)[C@@H](O)CN1CCCCCOCc2ccc(cc2)c3ccccc3C#N | 0.01 | CHEMBL3384691 | 1 |
| 310 | CHEMBL3359680 | OC[C@H]1N\C(=N/CC2CC2)\[C@H](O)[C@@H](O)[C@H]1O | 19.00 | CHEMBL3815659 | 1 |
| 311 | CHEMBL3354017 | OC[C@@H]1[C@@H](O)[C@H](O)[C@@H](O)CN1CCCCCCC23CC4CC(CC(C4)C2)C3 | 0.01 | CHEMBL3384691 | 1 |
| 312 | CHEMBL3359671 | Cc1cccc(C\N=C\2/N[C@H](CO)[C@H](O)[C@H](O)[C@H]2O)c1 | 5.10 | CHEMBL3815659 | 1 |
| 313 | CHEMBL470660 | OCCCCC(=O)CCCCCCC[C@@H](O)[C@H]1N[C@H](CO)[C@@H](O)[C@@H]1O | 0.01 | CHEMBL1007617 | 1 |
| 314 | CHEMBL3354026 | OC[C@H]1[C@@H](O)[C@H](O)[C@@H](O)CN1CCCCCOCC2CCCCC2 | 0.01 | CHEMBL3384691 | 1 |
| 315 | CHEMBL323939 | CC1C[C@H](NCCCCc2ccccc2)[C@@H](O)C(O)[C@@H]1O | 10.20 | CHEMBL647640 | 1 |
| 316 | CHEMBL3359674 | CCCCCCC\N=C\1/N[C@H](CO)[C@H](O)[C@H](O)[C@H]1O | 2.20 | CHEMBL3815659 | 1 |
| 317 | CHEMBL2011612 | OC[C@H]1[C@@H](O)C=CCN1CCCCCOCC23CC4CC(CC(C4)C2)C3 | 10.00 | CHEMBL2015864 | 1 |
| 318 | CHEMBL3359673 | CCC\N=C\1/N[C@H](CO)[C@H](O)[C@H](O)[C@H]1O | 4.80 | CHEMBL3815659 | 1 |
| 319 | CHEMBL3939205 | CCCCCCCC\N=C/1\SC[C@@H]2[C@@H](O)[C@H](O)[C@@H](CO)N12 | 2.40 | CHEMBL3869409 | 1 |
| 320 | CHEMBL3354052 | OC[C@@H]1[C@@H](O)[C@H](O)[C@@H](O)CN1CCCCCOCc2ccc(cc2)c3ccc(cc3)C(F)(F)F | 0.01 | CHEMBL3384691 | 1 |
| 321 | CHEMBL291020 | O[C@H]1[C@H](O)[C@@H](O)[C@@H]2O[C@@H]2[C@@H]1O | 30.00 | CHEMBL4007594 | 1 |
| 322 | CHEMBL3354020 | CC(=CCC\C(=C\CC\C(=C\CCOCCCCCN1C[C@H](O)[C@@H](O)[C@H](O)[C@H]1CO)\C)\C)C | 0.10 | CHEMBL3384691 | 1 |
| 323 | CHEMBL3354019 | OC[C@@H]1[C@@H](O)[C@H](O)[C@@H](O)CN1CCCCCOCC2C3CC4CC(CC2C4)C3 | 1.00•10^-3^ | CHEMBL3384691 | 1 |
| 324 | CHEMBL1086997 | CCCCN1C[C@H](O)[C@@H](O)[C@@H](O)[C@H]1CO | 0.30 | CHEMBL3384691 | 1 |

# **Table S2.** The dataset splits into training, validation and test sets.

| **No.** | **Compound ID** | **Class** | **Set** | | | | | | | | | |
| --- | --- | --- | --- | --- | --- | --- | --- | --- | --- | --- | --- | --- |
|  |  |  | **Split1** | **Split2** | **Split3** | **Split4** | **Split5** | **Split6** | **Split7** | **Split8** | **Split9** | **Split10** |
| 1 | CHEMBL3736317 | 0 | Training | Training | Test | Training | Training | Training | Training | Training | Validation | Training |
| 2 | CHEMBL82895 | 0 | Training | Training | Test | Training | Training | Training | Training | Test | Training | Test |
| 3 | CHEMBL2011623 | 0 | Training | Test | Training | Training | Validation | Training | Validation | Training | Training | Training |
| 4 | CHEMBL84272 | 0 | Validation | Training | Training | Test | Training | Validation | Test | Training | Training | Validation |
| 5 | CHEMBL501385 | 0 | Training | Validation | Training | Training | Training | Test | Validation | Training | Validation | Training |
| 6 | CHEMBL83278 | 0 | Training | Training | Training | Test | Training | Training | Training | Test | Training | Test |
| 7 | CHEMBL3739825 | 0 | Training | Training | Training | Training | Training | Training | Training | Validation | Training | Training |
| 8 | CHEMBL2011637 | 0 | Validation | Training | Test | Training | Test | Training | Training | Validation | Training | Training |
| 9 | CHEMBL3736333 | 0 | Training | Training | Training | Validation | Test | Training | Test | Training | Test | Test |
| 10 | CHEMBL4085739 | 0 | Training | Training | Training | Training | Training | Test | Validation | Training | Training | Training |
| 11 | CHEMBL186150 | 0 | Training | Training | Test | Training | Validation | Training | Training | Training | Training | Training |
| 12 | CHEMBL2011621 | 0 | Training | Training | Training | Test | Training | Training | Training | Training | Test | Training |
| 13 | CHEMBL87120 | 0 | Test | Training | Training | Test | Training | Training | Training | Training | Training | Validation |
| 14 | CHEMBL2011640 | 0 | Training | Training | Validation | Test | Training | Training | Training | Validation | Training | Training |
| 15 | CHEMBL84104 | 0 | Training | Training | Validation | Training | Training | Training | Training | Test | Test | Test |
| 16 | CHEMBL2011607 | 0 | Training | Training | Training | Training | Training | Training | Training | Validation | Training | Test |
| 17 | CHEMBL362754 | 0 | Training | Training | Test | Training | Training | Test | Test | Validation | Test | Test |
| 18 | CHEMBL2011624 | 0 | Training | Training | Training | Training | Training | Validation | Training | Training | Validation | Training |
| 19 | CHEMBL2011614 | 0 | Training | Validation | Training | Training | Training | Validation | Test | Training | Training | Training |
| 20 | CHEMBL3735749 | 0 | Training | Validation | Test | Training | Training | Training | Validation | Training | Test | Training |
| 21 | CHEMBL3754250 | 0 | Training | Training | Training | Training | Training | Training | Training | Validation | Training | Training |
| 22 | CHEMBL11216 | 0 | Training | Training | Training | Training | Training | Training | Validation | Test | Training | Validation |
| 23 | CHEMBL2011613 | 0 | Training | Training | Validation | Test | Validation | Training | Training | Test | Training | Training |
| 24 | CHEMBL2011608 | 0 | Training | Training | Training | Training | Training | Test | Training | Training | Training | Training |
| 25 | CHEMBL577370 | 0 | Test | Training | Training | Validation | Training | Validation | Training | Training | Training | Training |
| 26 | CHEMBL573995 | 0 | Validation | Training | Training | Training | Training | Training | Training | Training | Training | Training |
| 27 | CHEMBL4096086 | 0 | Validation | Test | Training | Training | Training | Test | Test | Test | Validation | Training |
| 28 | CHEMBL3736427 | 0 | Training | Validation | Test | Validation | Training | Training | Training | Training | Training | Validation |
| 29 | CHEMBL3735899 | 0 | Training | Training | Test | Training | Training | Training | Training | Validation | Training | Training |
| 30 | CHEMBL184028 | 0 | Training | Validation | Training | Training | Training | Training | Training | Training | Training | Validation |
| 31 | CHEMBL88408 | 0 | Training | Validation | Training | Training | Training | Training | Validation | Training | Training | Test |
| 32 | CHEMBL468062 | 0 | Training | Training | Test | Training | Validation | Training | Training | Training | Validation | Training |
| 33 | CHEMBL2011638 | 1 | Training | Test | Validation | Training | Training | Training | Training | Test | Test | Validation |
| 34 | CHEMBL470659 | 1 | Validation | Validation | Training | Training | Training | Test | Test | Test | Training | Training |
| 35 | CHEMBL3354620 | 1 | Training | Validation | Training | Training | Training | Training | Training | Training | Validation | Training |
| 36 | CHEMBL115510 | 1 | Training | Training | Training | Training | Training | Training | Test | Test | Training | Training |
| 37 | CHEMBL2011642 | 1 | Training | Training | Test | Training | Training | Training | Training | Training | Training | Training |
| 38 | CHEMBL324840 | 1 | Validation | Training | Training | Test | Training | Test | Training | Training | Training | Validation |
| 39 | CHEMBL186068 | 1 | Training | Training | Training | Training | Training | Training | Training | Training | Training | Validation |
| 40 | CHEMBL3354028 | 1 | Validation | Training | Validation | Training | Training | Training | Training | Training | Training | Training |
| 41 | CHEMBL3354036 | 1 | Training | Training | Training | Training | Training | Validation | Training | Training | Validation | Training |
| 42 | CHEMBL3354014 | 1 | Test | Training | Training | Validation | Training | Validation | Training | Test | Training | Training |
| 43 | CHEMBL3815031 | 1 | Training | Training | Training | Test | Test | Training | Training | Test | Validation | Training |
| 44 | CHEMBL3354021 | 1 | Training | Training | Validation | Training | Training | Test | Training | Test | Validation | Training |
| 45 | CHEMBL3354038 | 1 | Validation | Test | Test | Training | Training | Test | Training | Training | Training | Training |
| 46 | CHEMBL2011629 | 1 | Training | Training | Training | Training | Training | Training | Training | Training | Validation | Test |
| 47 | CHEMBL513394 | 1 | Test | Training | Training | Training | Validation | Training | Validation | Training | Training | Training |
| 48 | CHEMBL326599 | 1 | Training | Test | Validation | Training | Training | Training | Training | Training | Training | Validation |
| 49 | CHEMBL3354029 | 1 | Validation | Training | Training | Training | Test | Training | Training | Training | Training | Training |
| 50 | CHEMBL2011631 | 1 | Training | Training | Training | Training | Training | Training | Training | Test | Training | Validation |
| 51 | CHEMBL3354626 | 1 | Validation | Test | Validation | Training | Training | Test | Validation | Validation | Training | Training |
| 52 | CHEMBL86865 | 1 | Validation | Training | Test | Training | Test | Training | Training | Training | Training | Training |
| 53 | CHEMBL314757 | 1 | Training | Training | Training | Training | Training | Training | Test | Training | Training | Validation |
| 54 | CHEMBL3354022 | 1 | Training | Test | Training | Training | Training | Training | Training | Training | Training | Training |
| 55 | CHEMBL3354024 | 1 | Test | Training | Training | Training | Training | Validation | Validation | Training | Training | Validation |
| 56 | CHEMBL469844 | 1 | Training | Training | Validation | Test | Training | Training | Test | Test | Validation | Training |
| 57 | CHEMBL3354035 | 1 | Test | Training | Test | Training | Training | Training | Validation | Training | Training | Training |
| 58 | CHEMBL3359675 | 1 | Training | Training | Training | Validation | Training | Training | Training | Test | Validation | Training |
| 59 | CHEMBL3354049 | 1 | Training | Training | Training | Test | Validation | Training | Training | Training | Validation | Training |
| 60 | CHEMBL3354630 | 1 | Training | Training | Validation | Training | Validation | Validation | Training | Test | Training | Training |
| 61 | CHEMBL3354634 | 1 | Training | Training | Validation | Training | Training | Test | Training | Test | Training | Validation |
| 62 | CHEMBL3354018 | 1 | Training | Training | Training | Validation | Validation | Training | Validation | Validation | Validation | Training |
| 63 | CHEMBL3972306 | 1 | Training | Training | Validation | Test | Training | Validation | Test | Validation | Training | Test |
| 64 | CHEMBL3354055 | 1 | Validation | Training | Training | Training | Test | Training | Validation | Validation | Test | Test |
| 65 | CHEMBL9020 | 0 | Validation | Training | Training | Test | Training | Training | Test | Training | Training | Validation |
| 66 | CHEMBL2011601 | 0 | Training | Training | Training | Training | Training | Training | Training | Training | Training | Validation |
| 67 | CHEMBL356023 | 0 | Training | Training | Training | Training | Training | Training | Training | Training | Test | Training |
| 68 | CHEMBL3734817 | 0 | Training | Validation | Validation | Training | Training | Training | Training | Training | Validation | Training |
| 69 | CHEMBL2011602 | 0 | Validation | Validation | Training | Training | Training | Training | Training | Training | Training | Test |
| 70 | CHEMBL445624 | 0 | Test | Test | Training | Training | Training | Training | Training | Training | Validation | Training |
| 71 | CHEMBL511823 | 0 | Training | Training | Validation | Training | Training | Training | Training | Training | Training | Test |
| 72 | CHEMBL3980365 | 0 | Validation | Training | Training | Test | Test | Training | Validation | Training | Training | Training |
| 73 | CHEMBL313295 | 0 | Training | Training | Test | Training | Training | Training | Training | Training | Test | Test |
| 74 | CHEMBL2011610 | 0 | Training | Test | Test | Training | Training | Training | Validation | Training | Training | Training |
| 75 | CHEMBL3736274 | 0 | Training | Training | Training | Training | Training | Validation | Validation | Training | Validation | Training |
| 76 | CHEMBL2204848 | 0 | Training | Training | Training | Training | Training | Training | Training | Validation | Test | Training |
| 77 | CHEMBL2207396 | 0 | Test | Training | Training | Validation | Test | Training | Test | Training | Training | Training |
| 78 | CHEMBL2011630 | 0 | Training | Test | Training | Validation | Test | Training | Validation | Training | Training | Training |
| 79 | CHEMBL2011633 | 0 | Test | Training | Test | Training | Training | Training | Training | Training | Training | Training |
| 80 | CHEMBL275285 | 0 | Training | Validation | Training | Training | Training | Test | Training | Training | Training | Training |
| 81 | CHEMBL3970812 | 0 | Training | Training | Training | Training | Validation | Test | Training | Training | Validation | Training |
| 82 | CHEMBL369297 | 0 | Training | Training | Training | Training | Training | Test | Training | Test | Test | Training |
| 83 | CHEMBL2011605 | 0 | Test | Training | Training | Training | Validation | Validation | Training | Training | Training | Training |
| 84 | CHEMBL2011609 | 0 | Validation | Training | Training | Training | Training | Training | Training | Test | Training | Training |
| 85 | CHEMBL463872 | 0 | Training | Validation | Training | Training | Training | Training | Training | Validation | Validation | Test |
| 86 | CHEMBL86772 | 0 | Training | Validation | Training | Training | Training | Test | Training | Test | Training | Training |
| 87 | CHEMBL2011617 | 0 | Training | Training | Training | Training | Training | Training | Validation | Training | Test | Training |
| 88 | CHEMBL3735277 | 0 | Validation | Training | Test | Training | Validation | Training | Training | Training | Training | Training |
| 89 | CHEMBL152232 | 0 | Validation | Validation | Test | Test | Training | Training | Training | Training | Training | Training |
| 90 | CHEMBL421340 | 0 | Test | Training | Training | Training | Training | Training | Training | Training | Training | Training |
| 91 | CHEMBL2206827 | 0 | Training | Test | Test | Test | Test | Training | Validation | Training | Training | Test |
| 92 | CHEMBL1561 | 0 | Training | Test | Training | Training | Validation | Training | Validation | Validation | Training | Training |
| 93 | CHEMBL11516 | 0 | Training | Training | Training | Training | Validation | Training | Training | Training | Training | Training |
| 94 | CHEMBL314403 | 0 | Training | Training | Validation | Training | Test | Training | Training | Training | Training | Validation |
| 95 | CHEMBL3742008 | 0 | Test | Training | Training | Training | Training | Training | Training | Test | Training | Test |
| 96 | CHEMBL507492 | 0 | Test | Training | Validation | Training | Validation | Training | Training | Training | Training | Training |
| 97 | CHEMBL313375 | 0 | Training | Training | Training | Training | Training | Validation | Validation | Training | Training | Training |
| 98 | CHEMBL1818439 | 0 | Training | Training | Training | Training | Training | Validation | Training | Training | Training | Training |
| 99 | CHEMBL574865 | 0 | Training | Training | Training | Training | Test | Training | Training | Test | Training | Training |
| 100 | CHEMBL464053 | 0 | Training | Training | Validation | Test | Training | Training | Test | Training | Training | Validation |
| 101 | CHEMBL1933096 | 0 | Training | Training | Validation | Training | Validation | Training | Training | Test | Test | Training |
| 102 | CHEMBL3740658 | 0 | Training | Training | Training | Training | Training | Training | Test | Validation | Test | Training |
| 103 | CHEMBL2011620 | 0 | Training | Training | Validation | Test | Validation | Validation | Validation | Training | Training | Training |
| 104 | CHEMBL3736474 | 0 | Training | Training | Training | Test | Training | Validation | Training | Training | Training | Training |
| 105 | CHEMBL448744 | 0 | Training | Training | Test | Validation | Training | Training | Training | Test | Training | Training |
| 106 | CHEMBL3740283 | 0 | Training | Training | Training | Training | Training | Training | Training | Training | Validation | Training |
| 107 | CHEMBL3359684 | 0 | Test | Training | Training | Training | Training | Training | Training | Training | Training | Training |
| 108 | CHEMBL584580 | 0 | Training | Training | Training | Training | Training | Validation | Training | Training | Training | Training |
| 109 | CHEMBL1236649 | 0 | Training | Training | Training | Test | Validation | Training | Test | Validation | Training | Training |
| 110 | CHEMBL465105 | 0 | Validation | Training | Training | Test | Training | Training | Validation | Training | Training | Training |
| 111 | CHEMBL275509 | 0 | Training | Training | Training | Training | Training | Training | Training | Training | Validation | Validation |
| 112 | CHEMBL3133385 | 0 | Training | Training | Validation | Training | Training | Training | Training | Training | Training | Training |
| 113 | CHEMBL583176 | 0 | Training | Training | Training | Training | Training | Test | Training | Training | Test | Training |
| 114 | CHEMBL503122 | 0 | Training | Training | Training | Training | Training | Test | Training | Training | Training | Training |
| 115 | CHEMBL2011618 | 0 | Training | Training | Test | Training | Training | Test | Training | Test | Training | Training |
| 116 | CHEMBL502396 | 0 | Test | Test | Training | Training | Test | Training | Validation | Training | Training | Validation |
| 117 | CHEMBL1818435 | 0 | Test | Validation | Training | Test | Training | Training | Training | Validation | Test | Training |
| 118 | CHEMBL3359683 | 0 | Validation | Training | Training | Training | Test | Test | Training | Training | Validation | Training |
| 119 | CHEMBL11477 | 0 | Test | Test | Validation | Validation | Test | Validation | Training | Validation | Training | Validation |
| 120 | CHEMBL465034 | 0 | Test | Test | Validation | Training | Validation | Training | Training | Training | Training | Training |
| 121 | CHEMBL3133390 | 0 | Validation | Test | Training | Test | Training | Training | Training | Validation | Validation | Training |
| 122 | CHEMBL11322 | 0 | Validation | Training | Test | Training | Validation | Training | Training | Training | Test | Training |
| 123 | CHEMBL108084 | 0 | Training | Training | Training | Validation | Training | Training | Training | Training | Training | Training |
| 124 | CHEMBL505237 | 0 | Training | Training | Training | Training | Training | Test | Training | Training | Training | Training |
| 125 | CHEMBL2011622 | 0 | Training | Test | Training | Validation | Training | Training | Training | Training | Training | Training |
| 126 | CHEMBL80254 | 0 | Training | Test | Validation | Training | Validation | Training | Training | Training | Training | Training |
| 127 | CHEMBL2011639 | 0 | Training | Training | Test | Training | Validation | Training | Training | Training | Test | Training |
| 128 | CHEMBL259905 | 0 | Training | Training | Training | Validation | Training | Training | Training | Test | Test | Training |
| 129 | CHEMBL3736146 | 0 | Training | Test | Training | Training | Training | Training | Training | Test | Training | Validation |
| 130 | CHEMBL11566 | 0 | Training | Validation | Training | Test | Test | Training | Training | Training | Test | Validation |
| 131 | CHEMBL3891002 | 0 | Training | Training | Training | Training | Validation | Training | Validation | Validation | Training | Training |
| 132 | CHEMBL1566 | 0 | Validation | Training | Test | Training | Training | Validation | Training | Training | Training | Test |
| 133 | CHEMBL276643 | 0 | Training | Validation | Training | Training | Training | Training | Test | Training | Test | Test |
| 134 | CHEMBL468063 | 0 | Test | Training | Test | Training | Training | Training | Training | Validation | Training | Training |
| 135 | CHEMBL456583 | 0 | Training | Training | Training | Test | Test | Validation | Training | Training | Training | Training |
| 136 | CHEMBL312653 | 0 | Training | Training | Test | Training | Training | Validation | Training | Training | Training | Training |
| 137 | CHEMBL273605 | 0 | Test | Training | Training | Training | Training | Training | Validation | Validation | Training | Validation |
| 138 | CHEMBL3742045 | 0 | Training | Test | Training | Training | Training | Training | Training | Training | Training | Training |
| 139 | CHEMBL150938 | 0 | Validation | Training | Training | Training | Training | Training | Validation | Training | Training | Test |
| 140 | CHEMBL1818321 | 0 | Training | Test | Training | Training | Training | Training | Test | Training | Training | Test |
| 141 | CHEMBL3753015 | 0 | Validation | Training | Validation | Test | Training | Training | Validation | Training | Training | Test |
| 142 | CHEMBL466791 | 0 | Test | Validation | Training | Training | Training | Test | Training | Training | Training | Training |
| 143 | CHEMBL3133386 | 0 | Test | Test | Training | Training | Training | Training | Training | Training | Validation | Training |
| 144 | CHEMBL3943978 | 0 | Training | Training | Test | Test | Validation | Training | Training | Training | Test | Test |
| 145 | CHEMBL11766 | 0 | Test | Training | Training | Training | Training | Training | Test | Training | Training | Validation |
| 146 | CHEMBL3752112 | 0 | Training | Test | Validation | Test | Validation | Test | Training | Training | Training | Training |
| 147 | CHEMBL2115197 | 0 | Training | Test | Validation | Training | Training | Training | Test | Training | Validation | Training |
| 148 | CHEMBL2207397 | 0 | Validation | Training | Training | Validation | Training | Test | Training | Training | Training | Training |
| 149 | CHEMBL3736331 | 0 | Training | Training | Validation | Training | Training | Training | Validation | Training | Validation | Training |
| 150 | CHEMBL11452 | 0 | Test | Test | Training | Training | Test | Training | Training | Validation | Training | Test |
| 151 | CHEMBL2011616 | 0 | Training | Validation | Training | Training | Test | Training | Training | Test | Training | Training |
| 152 | CHEMBL2011619 | 0 | Training | Test | Training | Training | Validation | Test | Test | Training | Training | Training |
| 153 | CHEMBL463871 | 0 | Training | Training | Training | Validation | Test | Validation | Training | Test | Training | Validation |
| 154 | CHEMBL2011606 | 0 | Training | Training | Training | Training | Training | Validation | Training | Training | Validation | Validation |
| 155 | CHEMBL416887 | 0 | Validation | Training | Test | Training | Training | Training | Training | Training | Test | Training |
| 156 | CHEMBL2011615 | 0 | Test | Training | Training | Training | Training | Training | Test | Training | Training | Training |
| 157 | CHEMBL2206826 | 0 | Training | Training | Training | Validation | Validation | Training | Training | Training | Training | Training |
| 158 | CHEMBL3735475 | 0 | Training | Validation | Training | Training | Validation | Training | Training | Training | Training | Validation |
| 159 | CHEMBL3359679 | 0 | Training | Validation | Training | Training | Training | Test | Training | Training | Training | Training |
| 160 | CHEMBL187158 | 0 | Training | Test | Training | Validation | Training | Test | Training | Training | Training | Training |
| 161 | CHEMBL3133389 | 0 | Training | Training | Training | Training | Training | Training | Training | Training | Training | Training |
| 162 | CHEMBL1163254 | 0 | Training | Validation | Training | Test | Training | Training | Training | Training | Training | Test |
| 163 | CHEMBL1818437 | 0 | Test | Training | Validation | Training | Training | Validation | Training | Training | Training | Training |
| 164 | CHEMBL3736234 | 0 | Training | Training | Training | Training | Test | Training | Validation | Training | Test | Test |
| 165 | CHEMBL2011625 | 0 | Training | Training | Training | Training | Validation | Test | Training | Training | Training | Training |
| 166 | CHEMBL3735278 | 0 | Test | Test | Training | Test | Training | Training | Training | Validation | Training | Training |
| 167 | CHEMBL2409320 | 0 | Training | Training | Validation | Validation | Training | Test | Training | Training | Training | Training |
| 168 | CHEMBL276030 | 0 | Training | Training | Training | Training | Test | Validation | Training | Test | Training | Training |
| 169 | CHEMBL1818434 | 0 | Training | Validation | Training | Training | Training | Validation | Training | Training | Training | Validation |
| 170 | CHEMBL2011627 | 0 | Training | Training | Training | Validation | Training | Test | Training | Validation | Training | Training |
| 171 | CHEMBL3814915 | 0 | Test | Validation | Validation | Training | Training | Validation | Validation | Training | Training | Validation |
| 172 | CHEMBL11614 | 0 | Training | Validation | Test | Training | Validation | Validation | Training | Training | Training | Training |
| 173 | CHEMBL3133392 | 0 | Validation | Training | Validation | Training | Test | Validation | Training | Training | Training | Training |
| 174 | CHEMBL269410 | 0 | Training | Training | Training | Training | Training | Training | Training | Training | Test | Training |
| 175 | CHEMBL445980 | 0 | Training | Training | Training | Training | Validation | Training | Test | Training | Training | Training |
| 176 | CHEMBL1818438 | 0 | Training | Training | Test | Training | Training | Training | Training | Validation | Validation | Test |
| 177 | CHEMBL2011604 | 0 | Training | Training | Training | Test | Training | Training | Validation | Validation | Training | Training |
| 178 | CHEMBL511534 | 0 | Validation | Training | Training | Training | Test | Training | Training | Training | Training | Training |
| 179 | CHEMBL84940 | 0 | Training | Training | Training | Training | Test | Training | Test | Validation | Test | Training |
| 180 | CHEMBL185536 | 0 | Training | Training | Training | Test | Training | Training | Training | Training | Test | Training |
| 181 | CHEMBL1628264 | 0 | Training | Training | Validation | Training | Training | Training | Training | Test | Test | Test |
| 182 | CHEMBL3349573 | 0 | Test | Training | Test | Training | Training | Training | Training | Training | Validation | Test |
| 183 | CHEMBL357108 | 0 | Training | Test | Training | Training | Validation | Test | Test | Validation | Training | Training |
| 184 | CHEMBL88413 | 0 | Training | Training | Training | Validation | Training | Training | Validation | Training | Training | Training |
| 185 | CHEMBL1922579 | 0 | Training | Training | Training | Training | Training | Training | Training | Training | Validation | Training |
| 186 | CHEMBL368121 | 0 | Test | Training | Training | Training | Validation | Validation | Training | Training | Training | Training |
| 187 | CHEMBL454551 | 0 | Training | Test | Training | Validation | Test | Training | Training | Training | Training | Training |
| 188 | CHEMBL511366 | 0 | Training | Training | Training | Training | Validation | Training | Training | Training | Validation | Training |
| 189 | CHEMBL2011635 | 0 | Validation | Training | Training | Training | Training | Training | Test | Validation | Validation | Training |
| 190 | CHEMBL2204849 | 0 | Training | Training | Test | Training | Training | Training | Training | Training | Training | Training |
| 191 | CHEMBL3735383 | 0 | Training | Training | Test | Test | Training | Training | Training | Validation | Validation | Test |
| 192 | CHEMBL3133393 | 0 | Training | Validation | Validation | Validation | Training | Training | Validation | Validation | Training | Training |
| 193 | CHEMBL307429 | 0 | Training | Training | Training | Training | Training | Training | Training | Training | Test | Training |
| 194 | CHEMBL414447 | 0 | Training | Training | Training | Test | Test | Training | Training | Validation | Validation | Training |
| 195 | CHEMBL117201 | 1 | Training | Training | Training | Training | Training | Test | Training | Training | Validation | Validation |
| 196 | CHEMBL3359670 | 1 | Training | Training | Validation | Test | Validation | Test | Test | Training | Training | Validation |
| 197 | CHEMBL2011636 | 1 | Training | Training | Training | Training | Test | Training | Training | Training | Test | Training |
| 198 | CHEMBL3354044 | 1 | Training | Training | Training | Training | Training | Training | Training | Training | Training | Test |
| 199 | CHEMBL35576 | 1 | Test | Validation | Training | Training | Training | Training | Training | Test | Training | Test |
| 200 | CHEMBL3359668 | 1 | Validation | Test | Training | Training | Training | Test | Validation | Training | Training | Training |
| 201 | CHEMBL117074 | 1 | Training | Training | Training | Training | Test | Test | Training | Test | Training | Training |
| 202 | CHEMBL206468 | 1 | Training | Training | Test | Training | Test | Test | Test | Training | Training | Validation |
| 203 | CHEMBL470661 | 1 | Training | Test | Training | Training | Training | Training | Test | Training | Validation | Test |
| 204 | CHEMBL3359682 | 1 | Validation | Training | Training | Test | Training | Test | Test | Test | Validation | Training |
| 205 | CHEMBL3354635 | 1 | Training | Training | Validation | Training | Training | Training | Training | Training | Training | Training |
| 206 | CHEMBL3897971 | 1 | Training | Training | Training | Validation | Training | Training | Training | Validation | Training | Training |
| 207 | CHEMBL3354043 | 1 | Training | Training | Training | Test | Test | Training | Training | Training | Training | Training |
| 208 | CHEMBL3359124 | 1 | Training | Training | Training | Validation | Training | Test | Training | Test | Validation | Training |
| 209 | CHEMBL3354064 | 1 | Training | Training | Validation | Training | Training | Training | Training | Training | Training | Training |
| 210 | CHEMBL3354061 | 1 | Validation | Training | Training | Validation | Training | Validation | Training | Test | Training | Validation |
| 211 | CHEMBL3359677 | 1 | Validation | Training | Test | Training | Validation | Training | Training | Training | Training | Validation |
| 212 | CHEMBL3359122 | 1 | Training | Validation | Training | Validation | Test | Test | Training | Test | Training | Training |
| 213 | CHEMBL3916914 | 1 | Test | Training | Training | Validation | Training | Training | Training | Training | Training | Test |
| 214 | CHEMBL3354042 | 1 | Training | Training | Training | Training | Test | Test | Training | Validation | Test | Training |
| 215 | CHEMBL1933098 | 1 | Training | Training | Test | Training | Training | Training | Training | Training | Training | Training |
| 216 | CHEMBL3354627 | 1 | Validation | Training | Training | Training | Training | Training | Validation | Test | Training | Training |
| 217 | CHEMBL2011641 | 1 | Training | Test | Training | Training | Test | Training | Training | Training | Training | Test |
| 218 | CHEMBL3354050 | 1 | Training | Test | Training | Training | Training | Training | Validation | Training | Training | Training |
| 219 | CHEMBL116366 | 1 | Validation | Training | Training | Training | Training | Training | Training | Training | Training | Training |
| 220 | CHEMBL3359672 | 1 | Training | Training | Training | Test | Validation | Training | Training | Training | Training | Training |
| 221 | CHEMBL110830 | 1 | Test | Training | Training | Validation | Training | Training | Training | Training | Training | Training |
| 222 | CHEMBL3359678 | 1 | Validation | Test | Test | Training | Training | Training | Validation | Training | Training | Training |
| 223 | CHEMBL3354629 | 1 | Test | Test | Training | Training | Training | Training | Validation | Training | Training | Validation |
| 224 | CHEMBL469435 | 1 | Training | Training | Validation | Training | Training | Training | Test | Training | Training | Training |
| 225 | CHEMBL3354025 | 1 | Training | Training | Training | Training | Training | Training | Training | Test | Training | Training |
| 226 | CHEMBL311226 | 1 | Training | Validation | Validation | Validation | Training | Training | Training | Training | Test | Training |
| 227 | CHEMBL3354053 | 1 | Training | Training | Training | Training | Training | Training | Training | Training | Training | Training |
| 228 | CHEMBL3354048 | 1 | Training | Training | Training | Training | Training | Training | Training | Training | Validation | Training |
| 229 | CHEMBL364554 | 1 | Training | Training | Training | Training | Training | Training | Training | Training | Validation | Validation |
| 230 | CHEMBL3354062 | 1 | Training | Training | Training | Training | Training | Training | Training | Validation | Training | Validation |
| 231 | CHEMBL574645 | 1 | Training | Training | Training | Training | Validation | Training | Test | Training | Test | Validation |
| 232 | CHEMBL2011632 | 1 | Test | Validation | Test | Training | Training | Test | Training | Validation | Training | Validation |
| 233 | CHEMBL3354045 | 1 | Training | Training | Training | Training | Test | Training | Training | Test | Training | Training |
| 234 | CHEMBL3354032 | 1 | Training | Training | Training | Test | Training | Training | Training | Test | Training | Training |
| 235 | CHEMBL1088158 | 1 | Training | Training | Training | Training | Training | Training | Training | Training | Training | Training |
| 236 | CHEMBL3133387 | 1 | Training | Test | Test | Training | Training | Training | Validation | Training | Test | Training |
| 237 | CHEMBL3944457 | 1 | Training | Training | Test | Validation | Test | Validation | Training | Test | Validation | Training |
| 238 | CHEMBL425308 | 1 | Training | Training | Training | Test | Training | Training | Training | Training | Training | Test |
| 239 | CHEMBL485840 | 1 | Training | Training | Validation | Validation | Test | Training | Test | Training | Validation | Test |
| 240 | CHEMBL3354057 | 1 | Training | Training | Training | Training | Training | Validation | Test | Training | Test | Training |
| 241 | CHEMBL1086996 | 1 | Training | Training | Validation | Validation | Validation | Training | Training | Training | Training | Training |
| 242 | CHEMBL4059852 | 1 | Training | Test | Training | Test | Training | Test | Validation | Training | Training | Validation |
| 243 | CHEMBL3354059 | 1 | Training | Training | Training | Training | Training | Validation | Test | Training | Training | Validation |
| 244 | CHEMBL498968 | 1 | Training | Validation | Training | Test | Training | Training | Training | Training | Test | Training |
| 245 | CHEMBL470868 | 1 | Training | Training | Training | Training | Test | Training | Training | Training | Training | Training |
| 246 | CHEMBL2011628 | 1 | Training | Validation | Training | Training | Test | Training | Training | Training | Training | Training |
| 247 | CHEMBL2164231 | 1 | Training | Training | Validation | Training | Training | Training | Training | Training | Training | Test |
| 248 | CHEMBL3354625 | 1 | Test | Training | Test | Training | Training | Validation | Validation | Validation | Training | Training |
| 249 | CHEMBL3354034 | 1 | Training | Training | Training | Test | Test | Training | Training | Training | Training | Validation |
| 250 | CHEMBL1922580 | 1 | Training | Test | Training | Training | Training | Training | Test | Training | Test | Training |
| 251 | CHEMBL1076754 | 1 | Training | Training | Training | Training | Training | Training | Training | Validation | Training | Training |
| 252 | CHEMBL511581 | 1 | Training | Training | Training | Training | Training | Test | Training | Training | Training | Test |
| 253 | CHEMBL3354023 | 1 | Training | Training | Training | Training | Training | Training | Test | Training | Training | Training |
| 254 | CHEMBL3901083 | 1 | Training | Training | Training | Training | Training | Training | Training | Training | Test | Training |
| 255 | CHEMBL3354016 | 1 | Training | Training | Training | Training | Training | Training | Training | Training | Training | Training |
| 256 | CHEMBL2204850 | 1 | Training | Validation | Training | Training | Training | Training | Validation | Training | Training | Training |
| 257 | CHEMBL3354623 | 1 | Training | Test | Test | Training | Training | Training | Training | Validation | Training | Training |
| 258 | CHEMBL2011603 | 1 | Validation | Training | Training | Test | Validation | Test | Training | Training | Training | Training |
| 259 | CHEMBL450985 | 1 | Training | Training | Training | Training | Validation | Training | Training | Training | Training | Training |
| 260 | CHEMBL2011634 | 1 | Training | Validation | Training | Training | Test | Validation | Training | Validation | Training | Test |
| 261 | CHEMBL186291 | 1 | Training | Validation | Training | Training | Training | Validation | Training | Training | Training | Training |
| 262 | CHEMBL3354632 | 1 | Validation | Test | Test | Training | Training | Training | Validation | Validation | Test | Training |
| 263 | CHEMBL3354051 | 1 | Training | Training | Test | Training | Validation | Test | Training | Training | Training | Training |
| 264 | CHEMBL1818436 | 1 | Training | Training | Training | Training | Training | Training | Test | Training | Training | Training |
| 265 | CHEMBL3354631 | 1 | Training | Training | Training | Validation | Training | Training | Training | Test | Test | Training |
| 266 | CHEMBL3354060 | 1 | Test | Training | Training | Training | Training | Training | Test | Training | Training | Training |
| 267 | CHEMBL3354031 | 1 | Training | Training | Training | Training | Validation | Test | Training | Training | Training | Test |
| 268 | CHEMBL3354637 | 1 | Training | Training | Test | Training | Training | Validation | Training | Training | Training | Training |
| 269 | CHEMBL3359669 | 1 | Training | Training | Training | Validation | Training | Training | Training | Training | Training | Validation |
| 270 | CHEMBL2170199 | 1 | Validation | Test | Training | Validation | Training | Validation | Training | Validation | Test | Training |
| 271 | CHEMBL3354624 | 1 | Validation | Training | Training | Validation | Training | Validation | Training | Training | Training | Training |
| 272 | CHEMBL3354628 | 1 | Training | Validation | Training | Validation | Training | Validation | Test | Training | Test | Training |
| 273 | CHEMBL3354621 | 1 | Test | Training | Training | Validation | Training | Training | Training | Validation | Validation | Validation |
| 274 | CHEMBL3133388 | 1 | Test | Validation | Training | Training | Test | Training | Test | Training | Training | Training |
| 275 | CHEMBL2205523 | 1 | Training | Training | Validation | Training | Training | Test | Training | Training | Training | Training |
| 276 | CHEMBL3901171 | 1 | Training | Training | Training | Training | Training | Validation | Test | Validation | Test | Training |
| 277 | CHEMBL3359666 | 1 | Test | Training | Training | Validation | Training | Training | Training | Training | Training | Training |
| 278 | CHEMBL3354046 | 1 | Training | Training | Training | Training | Validation | Validation | Training | Training | Training | Test |
| 279 | CHEMBL3354622 | 1 | Test | Training | Test | Test | Training | Training | Training | Validation | Training | Training |
| 280 | CHEMBL3354063 | 1 | Training | Validation | Training | Validation | Training | Training | Training | Training | Training | Training |
| 281 | CHEMBL3354033 | 1 | Training | Training | Training | Training | Training | Training | Training | Training | Test | Training |
| 282 | CHEMBL3354636 | 1 | Training | Training | Training | Training | Training | Training | Training | Training | Validation | Training |
| 283 | CHEMBL3354040 | 1 | Test | Training | Training | Training | Training | Training | Training | Test | Training | Training |
| 284 | CHEMBL469427 | 1 | Training | Validation | Training | Training | Training | Test | Training | Training | Training | Training |
| 285 | CHEMBL3359681 | 1 | Validation | Test | Training | Training | Test | Training | Training | Training | Training | Training |
| 286 | CHEMBL2011611 | 1 | Training | Training | Training | Training | Training | Training | Training | Training | Training | Training |
| 287 | CHEMBL469437 | 1 | Training | Training | Training | Training | Validation | Training | Training | Validation | Training | Training |
| 288 | CHEMBL3354039 | 1 | Training | Training | Training | Training | Training | Training | Training | Training | Training | Training |
| 289 | CHEMBL1029 | 1 | Training | Training | Validation | Validation | Training | Validation | Training | Test | Training | Training |
| 290 | CHEMBL3354056 | 1 | Training | Training | Training | Training | Training | Training | Test | Training | Training | Training |
| 291 | CHEMBL3354037 | 1 | Training | Training | Training | Validation | Training | Validation | Validation | Training | Training | Training |
| 292 | CHEMBL3359667 | 1 | Validation | Training | Training | Training | Training | Training | Training | Training | Training | Validation |
| 293 | CHEMBL2011626 | 1 | Test | Training | Training | Validation | Validation | Training | Training | Training | Training | Training |
| 294 | CHEMBL3354633 | 1 | Training | Validation | Training | Training | Training | Training | Training | Training | Training | Training |
| 295 | CHEMBL3354054 | 1 | Test | Training | Test | Training | Training | Training | Training | Training | Training | Training |
| 296 | CHEMBL3354638 | 1 | Validation | Training | Training | Training | Training | Training | Test | Training | Training | Training |
| 297 | CHEMBL1818433 | 1 | Test | Training | Training | Training | Test | Training | Training | Training | Training | Training |
| 298 | CHEMBL3354058 | 1 | Validation | Training | Training | Validation | Training | Training | Training | Training | Validation | Test |
| 299 | CHEMBL3133391 | 1 | Training | Test | Training | Training | Training | Training | Training | Training | Test | Training |
| 300 | CHEMBL3354015 | 1 | Training | Validation | Validation | Training | Training | Training | Validation | Validation | Training | Training |
| 301 | CHEMBL3935498 | 1 | Training | Validation | Training | Test | Training | Training | Training | Training | Validation | Training |
| 302 | CHEMBL2029773 | 1 | Validation | Training | Training | Training | Test | Training | Test | Training | Training | Test |
| 303 | CHEMBL3359123 | 1 | Training | Training | Training | Training | Training | Training | Validation | Training | Validation | Training |
| 304 | CHEMBL2114289 | 1 | Training | Test | Training | Training | Training | Training | Test | Training | Test | Training |
| 305 | CHEMBL3354041 | 1 | Training | Training | Validation | Training | Validation | Validation | Training | Test | Training | Test |
| 306 | CHEMBL3354027 | 1 | Training | Training | Training | Training | Training | Test | Test | Training | Training | Training |
| 307 | CHEMBL3354030 | 1 | Validation | Test | Test | Validation | Training | Validation | Training | Training | Training | Training |
| 308 | CHEMBL3907555 | 1 | Validation | Training | Training | Test | Training | Training | Training | Test | Test | Training |
| 309 | CHEMBL3354047 | 1 | Training | Training | Training | Training | Training | Training | Training | Training | Training | Training |
| 310 | CHEMBL3359680 | 1 | Training | Validation | Validation | Training | Training | Training | Training | Training | Training | Test |
| 311 | CHEMBL3354017 | 1 | Test | Test | Training | Training | Validation | Training | Validation | Training | Training | Training |
| 312 | CHEMBL3359671 | 1 | Training | Training | Training | Training | Validation | Training | Training | Training | Training | Test |
| 313 | CHEMBL470660 | 1 | Training | Training | Validation | Validation | Training | Training | Training | Training | Training | Training |
| 314 | CHEMBL3354026 | 1 | Training | Validation | Training | Training | Test | Training | Training | Training | Training | Training |
| 315 | CHEMBL323939 | 1 | Test | Training | Training | Training | Training | Training | Training | Training | Training | Training |
| 316 | CHEMBL3359674 | 1 | Training | Training | Validation | Validation | Training | Validation | Training | Training | Training | Training |
| 317 | CHEMBL2011612 | 1 | Test | Training | Training | Training | Test | Training | Training | Training | Training | Test |
| 318 | CHEMBL3359673 | 1 | Training | Training | Training | Training | Training | Training | Validation | Training | Validation | Training |
| 319 | CHEMBL3939205 | 1 | Training | Validation | Training | Training | Training | Training | Training | Training | Training | Validation |
| 320 | CHEMBL3354052 | 1 | Training | Validation | Training | Test | Training | Training | Test | Training | Training | Training |
| 321 | CHEMBL291020 | 1 | Training | Validation | Training | Validation | Training | Training | Training | Test | Training | Training |
| 322 | CHEMBL3354020 | 1 | Training | Validation | Training | Training | Training | Training | Training | Training | Training | Validation |
| 323 | CHEMBL3354019 | 1 | Training | Training | Training | Training | Test | Validation | Test | Training | Training | Validation |
| 324 | CHEMBL1086997 | 1 | Training | Test | Training | Test | Training | Training | Training | Training | Test | Test |

# **Table S3.** The results of prediction performed for BIOFACQUIM database (<https://biofacquim.herokuapp.com/>) using ten selected ANN classifiers employing maxHBint3 and SpMax8_Bhs descriptors. The models were generated using different dataset splits into training validation and test set (supplementary Table S2).

| **InChIKey** | **SMILES** | **maxHBint3** | **SpMax8_Bhs** | **Class (predicted)** | | | | | | | | | | | | |
| --- | --- | --- | --- | --- | --- | --- | --- | --- | --- | --- | --- | --- | --- | --- | --- | --- |
|  |  |  |  | **Split1** (**RBF 2-24-2)** | **Split1 (RBF 2-22-2)** | **Split2 (RBF 2-27-2)** | **Split3 (MLP 2-4-2)** | **Split4 (RBF 2-24-2)** | **Split5 (MLP 2-4-2)** | **Split5 (RBF 2-22-2)** | **Split6 (MLP 2-5-2)** | **Split7 (MLP 2-4-2)** | **Split8 (RBF 2-27-2)** | **Split9 (RBF 2-22-2)** | **Split9 (RBF 2-28-2)** | **Split10 (RBF 2-30-2)** |
| NPJICTMALKLTFW-OFUAXYCQSA-N | O([C@H]1[C@H](O)[C@@H](O)[C@H](O)[C@@H](CO)O1)[C@@H]1CC=2[C@@](C)([C@@H]3[C@H]([C@H]4[C@@](C)([C@@H]([C@@H](CC[C@H](C(C)C)CC)C)CC4)CC3)CC=2)CC1 | 3.7903266 | 3.6483876 | 1 | 1 | 1 | 1 | 1 | 1 | 1 | 1 | 1 | 1 | 1 | 1 | 1 |
| PEWFWDOPJISUOK-UHFFFAOYSA-N | O(CC(O)C(O)(C)C)c1c2c(occ2)cc2OC(=O)C=Cc12 | 4.1900093 | 3.0023078 | 1 | 1 | 1 | 1 | 1 | 1 | 1 | 1 | 1 | 1 | 1 | 1 | 1 |
| CETCIHSCOIXIKA-ACIDHAGOSA-N | O(C)[C@H]1OC(=O)C(CC[C@@]2(C)[C@H](C)CC[C@]34C(C(=O)OC3)=C[C@@H](O[C@H]3[C@H](O)[C@@H](O)[C@H](O)[C@@H](CO)O3)CC24)=C1 | 3.9713325 | 3.8514536 | 1 | 1 | 1 | 1 | 1 | 1 | 1 | 1 | 1 | 1 | 1 | 1 | 1 |
| ZVLAQNYZLIHKAE-JLXBEBSSSA-N | O([C@H]1[C@H](O)[C@@H](O)[C@H](O)[C@@H](CO)O1)[C@@H]1C=C2C(=O)OC[C@]32C([C@](CCC=2C(=O)OCC=2)(C)[C@H](C)CC3)C1 | 3.9960755 | 3.7468074 | 1 | 1 | 1 | 1 | 1 | 1 | 1 | 1 | 1 | 1 | 1 | 1 | 1 |
| JMHINZPNTWTGKC-ZREMQXPESA-N | O([C@H]1[C@H](O)[C@@H](O)[C@H](O)[C@@H](CO)O1)[C@@H]1C=C2C(=O)OC[C@]32C([C@](CCC=2C(OC)OC(=O)C=2)(C)[C@H](C)CC3)C1 | 3.9655117 | 3.8534047 | 1 | 1 | 1 | 1 | 1 | 1 | 1 | 1 | 1 | 1 | 1 | 1 | 1 |
| ROSSVNHEVRUXGM-HEMPLKHUSA-N | O([C@H]1[C@H](O)[C@@H](O)[C@H](O)[C@@H](CO)O1)[C@@H]1C=C2C(=O)OC[C@]32C([C@](CCc2cocc2)(C)[C@H](C)CC3)C1 | 3.9963014 | 3.673624 | 1 | 1 | 1 | 1 | 1 | 1 | 1 | 1 | 1 | 1 | 1 | 1 | 1 |
| OOYXHYNAJPWYQC-XRGUOFIRSA-N | O=C(CC[C@@]1(C)[C@H](C)CC[C@]23C(C(=O)OC2)=C[C@@H](O[C@H]2[C@H](O)[C@@H](O)[C@H](O)[C@@H](CO)O2)CC13)C | 4.0446445 | 3.7086575 | 1 | 1 | 1 | 1 | 1 | 1 | 1 | 1 | 1 | 1 | 1 | 1 | 1 |
| MENXOAAQJIIUMB-OUWQEXSBSA-N | O=C1[C@]2(C)[C@H]3[C@@](O1)(Cc1c(O)c(O)c(C(C)C)c(O)c1C(=O)C3)CCC2 | 3.9362063 | 3.3024744 | 1 | 1 | 1 | 1 | 1 | 1 | 1 | 1 | 1 | 1 | 1 | 1 | 1 |
| MYXNWGACZJSMBT-VJXVFPJBSA-N | O=C1c2c(O)c([C@H]3[C@H](O)[C@@H](O)[C@H](O)[C@@H](CO)O3)c(O)cc2OC(c2ccc(O)cc2)=C1 | 4.3564731 | 4.104588 | 1 | 1 | 1 | 1 | 1 | 1 | 0 | 1 | 1 | 1 | 1 | 1 | 1 |
| SGEWCQFRYRRZDC-VPRICQMDSA-N | O=C1c2c(O)cc(O)c([C@H]3[C@H](O)[C@@H](O)[C@H](O)[C@@H](CO)O3)c2OC(c2ccc(O)cc2)=C1 | 4.2759883 | 4.1048288 | 1 | 1 | 1 | 1 | 1 | 1 | 0 | 1 | 1 | 1 | 1 | 1 | 1 |
| PUPKKEQDLNREIM-QNSQPKOQSA-N | O([C@H]1[C@H](O)[C@H](O)[C@@H](O)[C@H](C)O1)c1cc(O)c2C(=O)C(O[C@H]3[C@H](O)[C@H](O)[C@@H](O)[C@H](C)O3)=C(c3ccc(O)cc3)Oc2c1 | 4.3636117 | 4.1725301 | 1 | 1 | 1 | 1 | 1 | 1 | 0 | 1 | 1 | 1 | 1 | 1 | 1 |
| KMOUJOKENFFTPU-QNDFHXLGSA-N | O([C@H]1[C@H](O)[C@@H](O)[C@H](O)[C@@H](CO)O1)c1cc(O)c2C(=O)C=C(c3ccc(O)cc3)Oc2c1 | 4.5445839 | 3.8529669 | 1 | 1 | 1 | 1 | 1 | 1 | 1 | 1 | 0 | 1 | 1 | 1 | 1 |
| TXVLBRMNUYNGCT-KMEZTADASA-N | O=C(O)[C@@H](NC(=O)[C@@H](O)Cc1ccccc1)C[C@@]1(C(C=C)(C)C)C(=O)Nc2c(O)cccc12 | 4.6951843 | 3.7198145 | 1 | 1 | 1 | 1 | 1 | 1 | 1 | 1 | 0 | 1 | 1 | 0 | 1 |
| QZMIWNADYPPZTF-RLSLOFABSA-N | O=C(O)[C@@H](NC(=O)[C@@H](O)Cc1ccccc1)C[C@@]1(C(C=C)(C)C)C(=O)Nc2c1cccc2 | 4.5936635 | 3.657916 | 1 | 1 | 1 | 1 | 1 | 1 | 1 | 1 | 0 | 1 | 1 | 0 | 1 |
| HGLOWJDVJNISEN-NVXKJEIXSA-N | O([C@@H](C(O)(C)C)C[C@H](C(C)c1cc(O)c(C)cc1)C)[C@H]1[C@H](O[C@@H]2[C@@H](O)[C@H](O)[C@H](O)CO2)[C@@H](O)[C@H](O)[C@@H](CO)O1 | 4.5774047 | 4.2931942 | 1 | 1 | 1 | 1 | 1 | 1 | 0 | 1 | 0 | 1 | 1 | 1 | 0 |
| BEEAOSMHUSDOTP-NJYVYQBISA-N | O(C)c1c2[C@@H]3[C@@](C)([C@H](O)C(=O)C=C3OC)C=C3C(OC)=CC(=O)c(c(O)c1)c23 | 3.5014083 | 3.3296162 | 0 | 1 | 1 | 1 | 1 | 1 | 1 | 1 | 1 | 1 | 0 | 1 | 0 |
| MWKQZQOBWYIJHX-HSQYWUDLSA-N | O=C(OC[C@@]1(C)[C@H]2[C@@](C)(c3c(O)c(O)c(C(C)C)cc3C(=O)C2)CCC1)C | 3.4727061 | 3.1884696 | 0 | 1 | 1 | 1 | 1 | 1 | 1 | 1 | 1 | 1 | 0 | 1 | 0 |
| OLPZITFOWYCLMF-VUAAKQTLSA-N | O([C@@H](CCCC)[C@H]1OC(=O)C=C(OC)C1)[C@H]1[C@@H](O)[C@@H](OC)[C@H](OC)[C@@H](CO)O1 | 2.8740567 | 3.4228696 | 1 | 0 | 1 | 1 | 0 | 1 | 1 | 1 | 1 | 1 | 0 | 0 | 0 |
| HGUQYROCEMPGDU-BWIMDNAMSA-N | O=C(c1cc(O)c(O)cc1)C1=C(O)[C@@](C/C=C(/CC/C=C(\C)/C)\C)(C/C=C(\C)/C)C(=O)C=2[C@H](C3=C(O)C(=O)C=CO3)CC(C)(C)OC1=2 | 5.0032721 | 3.7438947 | 1 | 1 | 0 | 1 | 0 | 1 | 0 | 1 | 0 | 1 | 1 | 0 | 1 |
| HGUQYROCEMPGDU-YJMRDKADSA-N | O=C(c1cc(O)c(O)cc1)C1=C(O)[C@](C/C=C(/CC/C=C(\C)/C)\C)(C/C=C(\C)/C)C(=O)C=2[C@H](C3=C(O)C(=O)C=CO3)CC(C)(C)OC1=2 | 5.0032721 | 3.7438947 | 1 | 1 | 0 | 1 | 0 | 1 | 0 | 1 | 0 | 1 | 1 | 0 | 1 |
| GUNXKIKUNNXJKN-NKLJSFOLSA-N | O([C@@H]1[C@H](O)[C@H](O)[C@@H](CO)O1)c1c(O)c2c(c(O)c1)C(=O)O[C@H](C)C2 | 4.764957 | 3.608092 | 1 | 1 | 0 | 1 | 0 | 1 | 1 | 1 | 0 | 1 | 1 | 0 | 0 |
| COWWBPRRBQOULA-NABHEWRGSA-N | O=C(O[C@@H]1[C@@H](OC(=O)C)[C@](O)(C)C(=O)/C=C\[C@H](C)C[C@H]2OC(=O)C(=C)[C@H]12)C(=C)C | 2.8872924 | 3.472496 | 1 | 0 | 1 | 1 | 0 | 1 | 1 | 1 | 1 | 1 | 0 | 0 | 0 |
| YXBNQTGKGCNTPV-VCEHAEOKSA-N | O([C@H]([C@H](OC(=O)C[C@@H](C(=O)O)CC(=O)O)[C@@H](CCCC)C)[C@H](C[C@H](O)CCCC[C@@H](O)C[C@H](O)[C@@H](N)C)C)C(=O)C[C@@H](C(=O)O)CC(=O)O | 3.9444891 | 4.5294797 | 0 | 0 | 1 | 0 | 1 | 1 | 0 | 0 | 1 | 1 | 1 | 1 | 0 |
| MXKKFADFYXJREN-TXZJYACMSA-N | O[C@H]1[C@@]2(c3ccc(O)cc3)Oc3c(c(O)cc(O)c3)[C@@H]1c1c3O[C@@H]([C@@H](O)Cc3c(O)cc1O2)c1cc(O)c(O)cc1 | 5.3370494 | 4.0628552 | 1 | 1 | 0 | 1 | 0 | 1 | 0 | 1 | 0 | 1 | 1 | 0 | 0 |
| PRRUXSDCUPUYKC-OPMOSEIQSA-N | O=C(O)CCCCCCCCCC(O[C@H]1[C@H](O[C@H]2[C@H](O[C@H]3[C@H](O)[C@H](O)[C@@H](O[C@H]4[C@H](O)[C@@H](O)[C@H](O)[C@@H](C)O4)[C@H](C)O3)[C@@H](O)[C@H](C)[C@@H](CO)C2)[C@@H](O)[C@H](O)[C@@H](C)O1)CCCCC | 3.8990962 | 4.5601131 | 0 | 0 | 1 | 0 | 1 | 1 | 0 | 0 | 1 | 1 | 1 | 1 | 0 |
| DXPFQBFCOCKNEU-RMCHFIGYSA-N | O=C(O[C@@H]1[C@@H](OC(=O)C)[C@](O)(C)C(=O)/C=C\[C@H](C)C[C@H]2OC(=O)C(=C)[C@H]12)/C(=C/C)/C | 2.744074 | 3.4856932 | 1 | 0 | 1 | 1 | 0 | 1 | 1 | 1 | 1 | 0 | 0 | 0 | 0 |
| DXPFQBFCOCKNEU-MBWLHKRJSA-N | O=C(O[C@@H]1[C@@H](OC(=O)C)[C@](O)(C)C(=O)/C=C\[C@H](C)C[C@H]2OC(=O)C(=C)[C@H]12)/C(=C\C)/C | 2.744074 | 3.4856932 | 1 | 0 | 1 | 1 | 0 | 1 | 1 | 1 | 1 | 0 | 0 | 0 | 0 |
| KNCNSYQYJUBLPW-NRIIMPDMSA-N | O(C[C@@H]1[C@@H](O)[C@H](O)[C@@H](O)[C@H](Oc2c3C(c4cc(O)c(O)cc4)=CC(=O)Oc3cc(OC)c2)O1)[C@H]1[C@H](O)[C@@](O)(CO)CO1 | 5.5039059 | 4.1915842 | 1 | 1 | 0 | 1 | 0 | 1 | 0 | 1 | 1 | 1 | 0 | 0 | 0 |
| BDIGJZNTLJAZGG-YVGDPVPUSA-N | O([C@H](C(=O)[C@@H]1[C@@H]([C@H](O)[C@@H]2OC(=O)C=CC2)C1)C)C(=O)/C=C/c1cc(O)c(OC)cc1 | 2.7088141 | 3.4368901 | 1 | 0 | 1 | 1 | 0 | 1 | 1 | 1 | 1 | 0 | 0 | 0 | 0 |
| VEBNYMXKXIIGFX-IYVYCCGLSA-N | O=C(O[C@H]1C(O)[C@H](OC(=O)/C=C/c2cc(O)c(O)cc2)CC(O)(C(=O)OC)C1)/C=C/c1cc(O)c(O)cc1 | 5.7403871 | 4.0162625 | 1 | 1 | 0 | 1 | 0 | 1 | 0 | 1 | 1 | 1 | 0 | 0 | 0 |
| XSUATNGSAZMWRU-ZYAFLGLPSA-N | O(C[C@@H]1[C@@H](O)[C@H](O)[C@@H](O)[C@H](Oc2c3C(c4cc(O)c(O)cc4)=CC(=O)Oc3cc(O)c2)O1)[C@H]1[C@H](O)[C@@H](O)[C@H](O)CO1 | 5.4985719 | 4.2520596 | 1 | 1 | 0 | 1 | 0 | 1 | 0 | 1 | 1 | 1 | 0 | 0 | 0 |
| GXDJGKMWLJOJFR-WUSKNVGPSA-N | O(C[C@@H]1[C@@H](O)[C@H](O)[C@@H](O)[C@H](Oc2c3C(c4cc(O)c(O)cc4)=CC(=O)Oc3cc(OC)c2)O1)[C@H]1[C@H](O)[C@@H](O)[C@H](O)CO1 | 5.4607117 | 4.1982119 | 1 | 1 | 0 | 1 | 0 | 1 | 0 | 1 | 1 | 1 | 0 | 0 | 0 |
| DDGYXSWHWAQNRQ-MGPUTAFESA-N | O(C)c1c2occc2cc2C(=O)[C@]3(O)[C@@H](Oc12)COc1c3cc2OCOc2c1 | 3.0278088 | 3.1004699 | 0 | 0 | 1 | 1 | 0 | 1 | 1 | 1 | 1 | 1 | 0 | 0 | 0 |
| OVSQVDMCBVZWGM-DTGCRPNFSA-N | O([C@H]1[C@H](O)[C@@H](O)[C@@H](O)[C@@H](CO)O1)C1=C(c2cc(O)c(O)cc2)Oc2c(c(O)cc(O)c2)C1=O | 5.6584515 | 4.1659039 | 1 | 1 | 0 | 1 | 0 | 1 | 0 | 1 | 1 | 1 | 0 | 0 | 0 |
| OVSQVDMCBVZWGM-QSOFNFLRSA-N | O([C@H]1[C@H](O)[C@@H](O)[C@H](O)[C@@H](CO)O1)C1=C(c2cc(O)c(O)cc2)Oc2c(c(O)cc(O)c2)C1=O | 5.6584515 | 4.1659039 | 1 | 1 | 0 | 1 | 0 | 1 | 0 | 1 | 1 | 1 | 0 | 0 | 0 |
| OXGUCUVFOIWWQJ-HQBVPOQASA-N | O([C@H]1[C@H](O)[C@H](O)[C@@H](O)[C@H](C)O1)C1=C(c2cc(O)c(O)cc2)Oc2c(c(O)cc(O)c2)C1=O | 5.6560832 | 4.0941001 | 1 | 1 | 0 | 1 | 0 | 1 | 0 | 1 | 1 | 1 | 0 | 0 | 0 |
| PCWHVEGCZALKKT-LPLXGARPSA-N | O=C(OC[C@@H]1[C@H](O)[C@H](O)[C@@H](O)[C@H](OC2=C(c3cc(O)c(O)cc3)Oc3c(c(O)cc(O)c3)C2=O)O1)c1ccccc1 | 5.5446613 | 4.0517946 | 1 | 1 | 0 | 1 | 0 | 1 | 0 | 1 | 1 | 1 | 0 | 0 | 0 |
| KALBJZVIHLVACU-YNUABKSXSA-N | O=C(O)CCCCCCCCC[C@@H](O[C@H]1[C@H](O[C@@H]2[C@@H](O[C@@H]3[C@@H](O)[C@@H](O)[C@H](O[C@H]4[C@H](O)[C@@H](O)[C@@H](O)[C@@H](C)O4)[C@@H](C)O3)[C@H](O)[C@@H](O)[C@H](CO)O2)[C@@H](O)[C@H](O)[C@@H](C)O1)CCCC | 4.000093 | 4.5965153 | 0 | 0 | 1 | 0 | 1 | 1 | 0 | 0 | 1 | 1 | 1 | 1 | 0 |
| TYWCXSPTQQXINE-ROMNNNSQSA-N | O=C(O)CCCCCCCCC[C@@H](O[C@H]1[C@H](O[C@@H]2[C@@H](O[C@@H]3[C@@H](O)[C@@H](O)[C@H](O[C@H]4[C@H](O)[C@@H](O)[C@@H](O)[C@@H](C)O4)[C@@H](C)O3)[C@H](O)[C@@H](O)[C@H](CO)O2)[C@@H](O)[C@H](O)[C@@H](C)O1)CCCCCC | 3.9700293 | 4.5923334 | 0 | 0 | 1 | 0 | 1 | 1 | 0 | 0 | 1 | 1 | 1 | 1 | 0 |
| KOQREMYYHAAAAT-HBRPDFDGSA-N | O=C(O)CCCCCCCCC[C@@H](O[C@H]1[C@H](O[C@H]2[C@H](O[C@H]3[C@H](O)[C@H](O)[C@@H](O)[C@H](C)O3)[C@@H](O)[C@H](O)[C@@H](CO[C@H]3[C@H](O)[C@@H](O)[C@H](O)[C@@H](C)O3)O2)[C@@H](O)[C@H](O)[C@@H](C)O1)CCC | 3.9643494 | 4.5980296 | 0 | 0 | 1 | 0 | 1 | 1 | 0 | 0 | 1 | 1 | 1 | 1 | 0 |
| SHUQHAMYYBXIIQ-GCSSGBNASA-N | O=C(O)CCCCCCCCC[C@@H](O[C@H]1[C@H](O[C@H]2[C@H](O[C@H]3[C@H](O)[C@H](O)[C@@H](O)[C@H](C)O3)[C@@H](O[C@H]3[C@H](O)[C@@H](O)[C@H](O)[C@@H](C)O3)[C@H](O)[C@@H](C)O2)[C@@H](O)[C@H](O)[C@@H](C)O1)CCC | 3.6795757 | 4.5649097 | 0 | 0 | 1 | 0 | 1 | 1 | 0 | 0 | 1 | 1 | 1 | 1 | 0 |
| HNCIHRVMBZWZSI-XSFVSMFZSA-N | O=C(c1cc(O)c(O)cc1)C=1C(=O)C(C/C=C(/CC/C=C(\C)/C)\C)(C/C=C(\C)/C)C(=O)C23OC2C(O)C(C)(C)OC=13 | 5.1596105 | 3.6977392 | 1 | 1 | 0 | 1 | 0 | 1 | 0 | 1 | 0 | 1 | 1 | 0 | 0 |
| KEQANZVCLOIYPL-KGENOOAVSA-N | O=C(c1cc(O)c(O)cc1)C=1C(=O)C(C/C=C(/CC/C=C(\C)/C)\C)(C/C=C(\C)/C)C(=O)C23OC2CC(C)(C)OC=13 | 5.1587588 | 3.6282038 | 1 | 1 | 0 | 1 | 0 | 1 | 0 | 1 | 0 | 1 | 1 | 0 | 0 |
| LLSDPOXYUGFOKC-UHFFFAOYSA-N | O(C)c1c(Oc2c(OC)cc(C=3Oc4c(c(O)c(O)c(OC)c4)C(=O)C=3)cc2)ccc(C=2Oc3c(c(O)c(O)c(OC)c3)C(=O)C=2)c1 | 5.3602905 | 3.8760504 | 1 | 1 | 0 | 1 | 0 | 1 | 0 | 1 | 0 | 1 | 1 | 0 | 0 |
| MRFHAFKKJKWCOY-KPHUOKFYSA-N | O=C1[C@]2(C)[C@@H]3[C@@H](O1)C=C1C(=O)C(C(C)C)=C(O)C(=O)C1=CC3=CCC2 | 5.2641958 | 3.2821237 | 1 | 1 | 0 | 1 | 0 | 1 | 1 | 1 | 0 | 1 | 0 | 0 | 0 |
| UJZKICMAGUFXSG-NTCOEUGSSA-N | O=C1[C@]2(C)[C@@H]3[C@H]([C@@H](O)C=4C(=O)C(C(C)C)=C(O)C(=O)C=4C=C3C=CC2)O1 | 5.2438436 | 3.2935225 | 1 | 1 | 0 | 1 | 0 | 1 | 1 | 1 | 0 | 1 | 0 | 0 | 0 |
| JTCUWDQRUWSZLA-PSCQKVGZSA-N | O=C1[C@]2(C)[C@@H]3[C@H]([C@@H](O)C=4C(=O)C(C(C)C)=C(O)C(=O)C=4C=C3[C@H](O)CC2)O1 | 5.1488827 | 3.4728533 | 1 | 1 | 0 | 1 | 0 | 1 | 1 | 1 | 0 | 1 | 0 | 0 | 0 |
| LIOHNCKMCBYMAR-RAJNIJHNSA-N | O=C1[C@]2(C)[C@H]3C([C@H](O)CC2)=CC=2C(=O)C(O)=C(C(C)C)C(=O)C=2C=C3O1 | 5.2764994 | 3.3202979 | 1 | 1 | 0 | 1 | 0 | 1 | 1 | 1 | 0 | 1 | 0 | 0 | 0 |
| DXPFQBFCOCKNEU-AUNCTZJHSA-N | O=C(O[C@@H]1[C@@H](OC(=O)C)[C@](O)(C)C(=O)/C=C\[C@H](C)C[C@H]2OC(=O)C(=C)[C@H]12)C(=CC)C | 2.744074 | 3.4856932 | 1 | 0 | 1 | 1 | 0 | 1 | 1 | 1 | 1 | 0 | 0 | 0 | 0 |
| OBNVTTHRWSUAMB-IAPIXIRKSA-N | OC(C(O)CC[C@@H](C)c1cc(O)c(C)cc1)(C)C | 3.8434112 | 2.7869589 | 1 | 0 | 0 | 0 | 0 | 0 | 1 | 1 | 1 | 1 | 0 | 0 | 1 |
| OFCHTGCBFRTISG-VUEDXXQZSA-N | O(C)c1c(O)ccc([C@H]2OC[C@@H]3[C@@H](c4c(C)c5OCOc5cc4)OC[C@H]23)c1 | 2.7675752 | 3.1063376 | 0 | 0 | 1 | 1 | 0 | 1 | 1 | 1 | 1 | 0 | 0 | 0 | 0 |
| KRZBCHWVBQOTNZ-RDJMKVHDSA-N | O=C(O[C@H]1C(O)[C@H](OC(=O)/C=C/c2cc(O)c(O)cc2)CC(O)(C(=O)O)C1)/C=C/c1cc(O)c(O)cc1 | 5.9190073 | 4.0344231 | 1 | 0 | 0 | 1 | 0 | 1 | 0 | 1 | 1 | 1 | 0 | 0 | 0 |
| GXIDPDIXZHCJHP-KMJCNKHZSA-N | O=C(O)CCCCCCCCC[C@@H](O[C@H]1[C@H](O[C@@H]2[C@@H](O[C@H]3[C@H](O)[C@H](O)[C@@H](O)[C@H](C)O3)[C@H](O)[C@@H](O)[C@H](CO[C@H]3[C@H](O[C@H]4[C@H](O)[C@H](O)[C@@H](O)[C@H](C)O4)[C@@H](O)[C@H](O)[C@@H](C)O3)O2)[C@@H](O)[C@H](O)[C@@H](C)O1)CCC | 3.8279567 | 4.6691361 | 0 | 0 | 1 | 0 | 1 | 1 | 0 | 0 | 1 | 1 | 0 | 1 | 0 |
| GLAAQZFBFGEBPS-UHFFFAOYSA-N | O(C)c1c(O)c2C(=O)C=C(c3cc(OC)c(O)cc3)Oc2cc1O | 2.9154703 | 3.2440023 | 0 | 0 | 1 | 1 | 0 | 1 | 1 | 1 | 1 | 0 | 0 | 0 | 0 |
| DRRWBCNQOKKKOL-UHFFFAOYSA-N | O(C)c1c(O)c2C(=O)C=C(c3cc(OC)c(OC)cc3)Oc2cc1O | 2.6953913 | 3.2188402 | 0 | 0 | 1 | 1 | 0 | 1 | 1 | 1 | 1 | 0 | 0 | 0 | 0 |
| HRQRRWLXKJXUIZ-RHMWYWNKSA-N | O(Oc1c2C(c3cc(O)c(O)cc3)=CC(=O)Oc2cc(O)c1)[C@H]1[C@H](O)[C@@H](O)[C@H](O)CO1 | 5.7049326 | 3.8641136 | 1 | 1 | 0 | 1 | 0 | 1 | 0 | 1 | 0 | 1 | 0 | 0 | 0 |
| BDIFYHLEBJTHAL-LWRIQDNHSA-N | O(Oc1c2C(c3cc(O)c(O)cc3)=CC(=O)Oc2cc(OC)c1)[C@@H]1[C@H](O)[C@@H](O)[C@@H](CO)CO1 | 5.6390884 | 3.6459913 | 1 | 1 | 0 | 1 | 0 | 1 | 0 | 1 | 0 | 1 | 0 | 0 | 0 |
| GBPGQCGEJYVXSH-ZQEFQCJFSA-N | O(Oc1c2C(c3cc(O)c(O)cc3)=CC(=O)Oc2cc(OC)c1)[C@H]1[C@H](O)[C@@H](O)[C@H](O)CO1 | 5.6699678 | 3.6361762 | 1 | 1 | 0 | 1 | 0 | 1 | 0 | 1 | 0 | 1 | 0 | 0 | 0 |
| KQFUXLQBMQGNRT-UHFFFAOYSA-N | O(C)c1c(O)ccc(C2=C(OC)C(=O)c3c(O)cc(OC)cc3O2)c1 | 2.8801674 | 3.2058188 | 0 | 0 | 1 | 1 | 0 | 1 | 1 | 1 | 1 | 0 | 0 | 0 | 0 |
| JABJSHMLHVMIMA-UMGXQCJCSA-N | O=C(c1cc(O)c(O)cc1)C=1C(=O)[C@@](C/C=C(/CC/C=C(\C)/C)\C)(C/C=C(\C)/C)C(=O)C=1O | 5.5104833 | 3.6066337 | 1 | 1 | 0 | 1 | 0 | 1 | 0 | 1 | 0 | 1 | 0 | 0 | 0 |
| CVULDJMCSSACEO-VMPREFPWSA-N | O=C(OC[C@@H](NC(=O)c1ccccc1)Cc1ccccc1)[C@@H](NC(=O)c1ccccc1)Cc1ccccc1 | 5.0480311 | 3.3509887 | 0 | 1 | 0 | 1 | 0 | 1 | 1 | 1 | 0 | 1 | 0 | 0 | 0 |
| DWZAJFZEYZIHPO-UHFFFAOYSA-N | O(C)c1c(O)c2C(=O)C(OC)=C(c3ccc(OC)cc3)Oc2cc1O | 2.6857472 | 3.2245559 | 0 | 0 | 1 | 1 | 0 | 1 | 1 | 1 | 1 | 0 | 0 | 0 | 0 |
| GRUVGBVSXJLAOU-FDQSAEMMSA-N | O=C(O[C@H]1C=2C(=O)C(C(C)C)=C(O)C(=O)C=2[C@]2(C)[C@H]([C@](CO)(C)CCC2)C1)C | 4.3194456 | 3.3983911 | 0 | 0 | 1 | 1 | 1 | 1 | 0 | 1 | 1 | 0 | 0 | 0 | 0 |
| UYYYJFGICXAUGO-HAGHYFMRSA-N | O[C@@H]1[C@@]2(O[C@H]3[C@](O)(c4c(c(C)ccc4)O3)CC2)c2c(O1)cc(C)cc2 | 2.292104 | 2.994007 | 0 | 0 | 1 | 1 | 0 | 1 | 1 | 1 | 0 | 0 | 0 | 0 | 0 |
| OWQLBLNRUZULFV-UHFFFAOYSA-N | O(C)c1c(O)cc(O)c2C(=O)C(OC)=C(c3ccccc3)Oc12 | 2.8197639 | 2.9140849 | 0 | 0 | 1 | 1 | 0 | 1 | 1 | 1 | 0 | 0 | 0 | 0 | 0 |
| ZKXIUVREPAJEBX-AYVHDSGLSA-N | O=C(O)CCCCCCCCC[C@@H](O[C@H]1[C@H](O[C@]2(O)[C@@H](O)[C@H](O[C@H]3[C@H](O[C@H]4[C@H](O)[C@H](O)[C@@H](O)[C@H](C)O4)[C@H](O[C@H]4[C@H](O)[C@H](O)[C@@H](O)[C@H](C)O4)[C@H](O)[C@@H](C)O3)[C@@H](C)CO2)[C@@H](O)[C@H](O)[C@@H](C)O1)CCC | 3.6084651 | 4.6365227 | 0 | 0 | 1 | 0 | 1 | 1 | 0 | 0 | 1 | 1 | 0 | 0 | 0 |
| IPMAKCYEUXFJTI-UHFFFAOYSA-N | O(C)c1c(O)c(OC)cc2c1-c1c(c(OC)c(O)cc1)CC2 | 2.7154375 | 2.9158993 | 0 | 0 | 1 | 1 | 0 | 1 | 1 | 1 | 0 | 0 | 0 | 0 | 0 |
| LLRLPRUFUGWYOL-UHFFFAOYSA-N | O(C)c1c(O)c(OC)cc2c1c1c(c(OC)c(O)cc1)cc2 | 2.7787525 | 2.9281619 | 0 | 0 | 1 | 1 | 0 | 1 | 1 | 1 | 0 | 0 | 0 | 0 | 0 |
| MBNGWHIJMBWFHU-UHFFFAOYSA-N | O(C)c1c(O)cc(C=2Oc3c(c(O)cc(O)c3)C(=O)C=2)cc1 | 2.74924 | 2.9933127 | 0 | 0 | 1 | 1 | 0 | 1 | 1 | 1 | 0 | 0 | 0 | 0 | 0 |
| WJKXLOGUCSSDAB-JGVFFNPUSA-N | O(C)C=1[C@H](O)[C@@](O)(C)CC(=O)C=1 | 4.9580372 | 1.9295527 | 0 | 0 | 1 | 0 | 1 | 0 | 0 | 0 | 0 | 1 | 1 | 0 | 1 |
| XUZGFXBHALEMTN-BIIVOSGPSA-N | Cl[C@@H]1[C@](O)(C)[C@@H](O)C(OC)=CC1=O | 4.7161082 | 1.9437728 | 0 | 1 | 1 | 0 | 1 | 0 | 0 | 0 | 0 | 0 | 1 | 0 | 1 |
| HUCKMCHVBAWQIA-DGBHNKMOSA-N | O=C(O[C@@H]1[C@@H](O[C@H]2[C@H](O)[C@H](O)[C@@H](O)[C@H](C)O2)[C@H](OC[C@@H]2[C@@H](O)[C@H]3[C@@H](O[C@H]4[C@H](OC(=O)/C(=C/C)/C)[C@H](O)[C@@H](O)[C@H](C)O4)[C@@H](O2)O[C@@H]2[C@@H](O)[C@H](O)[C@@H](C)O[C@H]2O[C@@H](CCCCC)CCCCCCCCCC(=O)O3)O[C@H](C)[C@H]1O)/C(=C/C)/C | 3.3988562 | 4.5457118 | 0 | 0 | 1 | 0 | 1 | 1 | 0 | 0 | 1 | 1 | 0 | 0 | 0 |
| QMGPOYYFOFDFEO-JPYSVIHJSA-N | O([C@@H](C)[C@H]1O[C@@H]([C@H](O)[C@@H]2OC(=O)C=CC2)CC1)C(=O)/C=C/c1cc(O)c(O)cc1 | 5.7904448 | 3.5287192 | 1 | 0 | 0 | 1 | 0 | 1 | 0 | 1 | 0 | 1 | 0 | 0 | 0 |
| NLJQJJGYCFVOFQ-GCOVDLRRSA-N | O=C(OC)[C@@H](NC(=O)[C@@H](O)Cc1ccccc1)C[C@@]1(C(C=C)(C)C)C(=O)Nc2c(O)cccc12 | 4.6687358 | 3.5604555 | 1 | 0 | 0 | 1 | 0 | 1 | 1 | 1 | 0 | 0 | 0 | 0 | 0 |
| VMKCIRAJEVFSFR-LQTXRJQHSA-N | O=C1[C@H](Cc2ccccc2)OC(=O)[C@H]2N1[C@]1(C(C=C)(C)C)[C@@](O)(c3c(c(O)ccc3)N1)C2 | 2.429806 | 3.2973842 | 0 | 0 | 1 | 1 | 0 | 1 | 1 | 1 | 0 | 0 | 0 | 0 | 0 |
| HHYNJJSTUPNMQU-RUGJYQHNSA-N | O=C(O[C@H]1C=2C(=O)C(C(C)C)=C(O)C(=O)C=2C[C@]23OC(=O)[C@](C)([C@@H]2C1)CCC3)C | 4.7583113 | 3.4422997 | 0 | 0 | 0 | 1 | 0 | 1 | 1 | 1 | 0 | 1 | 0 | 0 | 0 |
| XXVSZSNOEWVDPA-KPHUOKFYSA-N | O=C1[C@]2(C)[C@@H]3[C@@H](O1)CC=1C(=O)C(C(C)C)=C(O)C(=O)C=1CC3=CCC2 | 5.0694989 | 3.2810153 | 0 | 1 | 0 | 1 | 0 | 1 | 1 | 1 | 0 | 0 | 0 | 0 | 0 |
| NFLLWTNAXDSKKC-MRFFXTKBSA-N | O=C1[C@]2(C)[C@H]3[C@@](O1)(CC=1C(=O)C(O)=C(C(C)C)C(=O)C=1C=C3)CCC2 | 5.0627859 | 3.1729319 | 0 | 1 | 0 | 1 | 0 | 1 | 1 | 1 | 0 | 0 | 0 | 0 | 0 |
| KJWFOHVSTFGWGZ-YCDQCDSPSA-N | O=C(O[C@@H]1[C@H](O)c2c3OC(=O)C=Cc3ccc2OC1(C)C)/C(=C\C)/C | 1.6041667 | 2.9528447 | 0 | 0 | 1 | 1 | 0 | 1 | 0 | 1 | 0 | 0 | 0 | 0 | 0 |
| QPLSCFLMIOADPA-RQJZHBNNSA-N | O=C(O[C@H]1[C@@H](O)C(C)(C)Oc2c1c1OC(=O)C=Cc1cc2)/C(=C\C)/C | 1.7889826 | 3.0964895 | 0 | 0 | 1 | 1 | 0 | 1 | 0 | 1 | 0 | 0 | 0 | 0 | 0 |
| JHCPPFWOQPOFRF-HZPDHXFCSA-N | O=C(O[C@H]1[C@@H](O)C(C)(C)Oc2c1c1OC(=O)C=Cc1cc2)C(C)C | 1.8063349 | 3.1128955 | 0 | 0 | 1 | 1 | 0 | 1 | 0 | 1 | 0 | 0 | 0 | 0 | 0 |
| GIVSZLKTIBWYRM-UHFFFAOYSA-N | O(C)c1c(OC)c2c3c(c(OC)cc2cc1O)cc(O)cc3 | 2.6437906 | 2.8351501 | 0 | 0 | 1 | 1 | 0 | 0 | 1 | 1 | 0 | 0 | 0 | 0 | 0 |
| YTRAYUIKLRABOQ-UHFFFAOYSA-N | O(C)c1c(O)c(OC)cc(CCc2cc(OC)c(O)cc2)c1 | 2.9016353 | 2.8335147 | 0 | 0 | 1 | 1 | 0 | 0 | 1 | 1 | 0 | 0 | 0 | 0 | 0 |
| MRYYPHKLLLULDL-UHFFFAOYSA-N | O(C)c1c(OC)c2c3c(O)cccc3c(OC)cc2cc1O | 2.6356783 | 2.7649583 | 0 | 0 | 1 | 1 | 0 | 0 | 1 | 1 | 0 | 0 | 0 | 0 | 0 |
| LJJIBKCHKKEYAS-OIDCBOCKSA-N | O=C(O)CCCCCCCCCC(O[C@H]1[C@H](O[C@H]2[C@H](O)[C@H](O)[C@@H](O[C@H]3[C@H](O)[C@H](O[C@H]4[C@H](O)[C@@H](O)[C@H](O)[C@@H](CO)O4)[C@@H](O[C@H]4[C@H](O)[C@H](O)[C@@H](O)[C@H](C)O4)[C@H](C)O3)[C@H](C)O2)[C@@H](O)[C@H](O)[C@@H](CO)O1)CCCCC | 3.7128111 | 4.7213372 | 0 | 0 | 0 | 0 | 1 | 1 | 0 | 0 | 1 | 1 | 0 | 0 | 0 |
| LTAIPRWVZLMEEJ-WOFCNMKLSA-N | O=C(O)CCCCCCCCCC(O[C@H]1[C@H](O[C@H]2[C@H](O[C@H]3[C@H](O)[C@H](C[C@H]4[C@H](O[C@H]5[C@H](O)[C@@H](O)[C@H](O)[C@@H](C)O5)[C@@H](O)[C@H](O)[C@@H](CO)O4)[C@@H](O[C@H]4[C@H](O)[C@@H](O)[C@@H](O)[C@@H](C)O4)[C@H](C)O3)[C@@H](O)[C@H](O)[C@@H](CO)O2)[C@@H](O)[C@H](O)[C@@H](C)O1)CCC | 3.4798881 | 4.7261104 | 0 | 0 | 0 | 0 | 1 | 1 | 0 | 0 | 1 | 1 | 0 | 0 | 0 |
| XVZMUMHZDANJSG-VTKNXRICSA-N | O=C(O)CCCCCCCCCC(O[C@H]1[C@H](O[C@H]2[C@H](O[C@H]3[C@H](O)[C@H](C[C@H]4[C@H](O[C@H]5[C@H](O)[C@@H](O)[C@H](O)[C@@H](C)O5)[C@@H](O)[C@H](O)[C@@H](CO)O4)[C@@H](O[C@H]4[C@H](O)[C@@H](O)[C@@H](O)[C@@H](C)O4)[C@H](C)O3)[C@@H](O)[C@H](O)[C@@H](CO)O2)[C@@H](O)[C@H](O)[C@@H](C)O1)CCCCC | 3.4498856 | 4.7215459 | 0 | 0 | 0 | 0 | 1 | 1 | 0 | 0 | 1 | 1 | 0 | 0 | 0 |
| KHEBRRJCDZMMMT-VUEVMRPESA-N | O=C(OC)CCCCCCCCCC(O[C@H]1[C@H](O[C@H]2[C@H](O[C@H]3[C@H](O)[C@H](C[C@H]4[C@H](O[C@H]5[C@H](O)[C@@H](O)[C@H](O)[C@@H](C)O5)[C@@H](O)[C@H](O)[C@@H](CO)O4)[C@@H](O[C@H]4[C@H](O)[C@@H](O)[C@@H](O)[C@@H](C)O4)[C@H](C)O3)[C@@H](O)[C@H](O)[C@@H](CO)O2)[C@@H](O)[C@H](O)[C@@H](C)O1)CCC | 3.4718093 | 4.7002339 | 0 | 0 | 0 | 0 | 1 | 1 | 0 | 0 | 1 | 1 | 0 | 0 | 0 |
| XEDWBBOLVGMERH-NCOBWLNYSA-N | O=C(OC)CCCCCCCCCC(O[C@H]1[C@H](O[C@H]2[C@H](O[C@H]3[C@H](O)[C@H](C[C@H]4[C@H](O[C@H]5[C@H](O)[C@@H](O)[C@H](O)[C@@H](C)O5)[C@@H](O)[C@H](O)[C@@H](CO)O4)[C@@H](O[C@H]4[C@H](O)[C@@H](O)[C@@H](O)[C@@H](C)O4)[C@H](C)O3)[C@@H](O)[C@H](O)[C@@H](CO)O2)[C@@H](O)[C@H](O)[C@@H](C)O1)CCCCC | 3.4417675 | 4.6967751 | 0 | 0 | 0 | 0 | 1 | 1 | 0 | 0 | 1 | 1 | 0 | 0 | 0 |
| UTXKXSRRVOYQSF-UHFFFAOYSA-N | O(C)c1c(OC)cc(CCc2cc(OC)c(O)cc2)cc1O | 2.9032787 | 2.8329359 | 0 | 0 | 1 | 1 | 0 | 0 | 1 | 1 | 0 | 0 | 0 | 0 | 0 |
| WZUVPPKBWHMQCE-XJKSGUPXSA-N | Oc1c(O)ccc2[C@@H]3[C@](O)(COc12)Cc1c3cc(O)c(O)c1 | 5.6278909 | 3.2055271 | 1 | 0 | 0 | 1 | 0 | 1 | 0 | 0 | 0 | 1 | 0 | 0 | 0 |
| QFYNDCRFXVUINR-XGICHPGQSA-N | O=C(OCC(O)(COC(=O)C)c1c(O)cc(C)cc1)/C(=C\C)/C | 1.5388517 | 2.9972852 | 0 | 0 | 1 | 1 | 0 | 1 | 0 | 1 | 0 | 0 | 0 | 0 | 0 |
| CQXZARCGOSILEP-WCIBSUBMSA-N | O=C(Oc1c(C2(CO)OC2)ccc(C)c1)/C(=C\C)/C | 2.8868246 | 2.810013 | 0 | 0 | 1 | 1 | 0 | 0 | 1 | 1 | 0 | 0 | 0 | 0 | 0 |
| CWVRJTMFETXNAD-JUHZACGLSA-N | O=C(O[C@H]1[C@H](O)[C@H](O)C[C@@](O)(C(=O)O)C1)/C=C/c1cc(O)c(O)cc1 | 6.2630461 | 3.983565 | 0 | 0 | 0 | 1 | 0 | 1 | 0 | 1 | 0 | 1 | 0 | 0 | 0 |
| CUCFSOKADUSMPF-ZANVPECISA-N | O(C)c1c(C)c2[C@](O)(C)[C@H](CO)OC(=O)c2c(O)c1 | 2.8027767 | 2.853764 | 0 | 0 | 1 | 1 | 0 | 0 | 1 | 1 | 0 | 0 | 0 | 0 | 0 |
| HDTRYLNUVZCQOY-LIZSDCNHSA-N | O([C@@H]1[C@H](O)[C@@H](O)[C@H](O)[C@@H](CO)O1)[C@@H]1[C@H](O)[C@@H](O)[C@H](O)[C@@H](CO)O1 | 4.6768456 | 4.8552447 | 0 | 1 | 0 | 1 | 0 | 1 | 0 | 0 | 0 | 1 | 0 | 0 | 0 |
| OPIUYBQHWXRHKP-HNKATWAKSA-N | O=C(O)CCCCCCCCC[C@H](O[C@H]1[C@H](O[C@H]2[C@H](O[C@H]3[C@H](O)[C@H](O[C@@H]4[C@@H](O[C@@H]5[C@@H](O)[C@H](O)[C@@H](O)[C@H](C)O5)[C@H](O)[C@@H](O)[C@H](CO)O4)[C@@H](O[C@H]4[C@H](O)[C@@H](O)[C@@H](O)[C@@H](C)O4)[C@H](C)O3)[C@@H](O)[C@H](O)[C@@H](CO)O2)[C@@H](O)[C@H](O)[C@@H](C)O1)CCCC | 3.5846609 | 4.7261105 | 0 | 0 | 0 | 0 | 1 | 1 | 0 | 0 | 1 | 1 | 0 | 0 | 0 |
| LSULNWMZUOBWRA-NIYMFQSSSA-N | O=C(O)CCCCCCCCC[C@H](O[C@H]1[C@H](O[C@H]2[C@H](O[C@H]3[C@H](O)[C@H](O[C@@H]4[C@@H](O[C@@H]5[C@@H](O)[C@H](O)[C@@H](O)[C@H](C)O5)[C@H](O)[C@@H](O)[C@H](CO)O4)[C@@H](O[C@H]4[C@H](O)[C@@H](O)[C@@H](O)[C@@H](C)O4)[C@H](C)O3)[C@@H](O)[C@H](O)[C@@H](CO)O2)[C@@H](O)[C@H](O)[C@@H](C)O1)CCCCCC | 3.5571838 | 4.7215459 | 0 | 0 | 0 | 0 | 1 | 1 | 0 | 0 | 1 | 1 | 0 | 0 | 0 |
| OXOOTMOEABRPHG-BBYXOMEOSA-N | O=C(O)C[C@@H](CCCCCCC[C@H](O[C@H]1[C@H](O[C@H]2[C@H](O[C@H]3[C@H](O)[C@H](O[C@@H]4[C@@H](O[C@@H]5[C@@H](O)[C@H](O)[C@@H](O)[C@H](C)O5)[C@H](O)[C@@H](O)[C@H](CO)O4)[C@@H](O[C@H]4[C@H](O)[C@@H](O)[C@@H](O)[C@@H](C)O4)[C@H](C)O3)[C@@H](O)[C@H](O)[C@@H](CO)O2)[C@@H](O)[C@H](O)[C@@H](C)O1)CCCCCC)[C@@H]1[C@H](O)[C@@H](O)[C@H](O)[C@@H](C)O1 | 3.5014139 | 4.7439431 | 0 | 0 | 0 | 0 | 1 | 1 | 0 | 0 | 1 | 1 | 0 | 0 | 0 |
| GOZCEKPKECLKNO-RKQHYHRCSA-N | O=C(C)c1ccc(O[C@H]2[C@H](O)[C@@H](O)[C@H](O)[C@@H](CO)O2)cc1 | 4.7187918 | 3.0179641 | 0 | 1 | 0 | 1 | 0 | 1 | 1 | 0 | 0 | 0 | 0 | 0 | 0 |
| DEEHWRGXKJRSMV-WMOYMDCYSA-N | O([C@@H](C)[C@H]1O[C@@H]([C@H](O)[C@@H]2OC(=O)C=CC2)CC1)C(=O)/C=C/c1ccc(O)cc1 | 2.0260775 | 3.2652193 | 0 | 0 | 1 | 1 | 0 | 1 | 0 | 1 | 0 | 0 | 0 | 0 | 0 |
| PRXVDDVCKQCKQP-PPCLKGCXSA-N | O([C@@H](C)[C@H]1O[C@@H]([C@H](O)[C@@H]2OC(=O)C=CC2)CC1)C(=O)/C=C/c1ccc(OC)cc1 | 2.0121785 | 3.2437656 | 0 | 0 | 1 | 1 | 0 | 1 | 0 | 1 | 0 | 0 | 0 | 0 | 0 |
| DEEHWRGXKJRSMV-SJFPRCBQSA-N | O([C@@H](C)[C@H]1O[C@@H]([C@H](O)[C@@H]2OC(=O)C=CC2)CC1)C(=O)/C=C\c1ccc(O)cc1 | 2.0260775 | 3.2652193 | 0 | 0 | 1 | 1 | 0 | 1 | 0 | 1 | 0 | 0 | 0 | 0 | 0 |
| PRXVDDVCKQCKQP-IZONMWRVSA-N | O([C@@H](C)[C@H]1O[C@@H]([C@H](O)[C@@H]2OC(=O)C=CC2)CC1)C(=O)/C=C\c1ccc(OC)cc1 | 2.0121785 | 3.2437656 | 0 | 0 | 1 | 1 | 0 | 1 | 0 | 1 | 0 | 0 | 0 | 0 | 0 |
| PHWNDHXZDCXMCU-AWEZNQCLSA-N | O(C)c1c2C(=O)OC[C@]3(O)C(OC)=CC(=O)c(c(O)c1)c23 | 1.7346355 | 3.0485766 | 0 | 0 | 1 | 1 | 0 | 1 | 0 | 1 | 0 | 0 | 0 | 0 | 0 |
| QIEMGQKOGFTYLN-UHFFFAOYSA-N | O(C)c1c(OC)ccc(C=2Oc3c(c(O)c(O)c(OC)c3)C(=O)C=2)c1 | 5.6062571 | 3.1890035 | 1 | 0 | 0 | 1 | 0 | 1 | 0 | 0 | 0 | 1 | 0 | 0 | 0 |
| PNCQKGVAPWZYRN-BGERDNNASA-N | O=C(O)[C@@H](NC(=O)C(O)Cc1ccccc1)Cc1c(C(C=C)(C)C)[nH]c2c1cccc2 | 4.6746709 | 3.3451022 | 0 | 0 | 0 | 1 | 0 | 1 | 1 | 1 | 0 | 0 | 0 | 0 | 0 |
| JLUYABBECFTSKK-AGEKVHLVSA-N | O=C(O[C@@]12[C@@](C(C=C)(C)C)(N3C(=O)[C@H](Cc4ccccc4)OC(=O)[C@@H]3C1)Nc1c(O)cccc21)C | 2.1130493 | 3.3686879 | 0 | 0 | 1 | 1 | 0 | 1 | 0 | 1 | 0 | 0 | 0 | 0 | 0 |
| GBMUTTKBDFEONI-OTLGMJNYSA-N | O=C1[C@H](Cc2ccccc2)OC(=O)[C@H]2N1[C@]1(C(C=C)(C)C)[C@@](O)(c3c(N1)cccc3)C2 | 2.0480482 | 3.1779348 | 0 | 0 | 1 | 1 | 0 | 1 | 0 | 1 | 0 | 0 | 0 | 0 | 0 |
| HWCRIILCNXONSY-SYMKQFHVSA-N | O=C1C(C(C)C)=C(O)C(=O)C2=C1[C@H](O)C[C@H]1[C@@]3(C)CO[C@@H](O)[C@@]21CCC3 | 4.5565909 | 3.3195719 | 0 | 0 | 0 | 1 | 0 | 1 | 1 | 1 | 0 | 0 | 0 | 0 | 0 |
| SCDGKJFOGSRPQB-JGZYSILTSA-N | O([C@H]1[C@H](O)[C@@H](O)[C@H](O)[C@@H](CO)O1)c1c2OC(=O)C=C(OC)c2c(C)cc1 | 4.5974708 | 3.4668603 | 0 | 0 | 0 | 1 | 0 | 1 | 1 | 1 | 0 | 0 | 0 | 0 | 0 |
| IZQSVPBOUDKVDZ-UHFFFAOYSA-N | O(C)c1c(O)ccc(C2=C(O)C(=O)c3c(O)cc(O)cc3O2)c1 | 6.3439052 | 3.2680766 | 0 | 0 | 0 | 1 | 0 | 1 | 0 | 0 | 0 | 1 | 0 | 0 | 0 |
| REFJWTPEDVJJIY-UHFFFAOYSA-N | O=C1C(O)=C(c2cc(O)c(O)cc2)Oc2c1c(O)cc(O)c2 | 6.481414 | 3.4002899 | 0 | 0 | 0 | 1 | 0 | 1 | 0 | 0 | 0 | 1 | 0 | 0 | 0 |
| BJDMHAYLPGRUFH-HIELJHRRSA-N | O([C@H](C(=O)[C@@H]1[C@@H]([C@H](O)[C@@H]2OC(=O)C=CC2)C1)C)C(=O)/C=C/c1ccc(O)cc1 | 1.7317013 | 3.4234648 | 0 | 0 | 1 | 0 | 0 | 1 | 0 | 1 | 0 | 0 | 0 | 0 | 0 |
| XGNLXIVLBLLTIG-FMRMVBGOSA-N | O([C@H](C(=O)[C@@H]1[C@@H]([C@H](O)[C@@H]2OC(=O)C=CC2)C1)C)C(=O)/C=C/c1ccc(OC)cc1 | 1.7168647 | 3.4119511 | 0 | 0 | 1 | 0 | 0 | 1 | 0 | 1 | 0 | 0 | 0 | 0 | 0 |
| UIWQVTWVFQWDQI-IQOMITAASA-N | O([C@H](C(=O)[C@@H]1[C@@H]([C@H](O)[C@@H]2OC(=O)C=CC2)C1)C)C(=O)/C=C\c1cc(O)ccc1 | 1.7313179 | 3.4235972 | 0 | 0 | 1 | 0 | 0 | 1 | 0 | 1 | 0 | 0 | 0 | 0 | 0 |
| CJJVWWCXCQUGER-UAZACNSMSA-N | O=C(O[C@H]1c2c(c(O)cc(C)c2)C(O)=C2C(=O)[C@@H]3c4c5O[C@@]6(C(=O)OC)[C@H](O)C=CC(O)=C6C(=O)c5c(O)cc4C[C@]12C=C3)C | 1.7193557 | 4.1925824 | 0 | 0 | 0 | 0 | 0 | 1 | 0 | 0 | 1 | 1 | 0 | 0 | 0 |
| YKQYIQHWWYVPHK-CPFSXVBKSA-N | O(C)c1c(C)c2[C@@]([C@@H](O)C)(C)OC(=O)c2c(O)c1 | 2.2781082 | 2.8158915 | 0 | 0 | 1 | 1 | 0 | 0 | 0 | 1 | 0 | 0 | 0 | 0 | 0 |
| MFJMOCPZLBNSPY-ZDUSSCGKSA-N | O(C)c1c(CO)c2[C@](O)(C)C(=C)OC(=O)c2c(O)c1 | 1.768894 | 2.800376 | 0 | 0 | 1 | 1 | 0 | 0 | 0 | 1 | 0 | 0 | 0 | 0 | 0 |
| QHWDTDHKZFTJKU-PYOFXGKSSA-N | O=C(O[C@@H]1[C@@H](O)[C@H](O[C@@H]2[C@@H](O)[C@H](O)[C@@H](CO)O[C@H]2O[C@H]2[C@@H](OC(=O)[C@H](CC)C)[C@H](O[C@@H]3[C@@H]4[C@H](O)[C@@H](COC(=O)C)O[C@H]3O[C@@H]3[C@@H](O)[C@H](O)[C@@H](C)O[C@H]3O[C@@H](CCC)CCCCCCC[C@H](O[C@@H]3[C@@H](O)[C@H](O)[C@@H](O)[C@H](C)O3)CC(=O)O4)O[C@@H](C)[C@@H]2O[C@H]2[C@H](O)[C@@H](OC(=O)CCCCCCCCCCC)[C@@H](O)[C@@H](C)O2)O[C@H](C)[C@H]1O)[C@H](CC)C | 3.008058 | 4.6980549 | 0 | 0 | 0 | 0 | 0 | 1 | 0 | 0 | 1 | 1 | 0 | 0 | 0 |
| NKXXQFOOOHJLRQ-DYFOXTGPSA-N | O=C(O[C@@H]1[C@@H](OC(=O)C(CC)C)[C@H](C)O[C@@H](O[C@@H]2[C@@H](O[C@H]3[C@H](O)[C@H](O)[C@@H](O)[C@H](C)C3)[C@@H](O)[C@H](O[C@@H]3[C@@H](O)[C@@H](O)[C@H](O[C@@H]4[C@@H](O)[C@@H](O)[C@@H](C)O[C@H]4O[C@H](CCCCCCCCCC(=O)O[C@@H]4[C@H](O)[C@@H](O)[C@H](C)O[C@H]4O[C@H]4[C@@H](OC(=O)CCCCCCCCCCC)[C@H](O[C@H]5[C@H](C)O[C@H]6O[C@@H]7[C@@H](O)[C@@H](O)[C@@H](C)O[C@H]7O[C@@H](CCCCC)CCCCCCCCCC(=O)O[C@@H]6[C@@H]5O)O[C@@H](C)[C@@H]4O[C@H]4[C@H](O)[C@H](O)[C@H](OC(=O)C(CC)C)[C@H](C)O4)CCCCC)O[C@H]3C)O[C@H]2C)[C@@H]1O)/C=C/c1ccccc1 | 2.283096 | 4.6435376 | 0 | 0 | 0 | 0 | 0 | 1 | 0 | 0 | 1 | 1 | 0 | 0 | 0 |
| YFMMGUARCLTHRY-CJYYLSOKSA-N | O=C(O[C@H]1[C@@H](O)[C@@H](O)[C@H](O[C@@H]2[C@@H](O[C@H]3[C@H](O)[C@H](O)[C@@H](OC(=O)CCCCCCCCC[C@@H](O[C@H]4[C@H](O[C@H]5[C@H](O)[C@H](O)[C@@H](O[C@H]6[C@H](OC(=O)CCCCCCCCC)[C@H](O[C@H]7[C@H](O)[C@H](O)[C@@H](O)[C@H](C)C7)[C@@H](O[C@H]7[C@H](O)[C@H](O)[C@@H](OC(=O)[C@@H](CC)C)[C@H](C)O7)[C@H](C)O6)[C@H](C)O5)[C@@H](O)[C@@H](O)[C@@H](C)O4)CCCCC)[C@H](C)O3)[C@@H](OC(=O)CCCCCCCCC)[C@H](O[C@H]3[C@H](C)O[C@@H]4[C@H](O)[C@@H]3OC(=O)CCCCCCCCC[C@H](CCCCC)O[C@@H]3O[C@H](C)[C@H](O)[C@H](O)[C@H]3O4)O[C@H]2C)O[C@H]1C)[C@@H](CC)C | 2.045433 | 4.6954726 | 0 | 0 | 0 | 0 | 0 | 1 | 0 | 0 | 1 | 1 | 0 | 0 | 0 |
| HKLDKRBGASBJRS-SYGNNDERSA-N | O=C(O[C@H]1[C@@H](O)[C@@H](O)[C@H](O[C@@H]2[C@@H](O[C@H]3[C@H](O)[C@H](O)[C@@H](OC(=O)CCCCCCCCC[C@@H](O[C@H]4[C@H](O[C@H]5[C@H](O)[C@H](O)[C@@H](O[C@H]6[C@H](OC(=O)CCCCCCCCCCC)[C@H](O[C@H]7[C@H](O)[C@H](O)[C@@H](O)[C@H](C)C7)[C@@H](O[C@H]7[C@H](O)[C@H](O)[C@@H](OC(=O)[C@@H](CC)C)[C@H](C)O7)[C@H](C)O6)[C@H](C)O5)[C@@H](O)[C@@H](O)[C@@H](C)O4)CCCCC)[C@H](C)O3)[C@@H](OC(=O)CCCCCCCCCCC)[C@H](O[C@H]3[C@H](C)O[C@@H]4[C@H](O)[C@@H]3OC(=O)CCCCCCCCC[C@H](CCCCC)O[C@@H]3O[C@H](C)[C@H](O)[C@H](O)[C@H]3O4)O[C@H]2C)O[C@H]1C)[C@@H](CC)C | 1.9877383 | 4.6960086 | 0 | 0 | 0 | 0 | 0 | 1 | 0 | 0 | 1 | 1 | 0 | 0 | 0 |
| DTEIVMDGEUSUOI-IKIOFCMYSA-N | O=C(O[C@H]1[C@@H](O)[C@@H](O)[C@H](O[C@@H]2[C@@H](O[C@H]3[C@H](OC(=O)CCCCCCCCC[C@@H](O[C@H]4[C@H](O[C@H]5[C@H](O)[C@H](O)[C@@H](O[C@H]6[C@H](OC(=O)CCCCCCCCCCC)[C@H](O[C@H]7[C@H](O)[C@H](O)[C@@H](O)[C@H](C)C7)[C@@H](O[C@H]7[C@H](O)[C@H](O)[C@@H](OC(=O)C(C)C)[C@H](C)O7)[C@H](C)O6)[C@H](C)O5)[C@@H](O)[C@@H](O)[C@@H](C)O4)CCCCC)[C@H](O)[C@@H](O)[C@H](C)O3)[C@@H](OC(=O)CCCCCCCCCCC)[C@H](O[C@H]3[C@H](C)O[C@H]4O[C@@H]5[C@@H](O)[C@@H](O)[C@@H](C)O[C@H]5O[C@@H](CCCCC)CCCCCCCCCC(=O)O[C@@H]4[C@@H]3O)O[C@H]2C)O[C@H]1C)C(C)C | 1.9983086 | 4.6957449 | 0 | 0 | 0 | 0 | 0 | 1 | 0 | 0 | 1 | 1 | 0 | 0 | 0 |
| CHWPMFMUQATVNK-IUCIJCHOSA-N | O[C@@H]1[C@]2(C(=C)C)O[C@@H]2[C@]2(C)[C@@H](C)[C@H](O)CCC2=C1 | 1.9241552 | 2.7553226 | 0 | 0 | 1 | 1 | 0 | 0 | 0 | 1 | 0 | 0 | 0 | 0 | 0 |
| ABOXHMBRQLJXDS-QFONWEIGSA-N | O=C(O[C@@H]1[C@@H](C)O[C@@H]2[C@H](O[C@H]3[C@H](O)[C@H](O)[C@@H](O)[C@H](C)O3)[C@H]1O[C@H]1[C@H](O)[C@@H]([C@@H](O)[C@H](C)O1)OC(=O)CCCCCCCCC[C@H](CCC)O[C@@H]1O[C@H](C)[C@@H](O)[C@H](O)[C@H]1O[C@H]1[C@H](OC(=O)/C(=C/C)/C)[C@H](O)[C@H]([C@H](C)O1)O2)/C(=C/C)/C | 3.1373486 | 4.4748331 | 0 | 0 | 0 | 0 | 0 | 1 | 0 | 0 | 1 | 1 | 0 | 0 | 0 |
| AFFBKAFUWKADJM-RFDHAYLPSA-N | O=C(O[C@@H]1[C@H](O)[C@@H](O)[C@H](C)O[C@H]1O[C@@H]1[C@@H](O)[C@H](O)[C@@H]2O[C@H]1O[C@@H]1[C@@H](O)[C@H](O)[C@@H](C)O[C@H]1O[C@@H](CCC)CCCCCCCCCC(=O)O[C@@H]1[C@H](O)[C@@H](O)[C@H](C)O[C@H]1O[C@@H]1[C@@H](O)[C@H](O)[C@@H](C)O[C@H]1OC2)/C(=C/C)/C | 3.0199128 | 4.5040865 | 0 | 0 | 0 | 0 | 0 | 1 | 0 | 0 | 1 | 1 | 0 | 0 | 0 |
| GJTLOGFPCMLBGM-QZEDAVLZSA-N | O=C(O[C@@H]1[C@H](O)[C@@H](O)[C@H](C)O[C@H]1O[C@@H]1[C@@H]2[C@H](O)[C@@H](C)O[C@H]1O[C@@H]1[C@@H](O)[C@@H](OC(=O)/C(=C/C)/C)[C@@H](O[C@H]1C)O[C@@H]1[C@@H](O)[C@H](O)[C@@H](C)O[C@H]1O[C@@H](CCC)CCCCCCCCCC(=O)O[C@@H]1[C@@H](O)[C@H](C)O[C@H]([C@@H]1O)O2)[C@@H]([C@H](O)C)C | 2.6519106 | 4.5611632 | 0 | 0 | 0 | 0 | 0 | 1 | 0 | 0 | 1 | 1 | 0 | 0 | 0 |
| UIWUPBBHRVAQMV-ZVKYRITESA-N | O=C(O[C@@H]1[C@H](O)[C@@H](O[C@H]2[C@H](O)[C@@H](O)[C@@H](OC(=O)/C(=C/C)/C)[C@@H](C)O2)[C@H](C)O[C@H]1O[C@@H]1[C@@H]2[C@H](O)[C@@H](CO)O[C@H]1O[C@@H]1[C@@H](O)[C@H](O)[C@@H](C)O[C@H]1O[C@@H](CCCC)CCCCCCCCCC(=O)O2)/C(=C/C)/C | 2.4468485 | 4.4751044 | 0 | 0 | 0 | 0 | 0 | 1 | 0 | 0 | 1 | 1 | 0 | 0 | 0 |
| OHIGTDSMWCKQMO-YNTSURPRSA-N | O=C(O[C@@H]1[C@H](O)[C@@H](O[C@H]2[C@H](O)[C@@H](O)[C@@H](OC(=O)/C(=C/C)/C)[C@@H](C)O2)[C@H](C)O[C@H]1O[C@@H]1[C@@H]2[C@H](O)[C@@H](CO)O[C@H]1O[C@@H]1[C@@H](O)[C@H](O)[C@@H](C)O[C@H]1O[C@@H](CCCCCC)CCCCCCCCCC(=O)O2)/C(=C/C)/C | 2.4067223 | 4.4791573 | 0 | 0 | 0 | 0 | 0 | 1 | 0 | 0 | 1 | 1 | 0 | 0 | 0 |
| IZXAJJIHCIQBDV-QZAYOBNPSA-N | O=C(O[C@H]1[C@H](C)O[C@H]2O[C@@H]3[C@@H](O)[C@H](O)[C@@H](C)O[C@H]3OC[C@@H]3[C@@H](O)[C@H](O)[C@@H](O[C@H]4[C@H](O)[C@H](O)[C@@H](OC(=O)C)[C@H](C)O4)[C@H](O[C@@H]4[C@@H](O)[C@H](O)[C@@H](C)O[C@H]4O[C@@H](CCC)CCCCCCCCCC(=O)O[C@@H]2[C@@H]1O)O3)[C@@H]([C@H](O)C)C | 2.5931188 | 4.6297613 | 0 | 0 | 0 | 0 | 0 | 1 | 0 | 0 | 1 | 1 | 0 | 0 | 0 |
| OXPMXNOQWPDRHJ-GBOYLNFRSA-N | O=C(O[C@H]1[C@H](O)[C@@H](C)O[C@H]2OC[C@@H]3[C@@H](O)[C@H](O)[C@@H](O[C@H]4[C@H](O)[C@H](O)[C@@H](OC(=O)C)[C@H](C)O4)[C@H](O[C@H]4[C@H](O)[C@@H](O)[C@H](C)O[C@@H]4O[C@@H](CCC)CCCCCCCCCC(=O)O[C@H]12)O3)[C@H]([C@@H](O)C)C | 2.7240215 | 4.6082972 | 0 | 0 | 0 | 0 | 0 | 1 | 0 | 0 | 1 | 1 | 0 | 0 | 0 |
| PZVXILWUBLKOBQ-DBASVXGUSA-N | O=C(O[C@H]1[C@H](O)[C@@H](C)O[C@H]2O[C@H]3[C@H](O)[C@@H](C)O[C@H]([C@@H]3O[C@H]3[C@H](O)[C@H](O)[C@@H](OC(=O)C)[C@H](C)O3)O[C@@H]3[C@@H](O)[C@H](O)[C@@H](C)O[C@H]3O[C@@H](CCC)CCCCCCCCCC(=O)O[C@H]12)[C@H]([C@@H](O)C)C | 2.3923184 | 4.5835961 | 0 | 0 | 0 | 0 | 0 | 1 | 0 | 0 | 1 | 1 | 0 | 0 | 0 |
| QWUHUBDKQQPMQG-UHFFFAOYSA-N | O(C)c1c(O)c(O)c2C(=O)C=C(c3cc(O)c(O)cc3)Oc2c1 | 5.9069723 | 3.2792184 | 0 | 0 | 0 | 1 | 0 | 1 | 0 | 0 | 0 | 1 | 0 | 0 | 0 |
| JKVQMJIPYGVPFF-UHFFFAOYSA-N | O=C(C)c1c(O)c2c(OC(CO)(C)C=C2)cc1 | 3.1714091 | 2.655101 | 0 | 0 | 0 | 0 | 0 | 0 | 1 | 1 | 0 | 1 | 0 | 0 | 0 |
| QVYSZKIZAPTGSX-UHFFFAOYSA-N | O(C)c1c(O)c2C(=O)C(O)=C(c3cc(OC)c(OC)cc3)Oc2cc1OC | 6.0522154 | 3.2549143 | 0 | 0 | 0 | 1 | 0 | 1 | 0 | 0 | 0 | 1 | 0 | 0 | 0 |
| PFTAWBLQPZVEMU-UKRRQHHQSA-N | O[C@H]1[C@@H](c2cc(O)c(O)cc2)Oc2c(c(O)cc(O)c2)C1 | 5.7851629 | 2.9499514 | 0 | 0 | 0 | 0 | 0 | 1 | 0 | 0 | 0 | 1 | 0 | 0 | 0 |
| KQJGPGHQDDZVHJ-ZFOCBLLKSA-N | O=C1[C@H](O)CC/C=C/[C@H](O)[C@H](O)[C@@H](CCC)O1 | 5.8073019 | 2.9142607 | 0 | 0 | 0 | 0 | 0 | 1 | 0 | 0 | 0 | 1 | 0 | 0 | 0 |
| SOHTUOALNFQEMJ-UHFFFAOYSA-N | O(C)c1c(O)c(OC)cc2c1-c1c(cc(O)cc1)CC2 | 2.5820919 | 2.6426718 | 0 | 0 | 0 | 0 | 0 | 0 | 1 | 1 | 0 | 0 | 0 | 0 | 0 |
| MMBACJOZVSORQJ-KTJIKPCYSA-N | O[C@@H]1[C@@]2(c3ccc(O)cc3)Oc3c(c(O)cc4O[C@]5([C@H](O)[C@H](c6c(O)cc(O)cc6O5)c34)c3ccc(O)cc3)[C@@H]1c1c3O[C@@H]([C@@H](O)Cc3c(O)cc1O2)c1ccc(O)cc1 | 1.3111565 | 4.1993738 | 0 | 0 | 0 | 0 | 0 | 1 | 0 | 0 | 0 | 1 | 0 | 0 | 0 |
| AUIUZJOPXKYLOS-UHFFFAOYSA-N | O(C)c1c(O)c2-c3c(cc(O)cc3)CCc2cc1OC | 2.2179969 | 2.6446861 | 0 | 0 | 0 | 1 | 0 | 0 | 0 | 1 | 0 | 0 | 0 | 0 | 0 |
| DZXMJUKEEAOGQU-UHFFFAOYSA-N | O(C)c1c(O)ccc(CCc2cc(OC)cc(OC)c2)c1 | 2.9114875 | 2.6541135 | 0 | 0 | 0 | 0 | 0 | 0 | 1 | 1 | 0 | 0 | 0 | 0 | 0 |
| NWPBSPADEDDKAO-UHFFFAOYSA-N | O(C)c1c(OC)c2-c3c(cc(O)cc3)CCc2cc1O | 2.6362482 | 2.6256186 | 0 | 0 | 0 | 0 | 0 | 0 | 1 | 1 | 0 | 0 | 0 | 0 | 0 |
| RBDNTVALYDCCCN-WCIBSUBMSA-N | O=C(OCC(O)(CO)c1c(O)cc(C)cc1)/C(=C\C)/C | 5.4511939 | 2.9234712 | 0 | 0 | 0 | 0 | 0 | 1 | 0 | 0 | 0 | 1 | 0 | 0 | 0 |
| YOHOPTQLMXBXDB-LRSUZXNRSA-N | O=C(OCC(O)(COC(=O)/C(=C\C)/C)c1c(O)cc(C)cc1)/C(=C\C)/C | 1.3323723 | 3.1536654 | 0 | 0 | 1 | 0 | 0 | 1 | 0 | 0 | 0 | 0 | 0 | 0 | 0 |
| CILMBWBPHLLNEH-UHFFFAOYSA-N | O(C)c1c(OC)cc(O)c2C(=O)C(O)=C(c3ccccc3)Oc12 | 6.2943974 | 2.9146767 | 0 | 0 | 0 | 0 | 0 | 1 | 0 | 0 | 0 | 1 | 0 | 0 | 0 |
| KIOAAZWXERJXQO-UHFFFAOYSA-N | O=C(CO)c1c(O)c2c(OC(C)(C)C=C2)cc1 | 7.4953184 | 2.5995565 | 0 | 0 | 0 | 0 | 0 | 0 | 0 | 1 | 0 | 1 | 0 | 0 | 0 |
| IYRMWMYZSQPJKC-UHFFFAOYSA-N | O=C1C(O)=C(c2ccc(O)cc2)Oc2c1c(O)cc(O)c2 | 6.469443 | 2.9038596 | 0 | 0 | 0 | 0 | 0 | 1 | 0 | 0 | 0 | 1 | 0 | 0 | 0 |
| VUNWBEIDIFFPMG-UHFFFAOYSA-N | O(C)c1c(O)c(OC)cc2c1c1c(cc(O)cc1)cc2 | 2.6302363 | 2.6065812 | 0 | 0 | 0 | 0 | 0 | 0 | 1 | 1 | 0 | 0 | 0 | 0 | 0 |
| BMSPEISBKGSBTR-UHFFFAOYSA-N | O(C)c1c(O)ccc(CCc2cc(OC)cc(O)c2)c1 | 2.9255254 | 2.6561865 | 0 | 0 | 0 | 0 | 0 | 0 | 1 | 1 | 0 | 0 | 0 | 0 | 0 |
| KVGGUNZKZGOZHC-ZDUSSCGKSA-N | O(C)c1c(C)c2[C@](O)(C)C(=C)OC(=O)c2c(O)c1 | 1.7825324 | 2.7594365 | 0 | 0 | 0 | 1 | 0 | 0 | 0 | 1 | 0 | 0 | 0 | 0 | 0 |
| RFLUVNUXLRYNNJ-LBPRGKRZSA-N | O=C1OC(=C)[C@@](O)(C)c2c(C)c(O)cc(O)c12 | 1.8527066 | 2.7574203 | 0 | 0 | 0 | 1 | 0 | 0 | 0 | 1 | 0 | 0 | 0 | 0 | 0 |
| SIXFVXJMCGPTRB-UJPOAAIJSA-N | O(C)c1ccc(O[C@H]2[C@H](O)[C@@H](O)[C@H](O)[C@@H](CO)O2)cc1 | 4.7571189 | 2.9142567 | 0 | 0 | 0 | 0 | 0 | 1 | 1 | 0 | 0 | 0 | 0 | 0 | 0 |
| PFTAWBLQPZVEMU-ZFWWWQNUSA-N | O[C@@H]1[C@H](c2cc(O)c(O)cc2)Oc2c(c(O)cc(O)c2)C1 | 5.7851629 | 2.9499514 | 0 | 0 | 0 | 0 | 0 | 1 | 0 | 0 | 0 | 1 | 0 | 0 | 0 |
| KGGCKPCQFGNZTC-UHFFFAOYSA-N | O(C)C=1C(=O)C(C)=C(O)C(=O)C=1 | 7.2482301 | 1.7634778 | 0 | 0 | 0 | 0 | 0 | 0 | 0 | 1 | 0 | 1 | 0 | 0 | 0 |
| OXXPMFLZLUGGPV-UHFFFAOYSA-N | O(C)C=1C(=O)C(C)=C(O)C(=O)C=1O | 7.480873 | 2.0319833 | 0 | 0 | 0 | 0 | 0 | 0 | 0 | 1 | 0 | 1 | 0 | 0 | 0 |
| ZGITUQPDWVLERE-UHFFFAOYSA-N | O=C1C(C)=C(O)C(=O)C=C1NC | 7.1228378 | 1.8277683 | 0 | 0 | 0 | 0 | 0 | 0 | 0 | 1 | 0 | 1 | 0 | 0 | 0 |
| LDRJANCOJOKOPS-UHFFFAOYSA-N | O(C)c1c2C(=O)C(O)=C(c3ccccc3)Oc2cc(O)c1 | 6.3357102 | 2.7659948 | 0 | 0 | 0 | 0 | 0 | 1 | 0 | 0 | 0 | 1 | 0 | 0 | 0 |
| SQFSKOYWJBQGKQ-UHFFFAOYSA-N | O(C)c1ccc(C2=C(O)C(=O)c3c(O)cc(O)cc3O2)cc1 | 6.3619718 | 2.8967477 | 0 | 0 | 0 | 0 | 0 | 1 | 0 | 0 | 0 | 1 | 0 | 0 | 0 |
| ATQPZSQVWCPVGV-UHFFFAOYSA-N | O=C(Oc1cc(O)c(C(=O)O)c(C)c1)c1c(O)cc(OC(=O)c2c(O)cc(O)cc2C)cc1C | 0 | 3.7300995 | 0 | 0 | 0 | 0 | 0 | 1 | 0 | 0 | 0 | 0 | 0 | 0 | 0 |
| OIPPWFOQEKKFEE-UHFFFAOYSA-N | Oc1cc(O)cc(C)c1 | 0 | 0.6503758 | 0 | 0 | 0 | 0 | 0 | 0 | 0 | 0 | 0 | 1 | 0 | 0 | 0 |
| XANCISIMFMVUPX-UPWRSJHQSA-N | O=C(O[C@@H]1C(C)(C)C2[C@@]3([C@]4(C([C@@]5(C)[C@@](C)([C@@H]([C@@H](CC[C@@H](C(=C)C)C)C)CC5)CC4)CC2)C3)CC1)C | 0 | 3.1462653 | 0 | 0 | 0 | 0 | 0 | 1 | 0 | 0 | 0 | 0 | 0 | 0 | 0 |
| HCUKNXBLSIDEJS-RUCUJZTOSA-N | O=C1C(C)(C)[C@H]2[C@@]3([C@]4([C@H]([C@@]5(C)[C@@](C)([C@@H]([C@@H](CC[C@@H](C(=C)C)C)C)CC5)CC4)CC2)C3)CC1 | 0 | 3.1345417 | 0 | 0 | 0 | 0 | 0 | 1 | 0 | 0 | 0 | 0 | 0 | 0 | 0 |
| OOBHNVPLRWKOTJ-ZBKSDFMUSA-N | O=C1C(C)(C)[C@H]2[C@@]3([C@]4([C@H]([C@@]5(C)[C@@](C)([C@@H]([C@@H](CC[C@H](C(=C)C)C(C)C)C)CC5)CC4)CC2)C3)CC1 | 0 | 3.1792872 | 0 | 0 | 0 | 0 | 0 | 1 | 0 | 0 | 0 | 0 | 0 | 0 | 0 |
| FEOYVNADIQNWQG-VGOCLHBASA-N | O=CO[C@@H]1C(C)(C)C2[C@@]3([C@]4(C([C@@]5(C)[C@@](C)([C@@H]([C@@H](CC[C@@H](C(=C)C)C)C)CC5)CC4)CC2)C3)CC1 | 0 | 3.1425858 | 0 | 0 | 0 | 0 | 0 | 1 | 0 | 0 | 0 | 0 | 0 | 0 | 0 |
| IXHACUTUTOCSJE-HWTFXIFRSA-N | O[C@@H]1C(C)(C)[C@H]2[C@@]3([C@]4([C@H]([C@@]5(C)[C@@](C)([C@@H]([C@@H](CC[C@@H](C(=C)C)C)C)CC5)CC4)CC2)C3)CC1 | 0 | 3.1413289 | 0 | 0 | 0 | 0 | 0 | 1 | 0 | 0 | 0 | 0 | 0 | 0 | 0 |
| OFMXGFHWLZPCFL-SVRPQWSVSA-N | O=C1[C@H](C)[C@]2(C)[C@H]([C@@]3(C)[C@@H]([C@]4(C)[C@](C)([C@H]5[C@@](C)(CC4)CCC(C)(C)C5)CC3)CC2)CC1 | 0 | 3.097709 | 0 | 0 | 0 | 0 | 0 | 1 | 0 | 0 | 0 | 0 | 0 | 0 | 0 |
| YPWQSKQSNNTXOL-TWWFCBCGSA-N | O=C1[C@H](C)[C@]2(C)[C@H]([C@@]3(C)[C@@H]([C@]4(C)[C@](C)([C@H]5[C@@](CO)(CC4)CCC(C)(C)C5)CC3)CC2)CC1 | 0 | 3.1981133 | 0 | 0 | 0 | 0 | 0 | 1 | 0 | 0 | 0 | 0 | 0 | 0 | 0 |
| ONRNCDHSZVITNY-TWWFCBCGSA-N | O=C[C@]12[C@H]([C@@]3(C)[C@@](C)([C@@H]4[C@](C)([C@H]5[C@@](C)([C@@H](C)C(=O)CC5)CC4)CC3)CC1)CC(C)(C)CC2 | 0 | 3.1781464 | 0 | 0 | 0 | 0 | 0 | 1 | 0 | 0 | 0 | 0 | 0 | 0 | 0 |
| YLXXSWJDIFLXSG-MRXNPFEDSA-N | O=C(O[C@H]1C(C)(C)Oc2c(c(OC)c3C(=O)C=C(C)Oc3c2)C1)C(C)C | 0 | 3.0345688 | 0 | 0 | 0 | 0 | 0 | 1 | 0 | 0 | 0 | 0 | 0 | 0 | 0 |
| BUTUNJHEBGRWGK-QZTJIDSGSA-N | O=C(O[C@H]1[C@@H](OC(=O)C)C(C)(C)Oc2c1c1OC(=O)C=Cc1cc2)C(C)C | 0 | 3.1760837 | 0 | 0 | 0 | 0 | 0 | 1 | 0 | 0 | 0 | 0 | 0 | 0 | 0 |
| IGMRYMQUDCGCFD-QZTJIDSGSA-N | O=C(O[C@H]1[C@@H](OC(=O)C)C(C)(C)Oc2c1c1OC(=O)C=Cc1cc2)CC | 0 | 3.1083779 | 0 | 0 | 0 | 0 | 0 | 1 | 0 | 0 | 0 | 0 | 0 | 0 | 0 |
| XYYJJFWJWWXIID-DWSJCSSQSA-N | O=C(OC/C/1=C/C=C\C(C)CC[C@H]2C(=C)C(=O)O[C@@H]2C\1)CC(C)C | 0 | 3.0457095 | 0 | 0 | 0 | 0 | 0 | 1 | 0 | 0 | 0 | 0 | 0 | 0 | 0 |
| JMXMIHVPTCKCDT-NTDRGAGCSA-N | O=C(/C=C/c1ccc(O)cc1)[C@@H]1C(C)(C)C2[C@@]3([C@]4(C([C@@]5(C)[C@@](C)([C@@H]([C@@H](CCC(C(=C)C)(C)C)C)CC5)CC4)CC2)C3)CC1 | 0 | 3.3431388 | 0 | 0 | 0 | 0 | 0 | 1 | 0 | 0 | 0 | 0 | 0 | 0 | 0 |
| QGFZRJUQPWGMGR-HLYDRURXSA-N | O[C@@H]1C(C)(C)C2[C@@](C)(C=3C([C@@]4(C)[C@@](C)([C@@H]([C@@H](CCC(C(=C)C)(C)C)C)CC4)CC=3)CC2)CC1 | 0 | 3.1531897 | 0 | 0 | 0 | 0 | 0 | 1 | 0 | 0 | 0 | 0 | 0 | 0 | 0 |
| BCONSRYNVDIUFV-HEMYWYTFSA-N | O[C@@H]1C(C)(C)C2[C@@](C)(C=3C([C@@]4(C)[C@@](C)([C@@H]([C@@H](CC[C@@H](C(=C)C)C)C)CC4)CC=3)CC2)CC1 | 0 | 3.1368665 | 0 | 0 | 0 | 0 | 0 | 1 | 0 | 0 | 0 | 0 | 0 | 0 | 0 |
| YCGKSBDRFMKJGQ-FFHDHRDUSA-N | [C@H](CCC(C(=C)C)(C)C)(C)[C@@H]1[C@]2(C)[C@](C)(C3[C@](C)([C@]45O[C@H](C(C)(C)C4CC3)CC5)CC2)CC1 | 0 | 3.1508598 | 0 | 0 | 0 | 0 | 0 | 1 | 0 | 0 | 0 | 0 | 0 | 0 | 0 |
| MPPQCCFNXBGKFC-RZTYQLBFSA-N | O(C)c1c(OC)ccc([C@H]2OC[C@@H]3[C@@H](c4c(C)c5OCOc5cc4)OC[C@H]23)c1 | 0 | 3.097153 | 0 | 0 | 0 | 0 | 0 | 1 | 0 | 0 | 0 | 0 | 0 | 0 | 0 |
| FYIHJFOIIUOEKY-LBTBCDHLSA-N | O(C)c1c([C@H]2OC[C@@H]3[C@@H](c4c(OC)c5OCOc5cc4)OC[C@H]23)ccc2OCOc12 | 0 | 3.1956695 | 0 | 0 | 0 | 0 | 0 | 1 | 0 | 0 | 0 | 0 | 0 | 0 | 0 |
| IZFXFVSOQMEZKB-MNFMDYEBSA-N | O=C1O[C@H](CCC)[C@@H](O)[C@@H](O)/C=C/CCC1 | 4.4634477 | 2.496977 | 0 | 0 | 1 | 0 | 0 | 0 | 0 | 0 | 0 | 0 | 0 | 0 | 0 |
| AFZOXJCUHIZZDU-UHFFFAOYSA-N | O=C1OC(CCCCCCCCCCCCCCCCCCCC)CC1 | 0 | 3.1069877 | 0 | 0 | 0 | 0 | 0 | 1 | 0 | 0 | 0 | 0 | 0 | 0 | 0 |
| FLVQHDPEFVLJND-UHFFFAOYSA-N | O=C1OC(CCCCCCCCCCCCCCCCCCCCC)CC1 | 0 | 3.1487607 | 0 | 0 | 0 | 0 | 0 | 1 | 0 | 0 | 0 | 0 | 0 | 0 | 0 |
| KWOWNYUWWHKUCK-UHFFFAOYSA-N | O=C1OC(CCCCCCCCCCCCCCCCCCCCCC)CC1 | 0 | 3.1862167 | 0 | 0 | 0 | 0 | 0 | 1 | 0 | 0 | 0 | 0 | 0 | 0 | 0 |
| RATVVPKFLMJAST-UHFFFAOYSA-N | O=C1OC(CCCCCCCCCCCCCCCCCCCCCCC)CC1 | 0 | 3.2198839 | 0 | 0 | 0 | 0 | 0 | 1 | 0 | 0 | 0 | 0 | 0 | 0 | 0 |
| JBDGMANPHCRIOU-UHFFFAOYSA-N | O=C1OC(CCCCCCCCCCCCCCCCCCCCCCCC)CC1 | 0 | 3.2502262 | 0 | 0 | 0 | 0 | 0 | 1 | 0 | 0 | 0 | 0 | 0 | 0 | 0 |
| BYWVQXXACNFXHT-UHFFFAOYSA-N | O=C1OC(CCCCCCCCCCCCCCCCCCCCCCCCC)CC1 | 0 | 3.2776466 | 0 | 0 | 0 | 0 | 0 | 1 | 0 | 0 | 0 | 0 | 0 | 0 | 0 |
| YLLQOTIGQVCOEH-UHFFFAOYSA-N | O=C1OC(CCCCCCCCCCCCCCCCCCCCCCCCCC)CC1 | 0 | 3.3024942 | 0 | 0 | 0 | 0 | 0 | 1 | 0 | 0 | 0 | 0 | 0 | 0 | 0 |
| VKISCDZKKVVZQH-BMRADRMJSA-N | O=C(CCCCCCCCCCCCCC/C=C/c1cc2OCOc2cc1)C | 0 | 3.051538 | 0 | 0 | 0 | 0 | 0 | 1 | 0 | 0 | 0 | 0 | 0 | 0 | 0 |
| SVTYLQARFHCNGC-UHFFFAOYSA-N | O=C(CCCCCCCCCCCCCCCCc1cc2OCOc2cc1)C | 0 | 3.0576305 | 0 | 0 | 0 | 0 | 0 | 1 | 0 | 0 | 0 | 0 | 0 | 0 | 0 |
| PCDQKEPIJYVOPG-NHBPQBDCSA-N | O=C(OC)[C@]12[C@@]3(O)[C@@](C)(CC[C@]1(C)[C@H]1[C@H](O)C[C@]4([C@H](O)C)[C@H]([C@]1(C)CC2)C=CC(=O)OC4)CCC(=C)C3 | 0 | 3.5617642 | 0 | 0 | 0 | 0 | 0 | 1 | 0 | 0 | 0 | 0 | 0 | 0 | 0 |
| KBMOWKVFQHQUFB-NHBPQBDCSA-N | O=C(OC)[C@]12[C@@]3(O)[C@@](C)(CC[C@]1(C)[C@H]1[C@H](O)C[C@]4([C@H](O)C)[C@H]([C@]1(C)CC2)C=CC(=O)OC4)CCC(C)=C3 | 0 | 3.5353096 | 0 | 0 | 0 | 0 | 0 | 1 | 0 | 0 | 0 | 0 | 0 | 0 | 0 |
| IBYSPSUANRCDTF-PJFLNFRUSA-N | O=C(OC)[C@]12[C@@]3(O)[C@@](C)(CC[C@]1(C)[C@H]1[C@H](O)[C@H](O)[C@]4([C@H](O)C)[C@H]([C@]1(C)CC2)C=CC(=O)OC4)CCC(=C)C3 | 0 | 3.7339233 | 0 | 0 | 0 | 0 | 0 | 1 | 0 | 0 | 0 | 0 | 0 | 0 | 0 |
| MYRCCYOWAVWIKR-NHBPQBDCSA-N | O=C(OC)[C@]12[C@@]3(O)[C@](C)(CC=C(C)C3)CC[C@]1(C)[C@H]1[C@H](O)C[C@]3([C@H](O)C)[C@H]([C@]1(C)CC2)C=CC(=O)OC3 | 0 | 3.545654 | 0 | 0 | 0 | 0 | 0 | 1 | 0 | 0 | 0 | 0 | 0 | 0 | 0 |
| HRMXSRHLUOTLBH-PJFLNFRUSA-N | O=C(OC)[C@]12[C@@]3(O)[C@](C)(CC=C(C)C3)CC[C@]1(C)[C@H]1[C@H](O)[C@H](O)[C@]3([C@H](O)C)[C@H]([C@]1(C)CC2)C=CC(=O)OC3 | 0 | 3.7081114 | 0 | 0 | 0 | 0 | 0 | 1 | 0 | 0 | 0 | 0 | 0 | 0 | 0 |
| PLOHJRTXRRLBGH-STODXTIPSA-N | O=C(O[C@@H]1[C@H](O)[C@]2([C@H](O)C)[C@H]([C@@]3(C)[C@H]1[C@]1(C)[C@](C(=O)OC)([C@@]4(O)[C@@](C)(CC1)CCC(=C)C4)CC3)C=CC(=O)OC2)C | 0 | 3.7478392 | 0 | 0 | 0 | 0 | 0 | 1 | 0 | 0 | 0 | 0 | 0 | 0 | 0 |
| BQZKAEWQBJHAIN-STODXTIPSA-N | O=C(O[C@@H]1[C@H](O)[C@]2([C@H](O)C)[C@H]([C@@]3(C)[C@H]1[C@]1(C)[C@](C(=O)OC)([C@@]4(O)[C@](C)(CC=C(C)C4)CC1)CC3)C=CC(=O)OC2)C | 0 | 3.7053411 | 0 | 0 | 0 | 0 | 0 | 1 | 0 | 0 | 0 | 0 | 0 | 0 | 0 |
| DFTOCERTSAMQSM-STODXTIPSA-N | O=C(O[C@H]1[C@@H](O)[C@@H]2[C@]3(C)[C@](C(=O)OC)([C@@]4(O)[C@@](C)(CC3)CCC(=C)C4)CC[C@@]2(C)[C@H]2[C@]1([C@H](O)C)COC(=O)C=C2)C | 0 | 3.7427323 | 0 | 0 | 0 | 0 | 0 | 1 | 0 | 0 | 0 | 0 | 0 | 0 | 0 |
| MAASNULAYUTHCM-STODXTIPSA-N | O=C(O[C@H]1[C@@H](O)[C@@H]2[C@]3(C)[C@](C(=O)OC)([C@@]4(O)[C@](C)(CC=C(C)C4)CC3)CC[C@@]2(C)[C@H]2[C@]1([C@H](O)C)COC(=O)C=C2)C | 0 | 3.7092905 | 0 | 0 | 0 | 0 | 0 | 1 | 0 | 0 | 0 | 0 | 0 | 0 | 0 |
| GAWIXWVDTYZWAW-UHFFFAOYSA-N | O[C@H](C | 0 | 0.496305 | 0 | 0 | 0 | 0 | 0 | 0 | 0 | 0 | 0 | 1 | 0 | 0 | 0 |
| DNVPQKQSNYMLRS-APGDWVJJSA-N | O[C@@H]1CC=2[C@@](C)([C@@H]3C([C@H]4[C@@](C)([C@@H]([C@@H](/C=C/[C@@H](C(C)C)C)C)CC4)CC3)=CC=2)CC1 | 0 | 3.0388315 | 0 | 0 | 0 | 0 | 0 | 1 | 0 | 0 | 0 | 0 | 0 | 0 | 0 |
| SLFMBUBDOYEWDU-XIDJHNHKSA-N | O=C1C(C)(C)C2[C@@]3([C@]4(C([C@@]5(C)[C@@](C)([C@@H]([C@@H](CCC(C(=C)C)(C)C)C)CC5)CC4)CC2)C3)CC1 | 0 | 3.1667886 | 0 | 0 | 0 | 0 | 0 | 1 | 0 | 0 | 0 | 0 | 0 | 0 | 0 |
| WWZTVIKABWJXIY-IRXLFIPWSA-N | O[C@@H]1C(C)(C)C2[C@@]3([C@]4(C([C@@]5(C)[C@@](C)([C@@H]([C@@H](CCC(C(=C)C)(C)C)C)CC5)CC4)CC2)C3)CC1 | 0 | 3.1762691 | 0 | 0 | 0 | 0 | 0 | 1 | 0 | 0 | 0 | 0 | 0 | 0 | 0 |
| PENVZGWIOSIDJF-UHFFFAOYSA-N | O(C)c1c2C(=O)CC(c3cc(-c4c(O)c5C(=O)C=C(c6ccc(O)cc6)Oc5cc4O)c(O)cc3)Oc2cc(O)c1 | 0 | 3.7301955 | 0 | 0 | 0 | 0 | 0 | 1 | 0 | 0 | 0 | 0 | 0 | 0 | 0 |
| SFEUTIOWNUGQMZ-ZHLOSDGBSA-N | O=C(O[C@@H]1C(C)(C)[C@H]2[C@@](C)([C@@H]3[C@](C)([C@@]4(C)[C@@H]([C@H]5[C@H](C)C(=C)CC[C@]5(C)CC4)CC3)CC2)CC1)C | 0 | 3.1617268 | 0 | 0 | 0 | 0 | 0 | 1 | 0 | 0 | 0 | 0 | 0 | 0 | 0 |
| NZCULBURCGAPSF-KTWGDNJDSA-N | O=C(O)[C@@]12[C@@H]([C@@H](C)[C@H](C)CC1)C=1[C@](C)([C@@]3(C)[C@@H]([C@]4(C)[C@H]([C@](CO)(C)[C@H](O)CC4)CC3)CC=1)CC2 | 0 | 3.3057255 | 0 | 0 | 0 | 0 | 0 | 1 | 0 | 0 | 0 | 0 | 0 | 0 | 0 |
| QAIPRVGONGVQAS-DUXPYHPUSA-N | O=C(O)/C=C/c1cc(O)c(O)cc1 | 6.1188421 | 2.0517014 | 0 | 0 | 0 | 0 | 0 | 0 | 0 | 0 | 0 | 1 | 0 | 0 | 0 |
| LNTHITQWFMADLM-UHFFFAOYSA-N | O=C(O)c1cc(O)c(O)c(O)c1 | 6.2557938 | 2.0201058 | 0 | 0 | 0 | 0 | 0 | 0 | 0 | 0 | 0 | 1 | 0 | 0 | 0 |
| FBSFWRHWHYMIOG-UHFFFAOYSA-N | O=C(OC)c1cc(O)c(O)c(O)c1 | 6.2217643 | 2.0523634 | 0 | 0 | 0 | 0 | 0 | 0 | 0 | 0 | 0 | 1 | 0 | 0 | 0 |
| UWHUTZOCTZJUKC-JKSUJKDBSA-N | Oc1c(O)cc2c([C@H]3[C@](O)(COc4c3ccc(O)c4)C2)c1 | 5.2765423 | 2.7733625 | 0 | 0 | 0 | 0 | 0 | 1 | 0 | 0 | 0 | 0 | 0 | 0 | 0 |
| QCDYQQDYXPDABM-UHFFFAOYSA-N | Oc1cc(O)cc(O)c1 | 0 | 0.6590587 | 0 | 0 | 0 | 0 | 0 | 0 | 0 | 0 | 0 | 1 | 0 | 0 | 0 |
| FXJPPIJUTVUQRL-NIQLMDKRSA-N | O=C(O[C@@H]1[C@H](OC(=O)c2ccccc2)[C@]2(C)[C@@H](OC(=O)C)CC[C@@H](C)[C@]32[C@H](O)[C@@H]1C(C)(C)O3)/C=C/c1ccccc1 | 0 | 3.478107 | 0 | 0 | 0 | 0 | 0 | 1 | 0 | 0 | 0 | 0 | 0 | 0 | 0 |
| KAPQKCUDPVRZEX-ZGIPRAPUSA-N | O=C(O[C@@H]1[C@H](OC(=O)c2ccccc2)[C@]2(C)[C@@H](OC(=O)C)CC[C@@](O)(C)[C@]32[C@H](O)[C@@H]1C(C)(C)O3)/C=C/c1ccccc1 | 0.0084358 | 3.7158797 | 0 | 0 | 0 | 0 | 0 | 1 | 0 | 0 | 0 | 0 | 0 | 0 | 0 |
| OVQQPOHQLNLSCP-HHATXKITSA-N | O=C(O[C@@H]1[C@H](OC(=O)c2ccccc2)[C@]2(C)[C@@H](OC(=O)C)CC[C@@](O)(C)[C@]32[C@H](OC(=O)C)[C@@H]1C(C)(C)O3)/C=C/c1ccccc1 | 0 | 3.8236869 | 0 | 0 | 0 | 0 | 0 | 1 | 0 | 0 | 0 | 0 | 0 | 0 | 0 |
| AGFAYIPDDGSEBN-IBXLWUJLSA-N | O[C@@]12c3c(OC1O[C@]1([C@@H](C)Oc4c1ccc(C)c4)CC2)cc(C)cc3 | 1.3230164 | 2.9362312 | 0 | 0 | 0 | 0 | 0 | 0 | 0 | 1 | 0 | 0 | 0 | 0 | 0 |
| IXNDLYSEFZRJIJ-QIXKMHTASA-N | O=C(O)C[C@@]1(C)CC2[C@@]3(C)[C@@](C)(C4[C@](C)(C5[C@@](C)([C@@H](C)C(=O)CC5)CC4)CC3)CC[C@@]2(C)CC1 | 0 | 3.251792 | 0 | 0 | 0 | 0 | 0 | 1 | 0 | 0 | 0 | 0 | 0 | 0 | 0 |
| OFMXGFHWLZPCFL-QDTNQAACSA-N | O=C1[C@H](C)[C@]2(C)C([C@@]3(C)C([C@]4(C)[C@](C)(C5[C@@](C)(CC4)CCC(C)(C)C5)CC3)CC2)CC1 | 0 | 3.097709 | 0 | 0 | 0 | 0 | 0 | 1 | 0 | 0 | 0 | 0 | 0 | 0 | 0 |
| CAMPYEYUASPOIB-IPQGBBEOSA-N | O=C1[C@H](C)[C@]2(C)C([C@@]3(C)C([C@]4(C)[C@](C)(C5[C@@](C)(CC4)CC[C@](CCO)(C)C5)CC3)CC2)CC1 | 0 | 3.2245085 | 0 | 0 | 0 | 0 | 0 | 1 | 0 | 0 | 0 | 0 | 0 | 0 | 0 |
| YPWQSKQSNNTXOL-JQKPCDNZSA-N | O=C1[C@H](C)[C@]2(C)C([C@@]3(C)C([C@]4(C)[C@](C)(C5[C@@](CO)(CC4)CCC(C)(C)C5)CC3)CC2)CC1 | 0 | 3.1981133 | 0 | 0 | 0 | 0 | 0 | 1 | 0 | 0 | 0 | 0 | 0 | 0 | 0 |
| XCDQFROEGGNAER-PPGXERKRSA-N | O[C@@H]1[C@H](C)[C@]2(C)C([C@@]3(C)C([C@]4(C)[C@](C)(C5[C@@](C)(CC4)CCC(C)(C)C5)CC3)CC2)CC1 | 0 | 3.1214906 | 0 | 0 | 0 | 0 | 0 | 1 | 0 | 0 | 0 | 0 | 0 | 0 | 0 |
| VOYZLKWKVLYJHD-IDELEHTOSA-N | O=C(O)/C(=C\CC[C@@H](C)[C@H]1[C@@]2(C)[C@@](C)(C=3[C@@H]([C@]4(C)C(C(C)(C)C(=O)CC4)CC=3)CC2)CC1)/C | 0 | 3.2791495 | 0 | 0 | 0 | 0 | 0 | 1 | 0 | 0 | 0 | 0 | 0 | 0 | 0 |
| UILQHUKSFUOOLH-XGRQLWIPSA-N | O=C(O)/C(=C\CC[C@@H](C)[C@H]1[C@@]2(C)[C@@](C)(C=3[C@@H]([C@]4(C)C(C(C)(C)[C@H](O)CC4)CC=3)CC2)CC1)/C | 0 | 3.2869414 | 0 | 0 | 0 | 0 | 0 | 1 | 0 | 0 | 0 | 0 | 0 | 0 | 0 |
| JFSHUTJDVKUMTJ-QHPUVITPSA-N | O[C@@H]1C(C)(C)[C@H]2[C@@](C)([C@@H]3[C@](C)([C@@]4(C)C([C@H]5[C@@](C)(CC4)CCC(C)(C)C5)=CC3)CC2)CC1 | 0 | 3.1028222 | 0 | 0 | 0 | 0 | 0 | 1 | 0 | 0 | 0 | 0 | 0 | 0 | 0 |
| KZJWDPNRJALLNS-VJSFXXLFSA-N | O[C@@H]1CC=2[C@@](C)([C@@H]3[C@H]([C@H]4[C@@](C)([C@@H]([C@@H](CC[C@H](C(C)C)CC)C)CC4)CC3)CC=2)CC1 | 0 | 3.1355353 | 0 | 0 | 0 | 0 | 0 | 1 | 0 | 0 | 0 | 0 | 0 | 0 | 0 |
| UBBRXVRQZJSDAK-FGUXGJARSA-N | O=C1O/C(=C/CCC)/C2=C1C1[C@H]([C@H]3C=C4C(=O)O/C(=C\CCC)/[C@@]14CC3)CC2 | 0 | 3.1470341 | 0 | 0 | 0 | 0 | 0 | 1 | 0 | 0 | 0 | 0 | 0 | 0 | 0 |
| VOYZLKWKVLYJHD-DSRSRPTBSA-N | O=C(O)/C(=C/CC[C@H](C)[C@H]1[C@@]2(C)[C@@](C)(C=3C([C@]4(C)C(C(C)(C)C(=O)CC4)CC=3)CC2)CC1)/C | 0 | 3.2791495 | 0 | 0 | 0 | 0 | 0 | 1 | 0 | 0 | 0 | 0 | 0 | 0 | 0 |
| UILQHUKSFUOOLH-YOOZGKBQSA-N | O=C(O)/C(=C/CC[C@H](C)[C@H]1[C@@]2(C)[C@@](C)(C=3C([C@]4(C)C(C(C)(C)[C@H](O)CC4)CC=3)CC2)CC1)/C | 0 | 3.2869414 | 0 | 0 | 0 | 0 | 0 | 1 | 0 | 0 | 0 | 0 | 0 | 0 | 0 |
| MIJYXULNPSFWEK-LGSDIRQTSA-N | O=C(O)[C@]12C(C=3[C@](C)([C@@]4(C)C([C@]5(C)C(C(C)(C)[C@H](O)CC5)CC4)CC=3)CC1)CC(C)(C)CC2 | 0 | 3.2948674 | 0 | 0 | 0 | 0 | 0 | 1 | 0 | 0 | 0 | 0 | 0 | 0 | 0 |
| YXHVCZZLWZYHSA-FPLPWBNLSA-N | O=C(O)c1c(O)cccc1CCCCCCC/C=C\CCCCCC | 0 | 3.1768565 | 0 | 0 | 0 | 0 | 0 | 1 | 0 | 0 | 0 | 0 | 0 | 0 | 0 |
| ADFWQBGTDJIESE-UHFFFAOYSA-N | O=C(O)c1c(O)cccc1CCCCCCCCCCCCCCC | 0 | 3.2086029 | 0 | 0 | 0 | 0 | 0 | 1 | 0 | 0 | 0 | 0 | 0 | 0 | 0 |
| OSXBYONEOVGKOX-ARJAWSKDSA-N | O=C(O)c1c(O)cccc1CCCCCCCCCCCCCCC/C=C\CC | 0 | 3.3206673 | 0 | 0 | 0 | 0 | 0 | 1 | 0 | 0 | 0 | 0 | 0 | 0 | 0 |
| KLYPIICREBFTGY-UHFFFAOYSA-N | O=C(O)c1c(O)cccc1CCCCCCCCCCCCCCCCCCC | 0 | 3.3406834 | 0 | 0 | 0 | 0 | 0 | 1 | 0 | 0 | 0 | 0 | 0 | 0 | 0 |
| OFAIMNXQEZOWPU-RXKIPKFJSA-N | O=C(O[C@H]1c2c(c(O)cc(C)c2)C(O)=C2C(=O)[C@@H]3c4c(O)c(C(=O)c5c(C(=O)OC)c(O)ccc5O)c(O)cc4C[C@]12C=C3)C | 0 | 4.1543919 | 0 | 0 | 0 | 0 | 0 | 1 | 0 | 0 | 0 | 0 | 0 | 0 | 0 |
| OXAJFKDURZRJOW-KRWDZBQOSA-N | O=C(OC)[C@@]12C(=O)C(C)=COC(OC)=C1C(=O)c1c(O)cccc1O2 | 0 | 3.2214148 | 0 | 0 | 0 | 0 | 0 | 1 | 0 | 0 | 0 | 0 | 0 | 0 | 0 |
| RLLCMRPOSFZYJJ-UHFFFAOYSA-N | O=C(Oc1c(C)c(O)c(C(=O)O)c(C)c1)c1c(OC)c(C)c(OC(=O)c2c(O)c(C)c(O)cc2C)c(C)c1C | 0 | 3.7485118 | 0 | 0 | 0 | 0 | 0 | 1 | 0 | 0 | 0 | 0 | 0 | 0 | 0 |
| MGGMNKJGDSNTKZ-UHFFFAOYSA-N | O=C(Oc1c(C)c(O)c(C(=O)O)c(C)c1C)c1c(O)c(C)c(OC(=O)c2c(O)c(C)c(O)cc2C)c(C)c1C | 0 | 3.7698875 | 0 | 0 | 0 | 0 | 0 | 1 | 0 | 0 | 0 | 0 | 0 | 0 | 0 |
| IGYZEKLJQWCRPT-UHFFFAOYSA-N | O=C(Oc1c(C)c(O)c(C(=O)O)c(C)c1C)c1c(OC)c(C)c(OC(=O)c2c(O)c(C)c(O)cc2C)c(C)c1C | 0 | 3.7556718 | 0 | 0 | 0 | 0 | 0 | 1 | 0 | 0 | 0 | 0 | 0 | 0 | 0 |
| SZCAUZSZQPVKQY-CCFHIKDMSA-N | O=C(OCCCCCCCCCCCCCCCCCCCCCCCCCC)/C=C/c1ccc(O)cc1 | 0 | 3.399452 | 0 | 0 | 0 | 0 | 0 | 1 | 0 | 0 | 0 | 0 | 0 | 0 | 0 |
| CWHBUKZWMZSTEA-KKYHWDRJSA-N | O=C(OCCCCCCCCCCCCCCCCCCCCCCCCCCCC)/C=C/c1ccc(O)cc1 | 0 | 3.4270963 | 0 | 0 | 0 | 0 | 0 | 1 | 0 | 0 | 0 | 0 | 0 | 0 | 0 |
| DUIIKPOJUKGTSI-LVYIWIAJSA-N | O=C(OCCCCCCCCCCCCCCCCCCCCCCCCCCCCCC)/C=C/c1ccc(O)cc1 | 0 | 3.4502997 | 0 | 0 | 0 | 0 | 0 | 1 | 0 | 0 | 0 | 0 | 0 | 0 | 0 |
| RGHHSNMVTDWUBI-UHFFFAOYSA-N | O=Cc1ccc(O)cc1 | 0 | 0.6015263 | 0 | 0 | 0 | 0 | 0 | 0 | 0 | 0 | 0 | 1 | 0 | 0 | 0 |
| JUVIOZPCNVVQFO-HBGVWJBISA-N | O(C)c1c(OC)cc2[C@@H]3C(=O)c4c(O[C@@H]3COc2c1)c1c(O[C@@H](C(=C)C)C1)cc4 | 0 | 3.0896964 | 0 | 0 | 0 | 0 | 0 | 1 | 0 | 0 | 0 | 0 | 0 | 0 | 0 |
| ZRZWBWPDBOVIGQ-YWZWRZHGSA-N | O=C1N(C)[C@]2(Cc3c4c(n([C@@]56[C@@H](N7C(=O)[C@@]8(CO)N(C)C(=O)[C@@]7(SS8)C5)Nc5c6cccc5)c3)cccc4)C(=O)N(C)[C@@]1(CO)SS2 | 0.5395745 | 3.9653888 | 0 | 0 | 0 | 0 | 0 | 1 | 0 | 0 | 0 | 0 | 0 | 0 | 0 |
| HHGPYJLEJGNWJA-UHFFFAOYSA-N | O(C)c1c(OC)ccc(C2=C(OC)C(=O)c3c(O)cc(OC)cc3O2)c1 | 0 | 3.1821762 | 0 | 0 | 0 | 0 | 0 | 1 | 0 | 0 | 0 | 0 | 0 | 0 | 0 |
| XUAORUWVUTVEEC-UHFFFAOYSA-N | O=C1c2c(O)c(c(O)cc2OC(c2ccc(O)cc2)=C1)-c1c(O)ccc(C2Oc3c(c(O)cc(O)c3)C(=O)C2)c1 | 0 | 4.0568852 | 0 | 0 | 0 | 0 | 0 | 1 | 0 | 0 | 0 | 0 | 0 | 0 | 0 |
| BORWSEZUWHQTOK-UHFFFAOYSA-N | O=C1c2c(O)c(c(O)cc2OC(c2ccc(O)cc2)=C1)-c1c(O)ccc(C=2Oc3c(c(O)cc(O)c3)C(=O)C=2)c1 | 0 | 4.0127318 | 0 | 0 | 0 | 0 | 0 | 1 | 0 | 0 | 0 | 0 | 0 | 0 | 0 |
| YUSWMAULDXZHPY-UHFFFAOYSA-N | O=C1c2c(O)cc(O)c(-c3c(O)ccc(C=4Oc5c(c(O)cc(O)c5)C(=O)C=4)c3)c2OC(c2ccc(O)cc2)=C1 | 0 | 4.0095369 | 0 | 0 | 0 | 0 | 0 | 1 | 0 | 0 | 0 | 0 | 0 | 0 | 0 |
| UBBRXVRQZJSDAK-ZJHGLIIDSA-N | O=C1O/C(=C\CCC)/C2=C1[C@H]1[C@H]([C@H]3C=C4C(=O)O/C(=C\CCC)/[C@@]14CC3)CC2 | 0 | 3.1470341 | 0 | 0 | 0 | 0 | 0 | 1 | 0 | 0 | 0 | 0 | 0 | 0 | 0 |
| STSOHAOGZMLWFR-SSDLBLMSSA-N | O=C1O[C@@H](C)[C@@H](O)c2c1c(O)ccc2 | 2.4061424 | 2.3472772 | 0 | 0 | 0 | 0 | 0 | 0 | 0 | 1 | 0 | 0 | 0 | 0 | 0 |
| STSOHAOGZMLWFR-CDUCUWFYSA-N | O=C1O[C@@H](C)[C@H](O)c2c1c(O)ccc2 | 2.4061424 | 2.3472772 | 0 | 0 | 0 | 0 | 0 | 0 | 0 | 1 | 0 | 0 | 0 | 0 | 0 |
| GAWIXWVDTYZWAW-UHFFFAOYSA-N | O([C@@H](C | 0 | 0.496305 | 0 | 0 | 0 | 0 | 0 | 0 | 0 | 0 | 0 | 1 | 0 | 0 | 0 |
| ODHSWYNEXJIJEI-YKJBWMMYSA-N | O=C(OC)C[C@H]1C(C)(C)[C@H](OC(=O)C)[C@@]2(O)C(=O)[C@]1(C)C1C(=C2)C2[C@](C)([C@H](c3cocc3)OC(=O)C2)CC1 | 0 | 3.6002806 | 0 | 0 | 0 | 0 | 0 | 1 | 0 | 0 | 0 | 0 | 0 | 0 | 0 |
| NMEMUNAMFPMDHM-ICQCPBIVSA-N | O=C(OC)C[C@H]1C(C)(C)[C@H](OC(=O)C)[C@@]2(O)C(=O)[C@]1(C)C1[C@@]3(O[C@H]23)C2[C@](C)([C@H](c3cocc3)OC(=O)C2)CC1 | 0 | 3.6322327 | 0 | 0 | 0 | 0 | 0 | 1 | 0 | 0 | 0 | 0 | 0 | 0 | 0 |
| JJTOVDGWVPPWNZ-DTFVITINSA-N | O=C(O[C@H]1C(C)(C)[C@H](CC(=O)OC)[C@]2(C)C(=O)[C@@]1(O)C=C1C3[C@](C)([C@H](c4cocc4)OC(=O)C3)CCC21)/C(=C/C)/C | 0 | 3.5894764 | 0 | 0 | 0 | 0 | 0 | 1 | 0 | 0 | 0 | 0 | 0 | 0 | 0 |
| MBIKCFMEQQFVRE-BTZXGELBSA-N | O=C(O[C@H]1C(C)(C)[C@H](CC(=O)OC)[C@]2(C)C(=O)[C@@]1(O)C=C1C3[C@](C)([C@H](c4cocc4)OC(=O)C3)CCC21)C(C)C | 0 | 3.5997176 | 0 | 0 | 0 | 0 | 0 | 1 | 0 | 0 | 0 | 0 | 0 | 0 | 0 |
| LOOFGUAWTZPQEG-BWSSPMROSA-N | O=C(O[C@H]1C(C)(C)[C@H](CC(=O)OC)[C@]2(C)C(=O)[C@@]1(O)[C@H]1O[C@@]31C1[C@](C)([C@H](c4cocc4)OC(=O)C1)CCC23)C(C)C | 0 | 3.6300137 | 0 | 0 | 0 | 0 | 0 | 1 | 0 | 0 | 0 | 0 | 0 | 0 | 0 |
| UMAZUSDJQFBJAR-JLZHRPIQSA-N | O=C(O[C@H]1C(C)(C)[C@H](CC(=O)OC)[C@]2(C)C(=O)[C@@]1(OC(=O)C)C=C1C3[C@](C)([C@H](c4cocc4)OC(=O)C3)CCC21)/C(=C/C)/C | 0 | 3.6050351 | 0 | 0 | 0 | 0 | 0 | 1 | 0 | 0 | 0 | 0 | 0 | 0 | 0 |
| IQZMMMBJYWYVKW-MQDCWUPWSA-N | O=C(O[C@H]1C(C)(C)[C@H](CC(=O)OC)[C@]2(C)C(=O)[C@@]1(OC(=O)C)C=C1C3[C@](C)([C@H](c4cocc4)OC(=O)C3)CCC21)C(C)C | 0 | 3.6158788 | 0 | 0 | 0 | 0 | 0 | 1 | 0 | 0 | 0 | 0 | 0 | 0 | 0 |
| COKNSFVQXLZHTF-YZMSHABZSA-N | O=C(O[C@H]1C(C)(C)[C@H](CC(=O)OC)[C@]2(C)C(=O)[C@@]1(OC(=O)C)[C@H]1O[C@@]31C1[C@](C)([C@H](c4cocc4)OC(=O)C1)CCC23)C(C)C | 0 | 3.6481053 | 0 | 0 | 0 | 0 | 0 | 1 | 0 | 0 | 0 | 0 | 0 | 0 | 0 |
| RFYQGKUTWAUUJW-UHFFFAOYSA-N | O(C)C=1C(=O)C(C)=C(Nc2ccc(CCCC)cc2)C(=O)C=1 | 4.7519727 | 2.7918845 | 0 | 0 | 0 | 0 | 0 | 1 | 0 | 0 | 0 | 0 | 0 | 0 | 0 |
| WCGUUGGRBIKTOS-GPOJBZKASA-N | O=C(O)[C@@]12[C@@H]([C@@H](C)[C@H](C)CC1)C=1[C@](C)([C@@]3(C)[C@@H]([C@]4(C)[C@H](C(C)(C)[C@@H](O)CC4)CC3)CC=1)CC2 | 0 | 3.2919114 | 0 | 0 | 0 | 0 | 0 | 1 | 0 | 0 | 0 | 0 | 0 | 0 | 0 |
| XUARCIYIVXVTAE-ZAPOICBTSA-N | OC[C@@]12[C@@H]([C@@H](C)[C@H](C)CC1)C=1[C@](C)([C@@]3(C)[C@@H]([C@]4(C)[C@H](C(C)(C)[C@@H](O)CC4)CC3)CC=1)CC2 | 0 | 3.2365048 | 0 | 0 | 0 | 0 | 0 | 1 | 0 | 0 | 0 | 0 | 0 | 0 | 0 |
| RILOVTGOFDASJT-LURJTMIESA-N | Clc1c(O)c2C(=O)c3c(O)cc(C[C@@H](O)C)cc3C(=O)c2cc1O | 0.0438117 | 3.3276718 | 0 | 0 | 0 | 0 | 0 | 1 | 0 | 0 | 0 | 0 | 0 | 0 | 0 |
| HJJVLOSOGVDXLM-UHFFFAOYSA-N | O(C)c1c2c3c(c(O)c1)C(=O)C=C(OC)C3=CC1(C(OC)=CC(=O)c3c(O)cc(OC)cc13)C2 | 0 | 3.5526958 | 0 | 0 | 0 | 0 | 0 | 1 | 0 | 0 | 0 | 0 | 0 | 0 | 0 |
| ORUUOHWWRMGNJL-RGXZNCPUSA-N | O=C(O[C@@]12[C@@](C(C=C)(C)C)(N3C(=O)[C@H](Cc4ccccc4)OC(=O)[C@@H]3C1)Nc1c2cccc1)C | 1.016528 | 3.1877748 | 0 | 0 | 0 | 0 | 0 | 1 | 0 | 0 | 0 | 0 | 0 | 0 | 0 |
| CRPNQSVBEWWHIJ-UHFFFAOYSA-N | O=Cc1c(O)c(O)c(O)cc1 | 6.2578213 | 1.3847689 | 0 | 0 | 0 | 0 | 0 | 0 | 0 | 1 | 0 | 0 | 0 | 0 | 0 |
| NOEGNKMFWQHSLB-UHFFFAOYSA-N | O=Cc1oc(CO)cc1 | 3.5446209 | 0.6624662 | 0 | 0 | 0 | 0 | 0 | 0 | 0 | 0 | 0 | 1 | 0 | 0 | 0 |
| NIMIDMFTGGTHOH-VYRBHSGPSA-N | O(C)c1c2C(=O)[C@H](O)C(c3ccccc3)Oc2cc(O)c1 | 5.2460052 | 2.7892344 | 0 | 0 | 0 | 0 | 0 | 1 | 0 | 0 | 0 | 0 | 0 | 0 | 0 |
| QPOCKDYYXFOBCM-VYRBHSGPSA-N | O(C)c1cc(O)c2C(=O)[C@H](O)C(c3ccccc3)Oc2c1 | 5.2986625 | 2.7892344 | 0 | 0 | 0 | 0 | 0 | 1 | 0 | 0 | 0 | 0 | 0 | 0 | 0 |
| SUYJZKRQHBQNCA-MLCCFXAWSA-N | O=C1[C@H](O)C(c2ccccc2)Oc2c1c(O)cc(O)c2 | 5.3931157 | 2.7972269 | 0 | 0 | 0 | 0 | 0 | 1 | 0 | 0 | 0 | 0 | 0 | 0 | 0 |
| QDLAGTHXVHQKRE-UHFFFAOYSA-N | O(C)c1cc(O)c2C(=O)c3c(C)cc(OC)cc3Oc2c1 | 0 | 2.7290598 | 0 | 0 | 0 | 0 | 0 | 0 | 0 | 0 | 0 | 0 | 0 | 0 | 0 |
| PXFMUVDLJWXOQM-UHFFFAOYSA-N | O=C(OC)c1c(O)c(C=O)c(O)cc1C | 0 | 2.2987815 | 0 | 0 | 0 | 0 | 0 | 0 | 0 | 0 | 0 | 0 | 0 | 0 | 0 |
| NCCWCZLEACWJIN-UHFFFAOYSA-N | O=C(OC)c1c(O)cc(O)cc1C | 0 | 2.1975236 | 0 | 0 | 0 | 0 | 0 | 0 | 0 | 0 | 0 | 0 | 0 | 0 | 0 |
| LRMGCIMCOOHPQA-UHFFFAOYSA-N | O=C(Oc1cc(O)cc(C)c1)c1c(O)cc(O)cc1C | 0 | 2.7664165 | 0 | 0 | 0 | 0 | 0 | 0 | 0 | 0 | 0 | 0 | 0 | 0 | 0 |
| AMLRGXJZYUJGES-UHFFFAOYSA-N | O(C)c1c2c(c(O)c3C(=O)C(C)=C(C)Oc13)c(OC)cc(OC)c2 | 0 | 2.9611102 | 0 | 0 | 0 | 0 | 0 | 0 | 0 | 0 | 0 | 0 | 0 | 0 | 0 |
| UMPIOBMYFRLDPN-UHFFFAOYSA-N | O(C)c1c2c(c(O)c3C(=O)C=C(C)Oc13)c(OC)cc(OC)c2 | 0 | 2.9170192 | 0 | 0 | 0 | 0 | 0 | 0 | 0 | 0 | 0 | 0 | 0 | 0 | 0 |
| CQIUKKVOEOPUDV-IYSWYEEDSA-N | O=C(O)C=1C(=O)C(C)=C2[C@H](C)[C@@H](C)OC=C2C=1O | 0 | 2.894399 | 0 | 0 | 0 | 0 | 0 | 0 | 0 | 0 | 0 | 0 | 0 | 0 | 0 |
| LXCFILQKKLGQFO-UHFFFAOYSA-N | O=C(OC)c1ccc(O)cc1 | 0 | 1.2456977 | 0 | 0 | 0 | 0 | 0 | 0 | 0 | 0 | 0 | 0 | 0 | 0 | 0 |
| RHMXXJGYXNZAPX-UHFFFAOYSA-N | O=C1c2c(O)cc(O)cc2C(=O)c2c1c(O)cc(C)c2 | 0 | 2.8607482 | 0 | 0 | 0 | 0 | 0 | 0 | 0 | 0 | 0 | 0 | 0 | 0 | 0 |
| AHRNUZBHBAHMHY-GFCCVEGCSA-N | Oc1c(C)cc([C@@H](CC/C=C(\C)/C)C)cc1 | 0 | 2.5172445 | 0 | 0 | 0 | 0 | 0 | 0 | 0 | 0 | 0 | 0 | 0 | 0 | 0 |
| FLGAFUAMEXILLB-QXENPCNLSA-N | O=C(O)[C@@]1(C)[C@@H]2[C@](C)([C@H]3[C@@]4(C[C@]5(C)[C@@H]([C@H]5C4)C3)CC2)CCC1 | 0 | 2.9701534 | 0 | 0 | 0 | 0 | 0 | 0 | 0 | 0 | 0 | 0 | 0 | 0 | 0 |
| FKWGCEDRLNNZOZ-GFCCVEGCSA-N | Oc1c(C)ccc([C@@H](CC/C=C(\C)/C)C)c1 | 0 | 2.5757841 | 0 | 0 | 0 | 0 | 0 | 0 | 0 | 0 | 0 | 0 | 0 | 0 | 0 |
| BQAQGBHVNVLESL-IAPIXIRKSA-N | Oc1c(C)ccc([C@@H](CCC2C(C)(C)O2)C)c1 | 0 | 2.6210888 | 0 | 0 | 0 | 0 | 0 | 0 | 0 | 0 | 0 | 0 | 0 | 0 | 0 |
| XQXPVVBIMDBYFF-UHFFFAOYSA-N | O=C(O)Cc1ccc(O)cc1 | 0 | 1.2703153 | 0 | 0 | 0 | 0 | 0 | 0 | 0 | 0 | 0 | 0 | 0 | 0 | 0 |
| XGDZEDRBLVIUMX-UHFFFAOYSA-N | O=C(OC)Cc1ccc(O)cc1 | 0 | 1.7068218 | 0 | 0 | 0 | 0 | 0 | 0 | 0 | 0 | 0 | 0 | 0 | 0 | 0 |
| YEKIIDIQOZQXAX-UHFFFAOYSA-N | O=C(OC)c1c2C(=O)c3c(O)cc(C)cc3Oc2ccc1 | 0 | 2.6934354 | 0 | 0 | 0 | 0 | 0 | 0 | 0 | 0 | 0 | 0 | 0 | 0 | 0 |
| YJOWXMGENDGFDH-IAGOWNOFSA-N | O=C(O[C@@H]1[C@H](OC(=O)C)c2c3OC(=O)C=Cc3ccc2OC1(C)C)C | 0 | 2.9176257 | 0 | 0 | 0 | 0 | 0 | 0 | 0 | 0 | 0 | 0 | 0 | 0 | 0 |
| QNOQNIIIQHXDAV-CGICMKJESA-N | O=C(O[C@H]1C(C)(C)Oc2c(c(OC)c3C(=O)C=C(C)Oc3c2)C1)/C(=C\C)/C | 0 | 3.0218139 | 0 | 0 | 0 | 0 | 0 | 0 | 0 | 0 | 0 | 0 | 0 | 0 | 0 |
| RRHCDWLSHIIIIT-NVWZYQMFSA-N | O=C(O[C@H]1C(C)(C)Oc2c(c3OC(=O)C=Cc3cc2)C1)/C(=C\C)/C | 0 | 2.8609357 | 0 | 0 | 0 | 0 | 0 | 0 | 0 | 0 | 0 | 0 | 0 | 0 | 0 |
| PKHNHXIOSSYBJU-CQSZACIVSA-N | O=C(O[C@H]1C(C)(C)Oc2c(c3OC(=O)C=Cc3cc2)C1)C(C)C | 0 | 2.8741512 | 0 | 0 | 0 | 0 | 0 | 0 | 0 | 0 | 0 | 0 | 0 | 0 | 0 |
| HKXQUNNSKMWIKJ-DGCLKSJQSA-N | O=C1Oc2c3[C@@H](O)[C@@H](O)C(C)(C)Oc3ccc2C=C1 | 4.1101285 | 2.6848652 | 0 | 0 | 0 | 0 | 0 | 0 | 0 | 0 | 0 | 0 | 0 | 0 | 0 |
| ZMXFKFQNJTXZBC-UHFFFAOYSA-N | O=C(OC(C=C)c1cc(OC)c(OC(=O)C(C)C)cc1)C(C)C | 0 | 2.9615367 | 0 | 0 | 0 | 0 | 0 | 0 | 0 | 0 | 0 | 0 | 0 | 0 | 0 |
| WEZKIKHPDIXTOZ-LSJUHERESA-N | O=C(OC/C/1=C/C=C\C(C)CC[C@H]2C(=C)C(=O)O[C@@H]2C\1)C(C)C | 0 | 3.0199829 | 0 | 0 | 0 | 0 | 0 | 0 | 0 | 0 | 0 | 0 | 0 | 0 | 0 |
| AKXLMIWFRPOECM-QKSACFKYSA-N | O=C(O[C@@H]1C(=C)[C@@H]2[C@H]3OC(=O)C(=C)C3CCC(=C)[C@@H]2C1)C(C)C | 0 | 3.0003016 | 0 | 0 | 0 | 0 | 0 | 0 | 0 | 0 | 0 | 0 | 0 | 0 | 0 |
| PIGAIBAKKDXNOK-PAZXDIGFSA-N | O=C(O[C@@H]1C(=C)[C@@H]2[C@H]3OC(=O)C(=C)C3CCC(=C)[C@@H]2C1)CC(C)C | 0 | 3.0185438 | 0 | 0 | 0 | 0 | 0 | 0 | 0 | 0 | 0 | 0 | 0 | 0 | 0 |
| ZMQPXMBRSIEPJQ-UHFFFAOYSA-N | O=C(Oc1c(OC)cc(C(OC(=O)CC(C)C)C=C)cc1)C(C)C | 0 | 2.9806108 | 0 | 0 | 0 | 0 | 0 | 0 | 0 | 0 | 0 | 0 | 0 | 0 | 0 |
| NETSQGRTUNRXEO-FTKZKSHWSA-N | O=C1C(=C)C2[C@H](O1)[C@H]1C(=C)CC[C@H]1C(=C)CC2 | 0 | 2.2903279 | 0 | 0 | 0 | 0 | 0 | 0 | 0 | 0 | 0 | 0 | 0 | 0 | 0 |
| MMTZAJNKISZWFG-LUIAVFIISA-N | O=C1C(=C)[C@H]2[C@H](O1)C/C(/C)=C\C=C/C(C)CC2 | 0 | 2.3908978 | 0 | 0 | 0 | 0 | 0 | 0 | 0 | 0 | 0 | 0 | 0 | 0 | 0 |
| FZYRENRMDAPHEM-UHFFFAOYSA-N | O(C)c1c2c(O)c3C(=O)C(C)=C(C)Oc3cc2cc(OC)c1 | 0 | 2.8105031 | 0 | 0 | 0 | 0 | 0 | 0 | 0 | 0 | 0 | 0 | 0 | 0 | 0 |
| HFPQKJMLIONCGP-UHFFFAOYSA-N | O(C)c1c2c(O)c3C(=O)C=C(C)Oc3cc2cc(OC)c1 | 0 | 2.65488 | 0 | 0 | 0 | 0 | 0 | 0 | 0 | 0 | 0 | 0 | 0 | 0 | 0 |
| AHQXDHIRTWHABS-QMMMGPOBSA-N | O(C)c1c2c(O)c3C(=O)C[C@H](C)Oc3cc2cc(OC)c1 | 0 | 2.6882827 | 0 | 0 | 0 | 0 | 0 | 0 | 0 | 0 | 0 | 0 | 0 | 0 | 0 |
| DALYWWKMTCBVOR-IUCAKERBSA-N | O(C)c1c2c(O)c3C(=O)[C@@H](C)[C@H](C)Oc3cc2cc(OC)c1 | 0 | 2.8849833 | 0 | 0 | 0 | 0 | 0 | 0 | 0 | 0 | 0 | 0 | 0 | 0 | 0 |
| DALYWWKMTCBVOR-BDAKNGLRSA-N | O(C)c1c2c(O)c3C(=O)[C@H](C)[C@H](C)Oc3cc2cc(OC)c1 | 0 | 2.8849833 | 0 | 0 | 0 | 0 | 0 | 0 | 0 | 0 | 0 | 0 | 0 | 0 | 0 |
| DWGSEYZQYRDTJV-QMMMGPOBSA-N | O(C)c1c2c(c(O)c3C(=O)C[C@H](C)Oc13)c(OC)cc(OC)c2 | 0 | 2.936477 | 0 | 0 | 0 | 0 | 0 | 0 | 0 | 0 | 0 | 0 | 0 | 0 | 0 |
| KRQMXQZQIPIILU-IUCAKERBSA-N | O(C)c1c2c(c(O)c3C(=O)[C@@H](C)[C@H](C)Oc13)c(OC)cc(OC)c2 | 0 | 2.9877291 | 0 | 0 | 0 | 0 | 0 | 0 | 0 | 0 | 0 | 0 | 0 | 0 | 0 |
| KRQMXQZQIPIILU-BDAKNGLRSA-N | O(C)c1c2c(c(O)c3C(=O)[C@H](C)[C@H](C)Oc13)c(OC)cc(OC)c2 | 0 | 2.9877291 | 0 | 0 | 0 | 0 | 0 | 0 | 0 | 0 | 0 | 0 | 0 | 0 | 0 |
| CQIUKKVOEOPUDV-FSPLSTOPSA-N | O=C(O)C=1C(=O)C(C)=C2[C@@H](C)[C@H](C)OC=C2C=1O | 0 | 2.894399 | 0 | 0 | 0 | 0 | 0 | 0 | 0 | 0 | 0 | 0 | 0 | 0 | 0 |
| OIMXTYUHMBQQJM-GDJCSSNXSA-N | O=C1C=C2[C@@](C)(C3C(=C4[C@@](C)([C@@H]([C@@H](/C=C/[C@@H](C(C)C)C)C)CC4)CC3)C=C2)CC1 | 0 | 3.013985 | 0 | 0 | 0 | 0 | 0 | 0 | 0 | 0 | 0 | 0 | 0 | 0 | 0 |
| AWOGQCSIVCQXBT-VUEDXXQZSA-N | O(C)c1c(OC)ccc([C@H]2OC[C@@H]3[C@@H](c4cc5OCOc5cc4)OC[C@H]23)c1 | 0 | 2.9143842 | 0 | 0 | 0 | 0 | 0 | 0 | 0 | 0 | 0 | 0 | 0 | 0 | 0 |
| IWZXZFNLDPFUKQ-NAJRYUOPSA-N | O=C1O[C@H](CCC)C[C@@H](O)/C=C/CCC1 | 0 | 2.4760568 | 0 | 0 | 0 | 0 | 0 | 0 | 0 | 0 | 0 | 0 | 0 | 0 | 0 |
| CPDBOUQRDMUUIN-UHFFFAOYSA-N | O=C(O)C(=C)C1CC(=O)C=2C(C)(C(C)CCC=2)C1 | 0 | 2.8316484 | 0 | 0 | 0 | 0 | 0 | 0 | 0 | 0 | 0 | 0 | 0 | 0 | 0 |
| PEYLVIRXSYMEHY-FHXLMMPLSA-N | OC[C@]1(C)[C@H]2C1[C@@H]1[C@H](C)CC[C@@H]1[C@](O)(C)CC2 | 0 | 2.7268944 | 0 | 0 | 0 | 0 | 0 | 0 | 0 | 0 | 0 | 0 | 0 | 0 | 0 |
| GLEWZPUZTYZURE-WYMLVPIESA-N | O=C(/C=C/CCCCCCCCCCCCc1cc2OCOc2cc1)C | 0 | 2.976983 | 0 | 0 | 0 | 0 | 0 | 0 | 0 | 0 | 0 | 0 | 0 | 0 | 0 |
| MRIJVCUMMLLFRJ-GHRIWEEISA-N | O=C(/C=C/CCCCCCCCCCCCc1ccccc1)C | 0 | 2.8463665 | 0 | 0 | 0 | 0 | 0 | 0 | 0 | 0 | 0 | 0 | 0 | 0 | 0 |
| HYOOYGBSEUETML-ZRDIBKRKSA-N | O=C(/C=C/CCCCCCCCCCc1cc2OCOc2cc1)C | 0 | 2.9117895 | 0 | 0 | 0 | 0 | 0 | 0 | 0 | 0 | 0 | 0 | 0 | 0 | 0 |
| XSCQBXUNBGFDEK-ACCUITESSA-N | O=C(CCCCCCCCCC/C=C/c1cc2OCOc2cc1)C | 0 | 2.90863 | 0 | 0 | 0 | 0 | 0 | 0 | 0 | 0 | 0 | 0 | 0 | 0 | 0 |
| RSLLRVSPUBUTHT-FYWRMAATSA-N | O=C(CCCCCCCCCCCC/C=C/c1cc2OCOc2cc1)C | 0 | 2.9658018 | 0 | 0 | 0 | 0 | 0 | 0 | 0 | 0 | 0 | 0 | 0 | 0 | 0 |
| OHKYJRJHRQFHDW-UHFFFAOYSA-N | O=C(CCCCCCCCCCCCCCc1cc2OCOc2cc1)C | 0 | 3.0004931 | 0 | 0 | 0 | 0 | 0 | 0 | 0 | 0 | 0 | 0 | 0 | 0 | 0 |
| NWTRBPHNLLSBBD-UHFFFAOYSA-N | O=C(CCCCCCCCCCCCCCc1ccccc1)C | 0 | 2.8761563 | 0 | 0 | 0 | 0 | 0 | 0 | 0 | 0 | 0 | 0 | 0 | 0 | 0 |
| WESJGASUMOLVBD-UHFFFAOYSA-N | O=C(CCCCCCCCCCCCc1cc2OCOc2cc1)C | 0 | 2.9414954 | 0 | 0 | 0 | 0 | 0 | 0 | 0 | 0 | 0 | 0 | 0 | 0 | 0 |
| XKYCRRQKLLZDJB-UHFFFAOYSA-N | O=C(CCCCCCCCCCc1cc2OCOc2cc1)C | 0 | 2.8684918 | 0 | 0 | 0 | 0 | 0 | 0 | 0 | 0 | 0 | 0 | 0 | 0 | 0 |
| KFHRRMKPUDPLGO-UHFFFAOYSA-N | O=C(OC)CC1OC(CCCCCCCCCCCCC)CCC1 | 0 | 3.0215676 | 0 | 0 | 0 | 0 | 0 | 0 | 0 | 0 | 0 | 0 | 0 | 0 | 0 |
| YGQMDFMXPBTIRS-UHFFFAOYSA-N | O=C(OC)CC1OC(CCCCCCCCCCCCCC)CC1 | 0 | 3.0019138 | 0 | 0 | 0 | 0 | 0 | 0 | 0 | 0 | 0 | 0 | 0 | 0 | 0 |
| UYTVHKZGZWOESS-UHFFFAOYSA-N | O=C(OC)CC1OC(CCCCCCCCCCc2ccccc2)CCC1 | 0 | 2.9846725 | 0 | 0 | 0 | 0 | 0 | 0 | 0 | 0 | 0 | 0 | 0 | 0 | 0 |
| SBMILRCXQPFDME-UHFFFAOYSA-N | O(C)C=1C(=O)C2=C(C(=O)C=1)c1c(cc(O)cc1)CC2 | 0 | 2.7485736 | 0 | 0 | 0 | 0 | 0 | 0 | 0 | 0 | 0 | 0 | 0 | 0 | 0 |
| HMYHQSWJLABPMD-UHFFFAOYSA-N | O(C)C=1C2(C(=O)c3c(O)cccc3CC2)CC(=O)C=1 | 0 | 2.5519175 | 0 | 0 | 0 | 0 | 0 | 0 | 0 | 0 | 0 | 0 | 0 | 0 | 0 |
| RDKDIPDDUFMMMT-UHFFFAOYSA-N | O(C)c1cc(O)c2-c3c(cc(O)cc3)CCc2c1 | 0 | 2.5789757 | 0 | 0 | 0 | 0 | 0 | 0 | 0 | 0 | 0 | 0 | 0 | 0 | 0 |
| HCRRYKYLZWKBBM-UHFFFAOYSA-N | O(C)c1c(OC)ccc(CCc2cc(OC)cc(OC)c2)c1 | 0 | 2.6561562 | 0 | 0 | 0 | 0 | 0 | 0 | 0 | 0 | 0 | 0 | 0 | 0 | 0 |
| FDJURJXPMJANDW-UHFFFAOYSA-N | O(C)c1cc(O)cc(CCc2cc(OC)ccc2)c1 | 0 | 2.5878022 | 0 | 0 | 0 | 0 | 0 | 0 | 0 | 0 | 0 | 0 | 0 | 0 | 0 |
| NSBYGUHECONSDC-UHFFFAOYSA-N | O(C)c1cc(O)cc(CCc2ccc(O)cc2)c1 | 0 | 2.5537467 | 0 | 0 | 0 | 0 | 0 | 0 | 0 | 0 | 0 | 0 | 0 | 0 | 0 |
| KZNIFHPLKGYRTM-UHFFFAOYSA-N | O=C1c2c(O)cc(O)cc2OC(c2ccc(O)cc2)=C1 | 0 | 2.7720865 | 0 | 0 | 0 | 0 | 0 | 0 | 0 | 0 | 0 | 0 | 0 | 0 | 0 |
| UMZJVKFVOMTAFO-UHFFFAOYSA-N | Oc1cc(O)cc(CCc2cc(O)ccc2)c1 | 0 | 2.4632931 | 0 | 0 | 0 | 0 | 0 | 0 | 0 | 0 | 0 | 0 | 0 | 0 | 0 |
| HITJFUSPLYBJPE-UHFFFAOYSA-N | Oc1cc(O)cc(CCc2ccc(O)cc2)c1 | 0 | 2.5074361 | 0 | 0 | 0 | 0 | 0 | 0 | 0 | 0 | 0 | 0 | 0 | 0 | 0 |
| LHLJANTYAXQUPZ-UHFFFAOYSA-N | O=C(OCC(=O)c1c(O)cc(C)cc1)C | 0 | 2.493969 | 0 | 0 | 0 | 0 | 0 | 0 | 0 | 0 | 0 | 0 | 0 | 0 | 0 |
| YCOFRPYSZKIPBQ-UHFFFAOYSA-N | O(C)C=1C(O)(C(=C)C)OC(=O)C=1 | 2.6205964 | 1.6916467 | 0 | 0 | 0 | 0 | 0 | 0 | 0 | 0 | 0 | 0 | 0 | 0 | 0 |
| YFRGOWXYOVAUBU-NZFNHWASSA-N | O=C1C(C)=C2C(O1)=CC1=C(O)C(=O)C[C@H](C)[C@@]1(C)C2 | 5.2784969 | 2.6689821 | 0 | 0 | 0 | 0 | 0 | 0 | 0 | 0 | 0 | 0 | 0 | 0 | 0 |
| YLHAQKVIXLQIFW-UHFFFAOYSA-N | O=C(OCc1c(OC)cccc1)c1c(O)cccc1 | 0 | 2.6413076 | 0 | 0 | 0 | 0 | 0 | 0 | 0 | 0 | 0 | 0 | 0 | 0 | 0 |
| PIWSYQREANNHKN-UHFFFAOYSA-N | O=C(OCc1cc(O)ccc1)c1c(OC)cccc1OC | 0 | 2.6078859 | 0 | 0 | 0 | 0 | 0 | 0 | 0 | 0 | 0 | 0 | 0 | 0 | 0 |
| DGYWXQIMCBTTQH-UHFFFAOYSA-N | O=C(OCc1cc(OC)ccc1)c1c(OC)cccc1O | 0 | 2.6310487 | 0 | 0 | 0 | 0 | 0 | 0 | 0 | 0 | 0 | 0 | 0 | 0 | 0 |
| DZDQHOAIKWCQDB-UHFFFAOYSA-N | O=C(OCc1cc(OC)ccc1)c1c(OC)cccc1OC | 0 | 2.6278851 | 0 | 0 | 0 | 0 | 0 | 0 | 0 | 0 | 0 | 0 | 0 | 0 | 0 |
| HKRGBDMRFLWKMB-UHFFFAOYSA-N | O=C(OCc1ccccc1)c1c(OC)c(OC)ccc1O | 0 | 2.7603516 | 0 | 0 | 0 | 0 | 0 | 0 | 0 | 0 | 0 | 0 | 0 | 0 | 0 |
| WKTUEASUPBAIPX-UHFFFAOYSA-N | O=C(OCc1ccccc1)c1c(OC)c(OC)ccc1OC | 0 | 2.7554258 | 0 | 0 | 0 | 0 | 0 | 0 | 0 | 0 | 0 | 0 | 0 | 0 | 0 |
| CGNJMCLXIMWKDY-UHFFFAOYSA-N | O=C(OCc1ccccc1)c1c(OC)cccc1O | 0 | 2.5689268 | 0 | 0 | 0 | 0 | 0 | 0 | 0 | 0 | 0 | 0 | 0 | 0 | 0 |
| PKIYLOACOOWBCU-UHFFFAOYSA-N | O=C(OCc1ccccc1)c1c(OC)cccc1OC | 0 | 2.5727151 | 0 | 0 | 0 | 0 | 0 | 0 | 0 | 0 | 0 | 0 | 0 | 0 | 0 |
| OKWRDLQBKAOJNC-GFCCVEGCSA-N | O(C)c1c2C(=O)O[C@H](C)CCCCCCCc2cc(O)c1 | 0 | 2.7821736 | 0 | 0 | 0 | 0 | 0 | 0 | 0 | 0 | 0 | 0 | 0 | 0 | 0 |
| UGQMRVRMYYASKQ-KQYNXXCUSA-N | O=C1NC=Nc2n([C@H]3[C@H](O)[C@H](O)[C@@H](CO)O3)cnc12 | 4.8473013 | 2.7001898 | 0 | 0 | 0 | 0 | 0 | 0 | 0 | 0 | 0 | 0 | 0 | 0 | 0 |
| YCCILVSKPBXVIP-UHFFFAOYSA-N | OCCc1ccc(O)cc1 | 0 | 1.2789197 | 0 | 0 | 0 | 0 | 0 | 0 | 0 | 0 | 0 | 0 | 0 | 0 | 0 |
| IGWDEVSBEKYORK-UHFFFAOYSA-N | O(CC=C(C)C)c1c2c(occ2)cc2OC(=O)C=Cc12 | 0 | 2.5764913 | 0 | 0 | 0 | 0 | 0 | 0 | 0 | 0 | 0 | 0 | 0 | 0 | 0 |
| MBRLOUHOWLUMFF-UHFFFAOYSA-N | O(C)c1c(CC=C(C)C)c2OC(=O)C=Cc2cc1 | 0 | 2.5715729 | 0 | 0 | 0 | 0 | 0 | 0 | 0 | 0 | 0 | 0 | 0 | 0 | 0 |
| QXKHYNVANLEOEG-UHFFFAOYSA-N | O(C)c1c2occc2cc2c1OC(=O)C=C2 | 0 | 2.065776 | 0 | 0 | 0 | 0 | 0 | 0 | 0 | 0 | 0 | 0 | 0 | 0 | 0 |
| ORHBXUUXSCNDEV-UHFFFAOYSA-N | O=C1Oc2c(ccc(O)c2)C=C1 | 0 | 1.2898956 | 0 | 0 | 0 | 0 | 0 | 0 | 0 | 0 | 0 | 0 | 0 | 0 | 0 |
| BGEBZHIAGXMEMV-UHFFFAOYSA-N | O(C)c1c2c(occ2)cc2OC(=O)C=Cc12 | 0 | 2.0654487 | 0 | 0 | 0 | 0 | 0 | 0 | 0 | 0 | 0 | 0 | 0 | 0 | 0 |
| SYTYLPHCLSSCOJ-UHFFFAOYSA-N | O(C)c1c(O)cc2c(OC(=O)C=C2)c1 | 3.0254244 | 1.95986 | 0 | 0 | 0 | 0 | 0 | 0 | 0 | 0 | 0 | 0 | 0 | 0 | 0 |
| RODXRVNMMDRFIK-UHFFFAOYSA-N | O(C)c1c(O)cc2OC(=O)C=Cc2c1 | 3.0965587 | 2.0748424 | 0 | 0 | 0 | 0 | 0 | 0 | 0 | 0 | 0 | 0 | 0 | 0 | 0 |
| IQUUXFOZWKSLBQ-UHFFFAOYSA-N | O(C)c1c(OC)c2c3c(O)cccc3c(OC)cc2cc1OC | 0 | 2.7836513 | 0 | 0 | 0 | 0 | 0 | 0 | 0 | 0 | 0 | 0 | 0 | 0 | 0 |
| ILUJQPXNXACGAN-UHFFFAOYSA-N | O=C(O)c1c(OC)cccc1 | 0 | 1.1437119 | 0 | 0 | 0 | 0 | 0 | 0 | 0 | 0 | 0 | 0 | 0 | 0 | 0 |
| AAUQLHHARJUJEH-UHFFFAOYSA-N | O=C(O)c1c(OC)cccc1O | 0 | 1.5815144 | 0 | 0 | 0 | 0 | 0 | 0 | 0 | 0 | 0 | 0 | 0 | 0 | 0 |
| NGSWKAQJJWESNS-ZZXKWVIFSA-N | O=C(O)/C=C/c1ccc(O)cc1 | 0 | 1.3124186 | 0 | 0 | 0 | 0 | 0 | 0 | 0 | 0 | 0 | 0 | 0 | 0 | 0 |
| GZGGCZADGIBRHT-DQLDELGASA-N | Clc1c(Cl)cc2c([nH]c3C(C)(C)[C@H]4[C@@]5(NC(=O)[C@@]6(N(C5)CCC6)C4)Cc23)c1 | 0.3082905 | 2.8350884 | 0 | 0 | 0 | 0 | 0 | 0 | 0 | 0 | 0 | 0 | 0 | 0 | 0 |
| DXPVAKSJZFQGSS-DQLDELGASA-N | Clc1cc2[nH]c3C(C)(C)[C@H]4[C@@]5(NC(=O)[C@@]6(N(C5)CCC6)C4)Cc3c2cc1 | 0.3273582 | 2.826343 | 0 | 0 | 0 | 0 | 0 | 0 | 0 | 0 | 0 | 0 | 0 | 0 | 0 |
| VCMZMLWIPPPAOG-UHFFFAOYSA-N | O=C(CCC(=O)c1c(O)cc(C)cc1)c1c(O)cc(C)cc1 | 0 | 2.9271257 | 0 | 0 | 0 | 0 | 0 | 0 | 0 | 0 | 0 | 0 | 0 | 0 | 0 |
| UUEIVUJSJLKAAM-SDQBBNPISA-N | O=C(Oc1c(C(C)C)ccc(C)c1)/C(=C\C)/C | 0 | 2.5663893 | 0 | 0 | 0 | 0 | 0 | 0 | 0 | 0 | 0 | 0 | 0 | 0 | 0 |
| XRTYDFQPBORLIK-XGICHPGQSA-N | O=C(Oc1c(C2(COC(=O)C)OC2)ccc(C)c1)/C(=C\C)/C | 0 | 2.8785745 | 0 | 0 | 0 | 0 | 0 | 0 | 0 | 0 | 0 | 0 | 0 | 0 | 0 |
| FGMNYSJXCIZEOU-WAYWQWQTSA-N | O=C(Oc1c(C(C)C)ccc(C)c1)/C=C\C | 0 | 2.4201675 | 0 | 0 | 0 | 0 | 0 | 0 | 0 | 0 | 0 | 0 | 0 | 0 | 0 |
| LONAZUBXVNGNKH-WZUFQYTHSA-N | O=C(Oc1c(C2(C(=O)C)OC2)ccc(C)c1)/C(=C\C)/C | 0 | 2.7971667 | 0 | 0 | 0 | 0 | 0 | 0 | 0 | 0 | 0 | 0 | 0 | 0 | 0 |
| MGSRCZKZVOBKFT-UHFFFAOYSA-N | Oc1c(C(C)C)ccc(C)c1 | 0 | 1.3810212 | 0 | 0 | 0 | 0 | 0 | 0 | 0 | 0 | 0 | 0 | 0 | 0 | 0 |
| BNWJOHGLIBDBOB-UHFFFAOYSA-N | O(C)c1c2OCOc2cc(CC=C)c1 | 0 | 1.9400984 | 0 | 0 | 0 | 0 | 0 | 0 | 0 | 0 | 0 | 0 | 0 | 0 | 0 |
| KSEBMYQBYZTDHS-HWKANZROSA-N | O=C(O)/C=C/c1cc(OC)c(O)cc1 | 3.0980471 | 2.1920996 | 0 | 0 | 0 | 0 | 0 | 0 | 0 | 0 | 0 | 0 | 0 | 0 | 0 |
| IQVQXVFMNOFTMU-DHZHZOJOSA-N | O=C1O/C(=C/CCC)/C2=C1C=CCC2 | 0 | 2.0372838 | 0 | 0 | 0 | 0 | 0 | 0 | 0 | 0 | 0 | 0 | 0 | 0 | 0 |
| WMBOCUXXNSOQHM-DHZHZOJOSA-N | O=C1O/C(=C/CCC)/c2c1cccc2 | 0 | 1.9807463 | 0 | 0 | 0 | 0 | 0 | 0 | 0 | 0 | 0 | 0 | 0 | 0 | 0 |
| ZGPJMFUBGMSEDV-CORQQEAWSA-N | O=C(O[C@@H]1[C@]2(C)[C@@H](C)CCC=C2C(=O)c2occ(C)c12)/C(=C\C)/C | 0 | 2.9398145 | 0 | 0 | 0 | 0 | 0 | 0 | 0 | 0 | 0 | 0 | 0 | 0 | 0 |
| PUBUQGJOCRORHJ-TWSVYZDPSA-N | O=C(O[C@@H]1[C@]2(C)[C@@H](C)CCC=C2C(=O)c2occ(C)c12)[C@H](CC)C | 0 | 2.9679225 | 0 | 0 | 0 | 0 | 0 | 0 | 0 | 0 | 0 | 0 | 0 | 0 | 0 |
| ZGPJMFUBGMSEDV-OLQHVUIGSA-N | O=C(O[C@H]1[C@]2(C)[C@@H](C)CCC=C2C(=O)c2occ(C)c12)/C(=C\C)/C | 0 | 2.9398145 | 0 | 0 | 0 | 0 | 0 | 0 | 0 | 0 | 0 | 0 | 0 | 0 | 0 |
| VWUPQZXUHCYORA-ZEQRYJAYSA-N | O=C(O[C@H]1[C@]2(C)[C@@H](C)C[C@@H](O)C=C2C(=O)c2occc12)/C(=C\C)/C | 0 | 3.011359 | 0 | 0 | 0 | 0 | 0 | 0 | 0 | 0 | 0 | 0 | 0 | 0 | 0 |
| VNNQNPHIASWXBS-AYVTZFPOSA-N | O=C1c2occ(C)c2[C@](O)(C)C=2[C@@H](C)CCCC1=2 | 0 | 2.6993367 | 0 | 0 | 0 | 0 | 0 | 0 | 0 | 0 | 0 | 0 | 0 | 0 | 0 |
| JPMYFOBNRRGFNO-UHFFFAOYSA-N | O(C)c1cc(O)c2C(=O)C=C(c3ccc(O)cc3)Oc2c1 | 0 | 2.7659948 | 0 | 0 | 0 | 0 | 0 | 0 | 0 | 0 | 0 | 0 | 0 | 0 | 0 |
| GHZRKUIAWAOWRH-UHFFFAOYSA-N | O=C(C)c1c(O)c2c(OC(C)(C)C=C2)cc1 | 0 | 2.5806982 | 0 | 0 | 0 | 0 | 0 | 0 | 0 | 0 | 0 | 0 | 0 | 0 | 0 |
| NPNUFJAVOOONJE-UHFFFAOYSA-N | C=C1C2C(C(C)(C)C2)CCC(C)=CCC1 | 0 | 2.3236818 | 0 | 0 | 0 | 0 | 0 | 0 | 0 | 0 | 0 | 0 | 0 | 0 | 0 |
| NVEQFIOZRFFVFW-UHFFFAOYSA-N | C=C1C2C(C(C)(C)C2)CCC2(C)OC2CC1 | 0 | 2.6269794 | 0 | 0 | 0 | 0 | 0 | 0 | 0 | 0 | 0 | 0 | 0 | 0 | 0 |
| MFIBJICNTUTPCP-UHFFFAOYSA-N | O(C)c1c2C(=O)OC=C3C(OC)=CC(=O)c(c(C)c1)c23 | 0 | 2.7772651 | 0 | 0 | 0 | 0 | 0 | 0 | 0 | 0 | 0 | 0 | 0 | 0 | 0 |
| HBTQKFCMVAYBOL-UHFFFAOYSA-N | O(C)c1c2c(C)cc(OC)c3C(=O)OC(OC)c(c(O)c1)c23 | 0 | 2.8532636 | 0 | 0 | 0 | 0 | 0 | 0 | 0 | 0 | 0 | 0 | 0 | 0 | 0 |
| IYNJGYCVIYBFBS-UHFFFAOYSA-N | O=C1c2c(O)cccc2C(=O)c2c(O)cc(C)cc12 | 0 | 2.5930767 | 0 | 0 | 0 | 0 | 0 | 0 | 0 | 0 | 0 | 0 | 0 | 0 | 0 |
| VYQXIUVIYICVCM-UHFFFAOYSA-N | O(C)c1cc(O)cc(CCc2cc(O)ccc2)c1 | 0 | 2.475314 | 0 | 0 | 0 | 0 | 0 | 0 | 0 | 0 | 0 | 0 | 0 | 0 | 0 |
| MSHFRERJPWKJFX-UHFFFAOYSA-N | O(C)c1ccc(CO)cc1 | 0 | 1.2537113 | 0 | 0 | 0 | 0 | 0 | 0 | 0 | 0 | 0 | 0 | 0 | 0 | 0 |
| POBIEWKRMCMVFH-PKNBQFBNSA-N | O=C(/C=C/C(O)(C)C)C(CCCC(O)(C=C)C)C | 0 | 2.8342205 | 0 | 0 | 0 | 0 | 0 | 0 | 0 | 0 | 0 | 0 | 0 | 0 | 0 |
| DZESPMMROLVXTM-OKPQQOGWSA-N | O=C1C(=C)C2[C@@H](O1)[C@@H]1[C@](O)(C)C=CC(=O)[C@@]1(C)CC2 | 0 | 2.8562829 | 0 | 0 | 0 | 0 | 0 | 0 | 0 | 0 | 0 | 0 | 0 | 0 | 0 |
| PENSQRMNZZWMGV-UHFFFAOYSA-N | O(C)c1c(C=2C(=O)Oc3c(cc4c(occ4)c3)C=2)cc2OCOc2c1 | 0 | 2.8316796 | 0 | 0 | 0 | 0 | 0 | 0 | 0 | 0 | 0 | 0 | 0 | 0 | 0 |
| RAJDDCCSNZAPCH-ZWKOTPCHSA-N | O=C1[C@H]2[C@@H](Oc3c1cc1c(occ1)c3)COc1c2cc2OCOc2c1 | 0 | 2.9221644 | 0 | 0 | 0 | 0 | 0 | 0 | 0 | 0 | 0 | 0 | 0 | 0 | 0 |
| CIGSWLXZMSXAAE-ZCFIWIBFSA-N | O(C)c1c(C)c2[C@H](C)C(=C)OC(=O)c2c(O)c1 | 0 | 2.5199042 | 0 | 0 | 0 | 0 | 0 | 0 | 0 | 0 | 0 | 0 | 0 | 0 | 0 |
| MSUXGUUSNDNHFC-UHFFFAOYSA-N | O=C1c2c(C)c(O)cc(O)c2C(=O)CC1 | 0 | 2.4470636 | 0 | 0 | 0 | 0 | 0 | 0 | 0 | 0 | 0 | 0 | 0 | 0 | 0 |
| ZXYYTDCENDYKBR-ZETCQYMHSA-N | O=C1c2c(O)cccc2[C@@H](O)CC1 | 0 | 2.0383146 | 0 | 0 | 0 | 0 | 0 | 0 | 0 | 0 | 0 | 0 | 0 | 0 | 0 |
| DANYIYRPLHHOCZ-UHFFFAOYSA-N | O(C)c1ccc(C=2Oc3c(c(O)cc(O)c3)C(=O)C=2)cc1 | 0 | 2.7659948 | 0 | 0 | 0 | 0 | 0 | 0 | 0 | 0 | 0 | 0 | 0 | 0 | 0 |
| NWZKOCPFFSDCBI-OCVOHJKQSA-N | O=C1/C(=C(\C)/C)/[C@H](O)[C@]2(C)[C@@H](C)[C@H](O)CCC2=C1 | 0 | 2.7412807 | 0 | 0 | 0 | 0 | 0 | 0 | 0 | 0 | 0 | 0 | 0 | 0 | 0 |
| TVDMUSYVWJLIDK-PSOPSSQASA-N | O=C1C(=C(C)C)C[C@]2(C)[C@@H](C)[C@H](O)CCC2=C1 | 0 | 2.6546797 | 0 | 0 | 0 | 0 | 0 | 0 | 0 | 0 | 0 | 0 | 0 | 0 | 0 |
| PCBDXYONDOCJPR-VAEXESPGSA-N | O=C1C2(C(=C)C)O[C@@H]2[C@]2(C)[C@@H](C)[C@H](O)CCC2=C1 | 0 | 2.7086435 | 0 | 0 | 0 | 0 | 0 | 0 | 0 | 0 | 0 | 0 | 0 | 0 | 0 |
| AIVUQNKTJDAYQX-RNJXMRFFSA-N | O(C)C=1[C@H](O)[C@]2(C)O[C@@H]2C(=O)C=1 | 2.6501259 | 1.6928583 | 0 | 0 | 0 | 0 | 0 | 0 | 0 | 0 | 0 | 0 | 0 | 0 | 0 |
| AYIDXPPINFIJKW-ZETCQYMHSA-N | O(C)c1c2C(=O)O[C@@H](C)Cc2ccc1 | 0 | 2.142368 | 0 | 0 | 0 | 0 | 0 | 0 | 0 | 0 | 0 | 0 | 0 | 0 | 0 |
| GUAACJNCCYVZCM-MELADBBJSA-N | O(C)C=1[C@H](O)[C@@](O)(C)[C@@H](Nc2ccccc2)C(=O)C=1 | 4.3238523 | 2.7960773 | 0 | 0 | 0 | 0 | 0 | 0 | 0 | 0 | 0 | 0 | 0 | 0 | 0 |
| AIVUQNKTJDAYQX-BIIVOSGPSA-N | O(C)C=1[C@H](O)[C@@]2(C)O[C@H]2C(=O)C=1 | 2.6501259 | 1.6928583 | 0 | 0 | 0 | 0 | 0 | 0 | 0 | 0 | 0 | 0 | 0 | 0 | 0 |
| YYWUABJYAOCACI-UHFFFAOYSA-N | O=C1N(C)C(Cc2ccccc2)C=2N(C(=O)c3c(N=2)cccc3)c2c1cccc2 | 0 | 2.8821236 | 0 | 0 | 0 | 0 | 0 | 0 | 0 | 0 | 0 | 0 | 0 | 0 | 0 |
| VMRNXWXXUDWZEX-UHFFFAOYSA-N | O=C1NC(=O)C=2C(=O)c3c(N(C)C1=2)cccc3 | 1.0027611 | 2.5516814 | 0 | 0 | 0 | 0 | 0 | 0 | 0 | 0 | 0 | 0 | 0 | 0 | 0 |
| YCGFIOZHNUQYSK-UHFFFAOYSA-N | O=C1NC(C(C)C)C=2N(C)c3c(C(=O)C1=2)cccc3 | 0.7326779 | 2.5984869 | 0 | 0 | 0 | 0 | 0 | 0 | 0 | 0 | 0 | 0 | 0 | 0 | 0 |
| FLHQAMWKNPOTDV-ZANVPECISA-N | O=C1N[C@@H]([C@H](CC)C)C=2N(C)c3c(C(=O)C1=2)cccc3 | 0.70039 | 2.7459707 | 0 | 0 | 0 | 0 | 0 | 0 | 0 | 0 | 0 | 0 | 0 | 0 | 0 |
| FLHQAMWKNPOTDV-TVQRCGJNSA-N | O=C1N[C@H]([C@H](CC)C)C=2N(C)c3c(C(=O)C1=2)cccc3 | 0.70039 | 2.7459707 | 0 | 0 | 0 | 0 | 0 | 0 | 0 | 0 | 0 | 0 | 0 | 0 | 0 |
| PLSSEPIRACGCBO-PFFFPCNUSA-N | O=C1C(=C)[C@H]2[C@H](O1)[C@H]1C(C)=CC[C@@H](O)[C@]1(C)CC2 | 0 | 2.7866487 | 0 | 0 | 0 | 0 | 0 | 0 | 0 | 0 | 0 | 0 | 0 | 0 | 0 |
| WHBSVFFDUQPHNS-UHFFFAOYSA-N | Clc1c(O)c2c(cc1)C=CC(=O)OC2 | 0.317348 | 1.5375997 | 0 | 0 | 0 | 0 | 0 | 0 | 0 | 0 | 0 | 0 | 0 | 0 | 0 |
| AMZNYVFIWCPUAY-UHFFFAOYSA-N | O=C(C)c1c(O)c(C)c(O)c(C)c1 | 0 | 1.9767162 | 0 | 0 | 0 | 0 | 0 | 0 | 0 | 0 | 0 | 0 | 0 | 0 | 0 |
| OMBMYWYFYLOVPT-UHFFFAOYSA-N | O=C1OCc2c(O)cccc2C=C1 | 0 | 1.5181637 | 0 | 0 | 0 | 0 | 0 | 0 | 0 | 0 | 0 | 0 | 0 | 0 | 0 |
| MWZYYACVPIJBPD-SIWOTSRUSA-N | O=C1[C@]2([C@@H](O)C(=C)O1)[C@@H](O)C(=O)C=C[C@H]2C | 5.0400506 | 2.5870186 | 0 | 0 | 0 | 0 | 0 | 0 | 0 | 0 | 0 | 0 | 0 | 0 | 0 |
| ANHHAPDWNRZSRR-YZLKNSBCSA-N | O=C1[C@]2([C@H](O)C(=C)O1)[C@@H](O)C(=O)CC[C@H]2C | 4.7836953 | 2.6717891 | 0 | 0 | 0 | 0 | 0 | 0 | 0 | 0 | 0 | 0 | 0 | 0 | 0 |
| VWNBIHJYLBWFKS-UPZJHPNMSA-N | O[C@@H]([C@@H](O)C)C[C@H]1OCc2c(O)cccc12 | 4.9933761 | 2.5505524 | 0 | 0 | 0 | 0 | 0 | 0 | 0 | 0 | 0 | 0 | 0 | 0 | 0 |
| ZNXZQBCPPUYJBI-UHFFFAOYSA-N | O(C)c1c(C)c2C(=O)C(OC)=CC(=O)c2c(O)c1 | 0 | 2.7609826 | 0 | 0 | 0 | 0 | 0 | 0 | 0 | 0 | 0 | 0 | 0 | 0 | 0 |
| OZEDYANFTHNHQG-UHFFFAOYSA-N | O(C)c1c2C(=O)OC=C3C(OC)=CC(=O)c(c(O)c1)c23 | 0 | 2.8081791 | 0 | 0 | 0 | 0 | 0 | 0 | 0 | 0 | 0 | 0 | 0 | 0 | 0 |
| GUAFOGOEJLSQBT-UHFFFAOYSA-N | O(C)c1c(OC)cc2c(OC(=O)C=C2)c1 | 0 | 2.0834306 | 0 | 0 | 0 | 0 | 0 | 0 | 0 | 0 | 0 | 0 | 0 | 0 | 0 |
| LZERJKGWTQYMBB-UHFFFAOYSA-N | O(C)c1cc(O)c2C(=O)C=C(c3ccc(OC)cc3)Oc2c1 | 0 | 2.7606818 | 0 | 0 | 0 | 0 | 0 | 0 | 0 | 0 | 0 | 0 | 0 | 0 | 0 |
| LIIALPBMIOVAHH-UHFFFAOYSA-N | O(C)c1cc2OC(=O)C=Cc2cc1 | 0 | 1.5717322 | 0 | 0 | 0 | 0 | 0 | 0 | 0 | 0 | 0 | 0 | 0 | 0 | 0 |
| WJUFSDZVCOTFON-UHFFFAOYSA-N | O=Cc1cc(OC)c(OC)cc1 | 0 | 1.4905257 | 0 | 0 | 0 | 0 | 0 | 0 | 0 | 0 | 0 | 0 | 0 | 0 | 0 |
| JSYQPAIDLVPBDA-JWUPAISJSA-N | O=C1C(O)=C(C)[C@@H]2C(C)(C)[C@@H]3[C@@]1([C@H](C)CC3)C2=O | 4.9302643 | 2.6906691 | 0 | 0 | 0 | 0 | 0 | 0 | 0 | 0 | 0 | 0 | 0 | 0 | 0 |
| JSYQPAIDLVPBDA-RAIZXLCNSA-N | O=C1C(O)=C(C)[C@H]2C(C)(C)[C@H]3[C@]1([C@H](C)CC3)C2=O | 4.9302643 | 2.6906691 | 0 | 0 | 0 | 0 | 0 | 0 | 0 | 0 | 0 | 0 | 0 | 0 | 0 |
| JZXORCGMYQZBBQ-SNVBAGLBSA-N | O=C1C(O)=C([C@@H](CCC=C(C)C)C)C(=O)C=C1C | 5.6475132 | 2.6097414 | 0 | 0 | 0 | 0 | 0 | 0 | 0 | 0 | 0 | 0 | 0 | 0 | 0 |
| QQQCWVDPMPFUGF-UHFFFAOYSA-N | O(C)c1c2C(=O)CC(c3ccccc3)Oc2cc(O)c1 | 0 | 2.6846329 | 0 | 0 | 0 | 0 | 0 | 0 | 0 | 0 | 0 | 0 | 0 | 0 | 0 |
| ORJDDOBAOGKRJV-UHFFFAOYSA-N | O(C)c1cc(O)c2C(=O)CC(c3ccccc3)Oc2c1 | 0 | 2.6701942 | 0 | 0 | 0 | 0 | 0 | 0 | 0 | 0 | 0 | 0 | 0 | 0 | 0 |
| RTIXKCRFFJGDFG-UHFFFAOYSA-N | O=C1c2c(O)cc(O)cc2OC(c2ccccc2)=C1 | 0 | 2.610088 | 0 | 0 | 0 | 0 | 0 | 0 | 0 | 0 | 0 | 0 | 0 | 0 | 0 |
| URFCJEUYXNAHFI-UHFFFAOYSA-N | O=C1c2c(O)cc(O)cc2OC(c2ccccc2)C1 | 0 | 2.6466126 | 0 | 0 | 0 | 0 | 0 | 0 | 0 | 0 | 0 | 0 | 0 | 0 | 0 |
