# Supplementary material for: Application 2D Descriptors and Artificial Neural Networks for Beta-Glucosidase Inhibitors Screening
Source: Molecules. 2020 Dec 15;25(24):5942. doi: 10.3390/molecules25245942 (PMC7765417; doi:10.3390/molecules25245942)
Supplement: Supplementary file 1 [file molecules-25-05942-s001.zip › molecules-1025529-SI/molecules-1025529-SI.pdf]

Table S1 The dataset used for model development and validation obtained from ChEMBL database (https://www.ebi.ac.uk/chembl/). In case of more than IC50 values the mean was taken into account. The classification threshold was 50 μM. Class 0 denotes weakly active or non-active compounds, while Class 1, active ones.

No.	Compound ID	SMILES	IC50 [μM]	ChEMBL Assay ID	Class
1	CHEMBL3736317	Cl.CCCCCCN[C@@H]1C=C[C@H](O)[C@H](O)[C@H]1O	>1000	CHEMBL3737684	0
2	CHEMBL82895	OC1CNC(CNCn2ccnc2)C1O	420.00	CHEMBL650045	0
3	CHEMBL2011623	CCCCN1CCC[C@H](O)[C@@H]1CO	>500	CHEMBL2015864	0
4	CHEMBL84272	OC1CNC(CNCC2CCN(Cc3ccccc3)CC2)C1O	430.00	CHEMBL650045	0
5	CHEMBL501385	OC[C@@H]1CNC[C@H](O)[C@H]1O	84.57	CHEMBL993569, CHEMBL1820880, CHEMBL1820877	0
6	CHEMBL83278	OC(O)CC1NCC(O)C1O	70.00	CHEMBL650045	0
7	CHEMBL3739825	COc1ccc(\C=C\c2cc(OC)cc(OC)c2O[C@H]3O[C@H](CO)[C@H](O)[C@H](O)[C@H]3O)cc1	350.00	CHEMBL3742524	0
8	CHEMBL2011637	CCCCN1CC[C@H](O)[C@@H](O)[C@@H]1CO	>1000	CHEMBL2015864	0
9	CHEMBL3736333	Cl.CC(C)CN[C@@H]1C=C[C@H](O)[C@H](O)[C@H]1O	>1000	CHEMBL3737684	0
10	CHEMBL4085739	CCCCC[C@H]1NC[C@@H](O)[C@@H](O)[C@H]1CO	100.00	CHEMBL4007594	0
11	CHEMBL186150	CCCCCCN1CC(O)C(O)C(O)C1CO	350.00	CHEMBL832785	0
12	CHEMBL2011621	OC[C@H]1[C@H](O)CCCN1CCCCCOCC23CC4CC(CC(C4)C2)C3	>1000	CHEMBL2015864	0
13	CHEMBL87120	NC1C(O)C(O)C(O)C1O	131.83	CHEMBL647637	0
14	CHEMBL2011640	OC[C@H]1[C@@H](O)[C@@H](O)CCN1CCCCCOCC23CC4CC(CC(C4)C2)C3	75.00	CHEMBL2015864	0
15	CHEMBL84104	OC1CNC(CNCc2ccccc2)C1O	370.00	CHEMBL650045	0
16	CHEMBL2011607	OC[C@@H]1NCC=C[C@H]1O	>500	CHEMBL2015864	0
17	CHEMBL362754	CCCN1CC(O)C(O)C(O)C1CO	900.00	CHEMBL832785	0
18	CHEMBL2011624	OC[C@H]1[C@@H](O)CCCN1CCCCCOCC23CC4CC(CC(C4)C2)C3	500.00	CHEMBL2015864	0
19	CHEMBL2011614	CCCCN1CCC[C@H](O)[C@H]1CO	>1000	CHEMBL2015864	0
20	CHEMBL3735749	Cl.O[C@H]1C=C[C@@H](NCc2ccccc2)[C@H](O)[C@H]1O	>1000	CHEMBL3737684	0
21	CHEMBL3754250	CO[C@@H]1[C@@H](O)[C@@H](O)[C@@H](CO)N=C1NCc2ccc(C)cc2	>5000	CHEMBL3815659	0
22	CHEMBL11216	Clc1c(Cl)c(Cl)c2C(=O)N(CCCc3ccccc3)C(=O)c2c1Cl	>500	CHEMBL647638	0

No.	Compound ID	SMILES	IC50 [µM]	ChEMBL Assay ID	Class
23	CHEMBL2011613	OC[C@H]1NCCC[C@@H]1O	>1000	CHEMBL2015864	0
24	CHEMBL2011608	CCCCN1CC=C[C@@H](O)[C@@H]1CO	>500	CHEMBL2015864	0
25	CHEMBL577370	OC[C@H]1O[C@@H]2[C@@H](NC(=O)CCCCOCC34CC5CC(CC(C5)C3)C4)[C@@H]2[C@@H](O)[C@@H]1O	>1000	CHEMBL1040323	0
26	CHEMBL573995	OC[C@H]1O[C@@H]2[C@@H](NCCCCCOCC34CC5CC(CC(C5)C3)C4)[C@@H]2[C@@H](O)[C@@H]1O	>1000	CHEMBL1040323	0
27	CHEMBL4096086	OC[C@@]1(NC[C@@H](O)[C@H]1O)C(=O)O	445.00	CHEMBL4044715	0
28	CHEMBL3736427	Cl.CC(C)CCN[C@@H]1C=C[C@H](O)[C@H](O)[C@H]1O	>1000	CHEMBL3737684	0
29	CHEMBL3735899	Cl.CCCCCCCCN[C@@H]1C=C(C)[C@H](O)[C@H](O)[C@H]1O	>1000	CHEMBL3737684	0
30	CHEMBL184028	CCCCCCCCCC1NC(CO)C(O)C(O)C1O	150.00	CHEMBL832785	0
31	CHEMBL88408	OCC1(O)C(O)C(O)C2N=C(Nc3ccccc3)OC12	>300	CHEMBL647636	0
32	CHEMBL468062	OC[C@H]1N[C@@H]([C@@H](O)[C@@H]1O)c2ccccc2	197.00	CHEMBL1014583	0
33	CHEMBL2011638	OC[C@H]1[C@H](O)[C@@H](O)CCN1CCCCCOCC23CC4CC(CC(C4)C2)C3	20.00	CHEMBL2015864	1
34	CHEMBL470659	OCCCC(=O)CCCCCCCC[C@@H](O)[C@H]1N[C@H](CO)[C@@H](O)[C@@H]1O	0.01	CHEMBL1007617	1
35	CHEMBL3354620	OC[C@@H]1[C@@H](O)[C@H](O)[C@@H](O)CN1CC(F)(F)CCCOCc2ccc(cc2)c3ccccc3	0.01	CHEMBL3384691	1
36	CHEMBL115510	CCCCCCNC1CC(C)C(O)C(O)C1O	0.73	CHEMBL647640	1
37	CHEMBL2011642	O[C@@H]1CN(CCCCCOCC23CC4CC(CC(C4)C2)C3)C[C@H](O)[C@H]1O	0.80	CHEMBL2015864	1
38	CHEMBL324840	CC1CC(NCCCCc2ccccc2)C(O)C(O)C1O	1.40	CHEMBL647640	1
39	CHEMBL186068	CCCCCCCCN1CC(O)C(O)C(O)C1CO	34.00	CHEMBL832785	1
40	CHEMBL3354028	OC[C@H]1[C@@H](O)[C@H](O)[C@@H](O)CN1CCCC\C=C/C23CC4CC(CC(C4)C2)C3	0.01	CHEMBL3384691	1
41	CHEMBL3354036	OC[C@H]1[C@@H](O)[C@H](O)[C@@H](O)CN1CCCCCOCc2cc3ccccc3c4ccccc24	0.20	CHEMBL3384691	1
42	CHEMBL3354014	OC[C@@H]1[C@@H](O)[C@H](O)[C@@H](O)CN1CCCCCOCC2CCCCC2	$4.00 \cdot 10^{-3}$	CHEMBL3384691	1
43	CHEMBL3815031	CCCCC(CC)CNC1=N[C@H](CO)[C@H](O)[C@H](O)[C@H]1O	2.50	CHEMBL3815659	1

No.	Compound ID	SMILES	IC50 [µM]	ChEMBL Assay ID	Class
44	CHEMBL3354021	OC[C@@H]1[C@@H](O)[C@H](O)[C@@H](O)CN1CCCCCOCc2ccccc2	$1.00 \cdot 10^{-3}$	CHEMBL3384691	1
45	CHEMBL3354038	OC[C@H]1[C@@H](O)[C@H](O)[C@@H](O)CN1CCCCCOCc2ccc(cc2)c3ccccc3	$3.00 \cdot 10^{-3}$	CHEMBL3384691	1
46	CHEMBL2011629	CCCCN1CC[C@@H](O)[C@@H](O)[C@@H]1CO	30.00	CHEMBL2015864	1
47	CHEMBL513394	OC[C@H]1N[C@H]([C@H](O)CCCCCCCC(=O)CCCO[C@@H]2O[C@H](CO)[C@@H](O)[C@H](O)[C@H]2O)[C@@H](O)[C@@H]1O	0.01	CHEMBL1007617	1
48	CHEMBL326599	CCCCCCCCCCCCN[C@H]1CC(C)[C@@H](O)C(O)[C@@H]1O	3.60	CHEMBL647640	1
49	CHEMBL3354029	OC[C@H]1[C@@H](O)[C@H](O)[C@@H](O)CN1CCCCCCOCC23CC4CC(CC(C4)C2)C3	$2.50 \cdot 10^{-3}$	CHEMBL3384691	1
50	CHEMBL2011631	CCCCN1CC[C@@H](O)[C@H](O)[C@@H]1CO	20.00	CHEMBL2015864	1
51	CHEMBL3354626	OC[C@@H]1[C@@H](O)[C@H](O)[C@@H](O)CN1CCCCCOCc2ccc(cc2C(F)(F)F)c3ccccc3	$1.20 \cdot 10^{-4}$	CHEMBL3384691	1
52	CHEMBL86865	OC1C(O)C2OC(=NC2C1O)Nc3ccccc3	10.85	CHEMBL647636	1
53	CHEMBL314757	CCCCCCCCNC1CC(C)C(O)C(O)C1O	1.50	CHEMBL647640	1
54	CHEMBL3354022	OC[C@@H]1[C@@H](O)[C@H](O)[C@@H](O)CN1CCCCCOCc2ccc3ccccc3c2	$1.00 \cdot 10^{-3}$	CHEMBL3384691	1
55	CHEMBL3354024	OC[C@@H]1[C@@H](O)[C@H](O)[C@@H](O)CN1CCCCCOCc2ccc3ccc4cccc5ccc2c3c45	$3.00 \cdot 10^{-3}$	CHEMBL3384691	1
56	CHEMBL469844	OC[C@H]1N[C@H](CO)[C@@H](O)[C@H]1O	23.90	CHEMBL1007695, CHEMBL1168037	1
57	CHEMBL3354035	OC[C@H]1[C@@H](O)[C@H](O)[C@@H](O)CN1CCCCCOCc2ccc3ccccc3c2	$1.00 \cdot 10^{-3}$	CHEMBL3384691	1
58	CHEMBL3359675	CCCCCCCCCCCC\N=C\1/N[C@H](CO)[C@H](O)[C@H](O)[C@H]1O	0.40	CHEMBL3815659	1
59	CHEMBL3354049	OC[C@@H]1[C@@H](O)[C@H](O)[C@@H](O)CN1CCCCCOCc2ccc(cc2)c3cccc(F)c3	$1.00 \cdot 10^{-3}$	CHEMBL3384691	1
60	CHEMBL3354630	OC[C@H]1[C@@H](O)[C@H](O)[C@@H](O)CN1CCCCCOCc2ccccc2c3ccccc3	$3.00 \cdot 10^{-3}$	CHEMBL3384691	1
61	CHEMBL3354634	OC[C@H]1[C@@H](O)[C@H](O)[C@@H](O)CN1CCCCCOCc2ccc(cc2C(F)(F)F)c3ccccc3	$1.00 \cdot 10^{-4}$	CHEMBL3384691	1

No.	Compound ID	SMILES	IC50 [µM]	ChEMBL Assay ID	Class
62	CHEMBL3354018	CC(C)CCC[C@@H](C)[C@H]1CC[C@H]2[C@@H]3CC=C4C[C@H](CC[C@]4(C)[C@H]3CC[C@]12C)OCCCCCN5C[C@H](O)[C@@H](O)[C@H](O)[C@H]5CO	0.01	CHEMBL3384691	1
63	CHEMBL3972306	CC1=CC(=O)Oc2cc(OCCCCn3cc(CCCCN4C[C@H](O)[C@@H](O)[C@H](O)[C@H]4CO)nn3)ccc12	9.30	CHEMBL3874807	1
64	CHEMBL3354055	OC[C@@H]1[C@@H](O)[C@H](O)[C@@H](O)CN1CCCCCOCc2ccc(cc2)c3ccc4OCCOc4c3	0.03	CHEMBL3384691	1
65	CHEMBL9020	O=C1N(C(=O)c2ccccc12)c3ccccc3	>500	CHEMBL647638	0
66	CHEMBL2011601	OC[C@H]1NCC=C[C@@H]1O	>1000	CHEMBL2015864	0
67	CHEMBL356023	CCCCN1CC(C)C(O)C(O)C1	80.00	CHEMBL820821	0
68	CHEMBL3734817	Cl.O[C@H]1C=C[C@@H](NC2CCCCC2)[C@H](O)[C@H]1O	>1000	CHEMBL3737684	0
69	CHEMBL2011602	CCCCN1CC=C[C@H](O)[C@H]1CO	250.00	CHEMBL2015864	0
70	CHEMBL445624	CC(=O)N(CC1NCC(O)C1O)Cc2ccccc2	430.00	CHEMBL650045	0
71	CHEMBL511823	CC(O)[C@@H](O)[C@H]1N[C@H](CO)[C@@H](O)[C@@H]1O	120.00	CHEMBL1007617	0
72	CHEMBL3980365	CN(C)c1cccc2c(cccc12)S(=O)(=O)NCCOCCn3cc(CCCCN4C[C@H](O)[C@@H](O)[C@H](O)[C@H]4CO)nn3	97.80	CHEMBL3874807	0
73	CHEMBL313295	OC1CNC(CNCc2occc2)C1O	140.00	CHEMBL650045	0
74	CHEMBL2011610	OC[C@@H]1NCC=C[C@@H]1O	>1000	CHEMBL2015864	0
75	CHEMBL3736274	Cl.CCCCCCCCCCN[C@@H]1C=C[C@H](O)[C@H](O)[C@H]1O	>1000	CHEMBL3737684	0
76	CHEMBL2204848	O[C@H]1[C@H](O)[C@H](C=C[C@@H]1N2CCc3ccccc23)N4CCc5ccccc45	67.70	CHEMBL2216697	0
77	CHEMBL2207396	O[C@@H]1CNC[C@@H]1O	600.00	CHEMBL650045	0
78	CHEMBL2011630	OC[C@H]1[C@H](O)[C@H](O)CCN1CCCCCOCC23CC4CC(CC(C4)C2)C3	100.00	CHEMBL2015864	0
79	CHEMBL2011633	CCCCN1CC[C@H](O)[C@H](O)[C@H]1CO	1000.00	CHEMBL2015864	0
80	CHEMBL275285	Clc1c(Cl)c(Cl)c2C(=O)N(CCCCc3ccccc3)C(=O)c2c1Cl	>500	CHEMBL647638	0
81	CHEMBL3970812	CCCCCCCCCCNC(=S)N1[C@@H](CO)[C@H](O)[C@@H](O)[C@@H]1CO	65.00	CHEMBL3869409	0
82	CHEMBL369297	OC[C@@H]1NC[C@@H](O)[C@H](O)[C@H]1O	980.00	CHEMBL1820877	0

No.	Compound ID	SMILES	IC50 [µM]	ChEMBL Assay ID	Class
83	CHEMBL2011605	CCCCN1CC=C[C@@H](O)[C@H]1CO	>1000	CHEMBL2015864	0
84	CHEMBL2011609	OC[C@H]1[C@H](O)C=CCN1CCCCCOCC23CC4CC(CC(C4)C2)C3	>500	CHEMBL2015864	0
85	CHEMBL463872	COc1ccc(cc1)[C@@H]2N[C@@H](CO)[C@H](O)[C@H]2O	>200	CHEMBL1014583	0
86	CHEMBL86772	OCC12OC(=NC1C(O)C(O)C2O)Nc3ccccc3	201.63	CHEMBL647636	0
87	CHEMBL2011617	CCCCN1CCC[C@@H](O)[C@H]1CO	>1000	CHEMBL2015864	0
88	CHEMBL3735277	Cl.CCCCN[C@@H]1C=C[C@H](O)[C@H](O)[C@H]1O	>1000	CHEMBL3737684	0
89	CHEMBL152232	CCCCN1CC(O)C(O)C(O)C1	530.00	CHEMBL820821	0
90	CHEMBL421340	CC(NCC1NCC(O)C1O)C(O)(c2ccccc2)c3ccccc3	550.00	CHEMBL650045	0
91	CHEMBL2206827	C[C@H](O)NC[C@H]1NC[C@H](O)[C@@H]1O	290.00	CHEMBL650045	0
92	CHEMBL1561	OCCN1C[C@H](O)[C@@H](O)[C@H](O)[C@H]1CO	231.00	CHEMBL993569, CHEMBL3874807	0
93	CHEMBL11516	Clc1c(Cl)c(Cl)c2C(=O)N(Cc3ccccc3)C(=O)c2c1Cl	>500	CHEMBL647638	0
94	CHEMBL314403	OCC1(O)C(O)C(O)C2(CO)OCC(=NC12)Nc3ccccc3	211.00	CHEMBL647636	0
95	CHEMBL3742008	COc1ccc(\C=C\c2cc(OC)cc(OC)c2O[C@H]3O[C@H](CO)[C@@H](O)[C@H](O)[C@H]3O)cc1	208.00	CHEMBL3742524	0
96	CHEMBL507492	COc1ccc(cc1)[C@H]2[C@H](O)[C@@H](O)[C@H](CO)N2C	>200	CHEMBL1014583	0
97	CHEMBL313375	OCC1C(O)C(O)C2OC(=NC12)Nc3ccccc3	>300	CHEMBL647636	0
98	CHEMBL1818439	OC[C@@H]1NCC[C@H](O)[C@@H]1O	>1000	CHEMBL2015864	0
99	CHEMBL574865	N[C@@H]1[C@H]2O[C@H](CO)[C@@H](O)[C@H](O)[C@@H]12	>1000	CHEMBL1040323	0
100	CHEMBL464053	COc1ccc(cc1OC)[C@@H]2[C@@H](O)[C@H](O)[C@@H](CO)N2C	>200	CHEMBL1014583	0
101	CHEMBL1933096	Cl.OC[C@@H]1CNC[C@@H](O)[C@@H](O)[C@@H]1O	157.00	CHEMBL1937704	0
102	CHEMBL3740658	COc1cc(OC)c(O[C@H]2O[C@H](CO)[C@@H](O)[C@H](O)[C@H]2O)c(\C=C\c3ccc(OC)c(OC)c3)c1	192.00	CHEMBL3742524	0
103	CHEMBL2011620	CCCCN1CCC[C@@H](O)[C@@H]1CO	>500	CHEMBL2015864	0
104	CHEMBL3736474	Cl.OCCCN[C@@H]1C=C[C@H](O)[C@H](O)[C@H]1O	>1000	CHEMBL3737684	0

No.	Compound ID	SMILES	IC50 [µM]	ChEMBL Assay ID	Class
105	CHEMBL448744	COc1ccc(cc1)[C@H]2N[C@H](CO)[C@@H](O)[C@@H]2O	122.00	CHEMBL1014583	0
106	CHEMBL3740283	COc1cc(OC)cc(\C=C\c2cc(OC)cc(OC)c2O[C@H]3O[C@H](CO)[C@@H](O)[C@H](O)[C@H]3O)c1	272.00	CHEMBL3742524	0
107	CHEMBL3359684	OC[C@H]1NC(=O)[C@H](O)[C@@H](O)[C@H]1O	>1000	CHEMBL3815659	0
108	CHEMBL584580	CCCC(=O)N[C@@H]1[C@H]2O[C@H](CO)[C@@H](O)[C@H](O)[C@@H]12	>1000	CHEMBL1040323	0
109	CHEMBL1236649	OC[C@H]1C[C@H](N[C@H]2C=C(CO)[C@@H](O)[C@H](O)[C@H]2O)[C@H](O)[C@@H](O)[C@@H]1O	>1000	CHEMBL3073479	0
110	CHEMBL465105	COc1ccc([C@@H]2[C@@H](O)[C@H](O)[C@@H](CO)N2C)c(OC)c1	>200	CHEMBL1014583	0
111	CHEMBL275509	Clc1c(Cl)c(Cl)c2C(=O)N(CCCCCCc3ccccc3)C(=O)c2c1Cl	>500	CHEMBL647638	0
112	CHEMBL3133385	Cl.CCCCCCN1C[C@H](O)[C@@H](O)[C@H](O)[C@H]1CO	59.00	CHEMBL3134886	0
113	CHEMBL583176	CCCCN[C@@H]1[C@H]2O[C@H](CO)[C@@H](O)[C@H](O)[C@@H]12	>1000	CHEMBL1040323	0
114	CHEMBL503122	COc1ccc(cc1O)[C@@H]2[C@@H](O)[C@H](O)[C@@H](CO)N2C	>200	CHEMBL1014583	0
115	CHEMBL2011618	OC[C@@H]1[C@H](O)CCCN1CCCCCOCC23CC4CC(CC(C4)C2)C3	>1000	CHEMBL2015864	0
116	CHEMBL502396	COc1ccc([C@H]2N[C@H](CO)[C@@H](O)[C@@H]2O)c(OC)c1	96.00	CHEMBL1014583	0
117	CHEMBL1818435	OC[C@@H]1NCC[C@H](O)[C@H]1O	>1000	CHEMBL2015864	0
118	CHEMBL3359683	OC[C@H]1NC(=N)[C@H](O)[C@@H](O)[C@H]1O	>1000	CHEMBL3815659	0
119	CHEMBL11477	Nc1ccc2C(=O)N(C(=O)c2c1)c3ccccc3	>500	CHEMBL647638	0
120	CHEMBL465034	COc1ccc(OC)c(c1)[C@@H]2[C@@H](O)[C@H](O)[C@@H](CO)N2C	>200	CHEMBL1014583	0
121	CHEMBL3133390	Cl.OCCNC(=N)N1C[C@H](O)[C@@H](O)[C@H](O)[C@H]1CO	457.00	CHEMBL3134886	0
122	CHEMBL11322	O=C1N(CCCc2ccccc2)C(=O)c3ccccc13	>500	CHEMBL647638	0
123	CHEMBL108084	OC[C@H]1NCC[C@@H](O)[C@@H]1O	>1000	CHEMBL2015864	0
124	CHEMBL505237	OC[C@H]1NCC[C@H](O)[C@H]1O	>1000	CHEMBL2015864	0
125	CHEMBL2011622	OC[C@@H]1NCCC[C@@H]1O	>1000	CHEMBL2015864	0
126	CHEMBL80254	OC[C@H]1NC[C@@H](O)[C@@H]1O	120.00	CHEMBL922471	0

No.	Compound ID	SMILES	IC50 [μM]	ChEMBL Assay ID	Class
127	CHEMBL2011639	CCCCN1CC[C@H](O)[C@H](O)[C@@H]1CO	>1000	CHEMBL2015864	0
128	CHEMBL259905	OC[C@@H]1N[C@H](CO)[C@@H](O)[C@@H]1O	320.00	CHEMBL1007695	0
129	CHEMBL3736146	Cl.CCCCCN[C@@H]1C=C[C@H](O)[C@H](O)[C@H]1O	>1000	CHEMBL3737684	0
130	CHEMBL11566	Oc1cccc2C(=O)N(C(=O)c12)c3ccccc3	>500	CHEMBL647638	0
131	CHEMBL3891002	CCCCCCCCCCCCNC(=S)N1[C@@H](CO)[C@H](O)[C@@H](O)[C@@H]1CO	73.00	CHEMBL3869409	0
132	CHEMBL1566	C[C@H]1O[C@H](O[C@H]2[C@H](O)[C@@H](O)[C@@H](O[C@H]3[C@H](O)[C@@H](O)[C@H](O)O[C@@H]3CO)O[C@@H]2CO)[C@H](O)[C@@H](O)[C@@H]1N[C@H]4C=C(CO)[C@@H](O)[C@H](O)[C@H]4O	103.12	CHEMBL2216697	0
133	CHEMBL276643	Clc1c(Cl)c(Cl)c2C(=O)N(CCCCCc3ccccc3)C(=O)c2c1Cl	>500	CHEMBL647638	0
134	CHEMBL468063	COc1ccc(cc1O)[C@H]2N[C@H](CO)[C@@H](O)[C@@H]2O	>200	CHEMBL1014583	0
135	CHEMBL456583	OC[C@H]1NCC[C@H](O)[C@@H]1O	416.00	CHEMBL2015864, CHEMBL1008472, CHEMBL1820877, CHEMBL1007669, CHEMBL647648	0
136	CHEMBL312653	OC[C@H]1N[C@H](CO)[C@@H](O)[C@@H]1O	64.67	CHEMBL922471, CHEMBL3869402, CHEMBL647648	0
137	CHEMBL273605	[O-][N+](=O)c1ccc2C(=O)N(C(=O)c2c1)c3ccccc3	>500	CHEMBL647638	0
138	CHEMBL3742045	COc1cc(OC)c(O[C@H]2O[C@H](CO)[C@H](O)[C@H](O)[C@H]2O)c(\C=C\c3ccc(OC)c(OC)c3)c1	170.00	CHEMBL3742524	0
139	CHEMBL150938	OC1CNCC(=C)C1O	>1000	CHEMBL820821	0
140	CHEMBL1818321	OC[C@H]1CNC[C@H](O)[C@@H]1O	278.00	CHEMBL1820880, CHEMBL1820877	0
141	CHEMBL3753015	CO[C@@H]1[C@H](O)[C@@H](O)C(=N[C@@H]1CO)NCc2ccc(C)cc2	>5000	CHEMBL3815659	0
142	CHEMBL466791	OC[C@H]1N[C@@H]([C@@H](O)[C@@H]1O)c2ccc(O)cc2	>200	CHEMBL1014583	0
143	CHEMBL3133386	CCCCNC(=O)N1C[C@H](O)[C@@H](O)[C@H](O)[C@H]1CO	>1000	CHEMBL3134886	0
144	CHEMBL3943978	CCCC\N=C/1\SC[C@@H]2[C@@H](O)[C@H](O)[C@@H](CO)N12	532.00	CHEMBL3869409	0

No.	Compound ID	SMILES	IC50 [µM]	ChEMBL Assay ID	Class
145	CHEMBL11766	Clc1c(Cl)c(Cl)c2C(=O)N(CCc3ccccc3)C(=O)c2c1Cl	>500	CHEMBL647638	0
146	CHEMBL3752112	Cc1ccc(CNC2=N[C@H](CO)[C@H](O)[C@H](O)C2)cc1	>5000	CHEMBL3815659	0
147	CHEMBL2115197	CC[C@H]1NC[C@H](O)[C@@H]1O	175.00	CHEMBL650045, CHEMBL647648	0
148	CHEMBL2207397	OCC(O)[C@H]1NC[C@H](O)[C@@H]1O	120.00	CHEMBL650045	0
149	CHEMBL3736331	Cl.CCC(CC)N[C@@H]1C=C[C@H](O)[C@H](O)[C@H]1O	>1000	CHEMBL3737684	0
150	CHEMBL11452	O=C1N(CCc2ccccc2)C(=O)c3ccccc13	>500	CHEMBL647638	0
151	CHEMBL2011616	OC[C@H]1NCCC[C@H]1O	>1000	CHEMBL2015864	0
152	CHEMBL2011619	OC[C@@H]1NCCC[C@H]1O	>1000	CHEMBL2015864	0
153	CHEMBL463871	COc1ccc(cc1)[C@@H]2[C@@H](O)[C@H](O)[C@@H](CO)N2C	>200	CHEMBL1014583	0
154	CHEMBL2011606	OC[C@@H]1[C@H](O)C=CCN1CCCCCOCC23CC4CC(CC(C4)C2)C3	130.00	CHEMBL2015864	0
155	CHEMBL416887	O=C1N(Cc2ccccc2)C(=O)c3ccccc13	>500	CHEMBL647638	0
156	CHEMBL2011615	OC[C@@H]1[C@@H](O)CCCN1CCCCCOCC23CC4CC(CC(C4)C2)C3	100.00	CHEMBL2015864	0
157	CHEMBL2206826	C[C@@H](O)NC[C@H]1NC[C@H](O)[C@@H]1O	410.00	CHEMBL650045	0
158	CHEMBL3735475	Cl.O[C@H]1C=C[C@@H](NCCc2ccccc2)[C@H](O)[C@H]1O	>1000	CHEMBL3737684	0
159	CHEMBL3359679	OC[C@H]1N\C(=N/C2CCCCC2)\[C@H](O)[C@@H](O)[C@H]1O	>5000	CHEMBL3815659	0
160	CHEMBL187158	CCCCCCCCCN1CC(O)C(O)C(O)C1CO	150.00	CHEMBL832785	0
161	CHEMBL3133389	Cl.OCCOCCNC(=N)N1C[C@H](O)[C@@H](O)[C@H](O)[C@H]1CO	>1000	CHEMBL3134886	0
162	CHEMBL1163254	CC[C@H]1CC[C@@H](O)[C@@H](CO[C@@H]2O[C@H](CO)[C@@H](O)[C@H](O)[C@H]2O)N1	321.00	CHEMBL1168037	0
163	CHEMBL1818437	OC[C@@H]1NCC[C@@H](O)[C@@H]1O	>1000	CHEMBL2015864	0
164	CHEMBL3736234	Cl.CCC(CC)CN[C@@H]1C=C[C@H](O)[C@H](O)[C@H]1O	>1000	CHEMBL3737684	0
165	CHEMBL2011625	CCCCN1CC[C@@H](O)[C@H](O)[C@H]1CO	>1000	CHEMBL2015864	0
166	CHEMBL3735278	Cl.O[C@H]1C=C[C@@H](NCC2CCCCC2)[C@H](O)[C@H]1O	>1000	CHEMBL3737684	0
167	CHEMBL2409320	OC[C@H](O)[C@@H]1NC[C@@H]1O	347.00	CHEMBL2412536	0

No.	Compound ID	SMILES	IC50 [μM]	ChEMBL Assay ID	Class
168	CHEMBL276030	Oc1ccc2C(=O)N(C(=O)c2c1)c3ccccc3	>500	CHEMBL647638	0
169	CHEMBL1818434	OC[C@@H]1CNC[C@H](O)[C@@H]1O	983.00	CHEMBL1820877	0
170	CHEMBL2011627	CCCCN1CC[C@@H](O)[C@@H](O)[C@H]1CO	>1000	CHEMBL2015864	0
171	CHEMBL3814915	Cc1ccc(CNC2=N[C@H](CO)[C@@H](O)[C@@H]2O)cc1	6200.00	CHEMBL3815659	0
172	CHEMBL11614	[O-][N+](=O)c1cccc2C(=O)N(C(=O)c12)c3ccccc3	>500	CHEMBL647638	0
173	CHEMBL3133392	Cl.OC[C@@H]1[C@@H](O)[C@H](O)[C@@H](O)CN1C(=N)NCCCc2ccccc2	67.00	CHEMBL3134886	0
174	CHEMBL269410	Nc1cccc2C(=O)N(C(=O)c12)c3ccccc3	>500	CHEMBL647638	0
175	CHEMBL445980	COc1ccc(OC)c(c1)[C@H]2N[C@H](CO)[C@@H](O)[C@@H]2O	169.00	CHEMBL1014583	0
176	CHEMBL1818438	OC[C@H]1NCC[C@@H](O)[C@H]1O	>1000	CHEMBL2015864	0
177	CHEMBL2011604	OC[C@H]1NCC=C[C@H]1O	>1000	CHEMBL2015864	0
178	CHEMBL511534	CCCCN1[C@H](CO)[C@@H](O)[C@H](O)[C@H]1c2ccc(OC)c(O)c2	>200	CHEMBL1014583	0
179	CHEMBL84940	OC1CNC(CNC2CCCC2)C1O	500.00	CHEMBL650045	0
180	CHEMBL185536	CC(=O)NC1CC(CO)C(O)C(O)C1O	14.00•10^{6}	CHEMBL831683	0
181	CHEMBL1628264	N[C@H]1C[C@H](CO)[C@@H](O)[C@H](O)[C@H]1O	13.00•10^{7}	CHEMBL831683	0
182	CHEMBL3349573	OC[C@@H](OS(=O)(=O)[O-])[C@@H](O)C[S+]1C[C@@H](O)[C@H](O)[C@@H]1CO	3400.00	CHEMBL650041	0
183	CHEMBL357108	CC1CNCC(O)C1O	500.00	CHEMBL820821	0
184	CHEMBL88413	NC1C(O)C(O)C(O)C1(O)CO	164.65	CHEMBL647637	0
185	CHEMBL1922579	Cl.CCCCCCCCN[C@@H]1C=C(CO)[C@H](O)[C@H](O)[C@H]1O	504.05	CHEMBL3737684, CHEMBL1924471	0
186	CHEMBL368121	OC[C@H]1NC[C@@H](O)[C@H](O)[C@H]1O	66.00	CHEMBL826964	0
187	CHEMBL454551	COc1ccc(cc1O)[C@@H]2N[C@@H](CO)[C@H](O)[C@H]2O	>200	CHEMBL1014583	0
188	CHEMBL511366	COc1ccc(cc1OC)[C@@H]2N[C@@H](CO)[C@H](O)[C@H]2O	>200	CHEMBL1014583	0
189	CHEMBL2011635	CCCCN1CC[C@H](O)[C@@H](O)[C@H]1CO	>1000	CHEMBL2015864	0

No.	Compound ID	SMILES	IC50 [μM]	ChEMBL Assay ID	Class
190	CHEMBL2204849	CC(=O)O[C@H]1[C@H](OC(=O)C)[C@H](C=C[C@@H]1n2ccc3ccccc23)n4ccc5ccccc45	52.80	CHEMBL2216697	0
191	CHEMBL3735383	Cl.CCCCCCCCN[C@@H]1C=C[C@H](O)[C@H](O)[C@H]1O	>1000	CHEMBL3737684	0
192	CHEMBL3133393	Cl.OC[C@@H]1[C@@H](O)[C@H](O)[C@@H](O)CN1C(=N)NCCCOCC23CC4CC(CC(C4)C2)C3	489.00	CHEMBL3134886	0
193	CHEMBL307429	OC[C@H]1NC[C@H](O)[C@@H](O)[C@@H]1O	241.45	CHEMBL826964, CHEMBL993569, CHEMBL1040323, CHEMBL647638, CHEMBL3134886, CHEMBL647636, CHEMBL3874807, CHEMBL832765, CHEMBL1820877, CHEMBL647648	0
194	CHEMBL414447	COC1CNCC(O)C1O	>1000	CHEMBL820821	0
195	CHEMBL117201	CCCCCCCCN[C@H]1CC(C)[C@@H](O)C(O)[C@@H]1O	14.70	CHEMBL647640	1
196	CHEMBL3359670	Cc1ccccc1C\N=C\2/N[C@H](CO)[C@H](O)[C@H](O)[C@H]2O	8.40	CHEMBL3815659	1
197	CHEMBL2011636	OC[C@@H]1[C@H](O)[C@@H](O)CCN1CCCCCOCC23CC4CC(CC(C4)C2)C3	3.00	CHEMBL2015864	1
198	CHEMBL3354044	COc1ccccc1c2ccc(COCCCCCN3C[C@H](O)[C@@H](O)[C@H](O)[C@H]3CO)cc2	$3.00 \cdot 10^{-3}$	CHEMBL3384691	1
199	CHEMBL35576	OC[C@@H]1[C@@H](O)[C@H](O)[C@@H](O)[C@@H]2O[C@H]12	4.54	CHEMBL1024152	1
200	CHEMBL3359668	OC[C@H]1N\C(=N/Cc2cccc(F)c2)\[C@H](O)[C@@H](O)[C@H]1O	11.00	CHEMBL3815659	1
201	CHEMBL117074	CCCCCCCCCCNC1CC(C)C(O)C(O)C1O	2.00	CHEMBL647640	1
202	CHEMBL206468	OC[C@H]1CNC[C@@H](O)[C@@H]1O	0.37	CHEMBL1820880, CHEMBL993569, CHEMBL1820877	1
203	CHEMBL470661	OC[C@H]1N[C@H]([C@H](O)CCCC[C@@H]2CCC[C@]3(CCCO3)O2)[C@@H](O)[C@@H]1O	0.01	CHEMBL1007617	1
204	CHEMBL3359682	OC[C@H]1N\C(=N/CC2CCCCCC2)\[C@H](O)[C@@H](O)[C@H]1O	5.00	CHEMBL3815659	1
205	CHEMBL3354635	OC[C@H]1[C@@H](O)[C@H](O)[C@@H](O)CN1CCCCCOCc2ccc(c3ccccc3)c(c2)C(F)(F)F	$1.00 \cdot 10^{-4}$	CHEMBL3384691	1
206	CHEMBL3897971	CCCCCCCCCC\N=C/1\SC[C@@H]2[C@@H](O)[C@H](O)[C@@H](CO)N12	1.00	CHEMBL3869409	1

No.	Compound ID	SMILES	IC50 [µM]	ChEMBL Assay ID	Class
207	CHEMBL3354043	COc1cccc(c1)c2ccc(COCCCCCN3C[C@H](O)[C@@H](O)[C@H](O)[C@H]3CO)cc2	0.02	CHEMBL3384691	1
208	CHEMBL3359124	OC[C@H]1N\C(=N/Cc2ccc(F)cc2)\[C@H](O)[C@@H](O)[C@H]1O	4.70	CHEMBL3815659	1
209	CHEMBL3354064	OC[C@@H]1[C@@H](O)[C@H](O)[C@@H](O)CN1CCCCCOCc2ccc(cn2)c3ccccc3	$3.00 \cdot 10^{-3}$	CHEMBL3384691	1
210	CHEMBL3354061	OC[C@@H]1[C@@H](O)[C@H](O)[C@@H](O)CN1CCCCCOCc2ccc(cc2)c3ccc(F)nc3	0.01	CHEMBL3384691	1
211	CHEMBL3359677	OC[C@H]1N\C(=N/CCCc2ccccc2)\[C@H](O)[C@@H](O)[C@H]1O	1.30	CHEMBL3815659	1
212	CHEMBL3359122	COc1ccc(C\N=C\2/N[C@H](CO)[C@H](O)[C@H](O)[C@H]2O)cc1	13.00	CHEMBL3815659	1
213	CHEMBL3916914	CCCCCC\N=C/1\SC[C@@H]2[C@@H](O)[C@H](O)[C@@H](CO)N12	16.00	CHEMBL3869409	1
214	CHEMBL3354042	COc1ccc(cc1)c2ccc(COCCCCCN3C[C@H](O)[C@@H](O)[C@H](O)[C@H]3CO)cc2	0.01	CHEMBL3384691	1
215	CHEMBL1933098	Cl.C[C@@H]1CNC[C@@H](O)[C@@H](O)[C@@H]1O	14.00	CHEMBL1937704	1
216	CHEMBL3354627	OC[C@@H]1[C@@H](O)[C@H](O)[C@@H](O)CN1CCCCCOCc2ccc(c3ccccc3)c(c2)C(F)(F)F	$8.00 \cdot 10^{-5}$	CHEMBL3384691	1
217	CHEMBL2011641	C[C@@H]1[C@@H](O)[C@H](O)[C@@H](O)CN1CCCCCOCC23CC4CC(CC(C4)C2)C3	0.03	CHEMBL2015864	1
218	CHEMBL3354050	OC[C@@H]1[C@@H](O)[C@H](O)[C@@H](O)CN1CCCCCOCc2ccc(cc2)c3ccccc3F	$2.00 \cdot 10^{-3}$	CHEMBL3384691	1
219	CHEMBL116366	CCCCCCCCCCN[C@H]1CC(C)[C@@H](O)C(O)[C@@H]1O	16.60	CHEMBL647640	1
220	CHEMBL3359672	Cc1ccc(C\N=C\2/N[C@H](CO)[C@H](O)[C@H](O)[C@H]2O)cc1	3.00	CHEMBL3815659	1
221	CHEMBL110830	O[C@@H]1CNC[C@H](O)[C@H]1O	5.55	CHEMBL993569, CHEMBL820821	1
222	CHEMBL3359678	OC[C@H]1N\C(=N/C2CC2)\[C@H](O)[C@@H](O)[C@H]1O	32.00	CHEMBL3815659	1
223	CHEMBL3354629	C[C@@H](OCCCCCN1C[C@H](O)[C@@H](O)[C@H](O)[C@@H]1CO)c2ccc(cc2)c3ccccc3	$1.00 \cdot 10^{-3}$	CHEMBL3384691	1
224	CHEMBL469435	OCC(O)CCCCC(O)CC(O)CCC[C@@H](O)[C@H]1N[C@H](CO)[C@@H](O)[C@@H]1O	6.60	CHEMBL1007617	1
225	CHEMBL3354025	OC[C@@H]1[C@@H](O)[C@H](O)[C@@H](O)CN1CCCCCOCc2ccc(cc2)c3ccccc3	$2.00 \cdot 10^{-3}$	CHEMBL3384691	1

No.	Compound ID	SMILES	IC50 [µM]	ChEMBL Assay ID	Class
226	CHEMBL311226	O[C@H]1CCN2C[C@H](O)[C@@H](O)[C@H](O)[C@@H]12	19.00	CHEMBL2342949	1
227	CHEMBL3354053	OC[C@@H]1[C@@H](O)[C@H](O)[C@@H](O)CN1CCCCCOCc2ccc(cc2)c3cc(cc(c3)C(F)(F)F)C(F)(F)F	0.02	CHEMBL3384691	1
228	CHEMBL3354048	OC[C@@H]1[C@@H](O)[C@H](O)[C@@H](O)CN1CCCCCOCc2ccc(cc2)c3ccc(F)cc3	$1.00 \cdot 10^{-3}$	CHEMBL3384691	1
229	CHEMBL364554	CCCCCCC1NC(CO)C(O)C(O)C1O	12.00	CHEMBL832785	1
230	CHEMBL3354062	OC[C@@H]1[C@@H](O)[C@H](O)[C@@H](O)CN1CCCCCOCc2ccccc2c3ccccc3	$3.00 \cdot 10^{-3}$	CHEMBL3384691	1
231	CHEMBL574645	OC[C@@H]1[C@@H](O)[C@H](O)[C@@H](O)CN1CCCCCOCC23CC4CC(CC(C4)C2)C3	$1.33 \cdot 10^{-3}$	CHEMBL3384691, CHEMBL1040323, CHEMBL2015864	1
232	CHEMBL2011632	OC[C@H]1[C@@H](O)[C@H](O)CCN1CCCCCOCC23CC4CC(CC(C4)C2)C3	0.50	CHEMBL2015864	1
233	CHEMBL3354045	OC[C@@H]1[C@@H](O)[C@H](O)[C@@H](O)CN1CCCCCOCc2ccc(cc2)c3ccc(cc3)C#N	$3.00 \cdot 10^{-3}$	CHEMBL3384691	1
234	CHEMBL3354032	OC[C@H]1[C@@H](O)[C@H](O)[C@@H](O)CN1CCCCCOCC2C3CC4CC(CC2C4)C3	$2.00 \cdot 10^{-3}$	CHEMBL3384691	1
235	CHEMBL1088158	OC[C@@H]1[C@H](O)[C@H](O)[C@@H](O)CN1CCCCCOCC23CC4CC(CC(C4)C2)C3	$1.00 \cdot 10^{-3}$	CHEMBL3384691	1
236	CHEMBL3133387	Cl.CCCCNC(=N)N1C[C@H](O)[C@@H](O)[C@H](O)[C@H]1CO	41.00	CHEMBL3134886	1
237	CHEMBL3944457	CCCCCCCCNC(=S)N1[C@H](CO)[C@@H](O)[C@H](O)[C@H]1CO	12.00	CHEMBL3869409	1
238	CHEMBL425308	CC(O)CC[C@@H]1CC[C@@H]2[C@@H](O)[C@H](O)[C@@H](CO)N12	49.00	CHEMBL1007617	1
239	CHEMBL485840	CCCCCCCCCOC(=O)N1C[C@H](O)[C@@H](O)[C@H](O)[C@H]1CO	0.02	CHEMBL971353	1
240	CHEMBL3354057	OC[C@@H]1[C@@H](O)[C@H](O)[C@@H](O)CN1CCCCCOCc2ccc(cc2)c3ccncc3	0.01	CHEMBL3384691	1
241	CHEMBL1086996	OC[C@H]1[C@@H](O)[C@H](O)[C@@H](O)CN1CCCCCOCC23CC4CC(CC(C4)C2)C3	0.02	CHEMBL2015864, CHEMBL3384691	1
242	CHEMBL4059852	OCC1(CO)NC[C@H](F)[C@H]1O	0.18	CHEMBL4044716	1
243	CHEMBL3354059	OC[C@@H]1[C@@H](O)[C@H](O)[C@@H](O)CN1CCCCCOCc2ccc(cc2)c3cncnc3	0.03	CHEMBL3384691	1
244	CHEMBL498968	CC(O)CC(O)[C@@H]1CC[C@@H]2[C@H](O)[C@H](O)[C@@H](CO)N12	9.50	CHEMBL1007617	1
245	CHEMBL470868	OC[C@H]1N[C@H](C(O)CCC[C@@H]2CCC[C@]3(CCCCO3)O2)[C@@H](O)[C@@H]1O	0.01	CHEMBL1007617	1

No.	Compound ID	SMILES	IC50 [µM]	ChEMBL Assay ID	Class
246	CHEMBL2011628	OC[C@@H]1[C@H](O)[C@H](O)CCN1CCCCCOCC23CC4CC(CC(C4)C2)C3	6.50	CHEMBL2015864	1
247	CHEMBL2164231	Cl.CCCCCCCCCN[C@H]1[C@H](O)[C@@H](O)[C@H](O)[C@@H](O)[C@H]1OCCCCCCCCC	4.30•10^{-3}	CHEMBL2168210	1
248	CHEMBL3354625	OC[C@@H]1[C@@H](O)[C@H](O)[C@@H](O)CN1CCCCCOCc2ccc(c(F)c2)c3ccccc3	8.00•10^{-5}	CHEMBL3384691	1
249	CHEMBL3354034	OC[C@H]1[C@@H](O)[C@H](O)[C@@H](O)CN1CCCCCOCc2ccccc2	3.00•10^{-3}	CHEMBL3384691	1
250	CHEMBL1922580	Cl.CCCCCCCCN[C@@H]1C=C(CO)[C@@H](O)[C@H](O)[C@H]1O	47.00	CHEMBL1924471	1
251	CHEMBL1076754	CCCCN1C[C@H](O)[C@@H](O)[C@H](O)[C@@H]1CO	0.25	CHEMBL3384691	1
252	CHEMBL511581	COc1ccc(cc1OC)[C@H]2N[C@H](CO)[C@@H](O)[C@@H]2O	34.00	CHEMBL1014583	1
253	CHEMBL3354023	OC[C@@H]1[C@@H](O)[C@H](O)[C@@H](O)CN1CCCCCOCc2cc3ccccc3c4ccccc24	0.10	CHEMBL3384691	1
254	CHEMBL3901083	CCCCCCCCCCCC\N=C/1\SC[C@@H]2[C@@H](O)[C@H](O)[C@@H](CO)N12	1.00	CHEMBL3869409	1
255	CHEMBL3354016	OC[C@@H]1[C@@H](O)[C@H](O)[C@@H](O)CN1CCCCCCOCC23CC4CC(CC(C4)C2)C3	2.00•10^{-3}	CHEMBL3384691	1
256	CHEMBL2204850	CC(=O)O[C@@H]1C=C[C@H]([C@H](OC(=O)C)[C@H]1O)N2CCc3ccccc23	24.30	CHEMBL2216697	1
257	CHEMBL3354623	C[C@@H](OCCCCCN1C[C@H](O)[C@@H](O)[C@H](O)[C@H]1CO)c2ccc(cc2)c3ccccc3	3.00•10^{-3}	CHEMBL3384691	1
258	CHEMBL2011603	OC[C@@H]1[C@@H](O)C=CCN1CCCCCOCC23CC4CC(CC(C4)C2)C3	30.00	CHEMBL2015864	1
259	CHEMBL450985	CCCCCCCCCCCCN1C[C@H](O)[C@@H](O)[C@H](O)[C@H]1CO	2.00•10^{-3}	CHEMBL971353	1
260	CHEMBL2011634	OC[C@@H]1[C@@H](O)[C@@H](O)CCN1CCCCCOCC23CC4CC(CC(C4)C2)C3	25.00	CHEMBL2015864	1
261	CHEMBL186291	CCCCCCCCC1NC(CO)C(O)C(O)C1O	27.00	CHEMBL832785	1
262	CHEMBL3354632	OC[C@H]1[C@@H](O)[C@H](O)[C@@H](O)CN1CC(F)(F)CCCOCc2ccc(cc2)c3ccccc3	2.00•10^{-3}	CHEMBL3384691	1
263	CHEMBL3354051	OC[C@@H]1[C@@H](O)[C@H](O)[C@@H](O)CN1CCCCCOCc2ccc(cc2)c3ccc(Cl)cc3	2.00•10^{-3}	CHEMBL3384691	1
264	CHEMBL1818436	OC[C@@H]1NCC[C@@H](O)[C@H]1O	30.00	CHEMBL2015864	1
265	CHEMBL3354631	OC[C@H]1[C@@H](O)[C@H](O)[C@@H](O)CN1CCCCCOCc2cccc(c2)c3ccccc3	2.00•10^{-3}	CHEMBL3384691	1

No.	Compound ID	SMILES	IC50 [µM]	ChEMBL Assay ID	Class
266	CHEMBL3354060	OC[C@@H]1[C@@H](O)[C@H](O)[C@@H](O)CN1CCCCCOCc2ccc(cc2)c3ccnc(F)c3	$4.00 \bullet 10^{-3}$	CHEMBL3384691	1
267	CHEMBL3354031	CC(C)CCC[C@@H](C)[C@H]1CC[C@H]2[C@@H]3CC=C4C[C@H](CC[C@]4(C)[C@H]3CC[C@]12C)OCCCCCN5C[C@H](O)[C@@H](O)[C@H](O)[C@@H]5CO	0.02	CHEMBL3384691	1
268	CHEMBL3354637	OC[C@H]1[C@@H](O)[C@H](O)[C@@H](O)CN1CCCCCOCc2ccc(c(F)c2)c3ccccc3	$6.00 \bullet 10^{-4}$	CHEMBL3384691	1
269	CHEMBL3359669	OC[C@H]1N\C(=N/Cc2ccccc2)\[C@H](O)[C@@H](O)[C@H]1O	2.80	CHEMBL3815659	1
270	CHEMBL2170199	OC[C@@H]1[C@@H](O)[C@H](O)[C@@H](O)CN1CCCCCC(=O)NC23CC4CC(CC(C4)C2)C3	0.01	CHEMBL3384691	1
271	CHEMBL3354624	OC[C@@H]1[C@@H](O)[C@H](O)[C@@H](O)CN1CCCCCOCc2ccc(cc2F)c3ccccc3	$8.00 \bullet 10^{-4}$	CHEMBL3384691	1
272	CHEMBL3354628	C[C@H](OCCCCCN1C[C@H](O)[C@@H](O)[C@H](O)[C@@H]1CO)c2ccc(cc2)c3ccccc3	$2.00 \bullet 10^{-3}$	CHEMBL3384691	1
273	CHEMBL3354621	OC[C@@H]1[C@@H](O)[C@H](O)[C@@H](O)CN1CCCC(F)(F)COCc2ccc(cc2)c3ccccc3	$1.00 \bullet 10^{-3}$	CHEMBL3384691	1
274	CHEMBL3133388	Cl.CCCCCCCCNC(=N)N1C[C@H](O)[C@@H](O)[C@H](O)[C@H]1CO	25.00	CHEMBL3134886	1
275	CHEMBL2205523	CC(=O)O[C@@H]1C=C[C@H]([C@H](OC(=O)C)[C@H]1O)n2ccc3ccccc23	23.10	CHEMBL2216697	1
276	CHEMBL3901171	OC[C@@H]1[C@@H](O)[C@H](O)[C@@H](O)CN1CCCCc2cn(CCCCOc3ccc4C(=CC(=O)Oc4c3)C(F)(F)F)nn2	3.80	CHEMBL3874807	1
277	CHEMBL3359666	CC(C)(C)c1ccc(C\N=C\2/N[C@H](CO)[C@H](O)[C@H](O)[C@H]2O)cc1	2.30	CHEMBL3815659	1
278	CHEMBL3354046	OC[C@@H]1[C@@H](O)[C@H](O)[C@@H](O)CN1CCCCCOCc2ccc(cc2)c3cccc(c3)C#N	$2.00 \bullet 10^{-3}$	CHEMBL3384691	1
279	CHEMBL3354622	C[C@H](OCCCCCN1C[C@H](O)[C@@H](O)[C@H](O)[C@H]1CO)c2ccc(cc2)c3ccccc3	$3.00 \bullet 10^{-3}$	CHEMBL3384691	1
280	CHEMBL3354063	OC[C@@H]1[C@@H](O)[C@H](O)[C@@H](O)CN1CCCCCOCc2cccc(c2)c3ccccc3	$2.00 \bullet 10^{-3}$	CHEMBL3384691	1
281	CHEMBL3354033	CC(=CCC\C(=C\CC\C(=C\CCOCCCCCN1C[C@H](O)[C@@H](O)[C@H](O)[C@@H]1CO)\C)\C)C	0.10	CHEMBL3384691	1
282	CHEMBL3354636	OC[C@H]1[C@@H](O)[C@H](O)[C@@H](O)CN1CCCCCOCc2ccc(cc2F)c3ccccc3	$4.00 \bullet 10^{-4}$	CHEMBL3384691	1

No.	Compound ID	SMILES	IC50 [µM]	ChEMBL Assay ID	Class
283	CHEMBL3354040	Cc1cccc(c1)c2ccc(COCCCCCN3C[C@H](O)[C@@H](O)[C@H](O)[C@H]3CO)cc2	$2.00 \cdot 10^{-3}$	CHEMBL3384691	1
284	CHEMBL469427	OC[C@H]1N[C@H]([C@H](O)CCCCCCCC(=O)CCCCO[C@@H]2O[C@H](CO)[C@@H](O)[C@H](O)[C@H]2O)[C@@H](O)[C@@H]1O	0.01	CHEMBL1007617	1
285	CHEMBL3359681	OC[C@H]1N\C(=N/CC2CCCCC2)\[C@H](O)[C@@H](O)[C@H]1O	1.60	CHEMBL3815659	1
286	CHEMBL2011611	CCCCN1CC=C[C@H](O)[C@@H]1CO	25.00	CHEMBL2015864	1
287	CHEMBL469437	OCC(O)CC(O)[C@@H]1CC[C@@H]2[C@H](O)[C@H](O)[C@@H](CO)N12	25.40	CHEMBL1007617	1
288	CHEMBL3354039	Cc1ccc(cc1)c2ccc(COCCCCCN3C[C@H](O)[C@@H](O)[C@H](O)[C@H]3CO)cc2	$3.00 \cdot 10^{-3}$	CHEMBL3384691	1
289	CHEMBL1029	CCCCN1C[C@H](O)[C@@H](O)[C@H](O)[C@H]1CO	0.23	CHEMBL1040323, CHEMBL3384691, CHEMBL2015864	1
290	CHEMBL3354056	OC[C@@H]1[C@@H](O)[C@H](O)[C@@H](O)CN1CCCCCOCc2ccc(cc2)c3ccc4OCCCOc4c3	0.02	CHEMBL3384691	1
291	CHEMBL3354037	OC[C@H]1[C@@H](O)[C@H](O)[C@@H](O)CN1CCCCCOCc2ccc3ccc4cccc5ccc2c3c45	$1.00 \cdot 10^{-3}$	CHEMBL3384691	1
292	CHEMBL3359667	OC[C@H]1N\C(=N/Cc2ccccc2F)\[C@H](O)[C@@H](O)[C@H]1O	1.80	CHEMBL3815659	1
293	CHEMBL2011626	OC[C@@H]1[C@@H](O)[C@H](O)CCN1CCCCCOCC23CC4CC(CC(C4)C2)C3	1.00	CHEMBL2015864	1
294	CHEMBL3354633	OC[C@H]1[C@@H](O)[C@H](O)[C@@H](O)CN1CCCC(F)(F)COCc2ccc(cc2)c3ccccc3	$2.00 \cdot 10^{-3}$	CHEMBL3384691	1
295	CHEMBL3354054	OC[C@@H]1[C@@H](O)[C@H](O)[C@@H](O)CN1CCCCCOCc2ccc(cc2)c3ccc4OCOc4c3	$2.00 \cdot 10^{-3}$	CHEMBL3384691	1
296	CHEMBL3354638	OC[C@@H]1[C@H](O)[C@H](O)[C@@H](O)CN1CCCCCOCc2ccc(cc2)c3ccccc3	$2.00 \cdot 10^{-3}$	CHEMBL3384691	1
297	CHEMBL1818433	OC[C@H]1CNC[C@@H](O)[C@H]1O	28.60	CHEMBL1820880, CHEMBL1820877	1
298	CHEMBL3354058	OC[C@@H]1[C@@H](O)[C@H](O)[C@@H](O)CN1CCCCCOCc2ccc(cc2)c3cccnc3	0.01	CHEMBL3384691	1
299	CHEMBL3133391	Cl.OC[C@@H]1[C@@H](O)[C@H](O)[C@@H](O)CN1C(=N)NCc2ccccc2	4.30	CHEMBL3134886	1
300	CHEMBL3354015	OC[C@@H]1[C@@H](O)[C@H](O)[C@@H](O)CN1CCCC\C=C/C23CC4CC(CC(C4)C2)C3	0.01	CHEMBL3384691	1

No.	Compound ID	SMILES	IC50 [µM]	ChEMBL Assay ID	Class
301	CHEMBL3935498	CCCCCCCCCCCCNC(=S)N1[C@H](CO)[C@@H](O)[C@H](O)[C@H]1CO	0.16	CHEMBL3869409	1
302	CHEMBL2029773	Cl.CCCCCCCCCSCC[C@H]1NC[C@@H](O)[C@H](O)[C@H]1O	2.00	CHEMBL2032683	1
303	CHEMBL3359123	OC[C@H]1N\C(=N/Cc2ccc(cc2)C(F)(F)F)\[C@H](O)[C@@H](O)[C@H]1O	2.80	CHEMBL3815659	1
304	CHEMBL2114289	C[C@@H]1CC(NCCc2ccccc2)[C@@H](O)[C@@H](O)[C@@H]1O	0.38	CHEMBL647640	1
305	CHEMBL3354041	Cc1ccccc1c2ccc(COCCCCCN3C[C@H](O)[C@@H](O)[C@H](O)[C@H]3CO)cc2	$5.00 \cdot 10^{-4}$	CHEMBL3384691	1
306	CHEMBL3354027	OC[C@H]1[C@@H](O)[C@H](O)[C@@H](O)CN1CCCCCC(=O)NC23CC4CC(CC(C4)C2)C3	0.02	CHEMBL3384691	1
307	CHEMBL3354030	OC[C@H]1[C@@H](O)[C@H](O)[C@@H](O)CN1CCCCCCC23CC4CC(CC(C4)C2)C3	0.02	CHEMBL3384691	1
308	CHEMBL3907555	CCCCCCCCCCNC(=S)N1[C@H](CO)[C@@H](O)[C@H](O)[C@H]1CO	0.74	CHEMBL3869409	1
309	CHEMBL3354047	OC[C@@H]1[C@@H](O)[C@H](O)[C@@H](O)CN1CCCCCOCc2ccc(cc2)c3ccccc3C#N	0.01	CHEMBL3384691	1
310	CHEMBL3359680	OC[C@H]1N\C(=N/CC2CC2)\[C@H](O)[C@@H](O)[C@H]1O	19.00	CHEMBL3815659	1
311	CHEMBL3354017	OC[C@@H]1[C@@H](O)[C@H](O)[C@@H](O)CN1CCCCCCC23CC4CC(CC(C4)C2)C3	0.01	CHEMBL3384691	1
312	CHEMBL3359671	Cc1cccc(C\N=C\2/N[C@H](CO)[C@H](O)[C@H](O)[C@H]2O)c1	5.10	CHEMBL3815659	1
313	CHEMBL470660	OCCCCC(=O)CCCCCCC[C@@H](O)[C@H]1N[C@H](CO)[C@@H](O)[C@@H]1O	0.01	CHEMBL1007617	1
314	CHEMBL3354026	OC[C@H]1[C@@H](O)[C@H](O)[C@@H](O)CN1CCCCCOCC2CCCCC2	0.01	CHEMBL3384691	1
315	CHEMBL323939	CC1C[C@H](NCCCc2ccccc2)[C@@H](O)C(O)[C@@H]1O	10.20	CHEMBL647640	1
316	CHEMBL3359674	CCCCCCC\N=C\1/N[C@H](CO)[C@H](O)[C@H](O)[C@H]1O	2.20	CHEMBL3815659	1
317	CHEMBL2011612	OC[C@H]1[C@@H](O)C=CCN1CCCCCOCC23CC4CC(CC(C4)C2)C3	10.00	CHEMBL2015864	1
318	CHEMBL3359673	CCC\N=C\1/N[C@H](CO)[C@H](O)[C@H](O)[C@H]1O	4.80	CHEMBL3815659	1
319	CHEMBL3939205	CCCCCCCC\N=C/1\SC[C@@H]2[C@@H](O)[C@H](O)[C@@H](CO)N12	2.40	CHEMBL3869409	1

No.	Compound ID	SMILES	IC50 [μM]	ChEMBL Assay ID	Class
320	CHEMBL3354052	OC[C@@H]1[C@@H](O)[C@H](O)[C@@H](O)CN1CCCCCOCc2ccc(cc2)c3ccc(cc3)C(F)(F)F	0.01	CHEMBL3384691	1
321	CHEMBL291020	O[C@H]1[C@H](O)[C@@H](O)[C@@H]2O[C@@H]2[C@@H]1O	30.00	CHEMBL4007594	1
322	CHEMBL3354020	CC(=CCC\C(=C\CC\C(=C\CCOCCCCCN1C[C@H](O)[C@@H](O)[C@H](O)[C@H]1CO)\C)\C)C	0.10	CHEMBL3384691	1
323	CHEMBL3354019	OC[C@@H]1[C@@H](O)[C@H](O)[C@@H](O)CN1CCCCCOCC2C3CC4CC(CC2C4)C3	$1.00 \cdot 10^{-3}$	CHEMBL3384691	1
324	CHEMBL1086997	CCCCN1C[C@H](O)[C@@H](O)[C@@H](O)[C@H]1CO	0.30	CHEMBL3384691	1

Table S2. The dataset splits into training, validation and test sets.

No.	Compound ID	Class	Set									
			Split1	Split2	Split3	Split4	Split5	Split6	Split7	Split8	Split9	Split10
1	CHEMBL3736317	0	Training	Training	Test	Training	Training	Training	Training	Training	Validation	Training
2	CHEMBL82895	0	Training	Training	Test	Training	Training	Training	Training	Test	Training	Test
3	CHEMBL2011623	0	Training	Test	Training	Training	Validation	Training	Validation	Training	Training	Training
4	CHEMBL84272	0	Validation	Training	Training	Test	Training	Validation	Test	Training	Training	Validation
5	CHEMBL501385	0	Training	Validation	Training	Training	Training	Test	Validation	Training	Validation	Training
6	CHEMBL83278	0	Training	Training	Training	Test	Training	Training	Training	Test	Training	Test
7	CHEMBL3739825	0	Training	Training	Training	Training	Training	Training	Training	Validation	Training	Training
8	CHEMBL2011637	0	Validation	Training	Test	Training	Test	Training	Training	Validation	Training	Training
9	CHEMBL3736333	0	Training	Training	Training	Validation	Test	Training	Test	Training	Test	Test
10	CHEMBL4085739	0	Training	Training	Training	Training	Training	Test	Validation	Training	Training	Training

No.	Compound ID	Class	Set									
			**Split1**	**Split2**	**Split3**	**Split4**	**Split5**	**Split6**	**Split7**	**Split8**	**Split9**	**Split10**
11	CHEMBL186150	0	Training	Training	Test	Training	Validation	Training	Training	Training	Training	Training
12	CHEMBL2011621	0	Training	Training	Training	Test	Training	Training	Training	Training	Test	Training
13	CHEMBL87120	0	Test	Training	Training	Test	Training	Training	Training	Training	Training	Validation
14	CHEMBL2011640	0	Training	Training	Validation	Test	Training	Training	Training	Validation	Training	Training
15	CHEMBL84104	0	Training	Training	Validation	Training	Training	Training	Training	Test	Test	Test
16	CHEMBL2011607	0	Training	Training	Training	Training	Training	Training	Training	Validation	Training	Test
17	CHEMBL362754	0	Training	Training	Test	Training	Training	Test	Test	Validation	Test	Test
18	CHEMBL2011624	0	Training	Training	Training	Training	Training	Validation	Training	Training	Validation	Training
19	CHEMBL2011614	0	Training	Validation	Training	Training	Training	Validation	Test	Training	Training	Training
20	CHEMBL3735749	0	Training	Validation	Test	Training	Training	Training	Validation	Training	Test	Training
21	CHEMBL3754250	0	Training	Training	Training	Training	Training	Training	Training	Validation	Training	Training
22	CHEMBL11216	0	Training	Training	Training	Training	Training	Training	Validation	Test	Training	Validation
23	CHEMBL2011613	0	Training	Training	Validation	Test	Validation	Training	Training	Test	Training	Training
24	CHEMBL2011608	0	Training	Training	Training	Training	Training	Test	Training	Training	Training	Training
25	CHEMBL577370	0	Test	Training	Training	Validation	Training	Validation	Training	Training	Training	Training
26	CHEMBL573995	0	Validation	Training	Training	Training	Training	Training	Training	Training	Training	Training
27	CHEMBL4096086	0	Validation	Test	Training	Training	Training	Test	Test	Test	Validation	Training
28	CHEMBL3736427	0	Training	Validation	Test	Validation	Training	Training	Training	Training	Training	Validation

No.	Compound ID	Class	Set									
			Split1	Split2	Split3	Split4	Split5	Split6	Split7	Split8	Split9	Split10
29	CHEMBL3735899	0	Training	Training	Test	Training	Training	Training	Training	Validation	Training	Training
30	CHEMBL184028	0	Training	Validation	Training	Training	Training	Training	Training	Training	Training	Validation
31	CHEMBL88408	0	Training	Validation	Training	Training	Training	Training	Validation	Training	Training	Test
32	CHEMBL468062	0	Training	Training	Test	Training	Validation	Training	Training	Training	Validation	Training
33	CHEMBL2011638	1	Training	Test	Validation	Training	Training	Training	Training	Test	Test	Validation
34	CHEMBL470659	1	Validation	Validation	Training	Training	Training	Test	Test	Test	Training	Training
35	CHEMBL3354620	1	Training	Validation	Training	Training	Training	Training	Training	Training	Validation	Training
36	CHEMBL115510	1	Training	Training	Training	Training	Training	Training	Test	Test	Training	Training
37	CHEMBL2011642	1	Training	Training	Test	Training	Training	Training	Training	Training	Training	Training
38	CHEMBL324840	1	Validation	Training	Training	Test	Training	Test	Training	Training	Training	Validation
39	CHEMBL186068	1	Training	Training	Training	Training	Training	Training	Training	Training	Training	Validation
40	CHEMBL3354028	1	Validation	Training	Validation	Training	Training	Training	Training	Training	Training	Training
41	CHEMBL3354036	1	Training	Training	Training	Training	Training	Validation	Training	Training	Validation	Training
42	CHEMBL3354014	1	Test	Training	Training	Validation	Training	Validation	Training	Test	Training	Training
43	CHEMBL3815031	1	Training	Training	Training	Test	Test	Training	Training	Test	Validation	Training
44	CHEMBL3354021	1	Training	Training	Validation	Training	Training	Test	Training	Test	Validation	Training
45	CHEMBL3354038	1	Validation	Test	Test	Training	Training	Test	Training	Training	Training	Training
46	CHEMBL2011629	1	Training	Training	Training	Training	Training	Training	Training	Training	Validation	Test

No.	Compound ID	Class	Set									
			Split1	Split2	Split3	Split4	Split5	Split6	Split7	Split8	Split9	Split10
47	CHEMBL513394	1	Test	Training	Training	Training	Validation	Training	Validation	Training	Training	Training
48	CHEMBL326599	1	Training	Test	Validation	Training	Training	Training	Training	Training	Training	Validation
49	CHEMBL3354029	1	Validation	Training	Training	Training	Test	Training	Training	Training	Training	Training
50	CHEMBL2011631	1	Training	Training	Training	Training	Training	Training	Training	Test	Training	Validation
51	CHEMBL3354626	1	Validation	Test	Validation	Training	Training	Test	Validation	Validation	Training	Training
52	CHEMBL86865	1	Validation	Training	Test	Training	Test	Training	Training	Training	Training	Training
53	CHEMBL314757	1	Training	Training	Training	Training	Training	Training	Test	Training	Training	Validation
54	CHEMBL3354022	1	Training	Test	Training	Training	Training	Training	Training	Training	Training	Training
55	CHEMBL3354024	1	Test	Training	Training	Training	Training	Validation	Validation	Training	Training	Validation
56	CHEMBL469844	1	Training	Training	Validation	Test	Training	Training	Test	Test	Validation	Training
57	CHEMBL3354035	1	Test	Training	Test	Training	Training	Training	Validation	Training	Training	Training
58	CHEMBL3359675	1	Training	Training	Training	Validation	Training	Training	Training	Test	Validation	Training
59	CHEMBL3354049	1	Training	Training	Training	Test	Validation	Training	Training	Training	Validation	Training
60	CHEMBL3354630	1	Training	Training	Validation	Training	Validation	Validation	Training	Test	Training	Training
61	CHEMBL3354634	1	Training	Training	Validation	Training	Training	Test	Training	Test	Training	Validation
62	CHEMBL3354018	1	Training	Training	Training	Validation	Validation	Training	Validation	Validation	Validation	Training
63	CHEMBL3972306	1	Training	Training	Validation	Test	Training	Validation	Test	Validation	Training	Test
64	CHEMBL3354055	1	Validation	Training	Training	Training	Test	Training	Validation	Validation	Test	Test

No.	Compound ID	Class	Set									
			Split1	Split2	Split3	Split4	Split5	Split6	Split7	Split8	Split9	Split10
65	CHEMBL9020	0	Validation	Training	Training	Test	Training	Training	Test	Training	Training	Validation
66	CHEMBL2011601	0	Training	Training	Training	Training	Training	Training	Training	Training	Training	Validation
67	CHEMBL356023	0	Training	Training	Training	Training	Training	Training	Training	Training	Test	Training
68	CHEMBL3734817	0	Training	Validation	Validation	Training	Training	Training	Training	Training	Validation	Training
69	CHEMBL2011602	0	Validation	Validation	Training	Training	Training	Training	Training	Training	Training	Test
70	CHEMBL445624	0	Test	Test	Training	Training	Training	Training	Training	Training	Validation	Training
71	CHEMBL511823	0	Training	Training	Validation	Training	Training	Training	Training	Training	Training	Test
72	CHEMBL3980365	0	Validation	Training	Training	Test	Test	Training	Validation	Training	Training	Training
73	CHEMBL313295	0	Training	Training	Test	Training	Training	Training	Training	Training	Test	Test
74	CHEMBL2011610	0	Training	Test	Test	Training	Training	Training	Validation	Training	Training	Training
75	CHEMBL3736274	0	Training	Training	Training	Training	Training	Validation	Validation	Training	Validation	Training
76	CHEMBL2204848	0	Training	Training	Training	Training	Training	Training	Training	Validation	Test	Training
77	CHEMBL2207396	0	Test	Training	Training	Validation	Test	Training	Test	Training	Training	Training
78	CHEMBL2011630	0	Training	Test	Training	Validation	Test	Training	Validation	Training	Training	Training
79	CHEMBL2011633	0	Test	Training	Test	Training	Training	Training	Training	Training	Training	Training
80	CHEMBL275285	0	Training	Validation	Training	Training	Training	Test	Training	Training	Training	Training
81	CHEMBL3970812	0	Training	Training	Training	Training	Validation	Test	Training	Training	Validation	Training
82	CHEMBL369297	0	Training	Training	Training	Training	Training	Test	Training	Test	Test	Training

No.	Compound ID	Class	Set									
			Split1	Split2	Split3	Split4	Split5	Split6	Split7	Split8	Split9	Split10
83	CHEMBL2011605	0	Test	Training	Training	Training	Validation	Validation	Training	Training	Training	Training
84	CHEMBL2011609	0	Validation	Training	Training	Training	Training	Training	Training	Test	Training	Training
85	CHEMBL463872	0	Training	Validation	Training	Training	Training	Training	Training	Validation	Validation	Test
86	CHEMBL86772	0	Training	Validation	Training	Training	Training	Test	Training	Test	Training	Training
87	CHEMBL2011617	0	Training	Training	Training	Training	Training	Training	Validation	Training	Test	Training
88	CHEMBL3735277	0	Validation	Training	Test	Training	Validation	Training	Training	Training	Training	Training
89	CHEMBL152232	0	Validation	Validation	Test	Test	Training	Training	Training	Training	Training	Training
90	CHEMBL421340	0	Test	Training	Training	Training	Training	Training	Training	Training	Training	Training
91	CHEMBL2206827	0	Training	Test	Test	Test	Test	Training	Validation	Training	Training	Test
92	CHEMBL1561	0	Training	Test	Training	Training	Validation	Training	Validation	Validation	Training	Training
93	CHEMBL11516	0	Training	Training	Training	Training	Validation	Training	Training	Training	Training	Training
94	CHEMBL314403	0	Training	Training	Validation	Training	Test	Training	Training	Training	Training	Validation
95	CHEMBL3742008	0	Test	Training	Training	Training	Training	Training	Training	Test	Training	Test
96	CHEMBL507492	0	Test	Training	Validation	Training	Validation	Training	Training	Training	Training	Training
97	CHEMBL313375	0	Training	Training	Training	Training	Training	Validation	Validation	Training	Training	Training
98	CHEMBL1818439	0	Training	Training	Training	Training	Training	Validation	Training	Training	Training	Training
99	CHEMBL574865	0	Training	Training	Training	Training	Test	Training	Training	Test	Training	Training
100	CHEMBL464053	0	Training	Training	Validation	Test	Training	Training	Test	Training	Training	Validation

No.	Compound ID	Class	Set									
			**Split1**	**Split2**	**Split3**	**Split4**	**Split5**	**Split6**	**Split7**	**Split8**	**Split9**	**Split10**
101	CHEMBL1933096	0	Training	Training	Validation	Training	Validation	Training	Training	Test	Test	Training
102	CHEMBL3740658	0	Training	Training	Training	Training	Training	Training	Test	Validation	Test	Training
103	CHEMBL2011620	0	Training	Training	Validation	Test	Validation	Validation	Validation	Training	Training	Training
104	CHEMBL3736474	0	Training	Training	Training	Test	Training	Validation	Training	Training	Training	Training
105	CHEMBL448744	0	Training	Training	Test	Validation	Training	Training	Training	Test	Training	Training
106	CHEMBL3740283	0	Training	Training	Training	Training	Training	Training	Training	Training	Validation	Training
107	CHEMBL3359684	0	Test	Training	Training	Training	Training	Training	Training	Training	Training	Training
108	CHEMBL584580	0	Training	Training	Training	Training	Training	Validation	Training	Training	Training	Training
109	CHEMBL1236649	0	Training	Training	Training	Test	Validation	Training	Test	Validation	Training	Training
110	CHEMBL465105	0	Validation	Training	Training	Test	Training	Training	Validation	Training	Training	Training
111	CHEMBL275509	0	Training	Training	Training	Training	Training	Training	Training	Training	Validation	Validation
112	CHEMBL3133385	0	Training	Training	Validation	Training	Training	Training	Training	Training	Training	Training
113	CHEMBL583176	0	Training	Training	Training	Training	Training	Test	Training	Training	Test	Training
114	CHEMBL503122	0	Training	Training	Training	Training	Training	Test	Training	Training	Training	Training
115	CHEMBL2011618	0	Training	Training	Test	Training	Training	Test	Training	Test	Training	Training
116	CHEMBL502396	0	Test	Test	Training	Training	Test	Training	Validation	Training	Training	Validation
117	CHEMBL1818435	0	Test	Validation	Training	Test	Training	Training	Training	Validation	Test	Training
118	CHEMBL3359683	0	Validation	Training	Training	Training	Test	Test	Training	Training	Validation	Training

No.	Compound ID	Class	Set									
			Split1	Split2	Split3	Split4	Split5	Split6	Split7	Split8	Split9	Split10
119	CHEMBL11477	0	Test	Test	Validation	Validation	Test	Validation	Training	Validation	Training	Validation
120	CHEMBL465034	0	Test	Test	Validation	Training	Validation	Training	Training	Training	Training	Training
121	CHEMBL3133390	0	Validation	Test	Training	Test	Training	Training	Training	Validation	Validation	Training
122	CHEMBL11322	0	Validation	Training	Test	Training	Validation	Training	Training	Training	Test	Training
123	CHEMBL108084	0	Training	Training	Training	Validation	Training	Training	Training	Training	Training	Training
124	CHEMBL505237	0	Training	Training	Training	Training	Training	Test	Training	Training	Training	Training
125	CHEMBL2011622	0	Training	Test	Training	Validation	Training	Training	Training	Training	Training	Training
126	CHEMBL80254	0	Training	Test	Validation	Training	Validation	Training	Training	Training	Training	Training
127	CHEMBL2011639	0	Training	Training	Test	Training	Validation	Training	Training	Training	Test	Training
128	CHEMBL259905	0	Training	Training	Training	Validation	Training	Training	Training	Test	Test	Training
129	CHEMBL3736146	0	Training	Test	Training	Training	Training	Training	Training	Test	Training	Validation
130	CHEMBL11566	0	Training	Validation	Training	Test	Test	Training	Training	Training	Test	Validation
131	CHEMBL3891002	0	Training	Training	Training	Training	Validation	Training	Validation	Validation	Training	Training
132	CHEMBL1566	0	Validation	Training	Test	Training	Training	Validation	Training	Training	Training	Test
133	CHEMBL276643	0	Training	Validation	Training	Training	Training	Training	Test	Training	Test	Test
134	CHEMBL468063	0	Test	Training	Test	Training	Training	Training	Training	Validation	Training	Training
135	CHEMBL456583	0	Training	Training	Training	Test	Test	Validation	Training	Training	Training	Training
136	CHEMBL312653	0	Training	Training	Test	Training	Training	Validation	Training	Training	Training	Training

No.	Compound ID	Class	Set									
			Split1	Split2	Split3	Split4	Split5	Split6	Split7	Split8	Split9	Split10
137	CHEMBL273605	0	Test	Training	Training	Training	Training	Training	Validation	Validation	Training	Validation
138	CHEMBL3742045	0	Training	Test	Training	Training	Training	Training	Training	Training	Training	Training
139	CHEMBL150938	0	Validation	Training	Training	Training	Training	Training	Validation	Training	Training	Test
140	CHEMBL1818321	0	Training	Test	Training	Training	Training	Training	Test	Training	Training	Test
141	CHEMBL3753015	0	Validation	Training	Validation	Test	Training	Training	Validation	Training	Training	Test
142	CHEMBL466791	0	Test	Validation	Training	Training	Training	Test	Training	Training	Training	Training
143	CHEMBL3133386	0	Test	Test	Training	Training	Training	Training	Training	Training	Validation	Training
144	CHEMBL3943978	0	Training	Training	Test	Test	Validation	Training	Training	Training	Test	Test
145	CHEMBL11766	0	Test	Training	Training	Training	Training	Training	Test	Training	Training	Validation
146	CHEMBL3752112	0	Training	Test	Validation	Test	Validation	Test	Training	Training	Training	Training
147	CHEMBL2115197	0	Training	Test	Validation	Training	Training	Training	Test	Training	Validation	Training
148	CHEMBL2207397	0	Validation	Training	Training	Validation	Training	Test	Training	Training	Training	Training
149	CHEMBL3736331	0	Training	Training	Validation	Training	Training	Training	Validation	Training	Validation	Training
150	CHEMBL11452	0	Test	Test	Training	Training	Test	Training	Training	Validation	Training	Test
151	CHEMBL2011616	0	Training	Validation	Training	Training	Test	Training	Training	Test	Training	Training
152	CHEMBL2011619	0	Training	Test	Training	Training	Validation	Test	Test	Training	Training	Training
153	CHEMBL463871	0	Training	Training	Training	Validation	Test	Validation	Training	Test	Training	Validation
154	CHEMBL2011606	0	Training	Training	Training	Training	Training	Validation	Training	Training	Validation	Validation

No.	Compound ID	Class	Set									
			Split1	Split2	Split3	Split4	Split5	Split6	Split7	Split8	Split9	Split10
155	CHEMBL416887	0	Validation	Training	Test	Training	Training	Training	Training	Training	Test	Training
156	CHEMBL2011615	0	Test	Training	Training	Training	Training	Training	Test	Training	Training	Training
157	CHEMBL2206826	0	Training	Training	Training	Validation	Validation	Training	Training	Training	Training	Training
158	CHEMBL3735475	0	Training	Validation	Training	Training	Validation	Training	Training	Training	Training	Validation
159	CHEMBL3359679	0	Training	Validation	Training	Training	Training	Test	Training	Training	Training	Training
160	CHEMBL187158	0	Training	Test	Training	Validation	Training	Test	Training	Training	Training	Training
161	CHEMBL3133389	0	Training	Training	Training	Training	Training	Training	Training	Training	Training	Training
162	CHEMBL1163254	0	Training	Validation	Training	Test	Training	Training	Training	Training	Training	Test
163	CHEMBL1818437	0	Test	Training	Validation	Training	Training	Validation	Training	Training	Training	Training
164	CHEMBL3736234	0	Training	Training	Training	Training	Test	Training	Validation	Training	Test	Test
165	CHEMBL2011625	0	Training	Training	Training	Training	Validation	Test	Training	Training	Training	Training
166	CHEMBL3735278	0	Test	Test	Training	Test	Training	Training	Training	Validation	Training	Training
167	CHEMBL2409320	0	Training	Training	Validation	Validation	Training	Test	Training	Training	Training	Training
168	CHEMBL276030	0	Training	Training	Training	Training	Test	Validation	Training	Test	Training	Training
169	CHEMBL1818434	0	Training	Validation	Training	Training	Training	Validation	Training	Training	Training	Validation
170	CHEMBL2011627	0	Training	Training	Training	Validation	Training	Test	Training	Validation	Training	Training
171	CHEMBL3814915	0	Test	Validation	Validation	Training	Training	Validation	Validation	Training	Training	Validation
172	CHEMBL11614	0	Training	Validation	Test	Training	Validation	Validation	Training	Training	Training	Training

No.	Compound ID	Class	Set									
			Split1	Split2	Split3	Split4	Split5	Split6	Split7	Split8	Split9	Split10
173	CHEMBL3133392	0	Validation	Training	Validation	Training	Test	Validation	Training	Training	Training	Training
174	CHEMBL269410	0	Training	Training	Training	Training	Training	Training	Training	Training	Test	Training
175	CHEMBL445980	0	Training	Training	Training	Training	Validation	Training	Test	Training	Training	Training
176	CHEMBL1818438	0	Training	Training	Test	Training	Training	Training	Training	Validation	Validation	Test
177	CHEMBL2011604	0	Training	Training	Training	Test	Training	Training	Validation	Validation	Training	Training
178	CHEMBL511534	0	Validation	Training	Training	Training	Test	Training	Training	Training	Training	Training
179	CHEMBL84940	0	Training	Training	Training	Training	Test	Training	Test	Validation	Test	Training
180	CHEMBL185536	0	Training	Training	Training	Test	Training	Training	Training	Training	Test	Training
181	CHEMBL1628264	0	Training	Training	Validation	Training	Training	Training	Training	Test	Test	Test
182	CHEMBL3349573	0	Test	Training	Test	Training	Training	Training	Training	Training	Validation	Test
183	CHEMBL357108	0	Training	Test	Training	Training	Validation	Test	Test	Validation	Training	Training
184	CHEMBL88413	0	Training	Training	Training	Validation	Training	Training	Validation	Training	Training	Training
185	CHEMBL1922579	0	Training	Training	Training	Training	Training	Training	Training	Training	Validation	Training
186	CHEMBL368121	0	Test	Training	Training	Training	Validation	Validation	Training	Training	Training	Training
187	CHEMBL454551	0	Training	Test	Training	Validation	Test	Training	Training	Training	Training	Training
188	CHEMBL511366	0	Training	Training	Training	Training	Validation	Training	Training	Training	Validation	Training
189	CHEMBL2011635	0	Validation	Training	Training	Training	Training	Training	Test	Validation	Validation	Training
190	CHEMBL2204849	0	Training	Training	Test	Training	Training	Training	Training	Training	Training	Training

No.	Compound ID	Class	Set									
			Split1	Split2	Split3	Split4	Split5	Split6	Split7	Split8	Split9	Split10
191	CHEMBL3735383	0	Training	Training	Test	Test	Training	Training	Training	Validation	Validation	Test
192	CHEMBL3133393	0	Training	Validation	Validation	Validation	Training	Training	Validation	Validation	Training	Training
193	CHEMBL307429	0	Training	Training	Training	Training	Training	Training	Training	Training	Test	Training
194	CHEMBL414447	0	Training	Training	Training	Test	Test	Training	Training	Validation	Validation	Training
195	CHEMBL117201	1	Training	Training	Training	Training	Training	Test	Training	Training	Validation	Validation
196	CHEMBL3359670	1	Training	Training	Validation	Test	Validation	Test	Test	Training	Training	Validation
197	CHEMBL2011636	1	Training	Training	Training	Training	Test	Training	Training	Training	Test	Training
198	CHEMBL3354044	1	Training	Training	Training	Training	Training	Training	Training	Training	Training	Test
199	CHEMBL35576	1	Test	Validation	Training	Training	Training	Training	Training	Test	Training	Test
200	CHEMBL3359668	1	Validation	Test	Training	Training	Training	Test	Validation	Training	Training	Training
201	CHEMBL117074	1	Training	Training	Training	Training	Test	Test	Training	Test	Training	Training
202	CHEMBL206468	1	Training	Training	Test	Training	Test	Test	Test	Training	Training	Validation
203	CHEMBL470661	1	Training	Test	Training	Training	Training	Training	Test	Training	Validation	Test
204	CHEMBL3359682	1	Validation	Training	Training	Test	Training	Test	Test	Test	Validation	Training
205	CHEMBL3354635	1	Training	Training	Validation	Training	Training	Training	Training	Training	Training	Training
206	CHEMBL3897971	1	Training	Training	Training	Validation	Training	Training	Training	Validation	Training	Training
207	CHEMBL3354043	1	Training	Training	Training	Test	Test	Training	Training	Training	Training	Training
208	CHEMBL3359124	1	Training	Training	Training	Validation	Training	Test	Training	Test	Validation	Training

No.	Compound ID	Class	Set									
			Split1	Split2	Split3	Split4	Split5	Split6	Split7	Split8	Split9	Split10
209	CHEMBL3354064	1	Training	Training	Validation	Training	Training	Training	Training	Training	Training	Training
210	CHEMBL3354061	1	Validation	Training	Training	Validation	Training	Validation	Training	Test	Training	Validation
211	CHEMBL3359677	1	Validation	Training	Test	Training	Validation	Training	Training	Training	Training	Validation
212	CHEMBL3359122	1	Training	Validation	Training	Validation	Test	Test	Training	Test	Training	Training
213	CHEMBL3916914	1	Test	Training	Training	Validation	Training	Training	Training	Training	Training	Test
214	CHEMBL3354042	1	Training	Training	Training	Training	Test	Test	Training	Validation	Test	Training
215	CHEMBL1933098	1	Training	Training	Test	Training	Training	Training	Training	Training	Training	Training
216	CHEMBL3354627	1	Validation	Training	Training	Training	Training	Training	Validation	Test	Training	Training
217	CHEMBL2011641	1	Training	Test	Training	Training	Test	Training	Training	Training	Training	Test
218	CHEMBL3354050	1	Training	Test	Training	Training	Training	Training	Validation	Training	Training	Training
219	CHEMBL116366	1	Validation	Training	Training	Training	Training	Training	Training	Training	Training	Training
220	CHEMBL3359672	1	Training	Training	Training	Test	Validation	Training	Training	Training	Training	Training
221	CHEMBL110830	1	Test	Training	Training	Validation	Training	Training	Training	Training	Training	Training
222	CHEMBL3359678	1	Validation	Test	Test	Training	Training	Training	Validation	Training	Training	Training
223	CHEMBL3354629	1	Test	Test	Training	Training	Training	Training	Validation	Training	Training	Validation
224	CHEMBL469435	1	Training	Training	Validation	Training	Training	Training	Test	Training	Training	Training
225	CHEMBL3354025	1	Training	Training	Training	Training	Training	Training	Training	Test	Training	Training
226	CHEMBL311226	1	Training	Validation	Validation	Validation	Training	Training	Training	Training	Test	Training

No.	Compound ID	Class	Set									
			**Split1**	**Split2**	**Split3**	**Split4**	**Split5**	**Split6**	**Split7**	**Split8**	**Split9**	**Split10**
227	CHEMBL3354053	1	Training	Training	Training	Training	Training	Training	Training	Training	Training	Training
228	CHEMBL3354048	1	Training	Training	Training	Training	Training	Training	Training	Training	Validation	Training
229	CHEMBL364554	1	Training	Training	Training	Training	Training	Training	Training	Training	Validation	Validation
230	CHEMBL3354062	1	Training	Training	Training	Training	Training	Training	Training	Validation	Training	Validation
231	CHEMBL574645	1	Training	Training	Training	Training	Validation	Training	Test	Training	Test	Validation
232	CHEMBL2011632	1	Test	Validation	Test	Training	Training	Test	Training	Validation	Training	Validation
233	CHEMBL3354045	1	Training	Training	Training	Training	Test	Training	Training	Test	Training	Training
234	CHEMBL3354032	1	Training	Training	Training	Test	Training	Training	Training	Test	Training	Training
235	CHEMBL1088158	1	Training	Training	Training	Training	Training	Training	Training	Training	Training	Training
236	CHEMBL3133387	1	Training	Test	Test	Training	Training	Training	Validation	Training	Test	Training
237	CHEMBL3944457	1	Training	Training	Test	Validation	Test	Validation	Training	Test	Validation	Training
238	CHEMBL425308	1	Training	Training	Training	Test	Training	Training	Training	Training	Training	Test
239	CHEMBL485840	1	Training	Training	Validation	Validation	Test	Training	Test	Training	Validation	Test
240	CHEMBL3354057	1	Training	Training	Training	Training	Training	Validation	Test	Training	Test	Training
241	CHEMBL1086996	1	Training	Training	Validation	Validation	Validation	Training	Training	Training	Training	Training
242	CHEMBL4059852	1	Training	Test	Training	Test	Training	Test	Validation	Training	Training	Validation
243	CHEMBL3354059	1	Training	Training	Training	Training	Training	Validation	Test	Training	Training	Validation
244	CHEMBL498968	1	Training	Validation	Training	Test	Training	Training	Training	Training	Test	Training

No.	Compound ID	Class	Set									
			**Split1**	**Split2**	**Split3**	**Split4**	**Split5**	**Split6**	**Split7**	**Split8**	**Split9**	**Split10**
245	CHEMBL470868	1	Training	Training	Training	Training	Test	Training	Training	Training	Training	Training
246	CHEMBL2011628	1	Training	Validation	Training	Training	Test	Training	Training	Training	Training	Training
247	CHEMBL2164231	1	Training	Training	Validation	Training	Training	Training	Training	Training	Training	Test
248	CHEMBL3354625	1	Test	Training	Test	Training	Training	Validation	Validation	Validation	Training	Training
249	CHEMBL3354034	1	Training	Training	Training	Test	Test	Training	Training	Training	Training	Validation
250	CHEMBL1922580	1	Training	Test	Training	Training	Training	Training	Test	Training	Test	Training
251	CHEMBL1076754	1	Training	Training	Training	Training	Training	Training	Training	Validation	Training	Training
252	CHEMBL511581	1	Training	Training	Training	Training	Training	Test	Training	Training	Training	Test
253	CHEMBL3354023	1	Training	Training	Training	Training	Training	Training	Test	Training	Training	Training
254	CHEMBL3901083	1	Training	Training	Training	Training	Training	Training	Training	Training	Test	Training
255	CHEMBL3354016	1	Training	Training	Training	Training	Training	Training	Training	Training	Training	Training
256	CHEMBL2204850	1	Training	Validation	Training	Training	Training	Training	Validation	Training	Training	Training
257	CHEMBL3354623	1	Training	Test	Test	Training	Training	Training	Training	Validation	Training	Training
258	CHEMBL2011603	1	Validation	Training	Training	Test	Validation	Test	Training	Training	Training	Training
259	CHEMBL450985	1	Training	Training	Training	Training	Validation	Training	Training	Training	Training	Training
260	CHEMBL2011634	1	Training	Validation	Training	Training	Test	Validation	Training	Validation	Training	Test
261	CHEMBL186291	1	Training	Validation	Training	Training	Training	Validation	Training	Training	Training	Training
262	CHEMBL3354632	1	Validation	Test	Test	Training	Training	Training	Validation	Validation	Test	Training

No.	Compound ID	Class	Set									
			**Split1**	**Split2**	**Split3**	**Split4**	**Split5**	**Split6**	**Split7**	**Split8**	**Split9**	**Split10**
263	CHEMBL3354051	1	Training	Training	Test	Training	Validation	Test	Training	Training	Training	Training
264	CHEMBL1818436	1	Training	Training	Training	Training	Training	Training	Test	Training	Training	Training
265	CHEMBL3354631	1	Training	Training	Training	Validation	Training	Training	Training	Test	Test	Training
266	CHEMBL3354060	1	Test	Training	Training	Training	Training	Training	Test	Training	Training	Training
267	CHEMBL3354031	1	Training	Training	Training	Training	Validation	Test	Training	Training	Training	Test
268	CHEMBL3354637	1	Training	Training	Test	Training	Training	Validation	Training	Training	Training	Training
269	CHEMBL3359669	1	Training	Training	Training	Validation	Training	Training	Training	Training	Training	Validation
270	CHEMBL2170199	1	Validation	Test	Training	Validation	Training	Validation	Training	Validation	Test	Training
271	CHEMBL3354624	1	Validation	Training	Training	Validation	Training	Validation	Training	Training	Training	Training
272	CHEMBL3354628	1	Training	Validation	Training	Validation	Training	Validation	Test	Training	Test	Training
273	CHEMBL3354621	1	Test	Training	Training	Validation	Training	Training	Training	Validation	Validation	Validation
274	CHEMBL3133388	1	Test	Validation	Training	Training	Test	Training	Test	Training	Training	Training
275	CHEMBL2205523	1	Training	Training	Validation	Training	Training	Test	Training	Training	Training	Training
276	CHEMBL3901171	1	Training	Training	Training	Training	Training	Validation	Test	Validation	Test	Training
277	CHEMBL3359666	1	Test	Training	Training	Validation	Training	Training	Training	Training	Training	Training
278	CHEMBL3354046	1	Training	Training	Training	Training	Validation	Validation	Training	Training	Training	Test
279	CHEMBL3354622	1	Test	Training	Test	Test	Training	Training	Training	Validation	Training	Training
280	CHEMBL3354063	1	Training	Validation	Training	Validation	Training	Training	Training	Training	Training	Training

No.	Compound ID	Class	Set									
			Split1	Split2	Split3	Split4	Split5	Split6	Split7	Split8	Split9	Split10
281	CHEMBL3354033	1	Training	Training	Training	Training	Training	Training	Training	Training	Test	Training
282	CHEMBL3354636	1	Training	Training	Training	Training	Training	Training	Training	Training	Validation	Training
283	CHEMBL3354040	1	Test	Training	Training	Training	Training	Training	Training	Test	Training	Training
284	CHEMBL469427	1	Training	Validation	Training	Training	Training	Test	Training	Training	Training	Training
285	CHEMBL3359681	1	Validation	Test	Training	Training	Test	Training	Training	Training	Training	Training
286	CHEMBL2011611	1	Training	Training	Training	Training	Training	Training	Training	Training	Training	Training
287	CHEMBL469437	1	Training	Training	Training	Training	Validation	Training	Training	Validation	Training	Training
288	CHEMBL3354039	1	Training	Training	Training	Training	Training	Training	Training	Training	Training	Training
289	CHEMBL1029	1	Training	Training	Validation	Validation	Training	Validation	Training	Test	Training	Training
290	CHEMBL3354056	1	Training	Training	Training	Training	Training	Training	Test	Training	Training	Training
291	CHEMBL3354037	1	Training	Training	Training	Validation	Training	Validation	Validation	Training	Training	Training
292	CHEMBL3359667	1	Validation	Training	Training	Training	Training	Training	Training	Training	Training	Validation
293	CHEMBL2011626	1	Test	Training	Training	Validation	Validation	Training	Training	Training	Training	Training
294	CHEMBL3354633	1	Training	Validation	Training	Training	Training	Training	Training	Training	Training	Training
295	CHEMBL3354054	1	Test	Training	Test	Training	Training	Training	Training	Training	Training	Training
296	CHEMBL3354638	1	Validation	Training	Training	Training	Training	Training	Test	Training	Training	Training
297	CHEMBL1818433	1	Test	Training	Training	Training	Test	Training	Training	Training	Training	Training
298	CHEMBL3354058	1	Validation	Training	Training	Validation	Training	Training	Training	Training	Validation	Test

No.	Compound ID	Class	Set									
			**Split1**	**Split2**	**Split3**	**Split4**	**Split5**	**Split6**	**Split7**	**Split8**	**Split9**	**Split10**
299	CHEMBL3133391	1	Training	Test	Training	Training	Training	Training	Training	Training	Test	Training
300	CHEMBL3354015	1	Training	Validation	Validation	Training	Training	Training	Validation	Validation	Training	Training
301	CHEMBL3935498	1	Training	Validation	Training	Test	Training	Training	Training	Training	Validation	Training
302	CHEMBL2029773	1	Validation	Training	Training	Training	Test	Training	Test	Training	Training	Test
303	CHEMBL3359123	1	Training	Training	Training	Training	Training	Training	Validation	Training	Validation	Training
304	CHEMBL2114289	1	Training	Test	Training	Training	Training	Training	Test	Training	Test	Training
305	CHEMBL3354041	1	Training	Training	Validation	Training	Validation	Validation	Training	Test	Training	Test
306	CHEMBL3354027	1	Training	Training	Training	Training	Training	Test	Test	Training	Training	Training
307	CHEMBL3354030	1	Validation	Test	Test	Validation	Training	Validation	Training	Training	Training	Training
308	CHEMBL3907555	1	Validation	Training	Training	Test	Training	Training	Training	Test	Test	Training
309	CHEMBL3354047	1	Training	Training	Training	Training	Training	Training	Training	Training	Training	Training
310	CHEMBL3359680	1	Training	Validation	Validation	Training	Training	Training	Training	Training	Training	Test
311	CHEMBL3354017	1	Test	Test	Training	Training	Validation	Training	Validation	Training	Training	Training
312	CHEMBL3359671	1	Training	Training	Training	Training	Validation	Training	Training	Training	Training	Test
313	CHEMBL470660	1	Training	Training	Validation	Validation	Training	Training	Training	Training	Training	Training
314	CHEMBL3354026	1	Training	Validation	Training	Training	Test	Training	Training	Training	Training	Training
315	CHEMBL323939	1	Test	Training	Training	Training	Training	Training	Training	Training	Training	Training
316	CHEMBL3359674	1	Training	Training	Validation	Validation	Training	Validation	Training	Training	Training	Training

No.	Compound ID	Class	Set									
			Split1	Split2	Split3	Split4	Split5	Split6	Split7	Split8	Split9	Split10
317	CHEMBL2011612	1	Test	Training	Training	Training	Test	Training	Training	Training	Training	Test
318	CHEMBL3359673	1	Training	Training	Training	Training	Training	Training	Validation	Training	Validation	Training
319	CHEMBL3939205	1	Training	Validation	Training	Training	Training	Training	Training	Training	Training	Validation
320	CHEMBL3354052	1	Training	Validation	Training	Test	Training	Training	Test	Training	Training	Training
321	CHEMBL291020	1	Training	Validation	Training	Validation	Training	Training	Training	Test	Training	Training
322	CHEMBL3354020	1	Training	Validation	Training	Training	Training	Training	Training	Training	Training	Validation
323	CHEMBL3354019	1	Training	Training	Training	Training	Test	Validation	Test	Training	Training	Validation
324	CHEMBL1086997	1	Training	Test	Training	Test	Training	Training	Training	Training	Test	Test

Table S3. The results of prediction performed for BIOFACQUIM database (https://biofacquim.herokuapp.com/) using ten selected ANN classifiers employing maxHBint3 and SpMax8_Bhs descriptors. The models were generated using different dataset splits into training validation and test set (supplementary Table S2).

InChIKey	SMILES	maxHBint3	SpMax8_Bhs	Class (predicted)												
				Split1 (RBF 2-24-2)	Split1 (RBF 2-22-2)	Split2 (RBF 2-27-2)	Split3 (MLP 2-4-2)	Split4 (RBF 2-24-2)	Split5 (MLP 2-4-2)	Split5 (RBF 2-22-2)	Split6 (MLP 2-5-2)	Split7 (MLP 2-4-2)	Split8 (RBF 2-27-2)	Split9 (RBF 2-22-2)	Split9 (RBF 2-28-2)	Split10 (RBF 2-30-2)
NPJICTMALKLTFW-OFUAXYCQSA-N	O([C@H]1[C@H](O)[C@@H](O)[C@H](O)[C@@H](CO)O1)[C@@H]1CC=2[C@@](C)([C@@H]3[C@H]([C@H]4[C@@](C)([C@@H]([C@@H](CC[C@H](C(C)C)CC)C)CC4)CC3)CC=2)CC1	3.7903266	3.6483876	1	1	1	1	1	1	1	1	1	1	1	1	1
PEWFWDOPJISUOK-UHFFFAOYSA-N	O(CC(O)C(O)(C)C)c1c2c(occ2)cc2OC(=O)C=Cc12	4.1900093	3.0023078	1	1	1	1	1	1	1	1	1	1	1	1	1

InChIKey	SMILES	maxHBint3	SpMax8_Bhs	Class (predicted)												
				Split1 (RBF 2-24-2)	Split1 (RBF 2-22-2)	Split2 (RBF 2-27-2)	Split3 (MLP 2-4-2)	Split4 (RBF 2-24-2)	Split5 (MLP 2-4-2)	Split5 (RBF 2-22-2)	Split6 (MLP 2-5-2)	Split7 (MLP 2-4-2)	Split8 (RBF 2-27-2)	Split9 (RBF 2-22-2)	Split9 (RBF 2-28-2)	Split10 (RBF 2-30-2)
CETCIHSCOIXIKA-ACIDHAGOSA-N	O(C)[C@H]1OC(=O)C(CC[C@@]2(C)[C@H](C)CC[C@]34C(C(=O)OC3)=C[C@@H](O[C@H]3[C@H](O)[C@@H](O)[C@H](O)[C@@H](CO)O3)CC24)=C1	3.9713325	3.8514536	1	1	1	1	1	1	1	1	1	1	1	1	1
ZVLAQNYZLIHKAE-JLXBEBSSSA-N	O([C@H]1[C@H](O)[C@@H](O)[C@H](O)[C@@H](CO)O1)[C@@H]1C=C2C(=O)OC[C@]32C([C@](CCC=2C(=O)OCC=2)(C)[C@H](C)CC3)C1	3.9960755	3.7468074	1	1	1	1	1	1	1	1	1	1	1	1	1
JMHINZPNTWTGKC-ZREMQXPESA-N	O([C@H]1[C@H](O)[C@@H](O)[C@H](O)[C@@H](CO)O1)[C@@H]1C=C2C(=O)OC[C@]32C([C@](CCC=2C(OC)OC(=O)C=2)(C)[C@H](C)CC3)C1	3.9655117	3.8534047	1	1	1	1	1	1	1	1	1	1	1	1	1
ROSSVNHEVRUXGM-HEMPLKHUSA-N	O([C@H]1[C@H](O)[C@@H](O)[C@H](O)[C@@H](CO)O1)[C@@H]1C=C2C(=O)OC[C@]32C([C@](CCc2cocc2)(C)[C@H](C)CC3)C1	3.9963014	3.673624	1	1	1	1	1	1	1	1	1	1	1	1	1
OOYXHYNAJPWYQC-XRGUOFIRSA-N	O=C(CC[C@@]1(C)[C@H](C)CC[C@]23C(C(=O)OC2)=C[C@@H](O[C@H]2[C@H](O)[C@@H](O)[C@H](O)[C@@H](CO)O2)CC13)C	4.0446445	3.7086575	1	1	1	1	1	1	1	1	1	1	1	1	1
MENXOAAQJIIUMB-OUWQEXSBSA-N	O=C1[C@]2(C)[C@H]3[C@@](O1)(Cc1c(O)c(O)c(C(C)C)c(O)c1C(=O)C3)CCC2	3.9362063	3.3024744	1	1	1	1	1	1	1	1	1	1	1	1	1
MYXNWGACZJSMBT-VJXVFPJBSA-N	O=C1c2c(O)c([C@H]3[C@H](O)[C@@H](O)[C@H](O)[C@@H](CO)O3)c(O)cc2OC(c2ccc(O)cc2)=C1	4.3564731	4.104588	1	1	1	1	1	1	0	1	1	1	1	1	1
SGEWCQFRYRRZDC-VPRICQMDSA-N	O=C1c2c(O)cc(O)c([C@H]3[C@H](O)[C@@H](O)[C@H](O)[C@@H](CO)O3)c2OC(c2ccc(O)cc2)=C1	4.2759883	4.1048288	1	1	1	1	1	1	0	1	1	1	1	1	1
PUPKKEQDLNREIM-QNSQPKOQSA-N	O([C@H]1[C@H](O)[C@H](O)[C@@H](O)[C@H](C)O1)c1cc(O)c2C(=O)C(O[C@H]3[C@H](O)[C@H](O)[C@@H](O)[C@H](C)O3)=C(c3ccc(O)cc3)Oc2c1	4.3636117	4.1725301	1	1	1	1	1	1	0	1	1	1	1	1	1
KMOUJOKENFFTPU-QNDFHXLGSA-N	O([C@H]1[C@H](O)[C@@H](O)[C@H](O)[C@@H](CO)O1)c1cc(O)c2C(=O)C=C(c3ccc(O)cc3)Oc2c1	4.5445839	3.8529669	1	1	1	1	1	1	1	1	0	1	1	1	1
TXVLBRMNUYNGCT-KMEZTADASA-N	O=C(O)[C@@H](NC(=O)[C@@H](O)Cc1ccccc1)C[C@@]1(C(C=C)(C)C)C(=O)Nc2c(O)cccc12	4.6951843	3.7198145	1	1	1	1	1	1	1	1	0	1	1	0	1
QZMIWNADYPPZTF-RLSLOFABSA-N	O=C(O)[C@@H](NC(=O)[C@@H](O)Cc1ccccc1)C[C@@]1(C(C=C)(C)C)C(=O)Nc2c1cccc2	4.5936635	3.657916	1	1	1	1	1	1	1	1	0	1	1	0	1
HGLOWJDVJNISEN-NVXKJEIXSA-N	O([C@@H](C(O)(C)C)C[C@H](C(C)c1cc(O)c(C)cc1)C)[C@H]1[C@H](O[C@@H]2[C@@H](O)[C@H](O)[C@H](O)CO2)[C@@H](O)[C@H](O)[C@@H](CO)O1	4.5774047	4.2931942	1	1	1	1	1	1	0	1	0	1	1	1	0

InChIKey	SMILES	maxHBint3	SpMax8_Bhs	Class (predicted)												
				Split1 (RBF 2-24-2)	Split1 (RBF 2-22-2)	Split2 (RBF 2-27-2)	Split3 (MLP 2-4-2)	Split4 (RBF 2-24-2)	Split5 (MLP 2-4-2)	Split5 (RBF 2-22-2)	Split6 (MLP 2-5-2)	Split7 (MLP 2-4-2)	Split8 (RBF 2-27-2)	Split9 (RBF 2-22-2)	Split9 (RBF 2-28-2)	Split10 (RBF 2-30-2)
BEEAOSMHUSDOTP-NJYVYQBISA-N	O(C)c1c2[C@@H]3[C@@](C)([C@H](O)C(=O)C=C3OC)C=C3C(OC)=CC(=O)c(c(O)c1)c23	3.5014083	3.3296162	0	1	1	1	1	1	1	1	1	1	0	1	0
MWKQZQOBWYIJHX-HSQYWUDLSA-N	O=C(OC[C@@]1(C)[C@H]2[C@@](C)(c3c(O)c(O)c(C(C)C)cc3C(=O)C2)CCC1)C	3.4727061	3.1884696	0	1	1	1	1	1	1	1	1	1	0	1	0
OLPZITFOWYCLMF-VUAAKQTLSA-N	O([C@@H](CCCC)[C@H]1OC(=O)C=C(OC)C1)[C@H]1[C@@H](O)[C@@H](OC)[C@H](OC)[C@@H](CO)O1	2.8740567	3.4228696	1	0	1	1	0	1	1	1	1	1	0	0	0
HGUQYROCEMPGDU-BWIMDNAMSA-N	O=C(c1cc(O)c(O)cc1)C1=C(O)[C@@](C/C=C(/CC/C=C(\C)/C)\C)(C/C=C(\C)/C)C(=O)C=2[C@H](C3=C(O)C(=O)C=CO3)CC(C)(C)OC1=2	5.0032721	3.7438947	1	1	0	1	0	1	0	1	0	1	1	0	1
HGUQYROCEMPGDU-YJMRDKADSA-N	O=C(c1cc(O)c(O)cc1)C1=C(O)[C@](C/C=C(/CC/C=C(\C)/C)\C)(C/C=C(\C)/C)C(=O)C=2[C@H](C3=C(O)C(=O)C=CO3)CC(C)(C)OC1=2	5.0032721	3.7438947	1	1	0	1	0	1	0	1	0	1	1	0	1
GUNXKIKUNNXJKN-NKLJSFOLSA-N	O([C@@H]1[C@H](O)[C@H](O)[C@@H](CO)O1)c1c(O)c2c(c(O)c1)C(=O)O[C@H](C)C2	4.764957	3.608092	1	1	0	1	0	1	1	1	0	1	1	0	0
COWWBPRRBQOULA-NABHEWRGSA-N	O=C(O[C@@H]1[C@@H](OC(=O)C)[C@](O)(C)C(=O)/C=C\[C@H](C)C[C@H]2OC(=O)C(=C)[C@H]12)C(=C)C	2.8872924	3.472496	1	0	1	1	0	1	1	1	1	1	0	0	0
YXBNQTGKGCNTPV-VCEHAEOKSA-N	O([C@H]([C@H](OC(=O)C[C@@H](C(=O)O)CC(=O)O)[C@@H](CCCC)C)[C@H](C[C@H](O)CCCC[C@@H](O)C[C@H](O)[C@@H](N)C)C)C(=O)C[C@@H](C(=O)O)CC(=O)O	3.9444891	4.5294797	0	0	1	0	1	1	0	0	1	1	1	1	0
MXKKFADFYXJREN-TXZJYACMSA-N	O[C@H]1[C@@]2(c3ccc(O)cc3)Oc3c(c(O)cc(O)c3)[C@@H]1c1c3O[C@@H]([C@@H](O)Cc3c(O)cc1O2)c1cc(O)c(O)cc1	5.3370494	4.0628552	1	1	0	1	0	1	0	1	0	1	1	0	0
PRRUXSDCUPUYKC-OPMOSEIQSA-N	O=C(O)CCCCCCCCCC(O[C@H]1[C@H](O[C@H]2[C@H](O[C@H]3[C@H](O)[C@H](O)[C@@H](O[C@H]4[C@H](O)[C@@H](O)[C@H](O)[C@@H](C)O4)[C@H](C)O3)[C@@H](O)[C@H](C)[C@@H](CO)C2)[C@@H](O)[C@H](O)[C@@H](C)O1)CCCCC	3.8990962	4.5601131	0	0	1	0	1	1	0	0	1	1	1	1	0
DXPFQBFCOCKNEU-RMCHFIGYSA-N	O=C(O[C@@H]1[C@@H](OC(=O)C)[C@](O)(C)C(=O)/C=C\[C@H](C)C[C@H]2OC(=O)C(=C)[C@H]12)/C(=C/C)/C	2.744074	3.4856932	1	0	1	1	0	1	1	1	1	0	0	0	0

InChIKey	SMILES	maxHBint3	SpMax8_Bhs	Class (predicted)												
				Split1 (RBF 2-24-2)	Split1 (RBF 2-22-2)	Split2 (RBF 2-27-2)	Split3 (MLP 2-4-2)	Split4 (RBF 2-24-2)	Split5 (MLP 2-4-2)	Split5 (RBF 2-22-2)	Split6 (MLP 2-5-2)	Split7 (MLP 2-4-2)	Split8 (RBF 2-27-2)	Split9 (RBF 2-22-2)	Split9 (RBF 2-28-2)	Split10 (RBF 2-30-2)
DXPFQBFCOCKNEU-MBWLHKRJSA-N	O=C(O[C@@H]1[C@@H](OC(=O)C)[C@](O)(C)C(=O)/C=C\[C@H](C)C[C@H]2OC(=O)C(=C)[C@H]12)/C(=C\C)/C	2.744074	3.4856932	1	0	1	1	0	1	1	1	1	0	0	0	0
KNCNSYQYJUBLPW-NRIIMPDMSA-N	O(C[C@@H]1[C@@H](O)[C@H](O)[C@@H](O)[C@H](Oc2c3C(c4cc(O)c(O)cc4)=CC(=O)Oc3cc(OC)c2)O1)[C@H]1[C@H](O)[C@@](O)(CO)CO1	5.5039059	4.1915842	1	1	0	1	0	1	0	1	1	1	0	0	0
BDIGJZNTLJAZGG-YVGDPVPUSA-N	O([C@H](C(=O)[C@@H]1[C@@H]([C@H](O)[C@@H]2OC(=O)C=CC2)C1)C)C(=O)/C=C/c1cc(O)c(OC)cc1	2.7088141	3.4368901	1	0	1	1	0	1	1	1	1	0	0	0	0
VEBNYMXKXIIGFX-IYVYCCGLSA-N	O=C(O[C@H]1C(O)[C@H](OC(=O)/C=C/c2cc(O)c(O)cc2)CC(O)(C(=O)OC)C1)/C=C/c1cc(O)c(O)cc1	5.7403871	4.0162625	1	1	0	1	0	1	0	1	1	1	0	0	0
XSUATNGSAZMWRU-ZYAFLGLPSA-N	O(C[C@@H]1[C@@H](O)[C@H](O)[C@@H](O)[C@H](Oc2c3C(c4cc(O)c(O)cc4)=CC(=O)Oc3cc(O)c2)O1)[C@H]1[C@H](O)[C@@H](O)[C@H](O)CO1	5.4985719	4.2520596	1	1	0	1	0	1	0	1	1	1	0	0	0
GXDJGKMWLJOJFR-WUSKNVGPSA-N	O(C[C@@H]1[C@@H](O)[C@H](O)[C@@H](O)[C@H](Oc2c3C(c4cc(O)c(O)cc4)=CC(=O)Oc3cc(OC)c2)O1)[C@H]1[C@H](O)[C@@H](O)[C@H](O)CO1	5.4607117	4.1982119	1	1	0	1	0	1	0	1	1	1	0	0	0
DDGYXSWHWAQNRQ-MGPUTAFESA-N	O(C)c1c2occc2cc2C(=O)[C@]3(O)[C@@H](Oc12)COc1c3cc2OCOc2c1	3.0278088	3.1004699	0	0	1	1	0	1	1	1	1	1	0	0	0
OVSQVDMCBVZWGM-DTGCRPNFSA-N	O([C@H]1[C@H](O)[C@@H](O)[C@@H](O)[C@@H](CO)O1)C1=C(c2cc(O)c(O)cc2)Oc2c(c(O)cc(O)c2)C1=O	5.6584515	4.1659039	1	1	0	1	0	1	0	1	1	1	0	0	0
OVSQVDMCBVZWGM-QSOFNFLRSA-N	O([C@H]1[C@H](O)[C@@H](O)[C@H](O)[C@@H](CO)O1)C1=C(c2cc(O)c(O)cc2)Oc2c(c(O)cc(O)c2)C1=O	5.6584515	4.1659039	1	1	0	1	0	1	0	1	1	1	0	0	0
OXGUCUVFOIWWQJ-HQBVPOQASA-N	O([C@H]1[C@H](O)[C@H](O)[C@@H](O)[C@H](C)O1)C1=C(c2cc(O)c(O)cc2)Oc2c(c(O)cc(O)c2)C1=O	5.6560832	4.0941001	1	1	0	1	0	1	0	1	1	1	0	0	0
PCWHVEGCZALKKT-LPLXGARPSA-N	O=C(OC[C@@H]1[C@H](O)[C@H](O)[C@@H](O)[C@H](OC2=C(c3cc(O)c(O)cc3)Oc3c(c(O)cc(O)c3)C2=O)O1)c1ccccc1	5.5446613	4.0517946	1	1	0	1	0	1	0	1	1	1	0	0	0
KALBJZVIHLVACU-YNUABKSXSA-N	O=C(O)CCCCCCCCC[C@@H](O[C@H]1[C@H](O[C@@H]2[C@@H](O[C@@H]3[C@@H](O)[C@@H](O)[C@H](O[C@H]4[C@H](O)[C@@H](O)[C@@H](O)[C@@H](C)O4)[C@@H](C)O3)[C@H](O)[C@@	4.000093	4.5965153	0	0	1	0	1	1	0	0	1	1	1	1	0

InChIKey	SMILES	maxHBint3	SpMax8_Bhs	Class (predicted)												
				Split1 (RBF 2-24-2)	Split1 (RBF 2-22-2)	Split2 (RBF 2-27-2)	Split3 (MLP 2-4-2)	Split4 (RBF 2-24-2)	Split5 (MLP 2-4-2)	Split5 (RBF 2-22-2)	Split6 (MLP 2-5-2)	Split7 (MLP 2-4-2)	Split8 (RBF 2-27-2)	Split9 (RBF 2-22-2)	Split9 (RBF 2-28-2)	Split10 (RBF 2-30-2)
	H](O)[C@H](CO)O2)[C@@H](O)[C@H](O)[C@@H](C)O1)CCCC															
TYWCXSPTQQXINE-ROMNNNSQSA-N	O=C(O)CCCCCCCCC[C@@H](O[C@H]1[C@H](O[C@@H]2[C@@H](O[C@@H]3[C@@H](O)[C@@H](O)[C@H](O[C@H]4[C@H](O)[C@@H](O)[C@@H](O)[C@@H](C)O4)[C@@H](C)O3)[C@H](O)[C@@H](O)[C@H](CO)O2)[C@@H](O)[C@H](O)[C@@H](C)O1)CCCCCC	3.9700293	4.5923334	0	0	1	0	1	1	0	0	1	1	1	1	0
KOQREMYYHAAAAT-HBRPDFDGSA-N	O=C(O)CCCCCCCCC[C@@H](O[C@H]1[C@H](O[C@H]2[C@H](O[C@H]3[C@H](O)[C@H](O)[C@@H](O)[C@H](C)O3)[C@@H](O)[C@H](O)[C@@H](CO[C@H]3[C@H](O)[C@@H](O)[C@H](O)[C@@H](C)O3)O2)[C@@H](O)[C@H](O)[C@@H](C)O1)CCC	3.9643494	4.5980296	0	0	1	0	1	1	0	0	1	1	1	1	0
SHUQHAMYYBXIIQ-GCSSGBNASA-N	O=C(O)CCCCCCCCC[C@@H](O[C@H]1[C@H](O[C@H]2[C@H](O[C@H]3[C@H](O)[C@H](O)[C@@H](O)[C@H](C)O3)[C@@H](O[C@H]3[C@H](O)[C@@H](O)[C@H](O)[C@@H](C)O3)[C@H](O)[C@@H](C)O2)[C@@H](O)[C@H](O)[C@@H](C)O1)CCC	3.6795757	4.5649097	0	0	1	0	1	1	0	0	1	1	1	1	0
HNCIHRVMBZWZSI-XSFVSMFZSA-N	O=C(c1cc(O)c(O)cc1)C=1C(=O)C(C/C=C(/CC/C=C(\C)/C)\C)(C/C=C(\C)/C)C(=O)C23OC2C(O)C(C)(C)OC=13	5.1596105	3.6977392	1	1	0	1	0	1	0	1	0	1	1	0	0
KEQANZVCLOIYPL-KGENOOAVSA-N	O=C(c1cc(O)c(O)cc1)C=1C(=O)C(C/C=C(/CC/C=C(\C)/C)\C)(C/C=C(\C)/C)C(=O)C23OC2CC(C)(C)OC=13	5.1587588	3.6282038	1	1	0	1	0	1	0	1	0	1	1	0	0
LLSDPOXYUGFOKC-UHFFFAOYSA-N	O(C)c1c(Oc2c(OC)cc(C=3Oc4c(c(O)c(O)c(OC)c4)C(=O)C=3)cc2)ccc(C=2Oc3c(c(O)c(O)c(OC)c3)C(=O)C=2)c1	5.3602905	3.8760504	1	1	0	1	0	1	0	1	0	1	1	0	0
MRFHAFKKJKWCOY-KPHUOKFYSA-N	O=C1[C@]2(C)[C@@H]3[C@@H](O1)C=C1C(=O)C(C(C)C)=C(O)C(=O)C1=CC3=CCC2	5.2641958	3.2821237	1	1	0	1	0	1	1	1	0	1	0	0	0
UJZKICMAGUFXSG-NTCOEUGSSA-N	O=C1[C@]2(C)[C@@H]3[C@H]([C@@H](O)C=4C(=O)C(C(C)C)=C(O)C(=O)C=4C=C3C=CC2)O1	5.2438436	3.2935225	1	1	0	1	0	1	1	1	0	1	0	0	0
JTCUWDQRUWSZLA-PSCQKVGZSA-N	O=C1[C@]2(C)[C@@H]3[C@H]([C@@H](O)C=4C(=O)C(C(C)C)=C(O)C(=O)C=4C=C3[C@H](O)CC2)O1	5.1488827	3.4728533	1	1	0	1	0	1	1	1	0	1	0	0	0

InChIKey	SMILES	maxHBint3	SpMax8_Bhs	Class (predicted)												
				Split1 (RBF 2-24-2)	Split1 (RBF 2-22-2)	Split2 (RBF 2-27-2)	Split3 (MLP 2-4-2)	Split4 (RBF 2-24-2)	Split5 (MLP 2-4-2)	Split5 (RBF 2-22-2)	Split6 (MLP 2-5-2)	Split7 (MLP 2-4-2)	Split8 (RBF 2-27-2)	Split9 (RBF 2-22-2)	Split9 (RBF 2-28-2)	Split10 (RBF 2-30-2)
LIOHNCKMCBYMAR-RAJNIJHNSA-N	O=C1[C@]2(C)[C@H]3C([C@H](O)CC2)=CC=2C(=O)C(O)=C(C(C)C)C(=O)C=2C=C3O1	5.2764994	3.3202979	1	1	0	1	0	1	1	1	0	1	0	0	0
DXPFQBFCOCKNEU-AUNCTZJHSA-N	O=C(O[C@@H]1[C@@H](OC(=O)C)[C@](O)(C)C(=O)/C=C\[C@H](C)C[C@H]2OC(=O)C(=C)[C@H]12)C(=CC)C	2.744074	3.4856932	1	0	1	1	0	1	1	1	1	0	0	0	0
OBNVTTHRWSUAMB-IAPIXIRKSA-N	OC(C(O)CC[C@@H](C)c1cc(O)c(C)cc1)(C)C	3.8434112	2.7869589	1	0	0	0	0	0	1	1	1	1	0	0	1
OFCHTGCBFRTISG-VUEDXXQZSA-N	O(C)c1c(O)ccc([C@H]2OC[C@@H]3[C@@H](c4c(C)c5OCOc5cc4)OC[C@H]23)c1	2.7675752	3.1063376	0	0	1	1	0	1	1	1	1	0	0	0	0
KRZBCHWVBQOTNZ-RDJMKVHDSA-N	O=C(O[C@H]1C(O)[C@H](OC(=O)/C=C/c2cc(O)c(O)cc2)CC(O)(C(=O)O)C1)/C=C/c1cc(O)c(O)cc1	5.9190073	4.0344231	1	0	0	1	0	1	0	1	1	1	0	0	0
GXIDPDIXZHCJHP-KMJCNKHZSA-N	O=C(O)CCCCCCCCC[C@@H](O[C@H]1[C@H](O[C@@H]2[C@@H](O[C@H]3[C@H](O)[C@H](O)[C@@H](O)[C@H](C)O3)[C@H](O)[C@@H](O)[C@H](CO[C@H]3[C@H](O[C@H]4[C@H](O)[C@H](O)[C@@H](O)[C@H](C)O4)[C@@H](O)[C@H](O)[C@@H](C)O3)O2)[C@@H](O)[C@H](O)[C@@H](C)O1)CCC	3.8279567	4.6691361	0	0	1	0	1	1	0	0	1	1	0	1	0
GLAAQZFBFGEBPS-UHFFFAOYSA-N	O(C)c1c(O)c2C(=O)C=C(c3cc(OC)c(O)cc3)Oc2cc1O	2.9154703	3.2440023	0	0	1	1	0	1	1	1	1	0	0	0	0
DRRWBCNQOKKKOL-UHFFFAOYSA-N	O(C)c1c(O)c2C(=O)C=C(c3cc(OC)c(OC)cc3)Oc2cc1O	2.6953913	3.2188402	0	0	1	1	0	1	1	1	1	0	0	0	0
HRQRRWLXKJXUIZ-RHMWYWNKSA-N	O(Oc1c2C(c3cc(O)c(O)cc3)=CC(=O)Oc2cc(O)c1)[C@H]1[C@H](O)[C@@H](O)[C@H](O)CO1	5.7049326	3.8641136	1	1	0	1	0	1	0	1	0	1	0	0	0
BDIFYHLEBJTHAL-LWRIQDNHSA-N	O(Oc1c2C(c3cc(O)c(O)cc3)=CC(=O)Oc2cc(OC)c1)[C@@H]1[C@H](O)[C@@H](O)[C@@H](CO)CO1	5.6390884	3.6459913	1	1	0	1	0	1	0	1	0	1	0	0	0
GBPGQCGEJYVXSH-ZQEFQCJFSA-N	O(Oc1c2C(c3cc(O)c(O)cc3)=CC(=O)Oc2cc(OC)c1)[C@H]1[C@H](O)[C@@H](O)[C@H](O)CO1	5.6699678	3.6361762	1	1	0	1	0	1	0	1	0	1	0	0	0
KQFUXLQBMQGNRT-UHFFFAOYSA-N	O(C)c1c(O)ccc(C2=C(OC)C(=O)c3c(O)cc(OC)cc3O2)c1	2.8801674	3.2058188	0	0	1	1	0	1	1	1	1	0	0	0	0
JABJSHMLHVMIMA-UMGXQCJCSA-N	O=C(c1cc(O)c(O)cc1)C=1C(=O)[C@@](C/C=C(/CC/C=C(\C)/C)\C)(C/C=C(\C)/C)C(=O)C=1O	5.5104833	3.6066337	1	1	0	1	0	1	0	1	0	1	0	0	0
CVULDJMCSSACEO-VMPREFPWSA-N	O=C(OC[C@@H](NC(=O)c1ccccc1)Cc1ccccc1)[C@@H](NC(=O)c1ccccc1)Cc1ccccc1	5.0480311	3.3509887	0	1	0	1	0	1	1	1	0	1	0	0	0

InChIKey	SMILES	maxHBint3	SpMax8_Bhs	Class (predicted)												
				Split1 (RBF 2-24-2)	Split1 (RBF 2-22-2)	Split2 (RBF 2-27-2)	Split3 (MLP 2-4-2)	Split4 (RBF 2-24-2)	Split5 (MLP 2-4-2)	Split5 (RBF 2-22-2)	Split6 (MLP 2-5-2)	Split7 (MLP 2-4-2)	Split8 (RBF 2-27-2)	Split9 (RBF 2-22-2)	Split9 (RBF 2-28-2)	Split10 (RBF 2-30-2)
DWZAJFZEYZIHPO-UHFFFAOYSA-N	O(C)c1c(O)c2C(=O)C(OC)=C(c3ccc(OC)cc3)Oc2cc1O	2.6857472	3.2245559	0	0	1	1	0	1	1	1	1	0	0	0	0
GRUVGBVSXJLAOU-FDQSAEMMSA-N	O=C(O[C@H]1C=2C(=O)C(C(C)C)=C(O)C(=O)C=2[C@]2(C)[C@H]([C@](CO)(C)CCC2)C1)C	4.3194456	3.3983911	0	0	1	1	1	1	0	1	1	0	0	0	0
UYYYJFGICXAUGO-HAGHYFMRSA-N	O[C@@H]1[C@@]2(O[C@H]3[C@](O)(c4c(c(C)ccc4)O3)CC2)c2c(O1)cc(C)cc2	2.292104	2.994007	0	0	1	1	0	1	1	1	0	0	0	0	0
OWQLBLNRUZULFV-UHFFFAOYSA-N	O(C)c1c(O)cc(O)c2C(=O)C(OC)=C(c3ccccc3)Oc12	2.8197639	2.9140849	0	0	1	1	0	1	1	1	0	0	0	0	0
ZKXIUVREPAJEBX-AYVHDSGLSA-N	O=C(O)CCCCCCCCC[C@@H](O[C@H]1[C@H](O[C@]2(O)[C@@H](O)[C@H](O[C@H]3[C@H](O[C@H]4[C@H](O)[C@H](O)[C@@H](O)[C@H](C)O4)[C@H](O[C@H]4[C@H](O)[C@H](O)[C@@H](O)[C@H](C)O4)[C@H](O)[C@@H](C)O3)[C@@H](C)CO2)[C@@H](O)[C@H](O)[C@@H](C)O1)CCC	3.6084651	4.6365227	0	0	1	0	1	1	0	0	1	1	0	0	0
IPMAKCYEUXFJTI-UHFFFAOYSA-N	O(C)c1c(O)c(OC)cc2c1-c1c(c(OC)c(O)cc1)CC2	2.7154375	2.9158993	0	0	1	1	0	1	1	1	0	0	0	0	0
LLRLPRUFUGWYOL-UHFFFAOYSA-N	O(C)c1c(O)c(OC)cc2c1c1c(c(OC)c(O)cc1)cc2	2.7787525	2.9281619	0	0	1	1	0	1	1	1	0	0	0	0	0
MBNGWHIJMBWFHU-UHFFFAOYSA-N	O(C)c1c(O)cc(C=2Oc3c(c(O)cc(O)c3)C(=O)C=2)cc1	2.74924	2.9933127	0	0	1	1	0	1	1	1	0	0	0	0	0
WJKXLOGUCSSDAB-JGVFFNPUSA-N	O(C)C=1[C@H](O)[C@@](O)(C)CC(=O)C=1	4.9580372	1.9295527	0	0	1	0	1	0	0	0	0	1	1	0	1
XUZGFXBHALEMTN-BIIVOSGPSA-N	Cl[C@@H]1[C@](O)(C)[C@@H](O)C(OC)=CC1=O	4.7161082	1.9437728	0	1	1	0	1	0	0	0	0	0	1	0	1
HUCKMCHVBAWQIA-DGBHNKMOSA-N	O=C(O[C@@H]1[C@@H](O[C@H]2[C@H](O)[C@H](O)[C@@H](O)[C@H](C)O2)[C@H](OC[C@@H]2[C@@H](O)[C@H]3[C@@H](O[C@H]4[C@H](OC(=O)/C(=C/C)/C)[C@H](O)[C@@H](O)[C@H](C)O4)[C@@H](O2)O[C@@H]2[C@@H](O)[C@H](O)[C@@H](C)O[C@H]2O[C@@H](CCCCC)CCCCCCCCCC(=O)O3)O[C@H](C)[C@H]1O)/C(=C/C)/C	3.3988562	4.5457118	0	0	1	0	1	1	0	0	1	1	0	0	0
QMGPOYYFOFDFEO-JPYSVIHJSA-N	O([C@@H](C)[C@H]1O[C@@H]([C@H](O)[C@@H]2OC(=O)C=CC2)CC1)C(=O)/C=C/c1cc(O)c(O)cc1	5.7904448	3.5287192	1	0	0	1	0	1	0	1	0	1	0	0	0

InChIKey	SMILES	maxHBint3	SpMax8_Bhs	Class (predicted)												
				Split1 (RBF 2-24-2)	Split1 (RBF 2-22-2)	Split2 (RBF 2-27-2)	Split3 (MLP 2-4-2)	Split4 (RBF 2-24-2)	Split5 (MLP 2-4-2)	Split5 (RBF 2-22-2)	Split6 (MLP 2-5-2)	Split7 (MLP 2-4-2)	Split8 (RBF 2-27-2)	Split9 (RBF 2-22-2)	Split9 (RBF 2-28-2)	Split10 (RBF 2-30-2)
NLJQJJGYCFVOFQ-GCOVDLRRSA-N	O=C(OC)[C@@H](NC(=O)[C@@H](O)Cc1ccccc1)C[C@@]1(C(C=C)(C)C)C(=O)Nc2c(O)cccc12	4.6687358	3.5604555	1	0	0	1	0	1	1	1	0	0	0	0	0
VMKCIRAJEVFSFR-LQTXRJQHSA-N	O=C1[C@H](Cc2ccccc2)OC(=O)[C@H]2N1[C@]1(C(C=C)(C)C)[C@@](O)(c3c(c(O)ccc3)N1)C2	2.429806	3.2973842	0	0	1	1	0	1	1	1	0	0	0	0	0
HHYNJJSTUPNMQU-RUGJYQHNSA-N	O=C(O[C@H]1C=2C(=O)C(C(C)C)=C(O)C(=O)C=2C[C@]23OC(=O)[C@](C)([C@@H]2C1)CCC3)C	4.7583113	3.4422997	0	0	0	1	0	1	1	1	0	1	0	0	0
XXVSZSNOEWVDPA-KPHUOKFYSA-N	O=C1[C@]2(C)[C@@H]3[C@@H](O1)CC=1C(=O)C(C(C)C)=C(O)C(=O)C=1CC3=CCC2	5.0694989	3.2810153	0	1	0	1	0	1	1	1	0	0	0	0	0
NFLLWTNAXDSKKC-MRFFXTKBSA-N	O=C1[C@]2(C)[C@H]3[C@@](O1)(CC=1C(=O)C(O)=C(C(C)C)C(=O)C=1C=C3)CCC2	5.0627859	3.1729319	0	1	0	1	0	1	1	1	0	0	0	0	0
KJWFOHVSTFGWGZ-YCDQCDSPSA-N	O=C(O[C@@H]1[C@H](O)c2c3OC(=O)C=Cc3ccc2OC1(C)C)/C(=C\C)/C	1.6041667	2.9528447	0	0	1	1	0	1	0	1	0	0	0	0	0
QPLSCFLMIOADPA-RQJZHBNNSA-N	O=C(O[C@H]1[C@@H](O)C(C)(C)Oc2c1c1OC(=O)C=Cc1cc2)/C(=C\C)/C	1.7889826	3.0964895	0	0	1	1	0	1	0	1	0	0	0	0	0
JHCPPFWOQPOFRF-HZPDHXFCSA-N	O=C(O[C@H]1[C@@H](O)C(C)(C)Oc2c1c1OC(=O)C=Cc1cc2)C(C)C	1.8063349	3.1128955	0	0	1	1	0	1	0	1	0	0	0	0	0
GIVSZLKTIBWYRM-UHFFFAOYSA-N	O(C)c1c(OC)c2c3c(c(OC)cc2cc1O)cc(O)cc3	2.6437906	2.8351501	0	0	1	1	0	0	1	1	0	0	0	0	0
YTRAYUIKLRABOQ-UHFFFAOYSA-N	O(C)c1c(O)c(OC)cc(CCc2cc(OC)c(O)cc2)c1	2.9016353	2.8335147	0	0	1	1	0	0	1	1	0	0	0	0	0
MRYYPHKLLLULDL-UHFFFAOYSA-N	O(C)c1c(OC)c2c3c(O)cccc3c(OC)cc2cc1O	2.6356783	2.7649583	0	0	1	1	0	0	1	1	0	0	0	0	0
LJJIBKCHKKEYAS-OIDCBOCKSA-N	O=C(O)CCCCCCCCCC(O[C@H]1[C@H](O[C@H]2[C@H](O)[C@H](O)[C@@H](O[C@H]3[C@H](O)[C@H](O[C@H]4[C@H](O)[C@@H](O)[C@H](O)[C@@H](CO)O4)[C@@H](O[C@H]4[C@H](O)[C@H](O)[C@@H](O)[C@H](C)O4)[C@H](C)O3)[C@H](C)O2)[C@@H](O)[C@H](O)[C@@H](CO)O1)CCCCC	3.7128111	4.7213372	0	0	0	0	1	1	0	0	1	1	0	0	0
LTAIPRWVZLMEEJ-WOFCNMKLSA-N	O=C(O)CCCCCCCCCC(O[C@H]1[C@H](O[C@H]2[C@H](O[C@H]3[C@H](O)[C@H](C[C@H]4[C@H](O[C@H]5[C@H](O)[C@@H](O)[C@H](O)[C@@H](C)O5)[C@@H](O)[C@H](O)[C@@H](CO)O4)[C@@H](O[C@H]4[C@H](O)[C@@H](O)[C@@H](O)[C@@H](C)O4)[C@H](C)O3)[C@@H](O)[C@H](O)[C@	3.4798881	4.7261104	0	0	0	0	1	1	0	0	1	1	0	0	0

InChIKey	SMILES	maxHBint3	SpMax8_Bhs	Class (predicted)												
				Split1 (RBF 2-24-2)	Split1 (RBF 2-22-2)	Split2 (RBF 2-27-2)	Split3 (MLP 2-4-2)	Split4 (RBF 2-24-2)	Split5 (MLP 2-4-2)	Split5 (RBF 2-22-2)	Split6 (MLP 2-5-2)	Split7 (MLP 2-4-2)	Split8 (RBF 2-27-2)	Split9 (RBF 2-22-2)	Split9 (RBF 2-28-2)	Split10 (RBF 2-30-2)
	@H](CO)O2)[C@@H](O)[C@H](O)[C@@H](C)O1)CCC															
XVZMUMHZDANJSG-VTKNXRICSA-N	O=C(O)CCCCCCCCCC(O[C@H]1[C@H](O[C@H]2[C@H](O[C@H]3[C@H](O)[C@H](C[C@H]4[C@H](O[C@H]5[C@H](O)[C@@H](O)[C@H](O)[C@@H](C)O5)[C@@H](O)[C@H](O)[C@@H](CO)O4)[C@@H](O[C@H]4[C@H](O)[C@@H](O)[C@@H](O)[C@@H](C)O4)[C@H](C)O3)[C@@H](O)[C@H](O)[C@@H](CO)O2)[C@@H](O)[C@H](O)[C@@H](C)O1)CCCCC	3.4498856	4.7215459	0	0	0	0	1	1	0	0	1	1	0	0	0
KHEBRRJCDZMMMT-VUEVMRPESA-N	O=C(OC)CCCCCCCCCC(O[C@H]1[C@H](O[C@H]2[C@H](O[C@H]3[C@H](O)[C@H](C[C@H]4[C@H](O[C@H]5[C@H](O)[C@@H](O)[C@H](O)[C@@H](C)O5)[C@@H](O)[C@H](O)[C@@H](CO)O4)[C@@H](O[C@H]4[C@H](O)[C@@H](O)[C@@H](O)[C@@H](C)O4)[C@H](C)O3)[C@@H](O)[C@H](O)[C@@H](CO)O2)[C@@H](O)[C@H](O)[C@@H](C)O1)CCC	3.4718093	4.7002339	0	0	0	0	1	1	0	0	1	1	0	0	0
XEDWBBOLVGMERH-NCOBWLNYSA-N	O=C(OC)CCCCCCCCCC(O[C@H]1[C@H](O[C@H]2[C@H](O[C@H]3[C@H](O)[C@H](C[C@H]4[C@H](O[C@H]5[C@H](O)[C@@H](O)[C@H](O)[C@@H](C)O5)[C@@H](O)[C@H](O)[C@@H](CO)O4)[C@@H](O[C@H]4[C@H](O)[C@@H](O)[C@@H](O)[C@@H](C)O4)[C@H](C)O3)[C@@H](O)[C@H](O)[C@@H](CO)O2)[C@@H](O)[C@H](O)[C@@H](C)O1)CCCCC	3.4417675	4.6967751	0	0	0	0	1	1	0	0	1	1	0	0	0
UTXKXSRRVOYQSF-UHFFFAOYSA-N	O(C)c1c(OC)cc(CCc2cc(OC)c(O)cc2)cc1O	2.9032787	2.8329359	0	0	1	1	0	0	1	1	0	0	0	0	0
WZUVPPKBWHMQCE-XJKSGUPXSA-N	Oc1c(O)ccc2[C@@H]3[C@](O)(COc12)Cc1c3cc(O)c(O)c1	5.6278909	3.2055271	1	0	0	1	0	1	0	0	0	1	0	0	0
QFYNDCRFXVUINR-XGICHPGQSA-N	O=C(OCC(O)(COC(=O)C)c1c(O)cc(C)cc1)/C(=C\C)/C	1.5388517	2.9972852	0	0	1	1	0	1	0	1	0	0	0	0	0
CQXZARCGOSILEP-WCIBSUBMSA-N	O=C(Oc1c(C2(CO)OC2)ccc(C)c1)/C(=C\C)/C	2.8868246	2.810013	0	0	1	1	0	0	1	1	0	0	0	0	0

InChIKey	SMILES	maxHBint3	SpMax8_Bhs	Class (predicted)												
				Split1 (RBF 2-24-2)	Split1 (RBF 2-22-2)	Split2 (RBF 2-27-2)	Split3 (MLP 2-4-2)	Split4 (RBF 2-24-2)	Split5 (MLP 2-4-2)	Split5 (RBF 2-22-2)	Split6 (MLP 2-5-2)	Split7 (MLP 2-4-2)	Split8 (RBF 2-27-2)	Split9 (RBF 2-22-2)	Split9 (RBF 2-28-2)	Split10 (RBF 2-30-2)
CWVRJTMFETXNAD-JUHZACGLSA-N	O=C(O[C@H]1[C@H](O)[C@H](O)C[C@@](O)(C(=O)O)C1)/C=C/c1cc(O)c(O)cc1	6.2630461	3.983565	0	0	0	1	0	1	0	1	0	1	0	0	0
CUCFSOKADUSMPF-ZANVPECISA-N	O(C)c1c(C)c2[C@](O)(C)[C@H](CO)OC(=O)c2c(O)c1	2.8027767	2.853764	0	0	1	1	0	0	1	1	0	0	0	0	0
HDTRYLNUVZCQOY-LIZSDCNHSA-N	O([C@@H]1[C@H](O)[C@@H](O)[C@H](O)[C@@H](CO)O1)[C@@H]1[C@H](O)[C@@H](O)[C@H](O)[C@@H](CO)O1	4.6768456	4.8552447	0	1	0	1	0	1	0	0	0	1	0	0	0
OPIUYBQHWXRHKP-HNKATWAKSA-N	O=C(O)CCCCCCCCC[C@H](O[C@H]1[C@H](O[C@H]2[C@H](O[C@H]3[C@H](O)[C@H](O[C@@H]4[C@@H](O[C@@H]5[C@@H](O)[C@H](O)[C@@H](O)[C@H](C)O5)[C@H](O)[C@@H](O)[C@H](CO)O4)[C@@H](O[C@H]4[C@H](O)[C@@H](O)[C@@H](O)[C@@H](C)O4)[C@H](C)O3)[C@@H](O)[C@H](O)[C@@H](CO)O2)[C@@H](O)[C@H](O)[C@@H](C)O1)CCCC	3.5846609	4.7261105	0	0	0	0	1	1	0	0	1	1	0	0	0
LSULNWMZUOBWRA-NIYMFQSSSA-N	O=C(O)CCCCCCCCC[C@H](O[C@H]1[C@H](O[C@H]2[C@H](O[C@H]3[C@H](O)[C@H](O[C@@H]4[C@@H](O[C@@H]5[C@@H](O)[C@H](O)[C@@H](O)[C@H](C)O5)[C@H](O)[C@@H](O)[C@H](CO)O4)[C@@H](O[C@H]4[C@H](O)[C@@H](O)[C@@H](O)[C@@H](C)O4)[C@H](C)O3)[C@@H](O)[C@H](O)[C@@H](CO)O2)[C@@H](O)[C@H](O)[C@@H](C)O1)CCCCCC	3.5571838	4.7215459	0	0	0	0	1	1	0	0	1	1	0	0	0
OXOOTMOEABRPHG-BBYXOMEOSA-N	O=C(O)C[C@@H](CCCCCCC[C@H](O[C@H]1[C@H](O[C@H]2[C@H](O[C@H]3[C@H](O)[C@H](O[C@@H]4[C@@H](O[C@@H]5[C@@H](O)[C@H](O)[C@@H](O)[C@H](C)O5)[C@H](O)[C@@H](O)[C@H](CO)O4)[C@@H](O[C@H]4[C@H](O)[C@@H](O)[C@@H](O)[C@@H](C)O4)[C@H](C)O3)[C@@H](O)[C@H](O)[C@@H](CO)O2)[C@@H](O)[C@H](O)[C@@H](C)O1)CCCCCC)[C@@H]1[C@H](O)[C@@H](O)[C@H](O)[C@@H](C)O1	3.5014139	4.7439431	0	0	0	0	1	1	0	0	1	1	0	0	0
GOZCEKPKECLKNO-RKQHYHRCSA-N	O=C(C)c1ccc(O[C@H]2[C@H](O)[C@@H](O)[C@H](O)[C@@H](CO)O2)cc1	4.7187918	3.0179641	0	1	0	1	0	1	1	0	0	0	0	0	0

InChIKey	SMILES	maxHBint3	SpMax8_Bhs	Class (predicted)												
				Split1 (RBF 2-24-2)	Split1 (RBF 2-22-2)	Split2 (RBF 2-27-2)	Split3 (MLP 2-4-2)	Split4 (RBF 2-24-2)	Split5 (MLP 2-4-2)	Split5 (RBF 2-22-2)	Split6 (MLP 2-5-2)	Split7 (MLP 2-4-2)	Split8 (RBF 2-27-2)	Split9 (RBF 2-22-2)	Split9 (RBF 2-28-2)	Split10 (RBF 2-30-2)
DEEHWRGXKJRSMV-WMOYMDCYSA-N	O([C@@H](C)[C@H]1O[C@@H]([C@H](O)[C@@H]2OC(=O)C=CC2)CC1)C(=O)/C=C/c1ccc(O)cc1	2.0260775	3.2652193	0	0	1	1	0	1	0	1	0	0	0	0	0
PRXVDDVCKQCKQP-PPCLKGCXSA-N	O([C@@H](C)[C@H]1O[C@@H]([C@H](O)[C@@H]2OC(=O)C=CC2)CC1)C(=O)/C=C/c1ccc(OC)cc1	2.0121785	3.2437656	0	0	1	1	0	1	0	1	0	0	0	0	0
DEEHWRGXKJRSMV-SJFPRCBQSA-N	O([C@@H](C)[C@H]1O[C@@H]([C@H](O)[C@@H]2OC(=O)C=CC2)CC1)C(=O)/C=C\c1ccc(O)cc1	2.0260775	3.2652193	0	0	1	1	0	1	0	1	0	0	0	0	0
PRXVDDVCKQCKQP-IZONMWRVSA-N	O([C@@H](C)[C@H]1O[C@@H]([C@H](O)[C@@H]2OC(=O)C=CC2)CC1)C(=O)/C=C\c1ccc(OC)cc1	2.0121785	3.2437656	0	0	1	1	0	1	0	1	0	0	0	0	0
PHWNDHXZDCXMCU-AWEZNQCLSA-N	O(C)c1c2C(=O)OC[C@]3(O)C(OC)=CC(=O)c(c(O)c1)c23	1.7346355	3.0485766	0	0	1	1	0	1	0	1	0	0	0	0	0
QIEMGQKOGFTYLN-UHFFFAOYSA-N	O(C)c1c(OC)ccc(C=2Oc3c(c(O)c(O)c(OC)c3)C(=O)C=2)c1	5.6062571	3.1890035	1	0	0	1	0	1	0	0	0	1	0	0	0
PNCQKGVAPWZYRN-BGERDNNASA-N	O=C(O)[C@@H](NC(=O)C(O)Cc1ccccc1)Cc1c(C(C=C)(C)C)[nH]c2c1cccc2	4.6746709	3.3451022	0	0	0	1	0	1	1	1	0	0	0	0	0
JLUYABBECFTSKK-AGEKVHLVSA-N	O=C(O[C@@]12[C@@](C(C=C)(C)C)(N3C(=O)[C@H](Cc4ccccc4)OC(=O)[C@@H]3C1)Nc1c(O)cccc21)C	2.1130493	3.3686879	0	0	1	1	0	1	0	1	0	0	0	0	0
GBMUTTKBDFEONI-OTLGMJNYSA-N	O=C1[C@H](Cc2ccccc2)OC(=O)[C@H]2N1[C@]1(C(C=C)(C)C)[C@@](O)(c3c(N1)cccc3)C2	2.0480482	3.1779348	0	0	1	1	0	1	0	1	0	0	0	0	0
HWCRIILCNXONSY-SYMKQFHVSA-N	O=C1C(C(C)C)=C(O)C(=O)C2=C1[C@H](O)C[C@H]1[C@@]3(C)CO[C@@H](O)[C@@]21CCC3	4.5565909	3.3195719	0	0	0	1	0	1	1	1	0	0	0	0	0
SCDGKJFOGSRPQB-JGZYSILTSA-N	O([C@H]1[C@H](O)[C@@H](O)[C@H](O)[C@@H](CO)O1)c1c2OC(=O)C=C(OC)c2c(C)cc1	4.5974708	3.4668603	0	0	0	1	0	1	1	1	0	0	0	0	0
IZQSVPBOUDKVDZ-UHFFFAOYSA-N	O(C)c1c(O)ccc(C2=C(O)C(=O)c3c(O)cc(O)cc3O2)c1	6.3439052	3.2680766	0	0	0	1	0	1	0	0	0	1	0	0	0
REFJWTPEDVJJIY-UHFFFAOYSA-N	O=C1C(O)=C(c2cc(O)c(O)cc2)Oc2c1c(O)cc(O)c2	6.481414	3.4002899	0	0	0	1	0	1	0	0	0	1	0	0	0
BJDMHAYLPGRUFH-HIELJHRRSA-N	O([C@H](C(=O)[C@@H]1[C@@H]([C@H](O)[C@@H]2OC(=O)C=CC2)C1)C)C(=O)/C=C/c1ccc(O)cc1	1.7317013	3.4234648	0	0	1	0	0	1	0	1	0	0	0	0	0
XGNLXIVLBLLTIG-FMRMVBGOSA-N	O([C@H](C(=O)[C@@H]1[C@@H]([C@H](O)[C@@H]2OC(=O)C=CC2)C1)C)C(=O)/C=C/c1ccc(OC)cc1	1.7168647	3.4119511	0	0	1	0	0	1	0	1	0	0	0	0	0
UIWQVTWVFQWDQI-IQOMITAASA-N	O([C@H](C(=O)[C@@H]1[C@@H]([C@H](O)[C@@H]2OC(=O)C=CC2)C1)C)C(=O)/C=C\c1cc(O)ccc1	1.7313179	3.4235972	0	0	1	0	0	1	0	1	0	0	0	0	0

InChIKey	SMILES	maxHBint3	SpMax8_Bhs	Class (predicted)												
				Split1 (RBF 2-24-2)	Split1 (RBF 2-22-2)	Split2 (RBF 2-27-2)	Split3 (MLP 2-4-2)	Split4 (RBF 2-24-2)	Split5 (MLP 2-4-2)	Split5 (RBF 2-22-2)	Split6 (MLP 2-5-2)	Split7 (MLP 2-4-2)	Split8 (RBF 2-27-2)	Split9 (RBF 2-22-2)	Split9 (RBF 2-28-2)	Split10 (RBF 2-30-2)
CJJVWWCXCQUGER-UAZACNSMSA-N	O=C(O[C@H]1c2c(c(O)cc(C)c2)C(O)=C2C(=O)[C@@H]3c4c5O[C@@]6(C(=O)OC)[C@H](O)C=CC(O)=C6C(=O)c5c(O)cc4C[C@]12C=C3)C	1.7193557	4.1925824	0	0	0	0	0	1	0	0	1	1	0	0	0
YKQYIQHWWYVPHK-CPFSXVBKSA-N	O(C)c1c(C)c2[C@@]([C@@H](O)C)(C)OC(=O)c2c(O)c1	2.2781082	2.8158915	0	0	1	1	0	0	0	1	0	0	0	0	0
MFJMOCPZLBNSPY-ZDUSSCGKSA-N	O(C)c1c(CO)c2[C@](O)(C)C(=C)OC(=O)c2c(O)c1	1.768894	2.800376	0	0	1	1	0	0	0	1	0	0	0	0	0
QHWDTDHKZFTJKU-PYOFXGKSSA-N	O=C(O[C@@H]1[C@@H](O)[C@H](O[C@@H]2[C@@H](O)[C@H](O)[C@@H](CO)O[C@H]2O[C@H]2[C@@H](OC(=O)[C@H](CC)C)[C@H](O[C@@H]3[C@@H]4[C@H](O)[C@@H](COC(=O)C)O[C@H]3O[C@@H]3[C@@H](O)[C@H](O)[C@@H](C)O[C@H]3O[C@@H](CCC)CCCCCCC[C@H](O[C@@H]3[C@@H](O)[C@H](O)[C@@H](O)[C@H](C)O3)CC(=O)O4)O[C@@H](C)[C@@H]2O[C@H]2[C@H](O)[C@@H](OC(=O)CCCCCCCCCCC)[C@@H](O)[C@@H](C)O2)O[C@H](C)[C@H]1O)[C@H](CC)C	3.008058	4.6980549	0	0	0	0	0	1	0	0	1	1	0	0	0
NKXXQFOOOHJLRQ-DYFOXTGPSA-N	O=C(O[C@@H]1[C@@H](OC(=O)C(CC)C)[C@H](C)O[C@@H](O[C@@H]2[C@@H](O[C@H]3[C@H](O)[C@H](O)[C@@H](O)[C@H](C)C3)[C@@H](O)[C@H](O[C@@H]3[C@@H](O)[C@@H](O)[C@H](O[C@@H]4[C@@H](O)[C@@H](O)[C@@H](C)O[C@H]4O[C@H](CCCCCCCCCC(=O)O[C@@H]4[C@H](O)[C@@H](O)[C@H](C)O[C@H]4O[C@H]4[C@@H](OC(=O)CCCCCCCCCCC)[C@H](O[C@H]5[C@H](C)O[C@H]6O[C@@H]7[C@@H](O)[C@@H](O)[C@@H](C)O[C@H]7O[C@@H](CCCCC)CCCCCCCCCC(=O)O[C@@H]6[C@@H]5O)O[C@@H](C)[C@@H]4O[C@H]4[C@H](O)[C@H](O)[C@H](OC(=O)C(CC)C)[C@H](C)O4)CCCCC)O[C@H]3C)O[C@H]2C)[C@@H]1O)/C=C/c1ccccc1	2.283096	4.6435376	0	0	0	0	0	1	0	0	1	1	0	0	0
YFMMGUARCLTHRY-CJYYLSOKSA-N	O=C(O[C@H]1[C@@H](O)[C@@H](O)[C@H](O[C@@H]2[C@@H](O[C@H]3[C@H](O)[C@H](O)[C@@H](OC(=O)CCCCCCCCC[C@@H](O[C@H]4[C@H	2.045433	4.6954726	0	0	0	0	0	1	0	0	1	1	0	0	0

InChIKey	SMILES	maxHBint3	SpMax8_Bhs	Class (predicted)												
				Split1 (RBF 2-24-2)	Split1 (RBF 2-22-2)	Split2 (RBF 2-27-2)	Split3 (MLP 2-4-2)	Split4 (RBF 2-24-2)	Split5 (MLP 2-4-2)	Split5 (RBF 2-22-2)	Split6 (MLP 2-5-2)	Split7 (MLP 2-4-2)	Split8 (RBF 2-27-2)	Split9 (RBF 2-22-2)	Split9 (RBF 2-28-2)	Split10 (RBF 2-30-2)
	](O[C@H]5[C@H](O)[C@H](O)[C@@H](O[C@H]6[C@H](OC(=O)CCCCCCCCC)[C@H](O[C@H]7[C@H](O)[C@H](O)[C@@H](O)[C@H](C)C7)[C@@H](O[C@H]7[C@H](O)[C@H](O)[C@@H](OC(=O)[C@@H](CC)C)[C@H](C)O7)[C@H](C)O6)[C@H](C)O5)[C@@H](O)[C@@H](O)[C@@H](C)O4)CCCCC)[C@H](C)O3)[C@@H](OC(=O)CCCCCCCCC)[C@H](O[C@H]3[C@H](C)O[C@@H]4[C@H](O)[C@@H]3OC(=O)CCCCCCCCC[C@H](CCCCC)O[C@@H]3O[C@H](C)[C@H](O)[C@H](O)[C@H]3O4)O[C@H]2C)O[C@H]1C)[C@@H](CC)C															
HKLDKRBGASBJRS-SYGNNDERSA-N	O=C(O[C@H]1[C@@H](O)[C@@H](O)[C@H](O[C@@H]2[C@@H](O[C@H]3[C@H](O)[C@H](O)[C@@H](OC(=O)CCCCCCCCC[C@@H](O[C@H]4[C@H](O[C@H]5[C@H](O)[C@H](O)[C@@H](O[C@H]6[C@H](OC(=O)CCCCCCCCCCC)[C@H](O[C@H]7[C@H](O)[C@H](O)[C@@H](O)[C@H](C)C7)[C@@H](O[C@H]7[C@H](O)[C@H](O)[C@@H](OC(=O)[C@@H](CC)C)[C@H](C)O7)[C@H](C)O6)[C@H](C)O5)[C@@H](O)[C@@H](O)[C@@H](C)O4)CCCCC)[C@H](C)O3)[C@@H](OC(=O)CCCCCCCCCCC)[C@H](O[C@H]3[C@H](C)O[C@@H]4[C@H](O)[C@@H]3OC(=O)CCCCCCCCC[C@H](CCCCC)O[C@@H]3O[C@H](C)[C@H](O)[C@H](O)[C@H]3O4)O[C@H]2C)O[C@H]1C)[C@@H](CC)C	1.9877383	4.6960086	0	0	0	0	0	1	0	0	1	1	0	0	0
DTEIVMDGEUSUOI-IKIOFCMYSA-N	O=C(O[C@H]1[C@@H](O)[C@@H](O)[C@H](O[C@@H]2[C@@H](O[C@H]3[C@H](OC(=O)CCCCCCCCC[C@@H](O[C@H]4[C@H](O[C@H]5[C@H](O)[C@H](O)[C@@H](O[C@H]6[C@H](OC(=O)CCCCCCCCCCC)[C@H](O[C@H]7[C@H](O)[C@H](O)[C@@H](O)[C@H](C)C7)[C@@H](O[C@H]7[C@H](O)[C@H](O)[C@@H](OC(=O)C(C)C)[C@H](C)O7)[C@H](C)O6)[C@H](C)O5)[C@@H](O)[C@@H](O)[C@@H](C)O4)CCCCC)[C@H](O)[C@@H](O)[C@H](C)O3)[C@@H](OC(=O)CCCCCCCCCCC)[C@H](O[C@	1.9983086	4.6957449	0	0	0	0	0	1	0	0	1	1	0	0	0

InChIKey	SMILES	maxHBint3	SpMax8_Bhs	Class (predicted)												
				Split1 (RBF 2-24-2)	Split1 (RBF 2-22-2)	Split2 (RBF 2-27-2)	Split3 (MLP 2-4-2)	Split4 (RBF 2-24-2)	Split5 (MLP 2-4-2)	Split5 (RBF 2-22-2)	Split6 (MLP 2-5-2)	Split7 (MLP 2-4-2)	Split8 (RBF 2-27-2)	Split9 (RBF 2-22-2)	Split9 (RBF 2-28-2)	Split10 (RBF 2-30-2)
	H]3[C@H](C)O[C@H]4O[C@@H]5[C@@H](O)[C@@H](O)[C@@H](C)O[C@H]5O[C@@H](CCCCC)CCCCCCCCCC(=O)O[C@@H]4[C@@H]3O)O[C@H]2C)O[C@H]1C)C(C)C															
CHWPMFMUQATVNK-IUCIJCHOSA-N	O[C@@H]1[C@]2(C(=C)C)O[C@@H]2[C@]2(C)[C@@H](C)[C@H](O)CCC2=C1	1.9241552	2.7553226	0	0	1	1	0	0	0	1	0	0	0	0	0
ABOXHMBRQLJXDS-QFONWEIGSA-N	O=C(O[C@@H]1[C@@H](C)O[C@@H]2[C@H](O[C@H]3[C@H](O)[C@H](O)[C@@H](O)[C@H](C)O3)[C@H]1O[C@H]1[C@H](O)[C@@H]([C@@H](O)[C@H](C)O1)OC(=O)CCCCCCCCC[C@H](CCC)O[C@@H]1O[C@H](C)[C@@H](O)[C@H](O)[C@H]1O[C@H]1[C@H](OC(=O)/C(=C/C)/C)[C@H](O)[C@H]([C@H](C)O1)O2)/C(=C/C)/C	3.1373486	4.4748331	0	0	0	0	0	1	0	0	1	1	0	0	0
AFFBKAFUWKADJM-RFDHAYLPSA-N	O=C(O[C@@H]1[C@H](O)[C@@H](O)[C@H](C)O[C@H]1O[C@@H]1[C@@H](O)[C@H](O)[C@@H]2O[C@H]1O[C@@H]1[C@@H](O)[C@H](O)[C@@H](C)O[C@H]1O[C@@H](CCC)CCCCCCCCCC(=O)O[C@@H]1[C@H](O)[C@@H](O)[C@H](C)O[C@H]1O[C@@H]1[C@@H](O)[C@H](O)[C@@H](C)O[C@H]1OC2)/C(=C/C)/C	3.0199128	4.5040865	0	0	0	0	0	1	0	0	1	1	0	0	0
GJTLOGFPCMLBGM-QZEDAVLZSA-N	O=C(O[C@@H]1[C@H](O)[C@@H](O)[C@H](C)O[C@H]1O[C@@H]1[C@@H]2[C@H](O)[C@@H](C)O[C@H]1O[C@@H]1[C@@H](O)[C@@H](OC(=O)/C(=C/C)/C)[C@@H](O[C@H]1C)O[C@@H]1[C@@H](O)[C@H](O)[C@@H](C)O[C@H]1O[C@@H](CCC)CCCCCCCCCC(=O)O[C@@H]1[C@@H](O)[C@H](C)O[C@H]([C@@H]1O)O2)[C@@H]([C@H](O)C)C	2.6519106	4.5611632	0	0	0	0	0	1	0	0	1	1	0	0	0
UIWUPBBHRVAQMV-ZVKYRITESA-N	O=C(O[C@@H]1[C@H](O)[C@@H](O[C@H]2[C@H](O)[C@@H](O)[C@@H](OC(=O)/C(=C/C)/C)[C@@H](C)O2)[C@H](C)O[C@H]1O[C@@H]1[C@@H]2[C@H](O)[C@@H](CO)O[C@H]1O[C@@H]1[C@@H](O)[C@H](O)[C@@H](C)O[C@H]1O[C@@H](CCCC)CCCCCCCCCC(=O)O2)/C(=C/C)/C	2.4468485	4.4751044	0	0	0	0	0	1	0	0	1	1	0	0	0

InChIKey	SMILES	maxHBint3	SpMax8_Bhs	Class (predicted)												
				Split1 (RBF 2-24-2)	Split1 (RBF 2-22-2)	Split2 (RBF 2-27-2)	Split3 (MLP 2-4-2)	Split4 (RBF 2-24-2)	Split5 (MLP 2-4-2)	Split5 (RBF 2-22-2)	Split6 (MLP 2-5-2)	Split7 (MLP 2-4-2)	Split8 (RBF 2-27-2)	Split9 (RBF 2-22-2)	Split9 (RBF 2-28-2)	Split10 (RBF 2-30-2)
OHIGTDSMWCKQMO-YNTSURPRSA-N	O=C(O[C@@H]1[C@H](O)[C@@H](O[C@H]2[C@H](O)[C@@H](O)[C@@H](OC(=O)/C(=C/C)/C)[C@@H](C)O2)[C@H](C)O[C@H]1O[C@@H]1[C@@H]2[C@H](O)[C@@H](CO)O[C@H]1O[C@@H]1[C@@H](O)[C@H](O)[C@@H](C)O[C@H]1O[C@@H](CCCCCC)CCCCCCCCCC(=O)O2)/C(=C/C)/C	2.4067223	4.4791573	0	0	0	0	0	1	0	0	1	1	0	0	0
IZXAJJIHCIQBDV-QZAYOBNPSA-N	O=C(O[C@H]1[C@H](C)O[C@H]2O[C@@H]3[C@@H](O)[C@H](O)[C@@H](C)O[C@H]3OC[C@@H]3[C@@H](O)[C@H](O)[C@@H](O[C@H]4[C@H](O)[C@H](O)[C@@H](OC(=O)C)[C@H](C)O4)[C@H](O[C@@H]4[C@@H](O)[C@H](O)[C@@H](C)O[C@H]4O[C@@H](CCC)CCCCCCCCCC(=O)O[C@@H]2[C@@H]1O)O3)[C@@H]([C@H](O)C)C	2.5931188	4.6297613	0	0	0	0	0	1	0	0	1	1	0	0	0
OXPMXNOQWPDRHJ-GBOYLNFRSA-N	O=C(O[C@H]1[C@H](O)[C@@H](C)O[C@H]2OC[C@@H]3[C@@H](O)[C@H](O)[C@@H](O[C@H]4[C@H](O)[C@H](O)[C@@H](OC(=O)C)[C@H](C)O4)[C@H](O[C@H]4[C@H](O)[C@@H](O)[C@H](C)O[C@@H]4O[C@@H](CCC)CCCCCCCCCC(=O)O[C@H]12)O3)[C@H]([C@@H](O)C)C	2.7240215	4.6082972	0	0	0	0	0	1	0	0	1	1	0	0	0
PZVXILWUBLKOBQ-DBASVXGUSA-N	O=C(O[C@H]1[C@H](O)[C@@H](C)O[C@H]2O[C@H]3[C@H](O)[C@@H](C)O[C@H]([C@@H]3O[C@H]3[C@H](O)[C@H](O)[C@@H](OC(=O)C)[C@H](C)O3)O[C@@H]3[C@@H](O)[C@H](O)[C@@H](C)O[C@H]3O[C@@H](CCC)CCCCCCCCCC(=O)O[C@H]12)[C@H]([C@@H](O)C)C	2.3923184	4.5835961	0	0	0	0	0	1	0	0	1	1	0	0	0
QWUHUBDKQQPMQG-UHFFFAOYSA-N	O(C)c1c(O)c(O)c2C(=O)C=C(c3cc(O)c(O)cc3)Oc2c1	5.9069723	3.2792184	0	0	0	1	0	1	0	0	0	1	0	0	0
JKVQMJIPYGVPFF-UHFFFAOYSA-N	O=C(C)c1c(O)c2c(OC(CO)(C)C=C2)cc1	3.1714091	2.655101	0	0	0	0	0	0	1	1	0	1	0	0	0
QVYSZKIZAPTGSX-UHFFFAOYSA-N	O(C)c1c(O)c2C(=O)C(O)=C(c3cc(OC)c(OC)cc3)Oc2cc1OC	6.0522154	3.2549143	0	0	0	1	0	1	0	0	0	1	0	0	0
PFTAWBLQPZVEMU-UKRRQHHQSA-N	O[C@H]1[C@@H](c2cc(O)c(O)cc2)Oc2c(c(O)cc(O)c2)C1	5.7851629	2.9499514	0	0	0	0	0	1	0	0	0	1	0	0	0

InChIKey	SMILES	maxHBint3	SpMax8_Bhs	Class (predicted)												
				Split1 (RBF 2-24-2)	Split1 (RBF 2-22-2)	Split2 (RBF 2-27-2)	Split3 (MLP 2-4-2)	Split4 (RBF 2-24-2)	Split5 (MLP 2-4-2)	Split5 (RBF 2-22-2)	Split6 (MLP 2-5-2)	Split7 (MLP 2-4-2)	Split8 (RBF 2-27-2)	Split9 (RBF 2-22-2)	Split9 (RBF 2-28-2)	Split10 (RBF 2-30-2)
KQJGPGHQDDZVHJ-ZFOCBLLKSA-N	O=C1[C@H](O)CC/C=C/[C@H](O)[C@H](O)[C@@H](CCC)O1	5.8073019	2.9142607	0	0	0	0	0	1	0	0	0	1	0	0	0
SOHTUOALNFQEMJ-UHFFFAOYSA-N	O(C)c1c(O)c(OC)cc2c1-c1c(cc(O)cc1)CC2	2.5820919	2.6426718	0	0	0	0	0	0	1	1	0	0	0	0	0
MMBACJOZVSORQJ-KTJIKPCYSA-N	O[C@@H]1[C@@]2(c3ccc(O)cc3)Oc3c(c(O)cc4O[C@]5([C@H](O)[C@H](c6c(O)cc(O)cc6O5)c34)c3ccc(O)cc3)[C@@H]1c1c3O[C@@H]([C@@H](O)Cc3c(O)cc1O2)c1ccc(O)cc1	1.3111565	4.1993738	0	0	0	0	0	1	0	0	0	1	0	0	0
AUIUZJOPXKYLOS-UHFFFAOYSA-N	O(C)c1c(O)c2-c3c(cc(O)cc3)CCc2cc1OC	2.2179969	2.6446861	0	0	0	1	0	0	0	1	0	0	0	0	0
DZXMJUKEEAOGQU-UHFFFAOYSA-N	O(C)c1c(O)ccc(CCc2cc(OC)cc(OC)c2)c1	2.9114875	2.6541135	0	0	0	0	0	0	1	1	0	0	0	0	0
NWPBSPADEDDKAO-UHFFFAOYSA-N	O(C)c1c(OC)c2-c3c(cc(O)cc3)CCc2cc1O	2.6362482	2.6256186	0	0	0	0	0	0	1	1	0	0	0	0	0
RBDNTVALYDCCCN-WCIBSUBMSA-N	O=C(OCC(O)(CO)c1c(O)cc(C)cc1)/C(=C\C)/C	5.4511939	2.9234712	0	0	0	0	0	1	0	0	0	1	0	0	0
YOHOPTQLMXBXDB-LRSUZXNRSA-N	O=C(OCC(O)(COC(=O)/C(=C\C)/C)c1c(O)cc(C)cc1)/C(=C\C)/C	1.3323723	3.1536654	0	0	1	0	0	1	0	0	0	0	0	0	0
CILMBWBPHLLNEH-UHFFFAOYSA-N	O(C)c1c(OC)cc(O)c2C(=O)C(O)=C(c3ccccc3)Oc12	6.2943974	2.9146767	0	0	0	0	0	1	0	0	0	1	0	0	0
KIOAAZWXERJXQO-UHFFFAOYSA-N	O=C(CO)c1c(O)c2c(OC(C)(C)C=C2)cc1	7.4953184	2.5995565	0	0	0	0	0	0	0	1	0	1	0	0	0
IYRMWMYZSQPJKC-UHFFFAOYSA-N	O=C1C(O)=C(c2ccc(O)cc2)Oc2c1c(O)cc(O)c2	6.469443	2.9038596	0	0	0	0	0	1	0	0	0	1	0	0	0
VUNWBEIDIFFPMG-UHFFFAOYSA-N	O(C)c1c(O)c(OC)cc2c1c1c(cc(O)cc1)cc2	2.6302363	2.6065812	0	0	0	0	0	0	1	1	0	0	0	0	0
BMSPEISBKGSBTR-UHFFFAOYSA-N	O(C)c1c(O)ccc(CCc2cc(OC)cc(O)c2)c1	2.9255254	2.6561865	0	0	0	0	0	0	1	1	0	0	0	0	0
KVGGUNZKZGOZHC-ZDUSSCGKSA-N	O(C)c1c(C)c2[C@](O)(C)C(=C)OC(=O)c2c(O)c1	1.7825324	2.7594365	0	0	0	1	0	0	0	1	0	0	0	0	0
RFLUVNUXLRYNNJ-LBPRGKRZSA-N	O=C1OC(=C)[C@@](O)(C)c2c(C)c(O)cc(O)c12	1.8527066	2.7574203	0	0	0	1	0	0	0	1	0	0	0	0	0
SIXFVXJMCGPTRB-UJPOAAIJSA-N	O(C)c1ccc(O[C@H]2[C@H](O)[C@@H](O)[C@H](O)[C@@H](CO)O2)cc1	4.7571189	2.9142567	0	0	0	0	0	1	1	0	0	0	0	0	0

InChIKey	SMILES	maxHBint3	SpMax8_Bhs	Class (predicted)												
				Split1 (RBF 2-24-2)	Split1 (RBF 2-22-2)	Split2 (RBF 2-27-2)	Split3 (MLP 2-4-2)	Split4 (RBF 2-24-2)	Split5 (MLP 2-4-2)	Split5 (RBF 2-22-2)	Split6 (MLP 2-5-2)	Split7 (MLP 2-4-2)	Split8 (RBF 2-27-2)	Split9 (RBF 2-22-2)	Split9 (RBF 2-28-2)	Split10 (RBF 2-30-2)
PFTAWBLQPZVEMU-ZFWWWQNUSA-N	O[C@@H]1[C@H](c2cc(O)c(O)cc2)Oc2c(c(O)cc(O)c2)C1	5.7851629	2.9499514	0	0	0	0	0	1	0	0	0	1	0	0	0
KGGCKPCQFGNZTC-UHFFFAOYSA-N	O(C)C=1C(=O)C(C)=C(O)C(=O)C=1	7.2482301	1.7634778	0	0	0	0	0	0	0	1	0	1	0	0	0
OXXPMFLZLUGGPV-UHFFFAOYSA-N	O(C)C=1C(=O)C(C)=C(O)C(=O)C=1O	7.480873	2.0319833	0	0	0	0	0	0	0	1	0	1	0	0	0
ZGITUQPDWVLERE-UHFFFAOYSA-N	O=C1C(C)=C(O)C(=O)C=C1NC	7.1228378	1.8277683	0	0	0	0	0	0	0	1	0	1	0	0	0
LDRJANCOJOKOPS-UHFFFAOYSA-N	O(C)c1c2C(=O)C(O)=C(c3ccccc3)Oc2cc(O)c1	6.3357102	2.7659948	0	0	0	0	0	1	0	0	0	1	0	0	0
SQFSKOYWJBQGKQ-UHFFFAOYSA-N	O(C)c1ccc(C2=C(O)C(=O)c3c(O)cc(O)cc3O2)cc1	6.3619718	2.8967477	0	0	0	0	0	1	0	0	0	1	0	0	0
ATQPZSQVWCPVGV-UHFFFAOYSA-N	O=C(Oc1cc(O)c(C(=O)O)c(C)c1)c1c(O)cc(OC(=O)c2c(O)cc(O)cc2C)cc1C	0	3.7300995	0	0	0	0	0	1	0	0	0	0	0	0	0
OIPPWFOQEKKFEE-UHFFFAOYSA-N	Oc1cc(O)cc(C)c1	0	0.6503758	0	0	0	0	0	0	0	0	0	1	0	0	0
XANCISIMFMVUPX-UPWRSJHQSA-N	O=C(O[C@@H]1C(C)(C)C2[C@@]3([C@]4(C([C@@]5(C)[C@@](C)([C@@H]([C@@H](CC[C@@H](C(=C)C)C)C)CC5)CC4)CC2)C3)CC1)C	0	3.1462653	0	0	0	0	0	1	0	0	0	0	0	0	0
HCUKNXBLSIDEJS-RUCUJZTOSA-N	O=C1C(C)(C)[C@H]2[C@@]3([C@]4([C@H]([C@@]5(C)[C@@](C)([C@@H]([C@@H](CC[C@@H](C(=C)C)C)C)CC5)CC4)CC2)C3)CC1	0	3.1345417	0	0	0	0	0	1	0	0	0	0	0	0	0
OOBHNVPLRWKOTJ-ZBKSDFMUSA-N	O=C1C(C)(C)[C@H]2[C@@]3([C@]4([C@H]([C@@]5(C)[C@@](C)([C@@H]([C@@H](CC[C@H](C(=C)C)C(C)C)C)CC5)CC4)CC2)C3)CC1	0	3.1792872	0	0	0	0	0	1	0	0	0	0	0	0	0
FEOYVNADIQNWQG-VGOCLHBASA-N	O=CO[C@@H]1C(C)(C)C2[C@@]3([C@]4(C([C@@]5(C)[C@@](C)([C@@H]([C@@H](CC[C@@H](C(=C)C)C)C)CC5)CC4)CC2)C3)CC1	0	3.1425858	0	0	0	0	0	1	0	0	0	0	0	0	0
IXHACUTUTOCSJE-HWTFXIFRSA-N	O[C@@H]1C(C)(C)[C@H]2[C@@]3([C@]4([C@H]([C@@]5(C)[C@@](C)([C@@H]([C@@H](CC[C@@H](C(=C)C)C)C)CC5)CC4)CC2)C3)CC1	0	3.1413289	0	0	0	0	0	1	0	0	0	0	0	0	0
OFMXGFHWLZPCFL-SVRPQWSVSA-N	O=C1[C@H](C)[C@]2(C)[C@H]([C@@]3(C)[C@@H]([C@]4(C)[C@](C)([C@H]5[C@@](C)(CC4)CCC(C)(C)C5)CC3)CC2)CC1	0	3.097709	0	0	0	0	0	1	0	0	0	0	0	0	0

InChIKey	SMILES	maxHBint3	SpMax8_Bhs	Class (predicted)												
				**Split1 (RBF 2-24-2)**	**Split1 (RBF 2-22-2)**	**Split2 (RBF 2-27-2)**	**Split3 (MLP 2-4-2)**	**Split4 (RBF 2-24-2)**	**Split5 (MLP 2-4-2)**	**Split5 (RBF 2-22-2)**	**Split6 (MLP 2-5-2)**	**Split7 (MLP 2-4-2)**	**Split8 (RBF 2-27-2)**	**Split9 (RBF 2-22-2)**	**Split9 (RBF 2-28-2)**	**Split10 (RBF 2-30-2)**
YPWQSKQSNNTXOL-TWWFCBCGSA-N	O=C1[C@H](C)[C@]2(C)[C@H]([C@@]3(C)[C@@H]([C@]4(C)[C@](C)([C@H]5[C@@](CO)(CC4)CCC(C)(C)C5)CC3)CC2)CC1	0	3.1981133	0	0	0	0	0	1	0	0	0	0	0	0	0
ONRNCDHSZVITNY-TWWFCBCGSA-N	O=C[C@]12[C@H]([C@@]3(C)[C@@](C)([C@@H]4[C@](C)([C@H]5[C@@](C)([C@@H](C)C(=O)CC5)CC4)CC3)CC1)CC(C)(C)CC2	0	3.1781464	0	0	0	0	0	1	0	0	0	0	0	0	0
YLXXSWJDIFLXSG-MRXNPFEDSA-N	O=C(O[C@H]1C(C)(C)Oc2c(c(OC)c3C(=O)C=C(C)Oc3c2)C1)C(C)C	0	3.0345688	0	0	0	0	0	1	0	0	0	0	0	0	0
BUTUNJHEBGRWGK-QZTJIDSGSA-N	O=C(O[C@H]1[C@@H](OC(=O)C)C(C)(C)Oc2c1c1OC(=O)C=Cc1cc2)C(C)C	0	3.1760837	0	0	0	0	0	1	0	0	0	0	0	0	0
IGMRYMQUDCGCFD-QZTJIDSGSA-N	O=C(O[C@H]1[C@@H](OC(=O)C)C(C)(C)Oc2c1c1OC(=O)C=Cc1cc2)CC	0	3.1083779	0	0	0	0	0	1	0	0	0	0	0	0	0
XYYJJFWJWWXIID-DWSJCSSQSA-N	O=C(OC/C/1=C/C=C\C(C)CC[C@H]2C(=C)C(=O)O[C@@H]2C\1)CC(C)C	0	3.0457095	0	0	0	0	0	1	0	0	0	0	0	0	0
JMXMIHVPTCKCDT-NTDRGAGCSA-N	O=C(/C=C/c1ccc(O)cc1)[C@@H]1C(C)(C)C2[C@@]3([C@]4(C([C@@]5(C)[C@@](C)([C@@H]([C@@H](CCC(C(=C)C)(C)C)C)CC5)CC4)CC2)C3)CC1	0	3.3431388	0	0	0	0	0	1	0	0	0	0	0	0	0
QGFZRJUQPWGMGR-HLYDRURXSA-N	O[C@@H]1C(C)(C)C2[C@@](C)(C=3C([C@@]4(C)[C@@](C)([C@@H]([C@@H](CCC(C(=C)C)(C)C)C)CC4)CC=3)CC2)CC1	0	3.1531897	0	0	0	0	0	1	0	0	0	0	0	0	0
BCONSRYNVDIUFV-HEMYWYTFSA-N	O[C@@H]1C(C)(C)C2[C@@](C)(C=3C([C@@]4(C)[C@@](C)([C@@H]([C@@H](CC[C@@H](C(=C)C)C)C)CC4)CC=3)CC2)CC1	0	3.1368665	0	0	0	0	0	1	0	0	0	0	0	0	0
YCGKSBDRFMKJGQ-FFHDHRDUSA-N	[C@H](CCC(C(=C)C)(C)C)(C)[C@@H]1[C@]2(C)[C@](C)(C3[C@](C)([C@]45O[C@H](C(C)(C)C4CC3)CC5)CC2)CC1	0	3.1508598	0	0	0	0	0	1	0	0	0	0	0	0	0
MPPQCCFNXBGKFC-RZTYQLBFSA-N	O(C)c1c(OC)ccc([C@H]2OC[C@@H]3[C@@H](c4c(C)c5OCOc5cc4)OC[C@H]23)c1	0	3.097153	0	0	0	0	0	1	0	0	0	0	0	0	0
FYIHJFOIIUOEKY-LBTBCDHLSA-N	O(C)c1c([C@H]2OC[C@@H]3[C@@H](c4c(OC)c5OCOc5cc4)OC[C@H]23)ccc2OCOc12	0	3.1956695	0	0	0	0	0	1	0	0	0	0	0	0	0
IZFXFVSOQMEZKB-MNFMDYEBSA-N	O=C1O[C@H](CCC)[C@@H](O)[C@@H](O)/C=C/CC1	4.4634477	2.496977	0	0	1	0	0	0	0	0	0	0	0	0	0
AFZOXJCUHIZZDU-UHFFFAOYSA-N	O=C1OC(CCCCCCCCCCCCCCCCCCCC)CC1	0	3.1069877	0	0	0	0	0	1	0	0	0	0	0	0	0

InChIKey	SMILES	maxHBint3	SpMax8_Bhs	Class (predicted)												
				Split1 (RBF 2-24-2)	Split1 (RBF 2-22-2)	Split2 (RBF 2-27-2)	Split3 (MLP 2-4-2)	Split4 (RBF 2-24-2)	Split5 (MLP 2-4-2)	Split5 (RBF 2-22-2)	Split6 (MLP 2-5-2)	Split7 (MLP 2-4-2)	Split8 (RBF 2-27-2)	Split9 (RBF 2-22-2)	Split9 (RBF 2-28-2)	Split10 (RBF 2-30-2)
FLVQHDPEFVLJND-UHFFFAOYSA-N	O=C1OC(CCCCCCCCCCCCCCCCCCCCC)CC1	0	3.1487607	0	0	0	0	0	1	0	0	0	0	0	0	0
KWOWNYUWWHKUCK-UHFFFAOYSA-N	O=C1OC(CCCCCCCCCCCCCCCCCCCCCC)CC1	0	3.1862167	0	0	0	0	0	1	0	0	0	0	0	0	0
RATVVPKFLMJAST-UHFFFAOYSA-N	O=C1OC(CCCCCCCCCCCCCCCCCCCCCCC)CC1	0	3.2198839	0	0	0	0	0	1	0	0	0	0	0	0	0
JBDGMANPHCRIOU-UHFFFAOYSA-N	O=C1OC(CCCCCCCCCCCCCCCCCCCCCCCC)CC1	0	3.2502262	0	0	0	0	0	1	0	0	0	0	0	0	0
BYWVQXXACNFXHT-UHFFFAOYSA-N	O=C1OC(CCCCCCCCCCCCCCCCCCCCCCCCC)CC1	0	3.2776466	0	0	0	0	0	1	0	0	0	0	0	0	0
YLLQOTIGQVCOEH-UHFFFAOYSA-N	O=C1OC(CCCCCCCCCCCCCCCCCCCCCCCCCC)CC1	0	3.3024942	0	0	0	0	0	1	0	0	0	0	0	0	0
VKISCDZKKVVZQH-BMRADRMJSA-N	O=C(CCCCCCCCCCCCCC/C=C/c1cc2OCOc2cc1)C	0	3.051538	0	0	0	0	0	1	0	0	0	0	0	0	0
SVTYLQARFHCNGC-UHFFFAOYSA-N	O=C(CCCCCCCCCCCCCCCCc1cc2OCOc2cc1)C	0	3.0576305	0	0	0	0	0	1	0	0	0	0	0	0	0
PCDQKEPIJYVOPG-NHBPQBDCSA-N	O=C(OC)[C@]12[C@@]3(O)[C@@](C)(CC[C@]1(C)[C@H]1[C@H](O)C[C@]4([C@H](O)C)[C@H]([C@]1(C)CC2)C=CC(=O)OC4)CCC(=C)C3	0	3.5617642	0	0	0	0	0	1	0	0	0	0	0	0	0
KBMOWKVFQHQUFB-NHBPQBDCSA-N	O=C(OC)[C@]12[C@@]3(O)[C@@](C)(CC[C@]1(C)[C@H]1[C@H](O)C[C@]4([C@H](O)C)[C@H]([C@]1(C)CC2)C=CC(=O)OC4)CCC(C)=C3	0	3.5353096	0	0	0	0	0	1	0	0	0	0	0	0	0
IBYSPSUANRCDTF-PJFLNFRUSA-N	O=C(OC)[C@]12[C@@]3(O)[C@@](C)(CC[C@]1(C)[C@H]1[C@H](O)[C@H](O)[C@]4([C@H](O)C)[C@H]([C@]1(C)CC2)C=CC(=O)OC4)CCC(=C)C3	0	3.7339233	0	0	0	0	0	1	0	0	0	0	0	0	0
MYRCCYOWAVWIKR-NHBPQBDCSA-N	O=C(OC)[C@]12[C@@]3(O)[C@](C)(CC=C(C)C3)CC[C@]1(C)[C@H]1[C@H](O)C[C@]3([C@H](O)C)[C@H]([C@]1(C)CC2)C=CC(=O)OC3	0	3.545654	0	0	0	0	0	1	0	0	0	0	0	0	0
HRMXSRHLUOTLBH-PJFLNFRUSA-N	O=C(OC)[C@]12[C@@]3(O)[C@](C)(CC=C(C)C3)CC[C@]1(C)[C@H]1[C@H](O)[C@H](O)[C@]3([C@H](O)C)[C@H]([C@]1(C)CC2)C=CC(=O)OC3	0	3.7081114	0	0	0	0	0	1	0	0	0	0	0	0	0
PLOHJRTXRRLBGH-STODXTIPSA-N	O=C(O[C@@H]1[C@H](O)[C@]2([C@H](O)C)[C@H]([C@@]3(C)[C@H]1[C@]1(C)[C@](C(=O)OC)([C	0	3.7478392	0	0	0	0	0	1	0	0	0	0	0	0	0

InChIKey	SMILES	maxHBint3	SpMax8_Bhs	Class (predicted)												
				Split1 (RBF 2-24-2)	Split1 (RBF 2-22-2)	Split2 (RBF 2-27-2)	Split3 (MLP 2-4-2)	Split4 (RBF 2-24-2)	Split5 (MLP 2-4-2)	Split5 (RBF 2-22-2)	Split6 (MLP 2-5-2)	Split7 (MLP 2-4-2)	Split8 (RBF 2-27-2)	Split9 (RBF 2-22-2)	Split9 (RBF 2-28-2)	Split10 (RBF 2-30-2)
	@@]4(O)[C@@](C)(CC1)CCC(=C)C4)CC3)C=CC(=O)OC2)C															
BQZKAEWQBJHAIN-STODXTIPSA-N	O=C(O[C@@H]1[C@H](O)[C@]2([C@H](O)C)[C@H]([C@@]3(C)[C@H]1[C@]1(C)[C@](C(=O)OC)([C@@]4(O)[C@](C)(CC=C(C)C4)CC1)CC3)C=CC(=O)OC2)C	0	3.7053411	0	0	0	0	0	1	0	0	0	0	0	0	0
DFTOCERTSAMQSM-STODXTIPSA-N	O=C(O[C@H]1[C@@H](O)[C@@H]2[C@]3(C)[C@](C(=O)OC)([C@@]4(O)[C@@](C)(CC3)CCC(=C)C4)CC[C@@]2(C)[C@H]2[C@]1([C@H](O)C)COC(=O)C=C2)C	0	3.7427323	0	0	0	0	0	1	0	0	0	0	0	0	0
MAASNULAYUTHCM-STODXTIPSA-N	O=C(O[C@H]1[C@@H](O)[C@@H]2[C@]3(C)[C@](C(=O)OC)([C@@]4(O)[C@](C)(CC=C(C)C4)CC3)CC[C@@]2(C)[C@H]2[C@]1([C@H](O)C)COC(=O)C=C2)C	0	3.7092905	0	0	0	0	0	1	0	0	0	0	0	0	0
GAWIXWVDTYZWAW-UHFFFAOYSA-N	O[C@H](C	0	0.496305	0	0	0	0	0	0	0	0	0	1	0	0	0
DNVPQKQSNYMLRS-APGDWVJJSA-N	O[C@@H]1CC=2[C@@](C)([C@@H]3C([C@H]4[C@@](C)([C@@H]([C@@H](/C=C/[C@@H](C(C)C)C)C)CC4)CC3)=CC=2)CC1	0	3.0388315	0	0	0	0	0	1	0	0	0	0	0	0	0
SLFMBUBDOYEWDU-XIDJHNHKSA-N	O=C1C(C)(C)C2[C@@]3([C@]4(C([C@@]5(C)[C@@](C)([C@@H]([C@@H](CCC(C(=C)C)(C)C)C)CC5)CC4)CC2)C3)CC1	0	3.1667886	0	0	0	0	0	1	0	0	0	0	0	0	0
WWZTVIKABWJXIY-IRXLFIPWSA-N	O[C@@H]1C(C)(C)C2[C@@]3([C@]4(C([C@@]5(C)[C@@](C)([C@@H]([C@@H](CCC(C(=C)C)(C)C)C)CC5)CC4)CC2)C3)CC1	0	3.1762691	0	0	0	0	0	1	0	0	0	0	0	0	0
PENVZGWIOSIDJF-UHFFFAOYSA-N	O(C)c1c2C(=O)CC(c3cc(-c4c(O)c5C(=O)C=C(c6ccc(O)cc6)Oc5cc4O)c(O)cc3)Oc2cc(O)c1	0	3.7301955	0	0	0	0	0	1	0	0	0	0	0	0	0
SFEUTIOWNUGQMZ-ZHLOSDGBSA-N	O=C(O[C@@H]1C(C)(C)[C@H]2[C@@](C)([C@@H]3[C@](C)([C@@]4(C)[C@@H]([C@H]5[C@H](C)C(=C)CC[C@]5(C)CC4)CC3)CC2)CC1)C	0	3.1617268	0	0	0	0	0	1	0	0	0	0	0	0	0
NZCULBURCGAPSF-KTWGDNJDSA-N	O=C(O)[C@@]12[C@@H]([C@@H](C)[C@H](C)CC1)C=1[C@](C)([C@@]3(C)[C@@H]([C@]4(C)[C@H]([C@](CO)(C)[C@H](O)CC4)CC3)CC=1)CC2	0	3.3057255	0	0	0	0	0	1	0	0	0	0	0	0	0

InChIKey	SMILES	maxHBint3	SpMax8_Bhs	Class (predicted)												
				Split1 (RBF 2-24-2)	Split1 (RBF 2-22-2)	Split2 (RBF 2-27-2)	Split3 (MLP 2-4-2)	Split4 (RBF 2-24-2)	Split5 (MLP 2-4-2)	Split5 (RBF 2-22-2)	Split6 (MLP 2-5-2)	Split7 (MLP 2-4-2)	Split8 (RBF 2-27-2)	Split9 (RBF 2-22-2)	Split9 (RBF 2-28-2)	Split10 (RBF 2-30-2)
QAIPRVGONGVQAS-DUXPYHPUSA-N	O=C(O)/C=C/c1cc(O)c(O)cc1	6.1188421	2.0517014	0	0	0	0	0	0	0	0	0	1	0	0	0
LNTHITQWFMADLM-UHFFFAOYSA-N	O=C(O)c1cc(O)c(O)c(O)c1	6.2557938	2.0201058	0	0	0	0	0	0	0	0	0	1	0	0	0
FBSFWRHWHYMIOG-UHFFFAOYSA-N	O=C(OC)c1cc(O)c(O)c(O)c1	6.2217643	2.0523634	0	0	0	0	0	0	0	0	0	1	0	0	0
UWHUTZOCTZJUKC-JKSUJKDBSA-N	Oc1c(O)cc2c([C@H]3[C@](O)(COc4c3ccc(O)c4)C2)c1	5.2765423	2.7733625	0	0	0	0	0	1	0	0	0	0	0	0	0
QCDYQQDYXPDABM-UHFFFAOYSA-N	Oc1cc(O)cc(O)c1	0	0.6590587	0	0	0	0	0	0	0	0	0	1	0	0	0
FXJPPIJUTVUQRL-NIQLMDKRSA-N	O=C(O[C@@H]1[C@H](OC(=O)c2ccccc2)[C@]2(C)[C@@H](OC(=O)C)CC[C@@H](C)[C@]32[C@H](O)[C@@H]1C(C)(C)O3)/C=C/c1ccccc1	0	3.478107	0	0	0	0	0	1	0	0	0	0	0	0	0
KAPQKCUDPVRZEX-ZGIPRAPUSA-N	O=C(O[C@@H]1[C@H](OC(=O)c2ccccc2)[C@]2(C)[C@@H](OC(=O)C)CC[C@@](O)(C)[C@]32[C@H](O)[C@@H]1C(C)(C)O3)/C=C/c1ccccc1	0.0084358	3.7158797	0	0	0	0	0	1	0	0	0	0	0	0	0
OVQQPOHQLNLSCP-HHATXKITSA-N	O=C(O[C@@H]1[C@H](OC(=O)c2ccccc2)[C@]2(C)[C@@H](OC(=O)C)CC[C@@](O)(C)[C@]32[C@H](OC(=O)C)[C@@H]1C(C)(C)O3)/C=C/c1ccccc1	0	3.8236869	0	0	0	0	0	1	0	0	0	0	0	0	0
AGFAYIPDDGSEBN-IBXLWUJLSA-N	O[C@@]12c3c(OC1O[C@]1([C@@H](C)Oc4c1ccc(C)c4)CC2)cc(C)cc3	1.3230164	2.9362312	0	0	0	0	0	0	0	1	0	0	0	0	0
IXNDLYSEFZRJIJ-QIXKMHTASA-N	O=C(O)C[C@@]1(C)CC2[C@@]3(C)[C@@](C)(C4[C@](C)(C5[C@@](C)([C@@H](C)C(=O)CC5)CC4)CC3)CC[C@@]2(C)CC1	0	3.251792	0	0	0	0	0	1	0	0	0	0	0	0	0
OFMXGFHWLZPCFL-QDTNQAACSA-N	O=C1[C@H](C)[C@]2(C)C([C@@]3(C)C([C@]4(C)[C@](C)(C5[C@@](C)(CC4)CCC(C)(C)C5)CC3)CC2)CC1	0	3.097709	0	0	0	0	0	1	0	0	0	0	0	0	0
CAMPYEYUASPOIB-IPQGBBEOSA-N	O=C1[C@H](C)[C@]2(C)C([C@@]3(C)C([C@]4(C)[C@](C)(C5[C@@](C)(CC4)CC[C@](CCO)(C)C5)CC3)CC2)CC1	0	3.2245085	0	0	0	0	0	1	0	0	0	0	0	0	0
YPWQSKQSNNTXOL-JQKPCDNZSA-N	O=C1[C@H](C)[C@]2(C)C([C@@]3(C)C([C@]4(C)[C@](C)(C5[C@@](CO)(CC4)CCC(C)(C)C5)CC3)CC2)CC1	0	3.1981133	0	0	0	0	0	1	0	0	0	0	0	0	0

InChIKey	SMILES	maxHBint3	SpMax8_Bhs	Class (predicted)												
				Split1 (RBF 2-24-2)	Split1 (RBF 2-22-2)	Split2 (RBF 2-27-2)	Split3 (MLP 2-4-2)	Split4 (RBF 2-24-2)	Split5 (MLP 2-4-2)	Split5 (RBF 2-22-2)	Split6 (MLP 2-5-2)	Split7 (MLP 2-4-2)	Split8 (RBF 2-27-2)	Split9 (RBF 2-22-2)	Split9 (RBF 2-28-2)	Split10 (RBF 2-30-2)
XCDQFROEGGNAER-PPGXERKRSA-N	O[C@@H]1[C@H](C)[C@]2(C)C([C@@]3(C)C([C@]4(C)[C@](C)(C5[C@@](C)(CC4)CCC(C)(C)C5)CC3)CC2)CC1	0	3.1214906	0	0	0	0	0	1	0	0	0	0	0	0	0
VOYZLKWKVLYJHD-IDELEHTOSA-N	O=C(O)/C(=C\CC[C@@H](C)[C@H]1[C@@]2(C)[C@@](C)(C=3[C@@H]([C@]4(C)C(C(C)(C)C(=O)CC4)CC=3)CC2)CC1)/C	0	3.2791495	0	0	0	0	0	1	0	0	0	0	0	0	0
UILQHUKSFUOOLH-XGRQLWIPSA-N	O=C(O)/C(=C\CC[C@@H](C)[C@H]1[C@@]2(C)[C@@](C)(C=3[C@@H]([C@]4(C)C(C(C)(C)[C@H](O)CC4)CC=3)CC2)CC1)/C	0	3.2869414	0	0	0	0	0	1	0	0	0	0	0	0	0
JFSHUTJDVKUMTJ-QHPUVITPSA-N	O[C@@H]1C(C)(C)[C@H]2[C@@](C)([C@@H]3[C@](C)([C@@]4(C)C([C@H]5[C@@](C)(CC4)CCC(C)(C)C5)=CC3)CC2)CC1	0	3.1028222	0	0	0	0	0	1	0	0	0	0	0	0	0
KZJWDPNRJALLNS-VJSFXXLFSA-N	O[C@@H]1CC=2[C@@](C)([C@@H]3[C@H]([C@H]4[C@@](C)([C@@H]([C@@H](CC[C@H](C(C)C)C)C)CC4)CC3)CC=2)CC1	0	3.1355353	0	0	0	0	0	1	0	0	0	0	0	0	0
UBBRXVRQZJSDAK-FGUXGJARSA-N	O=C1O/C(=C/CCC)/C2=C1C1[C@H]([C@H]3C=C4C(=O)O/C(=C\CCC)/[C@@]14CC3)CC2	0	3.1470341	0	0	0	0	0	1	0	0	0	0	0	0	0
VOYZLKWKVLYJHD-DSRSRPTBSA-N	O=C(O)/C(=C/CC[C@H](C)[C@H]1[C@@]2(C)[C@@](C)(C=3C([C@]4(C)C(C(C)(C)C(=O)CC4)CC=3)CC2)CC1)/C	0	3.2791495	0	0	0	0	0	1	0	0	0	0	0	0	0
UILQHUKSFUOOLH-YOOZGKBQSA-N	O=C(O)/C(=C/CC[C@H](C)[C@H]1[C@@]2(C)[C@@](C)(C=3C([C@]4(C)C(C(C)(C)[C@H](O)CC4)CC=3)CC2)CC1)/C	0	3.2869414	0	0	0	0	0	1	0	0	0	0	0	0	0
MIJYXULNPSFWEK-LGSDIRQTSA-N	O=C(O)[C@]12C(C=3[C@](C)([C@@]4(C)C([C@]5(C)C(C(C)(C)[C@H](O)CC5)CC4)CC=3)CC1)CC(C)(C)CC2	0	3.2948674	0	0	0	0	0	1	0	0	0	0	0	0	0
YXHVCZZLWZYHSA-FPLPWBNLSA-N	O=C(O)c1c(O)cccc1CCCCCCC/C=C\CCCCCC	0	3.1768565	0	0	0	0	0	1	0	0	0	0	0	0	0
ADFWQBGTDJIESE-UHFFFAOYSA-N	O=C(O)c1c(O)cccc1CCCCCCCCCCCCCCC	0	3.2086029	0	0	0	0	0	1	0	0	0	0	0	0	0
OSXBYONEOVGKOX-ARJAWSKDSA-N	O=C(O)c1c(O)cccc1CCCCCCCCCCCCCCC/C=C\CC	0	3.3206673	0	0	0	0	0	1	0	0	0	0	0	0	0
KLYPIICREBFTGY-UHFFFAOYSA-N	O=C(O)c1c(O)cccc1CCCCCCCCCCCCCCCCCCC	0	3.3406834	0	0	0	0	0	1	0	0	0	0	0	0	0

InChIKey	SMILES	maxHBint3	SpMax8_Bhs	Class (predicted)												
				Split1 (RBF 2-24-2)	Split1 (RBF 2-22-2)	Split2 (RBF 2-27-2)	Split3 (MLP 2-4-2)	Split4 (RBF 2-24-2)	Split5 (MLP 2-4-2)	Split5 (RBF 2-22-2)	Split6 (MLP 2-5-2)	Split7 (MLP 2-4-2)	Split8 (RBF 2-27-2)	Split9 (RBF 2-22-2)	Split9 (RBF 2-28-2)	Split10 (RBF 2-30-2)
OFAIMNXQEZOWPU-RXKIPKFJSA-N	O=C(O[C@H]1c2c(c(O)cc(C)c2)C(O)=C2C(=O)[C@@H]3c4c(O)c(C(=O)c5c(C(=O)OC)c(O)ccc5O)c(O)cc4C[C@]12C=C3)C	0	4.1543919	0	0	0	0	0	1	0	0	0	0	0	0	0
OXAJFKDURZRJOW-KRWDZBQOSA-N	O=C(OC)[C@@]12C(=O)C(C)=COC(OC)=C1C(=O)c1c(O)cccc1O2	0	3.2214148	0	0	0	0	0	1	0	0	0	0	0	0	0
RLLCMRPOSFZYJJ-UHFFFAOYSA-N	O=C(Oc1c(C)c(O)c(C(=O)O)c(C)c1)c1c(OC)c(C)c(OC(=O)c2c(O)c(C)c(O)cc2C)c(C)c1C	0	3.7485118	0	0	0	0	0	1	0	0	0	0	0	0	0
MGGMNKJGDSNTKZ-UHFFFAOYSA-N	O=C(Oc1c(C)c(O)c(C(=O)O)c(C)c1C)c1c(O)c(C)c(OC(=O)c2c(O)c(C)c(O)cc2C)c(C)c1C	0	3.7698875	0	0	0	0	0	1	0	0	0	0	0	0	0
IGYZEKLJQWCRPT-UHFFFAOYSA-N	O=C(Oc1c(C)c(O)c(C(=O)O)c(C)c1C)c1c(OC)c(C)c(OC(=O)c2c(O)c(C)c(O)cc2C)c(C)c1C	0	3.7556718	0	0	0	0	0	1	0	0	0	0	0	0	0
SZCAUZSZQPVKQY-CCFHIKDMSA-N	O=C(OCCCCCCCCCCCCCCCCCCCCCCCCCC)/C=C/c1ccc(O)cc1	0	3.399452	0	0	0	0	0	1	0	0	0	0	0	0	0
CWHBUKZWMZSTEA-KKYHWDRJSA-N	O=C(OCCCCCCCCCCCCCCCCCCCCCCCCCCCC)/C=C/c1ccc(O)cc1	0	3.4270963	0	0	0	0	0	1	0	0	0	0	0	0	0
DUIIKPOJUKGTSI-LVYIWIAJSA-N	O=C(OCCCCCCCCCCCCCCCCCCCCCCCCCCCCCC)/C=C/c1ccc(O)cc1	0	3.4502997	0	0	0	0	0	1	0	0	0	0	0	0	0
RGHHSNMVTDWUBI-UHFFFAOYSA-N	O=Cc1ccc(O)cc1	0	0.6015263	0	0	0	0	0	0	0	0	0	1	0	0	0
JUVIOZPCNVVQFO-HBGVWJBISA-N	O(C)c1c(OC)cc2[C@@H]3C(=O)c4c(O[C@@H]3COc2c1)c1c(O[C@@H](C(=C)C)C1)cc4	0	3.0896964	0	0	0	0	0	1	0	0	0	0	0	0	0
ZRZWBWPDBOVIGQ-YWZWRZHGSA-N	O=C1N(C)[C@]2(Cc3c4c(n([C@@]56[C@@H](N7C(=O)[C@@]8(CO)N(C)C(=O)[C@@]7(SS8)C5)Nc5c6cccc5)c3)cccc4)C(=O)N(C)[C@@]1(CO)SS2	0.5395745	3.9653888	0	0	0	0	0	1	0	0	0	0	0	0	0
HHGPYJLEJGNWJA-UHFFFAOYSA-N	O(C)c1c(OC)ccc(C2=C(OC)C(=O)c3c(O)cc(OC)cc3O2)c1	0	3.1821762	0	0	0	0	0	1	0	0	0	0	0	0	0
XUAORUWVUTVEEC-UHFFFAOYSA-N	O=C1c2c(O)c(c(O)cc2OC(c2ccc(O)cc2)=C1)-c1c(O)ccc(C2Oc3c(c(O)cc(O)c3)C(=O)C2)c1	0	4.0568852	0	0	0	0	0	1	0	0	0	0	0	0	0
BORWSEZUWHQTOK-UHFFFAOYSA-N	O=C1c2c(O)c(c(O)cc2OC(c2ccc(O)cc2)=C1)-c1c(O)ccc(C=2Oc3c(c(O)cc(O)c3)C(=O)C=2)c1	0	4.0127318	0	0	0	0	0	1	0	0	0	0	0	0	0
YUSWMAULDXZHPY-UHFFFAOYSA-N	O=C1c2c(O)cc(O)c(-c3c(O)ccc(C=4Oc5c(c(O)cc(O)c5)C(=O)C=4)c3)c2OC(c2ccc(O)cc2)=C1	0	4.0095369	0	0	0	0	0	1	0	0	0	0	0	0	0

InChIKey	SMILES	maxHBint3	SpMax8_Bhs	Class (predicted)												
				Split1 (RBF 2-24-2)	Split1 (RBF 2-22-2)	Split2 (RBF 2-27-2)	Split3 (MLP 2-4-2)	Split4 (RBF 2-24-2)	Split5 (MLP 2-4-2)	Split5 (RBF 2-22-2)	Split6 (MLP 2-5-2)	Split7 (MLP 2-4-2)	Split8 (RBF 2-27-2)	Split9 (RBF 2-22-2)	Split9 (RBF 2-28-2)	Split10 (RBF 2-30-2)
UBBRXVRQZJSDAK-ZJHGLIIDSA-N	O=C1O/C(=C\CCC)/C2=C1[C@H]1[C@H]([C@H]3C=C4C(=O)O/C(=C\CCC)/[C@@]14CC3)CC2	0	3.1470341	0	0	0	0	0	1	0	0	0	0	0	0	0
STSOHAOGZMLWFR-SSDLBLMSSA-N	O=C1O[C@@H](C)[C@@H](O)c2c1c(O)ccc2	2.4061424	2.3472772	0	0	0	0	0	0	0	1	0	0	0	0	0
STSOHAOGZMLWFR-CDUCUWFYSA-N	O=C1O[C@@H](C)[C@H](O)c2c1c(O)ccc2	2.4061424	2.3472772	0	0	0	0	0	0	0	1	0	0	0	0	0
GAWIXWVDTYZWAW-UHFFFAOYSA-N	O([C@@H](C	0	0.496305	0	0	0	0	0	0	0	0	0	1	0	0	0
ODHSWYNEXJIJEI-YKJBWMMYSA-N	O=C(OC)C[C@H]1C(C)(C)[C@H](OC(=O)C)[C@@]2(O)C(=O)[C@]1(C)C1C(=C2)C2[C@](C)([C@H](c3cocc3)OC(=O)C2)CC1	0	3.6002806	0	0	0	0	0	1	0	0	0	0	0	0	0
NMEMUNAMFPMDHM-ICQCPBIVSA-N	O=C(OC)C[C@H]1C(C)(C)[C@H](OC(=O)C)[C@@]2(O)C(=O)[C@]1(C)C1[C@@]3(O[C@H]23)C2[C@](C)([C@H](c3cocc3)OC(=O)C2)CC1	0	3.6322327	0	0	0	0	0	1	0	0	0	0	0	0	0
JJTOVDGWVPPWNZ-DTFVITINSA-N	O=C(O[C@H]1C(C)(C)[C@H](CC(=O)OC)[C@]2(C)C(=O)[C@@]1(O)C=C1C3[C@](C)([C@H](c4cocc4)OC(=O)C3)CCC21)/C(=C/C)/C	0	3.5894764	0	0	0	0	0	1	0	0	0	0	0	0	0
MBIKCFMEQQFVRE-BTZXGELBSA-N	O=C(O[C@H]1C(C)(C)[C@H](CC(=O)OC)[C@]2(C)C(=O)[C@@]1(O)C=C1C3[C@](C)([C@H](c4cocc4)OC(=O)C3)CCC21)C(C)C	0	3.5997176	0	0	0	0	0	1	0	0	0	0	0	0	0
LOOFGUAWTZPQEG-BWSSPMROSA-N	O=C(O[C@H]1C(C)(C)[C@H](CC(=O)OC)[C@]2(C)C(=O)[C@@]1(O)[C@H]1O[C@@]31C1[C@](C)([C@H](c4cocc4)OC(=O)C1)CCC23)C(C)C	0	3.6300137	0	0	0	0	0	1	0	0	0	0	0	0	0
UMAZUSDJQFBJAR-JLZHRPIQSA-N	O=C(O[C@H]1C(C)(C)[C@H](CC(=O)OC)[C@]2(C)C(=O)[C@@]1(OC(=O)C)C=C1C3[C@](C)([C@H](c4cocc4)OC(=O)C3)CCC21)/C(=C/C)/C	0	3.6050351	0	0	0	0	0	1	0	0	0	0	0	0	0
IQZMMMBJYWYVKW-MQDCWUPWSA-N	O=C(O[C@H]1C(C)(C)[C@H](CC(=O)OC)[C@]2(C)C(=O)[C@@]1(OC(=O)C)C=C1C3[C@](C)([C@H](c4cocc4)OC(=O)C3)CCC21)C(C)C	0	3.6158788	0	0	0	0	0	1	0	0	0	0	0	0	0
COKNSFVQXLZHTF-YZMSHABZSA-N	O=C(O[C@H]1C(C)(C)[C@H](CC(=O)OC)[C@]2(C)C(=O)[C@@]1(OC(=O)C)[C@H]1O[C@@]31C1[C@](C)([C@H](c4cocc4)OC(=O)C1)CCC23)C(C)C	0	3.6481053	0	0	0	0	0	1	0	0	0	0	0	0	0
RFYQGKUTWAUUJW-UHFFFAOYSA-N	O(C)C=1C(=O)C(C)=C(Nc2ccc(CCCC)cc2)C(=O)C=1	4.7519727	2.7918845	0	0	0	0	0	1	0	0	0	0	0	0	0

InChIKey	SMILES	maxHBint3	SpMax8_Bhs	Class (predicted)												
				Split1 (RBF 2-24-2)	Split1 (RBF 2-22-2)	Split2 (RBF 2-27-2)	Split3 (MLP 2-4-2)	Split4 (RBF 2-24-2)	Split5 (MLP 2-4-2)	Split5 (RBF 2-22-2)	Split6 (MLP 2-5-2)	Split7 (MLP 2-4-2)	Split8 (RBF 2-27-2)	Split9 (RBF 2-22-2)	Split9 (RBF 2-28-2)	Split10 (RBF 2-30-2)
WCGUUGGRBIKTOS-GPOJBZKASA-N	O=C(O)[C@@]12[C@@H]([C@@H](C)[C@H](C)CC1)C=1[C@](C)([C@@]3(C)[C@@H]([C@]4(C)[C@H](C(C)(C)[C@@H](O)CC4)CC3)CC=1)CC2	0	3.2919114	0	0	0	0	0	1	0	0	0	0	0	0	0
XUARCIYIVXVTAE-ZAPOICBTSA-N	OC[C@@]12[C@@H]([C@@H](C)[C@H](C)CC1)C=1[C@](C)([C@@]3(C)[C@@H]([C@]4(C)[C@H](C(C)(C)[C@@H](O)CC4)CC3)CC=1)CC2	0	3.2365048	0	0	0	0	0	1	0	0	0	0	0	0	0
RILOVTGOFDASJT-LURJTMIESA-N	Clc1c(O)c2C(=O)c3c(O)cc(C[C@@H](O)C)cc3C(=O)c2cc1O	0.0438117	3.3276718	0	0	0	0	0	1	0	0	0	0	0	0	0
HJJVLOSOGVDXLM-UHFFFAOYSA-N	O(C)c1c2c3c(c(O)c1)C(=O)C=C(OC)C3=CC1(C(OC)=CC(=O)c3c(O)cc(OC)cc13)C2	0	3.5526958	0	0	0	0	0	1	0	0	0	0	0	0	0
ORUUOHWWRMGNJL-RGXZNCPUSA-N	O=C(O[C@@]12[C@@](C(C=C)(C)C)(N3C(=O)[C@H](Cc4ccccc4)OC(=O)[C@@H]3C1)Nc1c2cccc1)C	1.016528	3.1877748	0	0	0	0	0	1	0	0	0	0	0	0	0
CRPNQSVBEWWHIJ-UHFFFAOYSA-N	O=Cc1c(O)c(O)c(O)cc1	6.2578213	1.3847689	0	0	0	0	0	0	0	1	0	0	0	0	0
NOEGNKMFWQHSLB-UHFFFAOYSA-N	O=Cc1oc(CO)cc1	3.5446209	0.6624662	0	0	0	0	0	0	0	0	0	1	0	0	0
NIMIDMFTGGTHOH-VYRBHSGPSA-N	O(C)c1c2C(=O)[C@H](O)C(c3ccccc3)Oc2cc(O)c1	5.2460052	2.7892344	0	0	0	0	0	1	0	0	0	0	0	0	0
QPOCKDYYXFOBCM-VYRBHSGPSA-N	O(C)c1cc(O)c2C(=O)[C@H](O)C(c3ccccc3)Oc2c1	5.2986625	2.7892344	0	0	0	0	0	1	0	0	0	0	0	0	0
SUYJZKRQHBQNCA-MLCCFXAWSA-N	O=C1[C@H](O)C(c2ccccc2)Oc2c1c(O)cc(O)c2	5.3931157	2.7972269	0	0	0	0	0	1	0	0	0	0	0	0	0
QDLAGTHXVHQKRE-UHFFFAOYSA-N	O(C)c1cc(O)c2C(=O)c3c(C)cc(OC)cc3Oc2c1	0	2.7290598	0	0	0	0	0	0	0	0	0	0	0	0	0
PXFMUVDLJWXOQM-UHFFFAOYSA-N	O=C(OC)c1c(O)c(C=O)c(O)cc1C	0	2.2987815	0	0	0	0	0	0	0	0	0	0	0	0	0
NCCWCZLEACWJIN-UHFFFAOYSA-N	O=C(OC)c1c(O)cc(O)cc1C	0	2.1975236	0	0	0	0	0	0	0	0	0	0	0	0	0
LRMGCIMCOOHPQA-UHFFFAOYSA-N	O=C(Oc1cc(O)cc(C)c1)c1c(O)cc(O)cc1C	0	2.7664165	0	0	0	0	0	0	0	0	0	0	0	0	0
AMLRGXJZYUJGES-UHFFFAOYSA-N	O(C)c1c2c(c(O)c3C(=O)C(C)=C(C)Oc13)c(OC)cc(OC)c2	0	2.9611102	0	0	0	0	0	0	0	0	0	0	0	0	0
UMPIOBMYFRLDPN-UHFFFAOYSA-N	O(C)c1c2c(c(O)c3C(=O)C=C(C)Oc13)c(OC)cc(OC)c2	0	2.9170192	0	0	0	0	0	0	0	0	0	0	0	0	0

InChIKey	SMILES	maxHBint3	SpMax8_Bhs	Class (predicted)												
				Split1 (RBF 2-24-2)	Split1 (RBF 2-22-2)	Split2 (RBF 2-27-2)	Split3 (MLP 2-4-2)	Split4 (RBF 2-24-2)	Split5 (MLP 2-4-2)	Split5 (RBF 2-22-2)	Split6 (MLP 2-5-2)	Split7 (MLP 2-4-2)	Split8 (RBF 2-27-2)	Split9 (RBF 2-22-2)	Split9 (RBF 2-28-2)	Split10 (RBF 2-30-2)
CQIUKKVOEOPUDV-IYSWYEEDSA-N	O=C(O)C=1C(=O)C(C)=C2[C@H](C)[C@@H](C)OC=C2C=1O	0	2.894399	0	0	0	0	0	0	0	0	0	0	0	0	0
LXCFILQKKLGQFO-UHFFFAOYSA-N	O=C(OC)c1ccc(O)cc1	0	1.2456977	0	0	0	0	0	0	0	0	0	0	0	0	0
RHMXXJGYXNZAPX-UHFFFAOYSA-N	O=C1c2c(O)cc(O)cc2C(=O)c2c1c(O)cc(C)c2	0	2.8607482	0	0	0	0	0	0	0	0	0	0	0	0	0
AHRNUZBHBAHMHY-GFCCVEGCSA-N	Oc1c(C)cc([C@@H](CC/C=C(\C)/C)C)cc1	0	2.5172445	0	0	0	0	0	0	0	0	0	0	0	0	0
FLGAFUAMEXILLB-QXENPCNLSA-N	O=C(O)[C@@]1(C)[C@@H]2[C@](C)([C@H]3[C@@]4(C[C@]5(C)[C@@H]([C@H]5C4)C3)CC2)CCC1	0	2.9701534	0	0	0	0	0	0	0	0	0	0	0	0	0
FKWGCEDRLNNZOZ-GFCCVEGCSA-N	Oc1c(C)ccc([C@@H](CC/C=C(\C)/C)C)c1	0	2.5757841	0	0	0	0	0	0	0	0	0	0	0	0	0
BQAQGBHVNVLESL-IAPIXIRKSA-N	Oc1c(C)ccc([C@@H](CCC2C(C)(C)O2)C)c1	0	2.6210888	0	0	0	0	0	0	0	0	0	0	0	0	0
XQXPVVBIMDBYFF-UHFFFAOYSA-N	O=C(O)Cc1ccc(O)cc1	0	1.2703153	0	0	0	0	0	0	0	0	0	0	0	0	0
XGDZEDRBLVIUMX-UHFFFAOYSA-N	O=C(OC)Cc1ccc(O)cc1	0	1.7068218	0	0	0	0	0	0	0	0	0	0	0	0	0
YEKIIDIQOZQXAX-UHFFFAOYSA-N	O=C(OC)c1c2C(=O)c3c(O)cc(C)cc3Oc2ccc1	0	2.6934354	0	0	0	0	0	0	0	0	0	0	0	0	0
YJOWXMGENDGFDH-IAGOWNOFSA-N	O=C(O[C@@H]1[C@H](OC(=O)C)c2c3OC(=O)C=Cc3ccc2OC1(C)C)C	0	2.9176257	0	0	0	0	0	0	0	0	0	0	0	0	0
QNOQNIIIQHXDAV-CGICMKJESA-N	O=C(O[C@H]1C(C)(C)Oc2c(c(OC)c3C(=O)C=C(C)Oc3c2)C1)/C(=C\C)/C	0	3.0218139	0	0	0	0	0	0	0	0	0	0	0	0	0
RRHCDWLSHIIIIT-NVWZYQMFSA-N	O=C(O[C@H]1C(C)(C)Oc2c(c3OC(=O)C=Cc3cc2)C1)/C(=C\C)/C	0	2.8609357	0	0	0	0	0	0	0	0	0	0	0	0	0
PKHNHXIOSSYBJU-CQSZACIVSA-N	O=C(O[C@H]1C(C)(C)Oc2c(c3OC(=O)C=Cc3cc2)C1)C(C)C	0	2.8741512	0	0	0	0	0	0	0	0	0	0	0	0	0
HKXQUNNSKMWIKJ-DGCLKSJQSA-N	O=C1Oc2c3[C@@H](O)[C@@H](O)C(C)(C)Oc3ccc2C=C1	4.1101285	2.6848652	0	0	0	0	0	0	0	0	0	0	0	0	0
ZMXFKFQNJTXZBC-UHFFFAOYSA-N	O=C(OC(C=C)c1cc(OC)c(OC(=O)C(C)C)cc1)C(C)C	0	2.9615367	0	0	0	0	0	0	0	0	0	0	0	0	0
WEZKIKHPDIXTOZ-LSJUHERESA-N	O=C(OC/C/1=C/C=C\C(C)CC[C@H]2C(=C)C(=O)O[C@@H]2C\1)C(C)C	0	3.0199829	0	0	0	0	0	0	0	0	0	0	0	0	0

InChIKey	SMILES	maxHBint3	SpMax8_Bhs	Class (predicted)												
				Split1 (RBF 2-24-2)	Split1 (RBF 2-22-2)	Split2 (RBF 2-27-2)	Split3 (MLP 2-4-2)	Split4 (RBF 2-24-2)	Split5 (MLP 2-4-2)	Split5 (RBF 2-22-2)	Split6 (MLP 2-5-2)	Split7 (MLP 2-4-2)	Split8 (RBF 2-27-2)	Split9 (RBF 2-22-2)	Split9 (RBF 2-28-2)	Split10 (RBF 2-30-2)
AKXLMIWFRPOECM-QKSACFKYSA-N	O=C(O[C@@H]1C(=C)[C@@H]2[C@H]3OC(=O)C(=C)C3CCC(=C)[C@@H]2C1)C(C)C	0	3.0003016	0	0	0	0	0	0	0	0	0	0	0	0	0
PIGAIBAKKDXNOK-PAZXDIGFSA-N	O=C(O[C@@H]1C(=C)[C@@H]2[C@H]3OC(=O)C(=C)C3CCC(=C)[C@@H]2C1)CC(C)C	0	3.0185438	0	0	0	0	0	0	0	0	0	0	0	0	0
ZMQPXMBRSIEPJQ-UHFFFAOYSA-N	O=C(Oc1c(OC)cc(C(OC(=O)CC(C)C)C=C)cc1)C(C)C	0	2.9806108	0	0	0	0	0	0	0	0	0	0	0	0	0
NETSQGRTUNRXEO-FTKZKSHWSA-N	O=C1C(=C)C2[C@H](O1)[C@H]1C(=C)CC[C@H]1C(=C)CC2	0	2.2903279	0	0	0	0	0	0	0	0	0	0	0	0	0
MMTZAJNKISZWFG-LUIAVFIISA-N	O=C1C(=C)[C@H]2[C@H](O1)C/C(/C)=C\C=C/C(C)CC2	0	2.3908978	0	0	0	0	0	0	0	0	0	0	0	0	0
FZYRENRMDAPHEM-UHFFFAOYSA-N	O(C)c1c2c(O)c3C(=O)C(C)=C(C)Oc3cc2cc(OC)c1	0	2.8105031	0	0	0	0	0	0	0	0	0	0	0	0	0
HFPQKJMLIONCGP-UHFFFAOYSA-N	O(C)c1c2c(O)c3C(=O)C=C(C)Oc3cc2cc(OC)c1	0	2.65488	0	0	0	0	0	0	0	0	0	0	0	0	0
AHQXDHIRTWHABS-QMMMGPOBSA-N	O(C)c1c2c(O)c3C(=O)C[C@H](C)Oc3cc2cc(OC)c1	0	2.6882827	0	0	0	0	0	0	0	0	0	0	0	0	0
DALYWWKMTCBVOR-IUCAKERBSA-N	O(C)c1c2c(O)c3C(=O)[C@@H](C)[C@H](C)Oc3cc2cc(OC)c1	0	2.8849833	0	0	0	0	0	0	0	0	0	0	0	0	0
DALYWWKMTCBVOR-BDAKNGLRSA-N	O(C)c1c2c(O)c3C(=O)[C@H](C)[C@H](C)Oc3cc2cc(OC)c1	0	2.8849833	0	0	0	0	0	0	0	0	0	0	0	0	0
DWGSEYZQYRDTJV-QMMMGPOBSA-N	O(C)c1c2c(c(O)c3C(=O)C[C@H](C)Oc13)c(OC)cc(OC)c2	0	2.936477	0	0	0	0	0	0	0	0	0	0	0	0	0
KRQMXQZQIPIILU-IUCAKERBSA-N	O(C)c1c2c(c(O)c3C(=O)[C@@H](C)[C@H](C)Oc13)c(OC)cc(OC)c2	0	2.9877291	0	0	0	0	0	0	0	0	0	0	0	0	0
KRQMXQZQIPIILU-BDAKNGLRSA-N	O(C)c1c2c(c(O)c3C(=O)[C@H](C)[C@H](C)Oc13)c(OC)cc(OC)c2	0	2.9877291	0	0	0	0	0	0	0	0	0	0	0	0	0
CQIUKKVOEOPUDV-FSPLSTOPSA-N	O=C(O)C=1C(=O)C(C)=C2[C@@H](C)[C@H](C)OC=C2C=1O	0	2.894399	0	0	0	0	0	0	0	0	0	0	0	0	0
OIMXTYUHMBQQJM-GDJCSSNXSA-N	O=C1C=C2[C@@](C)(C3C(=C4[C@@](C)([C@@H]([C@@H](/C=C/[C@@H](C(C)C)C)C)CC4)CC3)C=C2)CC1	0	3.013985	0	0	0	0	0	0	0	0	0	0	0	0	0
AWOGQCSIVCQXBT-VUEDXXQZSA-N	O(C)c1c(OC)ccc([C@H]2OC[C@@H]3[C@@H](c4cc5OCOc5cc4)OC[C@H]23)c1	0	2.9143842	0	0	0	0	0	0	0	0	0	0	0	0	0

InChIKey	SMILES	maxHBint3	SpMax8_Bhs	Class (predicted)												
				Split1 (RBF 2-24-2)	Split1 (RBF 2-22-2)	Split2 (RBF 2-27-2)	Split3 (MLP 2-4-2)	Split4 (RBF 2-24-2)	Split5 (MLP 2-4-2)	Split5 (RBF 2-22-2)	Split6 (MLP 2-5-2)	Split7 (MLP 2-4-2)	Split8 (RBF 2-27-2)	Split9 (RBF 2-22-2)	Split9 (RBF 2-28-2)	Split10 (RBF 2-30-2)
IWZXZFNLDPFUKQ-NAJRYUOPSA-N	O=C1O[C@H](CCC)C[C@@H](O)/C=C/CCC1	0	2.4760568	0	0	0	0	0	0	0	0	0	0	0	0	0
CPDBOUQRDMUUIN-UHFFFAOYSA-N	O=C(O)C(=C)C1CC(=O)C=2C(C)(C(C)CCC=2)C1	0	2.8316484	0	0	0	0	0	0	0	0	0	0	0	0	0
PEYLVIRXSYMEHY-FHXLMMPLSA-N	OC[C@]1(C)[C@H]2C1[C@@H]1[C@H](C)CC[C@@H]1[C@](O)(C)CC2	0	2.7268944	0	0	0	0	0	0	0	0	0	0	0	0	0
GLEWZPUZTYZURE-WYMLVPIESA-N	O=C(/C=C/CCCCCCCCCCCc1cc2OCOc2cc1)C	0	2.976983	0	0	0	0	0	0	0	0	0	0	0	0	0
MRIJVCUMMLLFRJ-GHRIWEEISA-N	O=C(/C=C/CCCCCCCCCCCc1ccccc1)C	0	2.8463665	0	0	0	0	0	0	0	0	0	0	0	0	0
HYOOYGBSEUETML-ZRDIBKRKSA-N	O=C(/C=C/CCCCCCCCCc1cc2OCOc2cc1)C	0	2.9117895	0	0	0	0	0	0	0	0	0	0	0	0	0
XSCQBXUNBGFDEK-ACCUITESSA-N	O=C(CCCCCCCCCC/C=C/c1cc2OCOc2cc1)C	0	2.90863	0	0	0	0	0	0	0	0	0	0	0	0	0
RSLLRVSPUBUTHT-FYWRMAATSA-N	O=C(CCCCCCCCCCCC/C=C/c1cc2OCOc2cc1)C	0	2.9658018	0	0	0	0	0	0	0	0	0	0	0	0	0
OHKYJRJHRQFHDW-UHFFFAOYSA-N	O=C(CCCCCCCCCCCCCCc1cc2OCOc2cc1)C	0	3.0004931	0	0	0	0	0	0	0	0	0	0	0	0	0
NWTRBPHNLLSBBD-UHFFFAOYSA-N	O=C(CCCCCCCCCCCCCCc1ccccc1)C	0	2.8761563	0	0	0	0	0	0	0	0	0	0	0	0	0
WESJGASUMOLVBD-UHFFFAOYSA-N	O=C(CCCCCCCCCCCCc1cc2OCOc2cc1)C	0	2.9414954	0	0	0	0	0	0	0	0	0	0	0	0	0
XKYCRRQKLLZDJB-UHFFFAOYSA-N	O=C(CCCCCCCCCCc1cc2OCOc2cc1)C	0	2.8684918	0	0	0	0	0	0	0	0	0	0	0	0	0
KFHRRMKPUDPLGO-UHFFFAOYSA-N	O=C(OC)CC1OC(CCCCCCCCCCCCC)CCC1	0	3.0215676	0	0	0	0	0	0	0	0	0	0	0	0	0
YGQMDFMXPBTIRS-UHFFFAOYSA-N	O=C(OC)CC1OC(CCCCCCCCCCCCCC)CC1	0	3.0019138	0	0	0	0	0	0	0	0	0	0	0	0	0
UYTVHKZGZWOESS-UHFFFAOYSA-N	O=C(OC)CC1OC(CCCCCCCCCCc2ccccc2)CCC1	0	2.9846725	0	0	0	0	0	0	0	0	0	0	0	0	0
SBMILRCXQPFDME-UHFFFAOYSA-N	O(C)C=1C(=O)C2=C(C(=O)C=1)c1c(cc(O)cc1)CC2	0	2.7485736	0	0	0	0	0	0	0	0	0	0	0	0	0
HMYHQSWJLABPMD-UHFFFAOYSA-N	O(C)C=1C2(C(=O)c3c(O)cccc3CC2)CC(=O)C=1	0	2.5519175	0	0	0	0	0	0	0	0	0	0	0	0	0

InChIKey	SMILES	maxHBint3	SpMax8_Bhs	Class (predicted)												
				Split1 (RBF 2-24-2)	Split1 (RBF 2-22-2)	Split2 (RBF 2-27-2)	Split3 (MLP 2-4-2)	Split4 (RBF 2-24-2)	Split5 (MLP 2-4-2)	Split5 (RBF 2-22-2)	Split6 (MLP 2-5-2)	Split7 (MLP 2-4-2)	Split8 (RBF 2-27-2)	Split9 (RBF 2-22-2)	Split9 (RBF 2-28-2)	Split10 (RBF 2-30-2)
RDKDIPDDUFMMMT-UHFFFAOYSA-N	O(C)c1cc(O)c2-c3c(cc(O)cc3)CCc2c1	0	2.5789757	0	0	0	0	0	0	0	0	0	0	0	0	0
HCRRYKYLZWKBBM-UHFFFAOYSA-N	O(C)c1c(OC)ccc(CCc2cc(OC)cc(OC)c2)c1	0	2.6561562	0	0	0	0	0	0	0	0	0	0	0	0	0
FDJURJXPMJANDW-UHFFFAOYSA-N	O(C)c1cc(O)cc(CCc2cc(OC)ccc2)c1	0	2.5878022	0	0	0	0	0	0	0	0	0	0	0	0	0
NSBYGUHECONSDC-UHFFFAOYSA-N	O(C)c1cc(O)cc(CCc2ccc(O)cc2)c1	0	2.5537467	0	0	0	0	0	0	0	0	0	0	0	0	0
KZNIFHPLKGYRTM-UHFFFAOYSA-N	O=C1c2c(O)cc(O)cc2OC(c2ccc(O)cc2)=C1	0	2.7720865	0	0	0	0	0	0	0	0	0	0	0	0	0
UMZJVKFVOMTAFO-UHFFFAOYSA-N	Oc1cc(O)cc(CCc2cc(O)ccc2)c1	0	2.4632931	0	0	0	0	0	0	0	0	0	0	0	0	0
HITJFUSPLYBJPE-UHFFFAOYSA-N	Oc1cc(O)cc(CCc2ccc(O)cc2)c1	0	2.5074361	0	0	0	0	0	0	0	0	0	0	0	0	0
LHLJANTYAXQUPZ-UHFFFAOYSA-N	O=C(OCC(=O)c1c(O)cc(C)cc1)C	0	2.493969	0	0	0	0	0	0	0	0	0	0	0	0	0
YCOFRPYSZKIPBQ-UHFFFAOYSA-N	O(C)C=1C(O)(C(=C)C)OC(=O)C=1	2.6205964	1.6916467	0	0	0	0	0	0	0	0	0	0	0	0	0
YFRGOWXYOVAUBU-NZFNHWASSA-N	O=C1C(C)=C2C(O1)=CC1=C(O)C(=O)C[C@H](C)[C@@]1(C)C2	5.2784969	2.6689821	0	0	0	0	0	0	0	0	0	0	0	0	0
YLHAQKVIXLQIFW-UHFFFAOYSA-N	O=C(OCc1c(OC)cccc1)c1c(O)cccc1	0	2.6413076	0	0	0	0	0	0	0	0	0	0	0	0	0
PIWSYQREANNHKN-UHFFFAOYSA-N	O=C(OCc1cc(O)ccc1)c1c(OC)cccc1OC	0	2.6078859	0	0	0	0	0	0	0	0	0	0	0	0	0
DGYWXQIMCBTTQH-UHFFFAOYSA-N	O=C(OCc1cc(OC)ccc1)c1c(OC)cccc1O	0	2.6310487	0	0	0	0	0	0	0	0	0	0	0	0	0
DZDQHOAIKWCQDB-UHFFFAOYSA-N	O=C(OCc1cc(OC)ccc1)c1c(OC)cccc1OC	0	2.6278851	0	0	0	0	0	0	0	0	0	0	0	0	0
HKRGBDMRFLWKMB-UHFFFAOYSA-N	O=C(OCc1ccccc1)c1c(OC)c(OC)ccc1O	0	2.7603516	0	0	0	0	0	0	0	0	0	0	0	0	0
WKTUEASUPBAIPX-UHFFFAOYSA-N	O=C(OCc1ccccc1)c1c(OC)c(OC)ccc1OC	0	2.7554258	0	0	0	0	0	0	0	0	0	0	0	0	0
CGNJMCLXIMWKDY-UHFFFAOYSA-N	O=C(OCc1ccccc1)c1c(OC)cccc1O	0	2.5689268	0	0	0	0	0	0	0	0	0	0	0	0	0

InChIKey	SMILES	maxHBint3	SpMax8_Bhs	Class (predicted)												
				Split1 (RBF 2-24-2)	Split1 (RBF 2-22-2)	Split2 (RBF 2-27-2)	Split3 (MLP 2-4-2)	Split4 (RBF 2-24-2)	Split5 (MLP 2-4-2)	Split5 (RBF 2-22-2)	Split6 (MLP 2-5-2)	Split7 (MLP 2-4-2)	Split8 (RBF 2-27-2)	Split9 (RBF 2-22-2)	Split9 (RBF 2-28-2)	Split10 (RBF 2-30-2)
PKIYLOACOOWBCU-UHFFFAOYSA-N	O=C(OCc1ccccc1)c1c(OC)cccc1OC	0	2.5727151	0	0	0	0	0	0	0	0	0	0	0	0	0
OKWRDLQBKAOJNC-GFCCVEGCSA-N	O(C)c1c2C(=O)O[C@H](C)CCCCCCCc2cc(O)c1	0	2.7821736	0	0	0	0	0	0	0	0	0	0	0	0	0
UGQMRVRMYYASKQ-KQYNXXCUSA-N	O=C1NC=Nc2n([C@H]3[C@H](O)[C@H](O)[C@@H](CO)O3)cnc12	4.8473013	2.7001898	0	0	0	0	0	0	0	0	0	0	0	0	0
YCCILVSKPBXVIP-UHFFFAOYSA-N	OCCc1ccc(O)cc1	0	1.2789197	0	0	0	0	0	0	0	0	0	0	0	0	0
IGWDEVSBEKYORK-UHFFFAOYSA-N	O(CC=C(C)C)c1c2c(occ2)cc2OC(=O)C=Cc12	0	2.5764913	0	0	0	0	0	0	0	0	0	0	0	0	0
MBRLOUHOWLUMFF-UHFFFAOYSA-N	O(C)c1c(CC=C(C)C)c2OC(=O)C=Cc2cc1	0	2.5715729	0	0	0	0	0	0	0	0	0	0	0	0	0
QXKHYNVANLEOEG-UHFFFAOYSA-N	O(C)c1c2occc2cc2c1OC(=O)C=C2	0	2.065776	0	0	0	0	0	0	0	0	0	0	0	0	0
ORHBXUUXSCNDEV-UHFFFAOYSA-N	O=C1Oc2c(ccc(O)c2)C=C1	0	1.2898956	0	0	0	0	0	0	0	0	0	0	0	0	0
BGEBZHIAGXMEMV-UHFFFAOYSA-N	O(C)c1c2c(occ2)cc2OC(=O)C=Cc12	0	2.0654487	0	0	0	0	0	0	0	0	0	0	0	0	0
SYTYLPHCLSSCOJ-UHFFFAOYSA-N	O(C)c1c(O)cc2c(OC(=O)C=C2)c1	3.0254244	1.95986	0	0	0	0	0	0	0	0	0	0	0	0	0
RODXRVNMMDRFIK-UHFFFAOYSA-N	O(C)c1c(O)cc2OC(=O)C=Cc2c1	3.0965587	2.0748424	0	0	0	0	0	0	0	0	0	0	0	0	0
IQUUXFOZWKSLBQ-UHFFFAOYSA-N	O(C)c1c(OC)c2c3c(O)cccc3c(OC)cc2cc1OC	0	2.7836513	0	0	0	0	0	0	0	0	0	0	0	0	0
ILUJQPXNXACGAN-UHFFFAOYSA-N	O=C(O)c1c(OC)cccc1	0	1.1437119	0	0	0	0	0	0	0	0	0	0	0	0	0
AAUQLHHARJUJEH-UHFFFAOYSA-N	O=C(O)c1c(OC)cccc1O	0	1.5815144	0	0	0	0	0	0	0	0	0	0	0	0	0
NGSWKAQJJWESNS-ZZXKWVIFSA-N	O=C(O)/C=C/c1ccc(O)cc1	0	1.3124186	0	0	0	0	0	0	0	0	0	0	0	0	0
GZGGCZADGIBRHT-DQLDELGASA-N	Clc1c(Cl)cc2c([nH]c3C(C)(C)[C@H]4[C@@]5(NC(=O)[C@@]6(N(C5)CCC6)C4)Cc23)c1	0.3082905	2.8350884	0	0	0	0	0	0	0	0	0	0	0	0	0
DXPVAKSJZFQGSS-DQLDELGASA-N	Clc1cc2[nH]c3C(C)(C)[C@H]4[C@@]5(NC(=O)[C@@]6(N(C5)CCC6)C4)Cc3c2cc1	0.3273582	2.826343	0	0	0	0	0	0	0	0	0	0	0	0	0

InChIKey	SMILES	maxHBint3	SpMax8_Bhs	Class (predicted)												
				Split1 (RBF 2-24-2)	Split1 (RBF 2-22-2)	Split2 (RBF 2-27-2)	Split3 (MLP 2-4-2)	Split4 (RBF 2-24-2)	Split5 (MLP 2-4-2)	Split5 (RBF 2-22-2)	Split6 (MLP 2-5-2)	Split7 (MLP 2-4-2)	Split8 (RBF 2-27-2)	Split9 (RBF 2-22-2)	Split9 (RBF 2-28-2)	Split10 (RBF 2-30-2)
VCMZMLWIPPPAOG-UHFFFAOYSA-N	O=C(CCC(=O)c1c(O)cc(C)cc1)c1c(O)cc(C)cc1	0	2.9271257	0	0	0	0	0	0	0	0	0	0	0	0	0
UUEIVUJSJLKAAM-SDQBBNPISA-N	O=C(Oc1c(C(C)C)ccc(C)c1)/C(=C\C)/C	0	2.5663893	0	0	0	0	0	0	0	0	0	0	0	0	0
XRTYDFQPBORLIK-XGICHPGQSA-N	O=C(Oc1c(C2(COC(=O)C)OC2)ccc(C)c1)/C(=C\C)/C	0	2.8785745	0	0	0	0	0	0	0	0	0	0	0	0	0
FGMNYSJXCIZEOU-WAYWQWQTSA-N	O=C(Oc1c(C(C)C)ccc(C)c1)/C=C\C	0	2.4201675	0	0	0	0	0	0	0	0	0	0	0	0	0
LONAZUBXVNGNKH-WZUFQYTHSA-N	O=C(Oc1c(C2(C(=O)C)OC2)ccc(C)c1)/C(=C\C)/C	0	2.7971667	0	0	0	0	0	0	0	0	0	0	0	0	0
MGSRCZKZVOBKFT-UHFFFAOYSA-N	Oc1c(C(C)C)ccc(C)c1	0	1.3810212	0	0	0	0	0	0	0	0	0	0	0	0	0
BNWJOHGLIBDBOB-UHFFFAOYSA-N	O(C)c1c2OCOc2cc(CC=C)c1	0	1.9400984	0	0	0	0	0	0	0	0	0	0	0	0	0
KSEBMYQBYZTDHS-HWKANZROSA-N	O=C(O)/C=C/c1cc(OC)c(O)cc1	3.0980471	2.1920996	0	0	0	0	0	0	0	0	0	0	0	0	0
IQVQXVFMNOFTMU-DHZHZOJOSA-N	O=C1O/C(=C/CCC)/C2=C1C=CCC2	0	2.0372838	0	0	0	0	0	0	0	0	0	0	0	0	0
WMBOCUXXNSOQHM-DHZHZOJOSA-N	O=C1O/C(=C/CCC)/c2c1cccc2	0	1.9807463	0	0	0	0	0	0	0	0	0	0	0	0	0
ZGPJMFUBGMSEDV-CORQQEAWSA-N	O=C(O[C@@H]1[C@]2(C)[C@@H](C)CCC=C2C(=O)c2occ(C)c12)/C(=C\C)/C	0	2.9398145	0	0	0	0	0	0	0	0	0	0	0	0	0
PUBUQGJOCRORHJ-TWSVYZDPSA-N	O=C(O[C@@H]1[C@]2(C)[C@@H](C)CCC=C2C(=O)c2occ(C)c12)[C@H](CC)C	0	2.9679225	0	0	0	0	0	0	0	0	0	0	0	0	0
ZGPJMFUBGMSEDV-OLQHVUIGSA-N	O=C(O[C@H]1[C@]2(C)[C@@H](C)CCC=C2C(=O)c2occ(C)c12)/C(=C\C)/C	0	2.9398145	0	0	0	0	0	0	0	0	0	0	0	0	0
VWUPQZXUHCYORA-ZEQRYJAYSA-N	O=C(O[C@H]1[C@]2(C)[C@@H](C)C[C@@H](O)C=C2C(=O)c2occc12)/C(=C\C)/C	0	3.011359	0	0	0	0	0	0	0	0	0	0	0	0	0
VNNQNPHIASWXBS-AYVTZFPOSA-N	O=C1c2occ(C)c2[C@](O)(C)C=2[C@@H](C)CCCC1=2	0	2.6993367	0	0	0	0	0	0	0	0	0	0	0	0	0
JPMYFOBNRRGFNO-UHFFFAOYSA-N	O(C)c1cc(O)c2C(=O)C=C(c3ccc(O)cc3)Oc2c1	0	2.7659948	0	0	0	0	0	0	0	0	0	0	0	0	0
GHZRKUIAWAOWRH-UHFFFAOYSA-N	O=C(C)c1c(O)c2c(OC(C)(C)C=C2)cc1	0	2.5806982	0	0	0	0	0	0	0	0	0	0	0	0	0

InChIKey	SMILES	maxHBint3	SpMax8_Bhs	Class (predicted)												
				Split1 (RBF 2-24-2)	Split1 (RBF 2-22-2)	Split2 (RBF 2-27-2)	Split3 (MLP 2-4-2)	Split4 (RBF 2-24-2)	Split5 (MLP 2-4-2)	Split5 (RBF 2-22-2)	Split6 (MLP 2-5-2)	Split7 (MLP 2-4-2)	Split8 (RBF 2-27-2)	Split9 (RBF 2-22-2)	Split9 (RBF 2-28-2)	Split10 (RBF 2-30-2)
NPNUFJAVOOONJE-UHFFFAOYSA-N	C=C1C2C(C(C)(C)C2)CCC(C)=CCC1	0	2.3236818	0	0	0	0	0	0	0	0	0	0	0	0	0
NVEQFIOZRFFVFW-UHFFFAOYSA-N	C=C1C2C(C(C)(C)C2)CCC2(C)OC2CC1	0	2.6269794	0	0	0	0	0	0	0	0	0	0	0	0	0
MFIBJICNTUTPCP-UHFFFAOYSA-N	O(C)c1c2C(=O)OC=C3C(OC)=CC(=O)c(c(C)c1)c23	0	2.7772651	0	0	0	0	0	0	0	0	0	0	0	0	0
HBTQKFCMVAYBOL-UHFFFAOYSA-N	O(C)c1c2c(C)cc(OC)c3C(=O)OC(OC)c(c(O)c1)c23	0	2.8532636	0	0	0	0	0	0	0	0	0	0	0	0	0
IYNJGYCVIYBFBS-UHFFFAOYSA-N	O=C1c2c(O)cccc2C(=O)c2c(O)cc(C)cc12	0	2.5930767	0	0	0	0	0	0	0	0	0	0	0	0	0
VYQXIUVIYICVCM-UHFFFAOYSA-N	O(C)c1cc(O)cc(CCc2cc(O)ccc2)c1	0	2.475314	0	0	0	0	0	0	0	0	0	0	0	0	0
MSHFRERJPWKJFX-UHFFFAOYSA-N	O(C)c1ccc(CO)cc1	0	1.2537113	0	0	0	0	0	0	0	0	0	0	0	0	0
POBIEWKRMCMVFH-PKNBQFBNSA-N	O=C(/C=C/C(O)(C)C)C(CCCC(O)(C=C)C)C	0	2.8342205	0	0	0	0	0	0	0	0	0	0	0	0	0
DZESPMMROLVXTM-OKPQQOGWSA-N	O=C1C(=C)C2[C@@H](O1)[C@@H]1[C@](O)(C)C=CC(=O)[C@@]1(C)CC2	0	2.8562829	0	0	0	0	0	0	0	0	0	0	0	0	0
PENSQRMNZZWMGV-UHFFFAOYSA-N	O(C)c1c(C=2C(=O)Oc3c(cc4c(occ4)c3)C=2)cc2OCOc2c1	0	2.8316796	0	0	0	0	0	0	0	0	0	0	0	0	0
RAJDDCCSNZAPCH-ZWKOTPCHSA-N	O=C1[C@H]2[C@@H](Oc3c1cc1c(occ1)c3)COc1c2cc2OCOc2c1	0	2.9221644	0	0	0	0	0	0	0	0	0	0	0	0	0
CIGSWLXZMSXAAE-ZCFIWIBFSA-N	O(C)c1c(C)c2[C@H](C)C(=C)OC(=O)c2c(O)c1	0	2.5199042	0	0	0	0	0	0	0	0	0	0	0	0	0
MSUXGUUSNDNHFC-UHFFFAOYSA-N	O=C1c2c(C)c(O)cc(O)c2C(=O)CC1	0	2.4470636	0	0	0	0	0	0	0	0	0	0	0	0	0
ZXYYTDCENDYKBR-ZETCQYMHSA-N	O=C1c2c(O)cccc2[C@@H](O)CC1	0	2.0383146	0	0	0	0	0	0	0	0	0	0	0	0	0
DANYIYRPLHHOCZ-UHFFFAOYSA-N	O(C)c1ccc(C=2Oc3c(c(O)cc(O)c3)C(=O)C=2)cc1	0	2.7659948	0	0	0	0	0	0	0	0	0	0	0	0	0
NWZKOCPFFSDCBI-OCVOHJKQSA-N	O=C1/C(=C(\C)/C)/[C@H](O)[C@]2(C)[C@@H](C)[C@H](O)CCC2=C1	0	2.7412807	0	0	0	0	0	0	0	0	0	0	0	0	0
TVDMUSYVWJLIDK-PSOPSSQASA-N	O=C1C(=C(C)C)C[C@]2(C)[C@@H](C)[C@H](O)CCC2=C1	0	2.6546797	0	0	0	0	0	0	0	0	0	0	0	0	0

InChIKey	SMILES	maxHBint3	SpMax8_Bhs	Class (predicted)												
				Split1 (RBF 2-24-2)	Split1 (RBF 2-22-2)	Split2 (RBF 2-27-2)	Split3 (MLP 2-4-2)	Split4 (RBF 2-24-2)	Split5 (MLP 2-4-2)	Split5 (RBF 2-22-2)	Split6 (MLP 2-5-2)	Split7 (MLP 2-4-2)	Split8 (RBF 2-27-2)	Split9 (RBF 2-22-2)	Split9 (RBF 2-28-2)	Split10 (RBF 2-30-2)
PCBDXYONDOCJPR-VAEXESPGSA-N	O=C1C2(C(=C)C)O[C@@H]2[C@]2(C)[C@@H](C)[C@H](O)CCC2=C1	0	2.7086435	0	0	0	0	0	0	0	0	0	0	0	0	0
AIVUQNKTJDAYQX-RNJXMRFFSA-N	O(C)C=1[C@H](O)[C@]2(C)O[C@@H]2C(=O)C=1	2.6501259	1.6928583	0	0	0	0	0	0	0	0	0	0	0	0	0
AYIDXPPINFIJKW-ZETCQYMHSA-N	O(C)c1c2C(=O)O[C@@H](C)Cc2ccc1	0	2.142368	0	0	0	0	0	0	0	0	0	0	0	0	0
GUAACJNCCYVZCM-MELADBBJSA-N	O(C)C=1[C@H](O)[C@@](O)(C)[C@@H](Nc2ccccc2)C(=O)C=1	4.3238523	2.7960773	0	0	0	0	0	0	0	0	0	0	0	0	0
AIVUQNKTJDAYQX-BIIVOSGPSA-N	O(C)C=1[C@H](O)[C@@]2(C)O[C@H]2C(=O)C=1	2.6501259	1.6928583	0	0	0	0	0	0	0	0	0	0	0	0	0
YYWUABJYAOCACI-UHFFFAOYSA-N	O=C1N(C)C(Cc2ccccc2)C=2N(C(=O)c3c(N=2)cccc3)c2c1cccc2	0	2.8821236	0	0	0	0	0	0	0	0	0	0	0	0	0
VMRNXWXXUDWZEX-UHFFFAOYSA-N	O=C1NC(=O)C=2C(=O)c3c(N(C)C1=2)cccc3	1.0027611	2.5516814	0	0	0	0	0	0	0	0	0	0	0	0	0
YCGFIOZHNUQYSK-UHFFFAOYSA-N	O=C1NC(C(C)C)C=2N(C)c3c(C(=O)C1=2)cccc3	0.7326779	2.5984869	0	0	0	0	0	0	0	0	0	0	0	0	0
FLHQAMWKNPOTDV-ZANVPECISA-N	O=C1N[C@@H]([C@H](CC)C)C=2N(C)c3c(C(=O)C1=2)cccc3	0.70039	2.7459707	0	0	0	0	0	0	0	0	0	0	0	0	0
FLHQAMWKNPOTDV-TVQRCGJNSA-N	O=C1N[C@H]([C@H](CC)C)C=2N(C)c3c(C(=O)C1=2)cccc3	0.70039	2.7459707	0	0	0	0	0	0	0	0	0	0	0	0	0
PLSSEPIRACGCBO-PFFFPCNUSA-N	O=C1C(=C)[C@H]2[C@H](O1)[C@H]1C(C)=CC[C@@H](O)[C@]1(C)CC2	0	2.7866487	0	0	0	0	0	0	0	0	0	0	0	0	0
WHBSVFFDUQPHNS-UHFFFAOYSA-N	Clc1c(O)c2c(cc1)C=CC(=O)OC2	0.317348	1.5375997	0	0	0	0	0	0	0	0	0	0	0	0	0
AMZNYVFIWCPUAY-UHFFFAOYSA-N	O=C(C)c1c(O)c(C)c(O)c(C)c1	0	1.9767162	0	0	0	0	0	0	0	0	0	0	0	0	0
OMBMYWYFYLOVPT-UHFFFAOYSA-N	O=C1OCc2c(O)cccc2C=C1	0	1.5181637	0	0	0	0	0	0	0	0	0	0	0	0	0
MWZYYACVPIJBPD-SIWOTSRUSA-N	O=C1[C@]2([C@@H](O)C(=C)O1)[C@@H](O)C(=O)C=C[C@H]2C	5.0400506	2.5870186	0	0	0	0	0	0	0	0	0	0	0	0	0
ANHHAPDWNRZSRR-YZLKNSBCSA-N	O=C1[C@]2([C@H](O)C(=C)O1)[C@@H](O)C(=O)CC[C@H]2C	4.7836953	2.6717891	0	0	0	0	0	0	0	0	0	0	0	0	0
VWNBIHJYLBWFKS-UPZJHPNMSA-N	O[C@@H]([C@@H](O)C)C[C@H]1OCc2c(O)cccc12	4.9933761	2.5505524	0	0	0	0	0	0	0	0	0	0	0	0	0

InChIKey	SMILES	maxHBint3	SpMax8_Bhs	Class (predicted)												
				Split1 (RBF 2-24-2)	Split1 (RBF 2-22-2)	Split2 (RBF 2-27-2)	Split3 (MLP 2-4-2)	Split4 (RBF 2-24-2)	Split5 (MLP 2-4-2)	Split5 (RBF 2-22-2)	Split6 (MLP 2-5-2)	Split7 (MLP 2-4-2)	Split8 (RBF 2-27-2)	Split9 (RBF 2-22-2)	Split9 (RBF 2-28-2)	Split10 (RBF 2-30-2)
ZNXZQBCPPUYJBI-UHFFFAOYSA-N	O(C)c1c(C)c2C(=O)C(OC)=CC(=O)c2c(O)c1	0	2.7609826	0	0	0	0	0	0	0	0	0	0	0	0	0
OZEDYANFTHNHQG-UHFFFAOYSA-N	O(C)c1c2C(=O)OC=C3C(OC)=CC(=O)c(c(O)c1)c23	0	2.8081791	0	0	0	0	0	0	0	0	0	0	0	0	0
GUAFOGOEJLSQBT-UHFFFAOYSA-N	O(C)c1c(OC)cc2c(OC(=O)C=C2)c1	0	2.0834306	0	0	0	0	0	0	0	0	0	0	0	0	0
LZERJKGWTQYMBB-UHFFFAOYSA-N	O(C)c1cc(O)c2C(=O)C=C(c3ccc(OC)cc3)Oc2c1	0	2.7606818	0	0	0	0	0	0	0	0	0	0	0	0	0
LIIALPBMIOVAHH-UHFFFAOYSA-N	O(C)c1cc2OC(=O)C=Cc2cc1	0	1.5717322	0	0	0	0	0	0	0	0	0	0	0	0	0
WJUFSDZVCOTFON-UHFFFAOYSA-N	O=Cc1cc(OC)c(OC)cc1	0	1.4905257	0	0	0	0	0	0	0	0	0	0	0	0	0
JSYQPAIDLVPBDA-JWUPAISJSA-N	O=C1C(O)=C(C)[C@@H]2C(C)(C)[C@@H]3[C@@]1([C@H](C)CC3)C2=O	4.9302643	2.6906691	0	0	0	0	0	0	0	0	0	0	0	0	0
JSYQPAIDLVPBDA-RAIZXLCNSA-N	O=C1C(O)=C(C)[C@H]2C(C)(C)[C@H]3[C@]1([C@H](C)CC3)C2=O	4.9302643	2.6906691	0	0	0	0	0	0	0	0	0	0	0	0	0
JZXORCGMYQZBBQ-SNVBAGLBSA-N	O=C1C(O)=C([C@@H](CCC=C(C)C)C)C(=O)C=C1C	5.6475132	2.6097414	0	0	0	0	0	0	0	0	0	0	0	0	0
QQQCWVDPMPFUGF-UHFFFAOYSA-N	O(C)c1c2C(=O)CC(c3ccccc3)Oc2cc(O)c1	0	2.6846329	0	0	0	0	0	0	0	0	0	0	0	0	0
ORJDDOBAOGKRJV-UHFFFAOYSA-N	O(C)c1cc(O)c2C(=O)CC(c3ccccc3)Oc2c1	0	2.6701942	0	0	0	0	0	0	0	0	0	0	0	0	0
RTIXKCRFFJGDFG-UHFFFAOYSA-N	O=C1c2c(O)cc(O)cc2OC(c2ccccc2)=C1	0	2.610088	0	0	0	0	0	0	0	0	0	0	0	0	0
URFCJEUYXNAHFI-UHFFFAOYSA-N	O=C1c2c(O)cc(O)cc2OC(c2ccccc2)C1	0	2.6466126	0	0	0	0	0	0	0	0	0	0	0	0	0
